# Dissecting the Dynamics of HIV-1 Protein Sequence Diversity

**DOI:** 10.1371/journal.pone.0059994

**Published:** 2013-04-04

**Authors:** Yongli Hu, Paul ThiamJoo Tan, Tin Wee Tan, J. Thomas August, Asif M. Khan

**Affiliations:** 1 Perdana University Graduate School of Medicine, Selangor Darul Ehsan, Malaysia; 2 Department of Pharmacology and Molecular Sciences, The Johns Hopkins University School of Medicine, Baltimore, Maryland, United States of America; 3 Department of Biochemistry, Yong Loo Lin School of Medicine, National University of Singapore, Singapore, Singapore; 4 National Public Health Laboratory, Communicable Disease Division, Ministry of Health, Singapore, Singapore; Massachusetts General Hospital, United States of America

## Abstract

The rapid mutation of human immunodeficiency virus-type 1 (HIV-1) and the limited characterization of the composition and incidence of the variant population are major obstacles to the development of an effective HIV-1 vaccine. This issue was addressed by a comprehensive analysis of over 58,000 clade B HIV-1 protein sequences reported over at least 26 years. The sequences were aligned and the 2,874 overlapping nonamer amino acid positions of the viral proteome, each a possible core binding domain for human leukocyte antigen molecules and T-cell receptors, were quantitatively analyzed for four patterns of sequence motifs: (1) “index”, the most prevalent sequence; (2) “major” variant, the most common variant sequence; (3) “minor” variants, multiple different sequences, each with an incidence less than that of the major variant; and (4) “unique” variants, each observed only once in the alignment. The collective incidence of the major, minor, and unique variants at each nonamer position represented the total variant population for the position. Positions with more than 50% total variants contained correspondingly reduced incidences of index and major variant sequences and increased minor and unique variants. Highly diverse positions, with 80 to 98% variant nonamer sequences, were present in each protein, including 5% of Gag, and 27% of Env and Nef, each. The multitude of different variant nonamer sequences (*i.e.* nonatypes; up to 68%) at the highly diverse positions, represented by the major, multiple minor, and multiple unique variants likely supported variants function both in immune escape and as altered peptide ligands with deleterious T-cell responses. The patterns of mutational change were consistent with the sequences of individual HXB2 and C1P viruses and can be considered applicable to all HIV-1 viruses. This characterization of HIV-1 protein mutation provides a foundation for the design of peptide-based vaccines and therapeutics.

## Introduction

The quasispecies replication of RNA viruses has been recognized for over 30 years following the initial observation of the high proportion of mutants in a growing population of bacteriophage Qβ [1]. HIV-1 is now a classic example of this model of rapid genome evolution [2]–[6]. As a result of high rates of genetic mutation [7] and recombination [8], cell infection by HIV-1 is followed by immune escape of viruses with related and highly diverse genotypes [9], [10]. Even a single founder virus, when introduced into cells, is quickly transformed into a quasispecies assortment of progeny viruses [11]–[13]. Given the vast array of different genotypes generated in every replication cycle, the challenge of designing a vaccine that would prevent the immune escape of the mutant progeny of infected cells is widely recognized [14]–[17]. A continuing goal is a greater understanding of HIV-1 diversity and an effective strategy to overcome this diversity. Towards this end, there is a need for more detailed and quantitative analysis of the extent of HIV-1 mutational changes, including the composition and incidence of the different variants of the viral proteome.

Herein, we describe a large-scale analysis of the diversity of HIV-1 sequences reported over at least 26 years. Clade B was selected for the developmental studies as it had the largest number of recorded HIV-1 sequences. Over 58,000 clade B sequences, both partial- and full-length and distributed among the nine proteins, were aligned, with over 1,000 sequences at most nonamer positions (Table S1). As the immune-relevance of the sequences was a primary focus of the study, the analysis was conducted with 2,874 nonamer positions, overlapping eight residues (1–9, 2–10, *etc*.), where each nonamer at the positions is a potential viral human leukocyte antigen (HLA) binding core [18] and T-cell receptor (TCR) ligand [19]. As expected, there were major differences in the fractions of variant nonamer sequences of the proteins, with more conserved positions in Gag and Pol, and greater diversity in Env and Nef. However, despite the differences in the structure and function of the proteins, or the extent of their genomic diversity, the viral proteins shared characteristic patterns of change in the incidence of index, major, minor, and unique nonamer motifs with increased mutation. This quantitative characterization of the mutational changes of the clade B proteome provides data that are applicable to the design of vaccines and therapeutics that are least impacted by the diversity of the viral proteins. HIV-infected individuals, “elite controllers”, who maintain low levels of plasma virus [20]–[22], as well as animal model experiments [23]–[27] suggest that vaccines that generate CD8+ T cells, even against only a few epitopes, can result in a long-term control of virus proliferation. We suggest that an effective immune response to HIV-1 in humans might be possible with a vaccine comprised solely of the limited set of highly conserved protein sequences, as described herein, that may provide memory responses not compromised by immune escape or the presence of competing altered peptide ligands.

## Materials and Methods

### Data Preparation, Selection and Alignment of HIV-1 Clade B Proteome

HIV-1 protein sequence records were retrieved from the NCBI Entrez Protein Database [28] in August 2008 by searching the NCBI taxonomy browser for HIV-1 (Taxonomy ID 11676). HIV-1 clade B records were extracted from the collected data by use of BLAST [29]–[32] searches (version 2.2.18; parameters: low complexity filter – off, expect –10, descriptions and alignments –100,000), using the HIV-1 clade B protein reference sequences from the Los Alamos HIV sequence database (www.hiv.lanl.gov) as queries (Table S2). Cutoff for the classification of each clade B protein was determined by manual inspection of the BLAST output. Possible misclassification of non-clade B HIV-1 sequences as clade B was not apparent as the patterns of motif change were similar for each protein, particularly for Gag, Pol, and Env proteins that form the major basis of phylogenetic relationships [33], [34]. Additonal phylogenetic study for classification of the sequences as clade B was not practical given the high viral diversity, large number of sequences analysed, and the difficulty/subjectivity in the interpretation of the phylogenetic tree due to likely low bootstrap values. Duplicate sequences of each protein were removed to minimize bias that may result from collection of redundant protein sequences derived from identical HIV-1 isolates. Highly similar sequences were retained because arbitrary removal of such sequences would introduce additional bias. Further selection of one sequence per subject was deemed unnecessary as it would not give a true representation of the HIV-1 viral diversity as a complex quasispecies of genetically related but distinct sequences in individual subjects. Thus, all non-redundant sequences, both full-length and partial, were aligned and analyzed. Partial sequences were included in the alignment because they provided additional data for the study of diversity. Multiple sequence alignment was difficult for some of the proteins because of the large number of diverse and partial sequences, and thus different approaches were explored. Sequence alignments of Vif, Vpr and Vpu were performed with PROMALS3D [35]. The large sequence datasets of Gag, Pol, Tat, Rev, Env, and Nef were first split into smaller and more manageable groups (about 200–500 sequences per group). These smaller groups were aligned by use of PROMALS3D or CLUSTAL W [35], [36] and refined by use of RASCAL [37]. They were then merged into full protein multiple sequence alignment, with conserved sites as anchors to facilitate reliable alignment of the highly diverse positions (Figure S1). All multiple sequence alignments were manually inspected and corrected for misalignments. Variation in the number of collected sequences between adjacent positions of the alignment resulted from the inclusion of partial sequences. Alignment positions with high fractions, 95% or more, of gaps (insertions or deletions) were removed in order to minimize alignment errors. Because of its great diversity, only 9,661 of the 29,211 extracted Env protein sequences were successfully aligned and analyzed. The selection of these Env sequences was semi-selective, however, the large number of sequences aligned and analyzed help to reduce sampling bias.

A caveat is the unknown history of the many recorded viral sequences; for example, the source of the virus, infection history, possible treatment, or details of cultivation. In addition, the quality of the sequences from the database is also unproven. Nevertheless, it is unlikely that analysis of another population of HIV-1 would differ significantly from the data herein. This assertion is supported by the sequences of individual infectious clade B viruses, HXB2 [38] and C1P [39], that have nonamer position patterns of conservation and variability consistent to those of the historically recorded viruses.

### Nonamer Sequence Analysis Approach

HIV-1 clade B sequence alignment diversity was based on a sliding window approach of size nine, overlapping eight amino acids (1–9, 2–10, 3–11, *etc.*) that captured the entire nonamer repertoire of the proteome [40], [41]. Nonamer sequences represent the possible core HLA and TCR binding domains, and provide a defined examination of the protein diversity in relation to cellular immunity. It can be assumed that each nonamer is a potential T-cell epitope because of the large array of HLAs with different binding specificities in the human population (HLA Informatics Group [http://www.anthonynolan.org.uk/HIG]) and given that an average of 0.1–5% of overlapping nonamers spanning a protein will bind to a particular HLA molecule [42]. Moreover, in this context, the potential immune relevance of a mutation is greatly expanded whereby a single amino acid substitution affects nine overlapping nonamers spanning a region of 17 amino acids, except mutations occurring in the first and last eight amino acids of the protein termini (Figure S2). These features of immune-relevant diversity would not be captured by variability analysis of sequence alignments with a sliding window of size one (single amino acid approach). Further, mutational entropy is completely different between these two approaches, with a maximum entropy of 39 for nonamer approach versus 4.2 for single amino acid approach.

### Shannon’s Nonamer Entropy and Quantitative Analyses of Diversity Motifs

**Figure 1:**
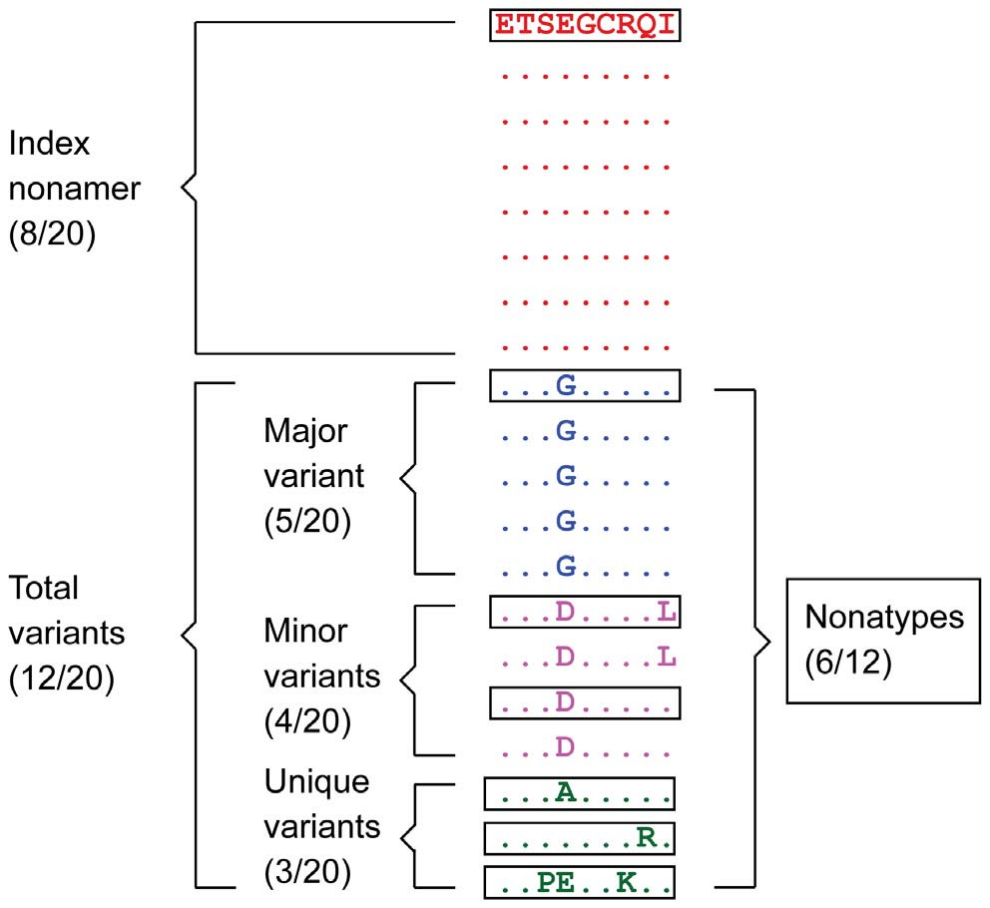
Definitions of HIV-1 clade B nonamer sequence motifs. The different sequence motifs of the aligned HIV-1 clade B isolates were identified as shown with 20 sequences of a model nonamer position. The “Index” nonamer is the most prevalent sequence, present in 8 of the 20 isolates. The “Major” variant is the most common variant of the index (5/20). “Minor” variants are multiple different repeated sequences, each with incidences less than that or occasionally equal of the major variant. “Unique” variants are those represented by a single aligned sequence. “Nonatypes” (boxed) are the distinct variant sequences at a given nonamer position; in this example one of major, two of minor, and three of unique.

Shannon’s nonamer entropy [31], [43], [44] was used as a general measure of HIV-1 protein sequence diversity. Each nonamer position in the protein alignments was quantitatively analysed for the incidence (% occurrence) of the different sequences present at the position, with arbitrary sorting for equal incidences (Figure 1). The first ranked sequence of each position was defined as the index, while the remaining ranked sequences with at least one amino acid difference from the index were defined as variants and organized as three motifs: (1) “major” variant, the most prevalent individual variant of the index; (2) “minor” variants, multiple different sequences, each present in two or more of the aligned viral sequences, and each with an incidence less than or equal (occasionally) to that of the major variant; and (3) “unique” variants, each unique to a single viral isolate. The combined incidence of the major, minor and unique variants at a nonamer position was inversely related to the corresponding incidence of the index sequence and represented the total variant population at the position. The incidence of distinct variant nonamer sequences at a given nonamer position (nonatypes) was also determined. Further, the correspondence of the index sequences to the sequences of the original clade B isolate, HXB2 [38] and an arbitrarily selected recent strain, C1P [39] was evaluated. HXB2 is considered the clade B prototype sequence [45], while C1P is an infectious virus cloned full-length from residual viral RNAs recovered from the plasma of a HAART-treated patient and sequenced by modern methodologies.

Variant nonamers that contained gaps (-) or any one of the unresolved characters, including B (asparagine or aspartic acid), J (leucine or Isoleucine), X (unspecified or unknown amino acid), and Z (glutamine or glutamic acid) were excluded from the quantification. All data analyses were performed with the 2,874 aligned nonamer positions of the proteome that had more than 100 sequences (out of 3,133 total aligned proteome nonamer positions). However, data for positions with less than 100 sequences are shown in some figures and tables to maintain the protein sequence structure.

## Results

### Shannon Entropy Overview of HIV-1 Clade B Proteome Sequence Diversity

**Figure 2:**
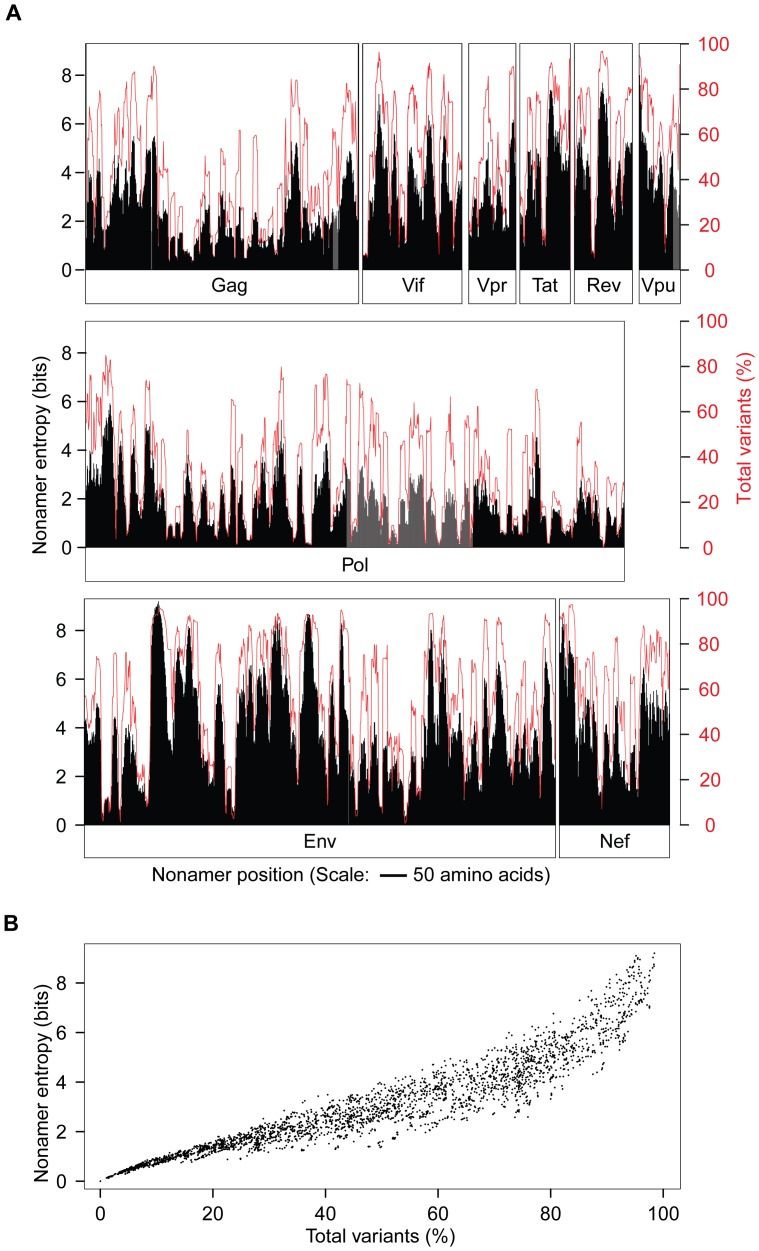
HIV-1 clade B proteome nonamer sequence entropy and total variants. A. Entropy (in black) and incidence of the total variants of the index (in red) was measured for each aligned nonamer (nine amino acids) position (1–9, 2–10, *etc.*) of the proteins. The entropy values indicate the level of variability at the corresponding nonamer positions, with zero representing completely conserved sites (0% total variants incidence), with a maximum of about 9 at the extremely variant sites (∼98% total variants incidence). **B.** Relationship of entropy and the incidence of total variants for the proteome nonamer positions.

Shannon entropy [31], [43], [44] of the aligned nonamer positions was used as a measure of overall sequence diversity of the HIV-1 clade B proteome (Figure 2A). The remarkable complexity of the viral protein sequence structure was evident from the extensive presence of high entropy nonamer positions, with a large fraction of variant sequences in each of the HIV-1 proteins, including Gag and Pol, the most conserved. The maximum evolutionary entropy (9.2) of HIV-1 nonamer sequences far exceeded that of ∼6 for avian influenza [32], ∼4 for dengue [31], and ∼2 for West Nile virus [30]. Each of the HIV-1 proteins was found to contain positions with greater than 50% variant sequences, and some with variants as high as 98% of the aligned nonamer sequences. A severe discordance in the relationship between entropy and the incidence of variants of the nonamer positions was also evident (Figure 2B). While high entropy is a general indication of high diversity, several positions of lower entropy (<2.0) contained high incidences of variants, some comparable to those of higher entropy regions (>4.0). In general, only positions with variants incidence of less than 20% had a relatively linear relationship with entropy. In contrast, positions with more than 20% variants displayed a wide range and non-linear rise in entropy that increased greatly at positions with an incidence of variants ≥80%. The wide range of entropy can be attributed to the composition and incidence of the variant motifs that comprised of many different sequences.

### Dissecting HIV-1 Clade B Proteome Sequence Diversity: Scope of the Analysis

Dissecting protein sequence diversity of HIV-1 clade B included quantitative analyses of diversity motifs of each of the 2,874 aligned nonamer positions of the proteome (Table S3). An example of this data is shown with 27 aligned overlapping nonamer positions of Env 114–148 (Table 1). Each of the 27 aligned nonamer positions contained about 1,000 to 3,900 sequences. The first five positions (Env 114–122 to 118–126) were relatively conserved with entropies of about 1.0, and index nonamers with incidences of 85% to 89%. These conserved sequences were identical to the corresponding sequences of the HXB2 and C1P. The remaining small fraction (11 to 15%) of the aligned sequences at these conserved positions were variants of the index nonamers and represented by about equal fractions of the major and minor variants (5–7%), and a minimal fraction, ∼1 to ∼2%, of unique sequences. The incidence of the different variant nonamer sequenes (nonatypes) at these positions was about 2%, represented by the single major variant, different minor variants, and unique variants. In contrast, the remaining nonamer positions, Env 119–127 to 140–148, included positions of high diversity, with entropy as high as about 9.2, as few as 2% index sequences, 98% as variants of the index, and nonatypes as high as 36%, based almost totally on different minor and unique variants. As an indication of the high variability of these positions in the HIV-1 population, none of the approximately 2,000 to 4,000 sequences of the aligned population contained nonamers that were identical to those of C1P, and only a small fraction (1% or less) corresponded to HXB2 nonamers.

**Table 1:** A sample of the quantitative analysis of HIV-1 clade B Env protein diversity∧.

Aligned nonamers			H(x)^c^	Index^d^		Variants^e^				Nonatypes^i^	HXB2^j^		C1P^k^	
Position^a^		No.^b^		Sequence	[%]	Total	Major^f^	Minor^g^	Unique^h^		Sequence	[%]	Sequence	[%]
						[%]								
^&^	114–122	1032	1.0	SLKPCVKLT	86	14	6	7	1	2	.........	86	.........	86
^&^	115–123	1034	1.0	LKPCVKLTP	86	14	6	7	1	2	.........	86	.........	86
^&^	116–124	1066	1.1	KPCVKLTPL	85	15	6	8	2	3	.........	85	.........	85
^&^	117–125	1517	0.8	PCVKLTPLC	89	11	4	6	1	2	.........	89	.........	89
^&^	118–126	1568	0.8	CVKLTPLCV	89	11	5	5	1	2	.........	89	.........	89
^&^	119–127	1594	1.1	VKLTPLCVT	86	14	5	7	1	2	........S	1	.........	86
^&^	120–128	2665	1.0	KLTPLCVTL	88	12	4	7	1	2	.......S.	1	.........	88
^&^	121–129	2670	1.7	LTPLCVTLN	76	24	5	17	1	2	......S.K	<1	.........	76
^&^	122–130	3112	1.9	TPLCVTLNC	74	26	5	20	1	2	.....S.K.	1	.........	74
^&^	123–131	3326	2.6	PLCVTLNCT	66	34	4	28	2	4	....S.K..	1	........I	2
^&^	124–132	3368	4.1	LCVTLNCTD	44	56	13	40	3	6	...S.K...	1	.......I.	1
^+^	125–133	3673	6.2	CVTLNCTDN	12	88	10	72	6	11	..S.K...L	1	......I.V	<1
^+^	126–134	3675	7.4	VTLNCTDNW	10	90	3	79	8	15	.S.K...LK	1	.....I.VN	<1
^+^	127–135	3677	7.8	TLNCTDNWN	9	91	3	78	9	19	S.K...LK.	1	....I.VNI	0
^+^	128–136	3719	8.3	LNCTDNWNN	6	94	2	81	11	22	.K...LK.D	1	...I.VNIT	0
^+^	129–137	3725	8.3	NCTDNWNNT	6	94	2	79	12	24	K...LK.D.	<1	..I.VNITN	0
^+^	130–138	3912	8.6	CTDNWNNTG	6	94	2	79	13	25	...LK.D.N	1	.I.VNITNT	0
^+^	131–139	3917	8.7	TDNWNNTGN	5	95	2	79	14	27	..LK.D.NT	1	I.VNITNT.	0
^+^	132–140	3911	8.9	DNWNNTGNV	5	95	2	78	15	28	.LK.D.NTN	<1	.VNITNT.S	0
^+^	133–141	3863	8.8	NWNNTGNVS	5	95	2	77	16	29	LK.D.NTN.	<1	VNITNT.ST	0
^+^	134–142	3838	9.0	WNNTGNVSD	5	95	2	77	16	30	K.D.NTN.S	<1	NITNT.STN	0
^+^	135–143	3807	9.0	NNTGNVSDS	5	95	2	76	17	31	.D.NTN.S.	1	ITNT.STNP	0
^+^	136–144	3747	9.0	NTGNVSDSS	5	95	2	76	18	32	D.NTN.S.G	1	TNT.STNPT	0
^+^	137–145	3701	9.1	TGNVSDSSW	5	95	2	75	18	32	.NTN.S.GR	1	NT.STNPTS	0
^+^	138–146	3473	8.9	GNVSDSSWK	4	96	1	76	19	34	NTN.S.GRM	1	T.STNPTSS	0
^+^	139–147	3090	9.2	TSVNSNSSG	2	98	1	78	19	35	.NSS.GRMI	1	N.T.PT..W	0

**Figure 3:**
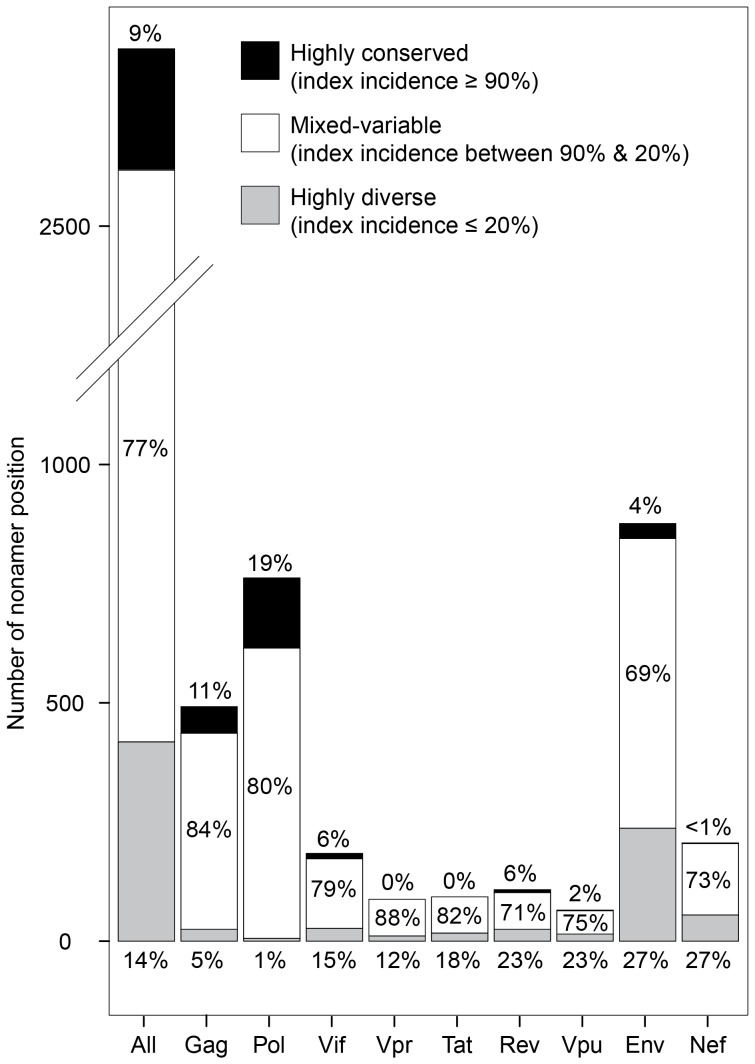
Distribution of conservation level of nonamer positions. The nonamer positions of the proteome and the individual proteins were defined as highly conserved (black, index incidence ≥90%), mixed-variable (white, index incidence <90% & >20%), and highly diverse (grey, index incidence ≤20%). Highly conserved positions were only 9% of total proteome nonamer positions, ranging from 0% for Vpr and Tat, to 19% for Pol. Highly diverse positions comprised 14% of the proteome, ranging from 1% Pol to 27% Env and Nef, each. Mixed-variable positions comprised 77% of the proteome, ranging from 69% Env to 88% Vpr.

∧ All percentages are shown to the nearest whole number.

a Amino acid number at the start and end of the nonamer position in the protein alignment. The symbols ^&^ and^+^denote the mixed-variable and highly diverse nonamer positions, respectively (see Figure 3 for definitions).

b Total number of protein sequences analysed at the respective nonamer position; the differences between nonamer positions was due to the inclusion of both partial and full-length sequences in the alignments.

c Shannon nonamer entropy (H(x); see Figure 2 for details).

d The index nonamer is the most prevalent sequence at the given aligned nonamer position.

e Variants differ by one or more amino acids from the index sequence.

f The major variant is the most common variant sequence at the position.

g Minor variants are multiple different repeated sequences, each occurring more than once and with an incidence less than or occasionally equal to the major.

h Unique variants are those that occur only once in the alignment.

i Nonatypes are the distinct sequences among the variants.

j HXB2 nonamer sequence and its incidence at the given nonamer position; amino acids identical to the index are denoted as “.”.

k C1P nonamer sequence and its incidence at the given nonamer position; amino acids identical to the index are denoted as “.”. HXB2 and C1P nonamers not found at the aligned nonamer position are indicated with 0% incidence.

### Distribution of Conserved and Variable Nonamer Positions in HIV-1 Clade B Proteome

Nearly all nonamer positions of the aligned viral proteins contained variants with one or more mutations. Only two Pol positions with more than 100 sequences in the alignment (Pol 956–964, 957–965) were evolutionarily completely conserved among the recorded sequences that were analyzed in this study. Highly conserved positions with index sequences in 90% or more of the aligned viruses represented only 9% of the proteome, mostly in Pol, Gag, and even Env (Figure 3). The remaining small fraction of variant nonamers, 10% or less, of these highly conserved positions generally contained one amino acid mismatch to the index (median average; data not shown). Highly diverse positions, with 80% or more total variants, represented 14% of the proteome and 23 to 27% of the Env, Nef, Vpu and Rev sequences. The variant sequences of these highly diverse positions differed greatly from the index sequences, many with multiple amino acid mismatches (median average of 3), contributed largely by the unique variants. Overall, a large fraction (91%) of the proteome nonamer positions contained more than 10% variants of the index sequence (*i.e.* mixed-variable and highly diverse nonamer positions).

### Dynamics of Variant Motifs with Increased Mutation

**Figure 4:**
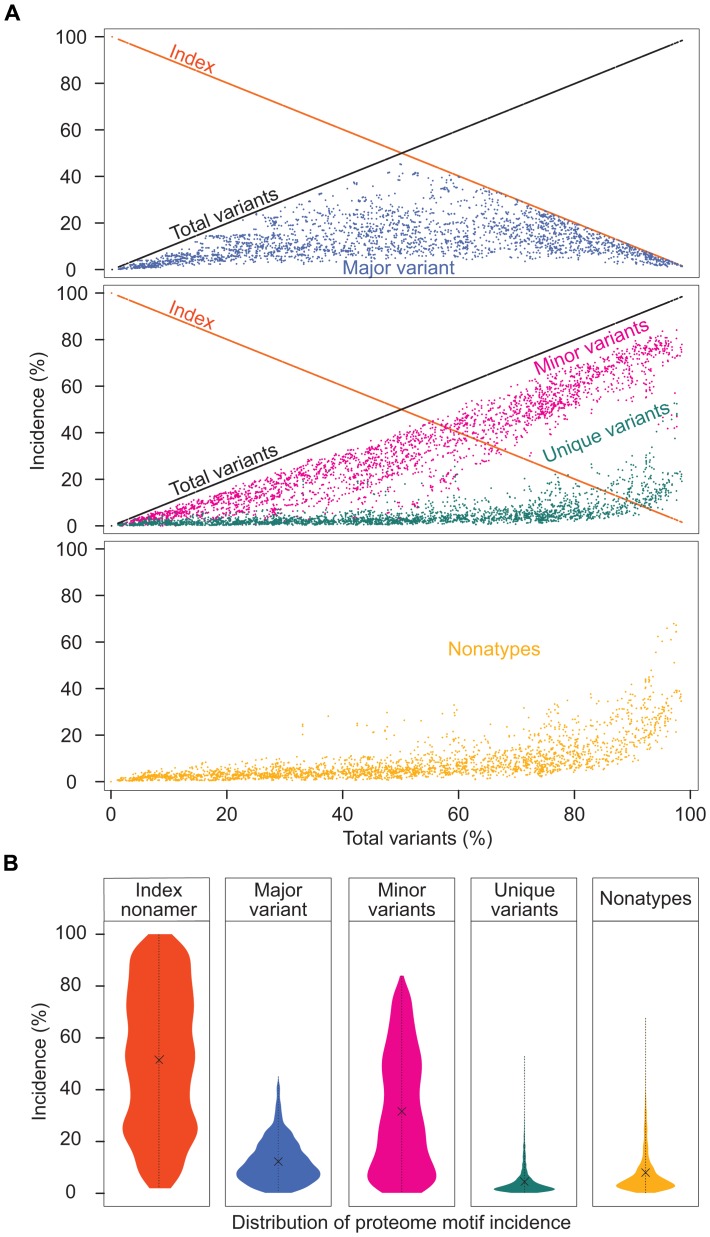
Dynamics of diversity motifs of HIV-1 clade B proteome. **A.** Motif incidence in relation to total variants incidence: index sequence (orange), total variants (black), major variant (blue), minor variants (pink), unique variants (green), and nonatypes (yellow). **B.** Violin plot of the frequency distribution of the indicated proteome sequence motifs. The width of the plot (x-axis) represents the frequency distribution of a given incidence of the indicated motif. “**x**” represents the arithmetic mean incidence value.

A singular finding was the distinctive dynamics of the individual variant motifs of the HIV-1 clade B proteome in relation to increased mutations (Figure 4A). The extreme range in the conservation decrease represented by incidences of index sequences, 100% to 2%, was inversely matched by the increase of total variants incidences, 0% to 98%. Although these inversely related incidences of the index and total variants appear to be in a continuum, drawn as a line (Figure 4A), the data depicted were the actual values of the 2,874 nonamer positions examined. The three variant motifs, each exhibited a characteristic distribution of incidence change with increased total variants. The major variant had a distinctive pyramidal incidence pattern based on the fact that, by definition, the major variant could not exceed the index sequence. Thus, at nonamer positions with greater than 50% total variants, there was a corresponding reduced incidence of both the index sequence and the major variant. However, although the mean incidence of the major variant was only about 10%, there were many positions with over 50% total variants that contained almost equal incidence of the index and major variant. It is apparent that index, antigen epitopes of highly diverse proteomic positions have little quantitative dominance as an immune target over the major variant. The minor variants, each with individual incidence less than or occasionally equal to that of the major variant, were collectively the predominant variant motif for majority of positions, particularly those with greater than 50% total variants. It is evident that the minor variants are a highly dynamic population resulting from fitness selection support in the continued mutation of the index sequences and major variants. The multiple different sequences of the minor variants present additional great variety of potential epitope sequences as immune escape variants or altered peptide ligands. The remaining population of variant nonamer sequences comprised of unique variants, each representing a single viral genotype within the recorded population. Unique variants were distinctive for their presence at almost every nonamer position, including the highly conserved, but with low incidences (average of 3%), despite the increase of other variant sequences. Notably, at positions of greater than about 60% total variants, the incidences of unique variants for some positions were dramatically increased in each of the proteins, particularly in Nef with a maximum incidence of 53%. The increased observation of unique sequences represented positions of hyper-variability, with index incidence of less than or equal to 10% and the remaining 90% or more variants as mostly different minor and unique nonamer sequences. The presence of these unique variants in every protein and at nearly all nonamer positions (Table S3), even those highly conserved, indicates that they are not artifacts of sequencing or result of truncated partial sequences (*e.g.* due to premature stop codon). Nonatypes, a measure of distinct variant sequences (Figure 4A), comprised a large fraction (up to 68%) of the highly diverse positions, composed largely of different minor and unique variants.

The incidence distributions of the index and variant motifs for the proteome are depicted by use of violin plots (Figure 4B). Overall, the index nonamer was the principle motif, but with a mean incidence of only about 52%. The predominant mutation of the index was the minor variant motif, with an incidence distribution inversely related to that of the index. The majority of the major and unique variants were of low incidence, as also shown in Figure 4A, but with a fraction of high incidence unique variants, particularly at positions of ≥80% total variants incidences (*i.e.* highly diverse positions). The nonatypes, composed of the single major variant, the different minor variants, and each of the singular unique variants at a nonamer position, were also chiefly of low incidence, except at highly diverse positions due to the great increase in unique variants incidences. The increased incidence of different variants at highly diverse positions provides an extreme repertoire of possible immune escape variants for a quasispecies population.

**Figure 5:**
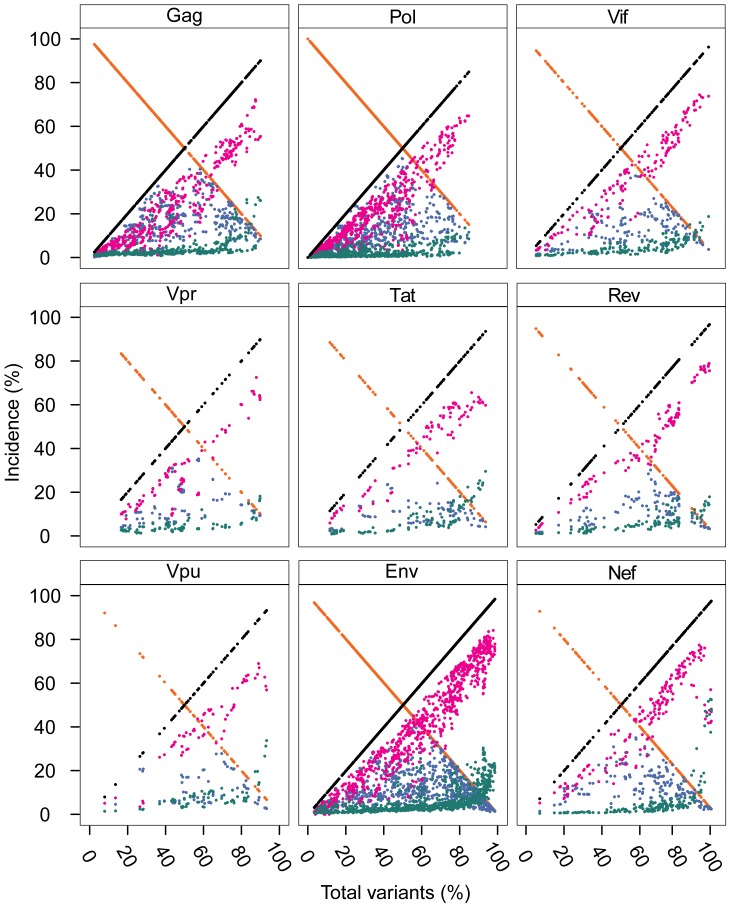
Dynamics of diversity motifs of HIV-1 clade B proteins. The color key for each sequence motif is described in Figure 4.

**Figure 6:**
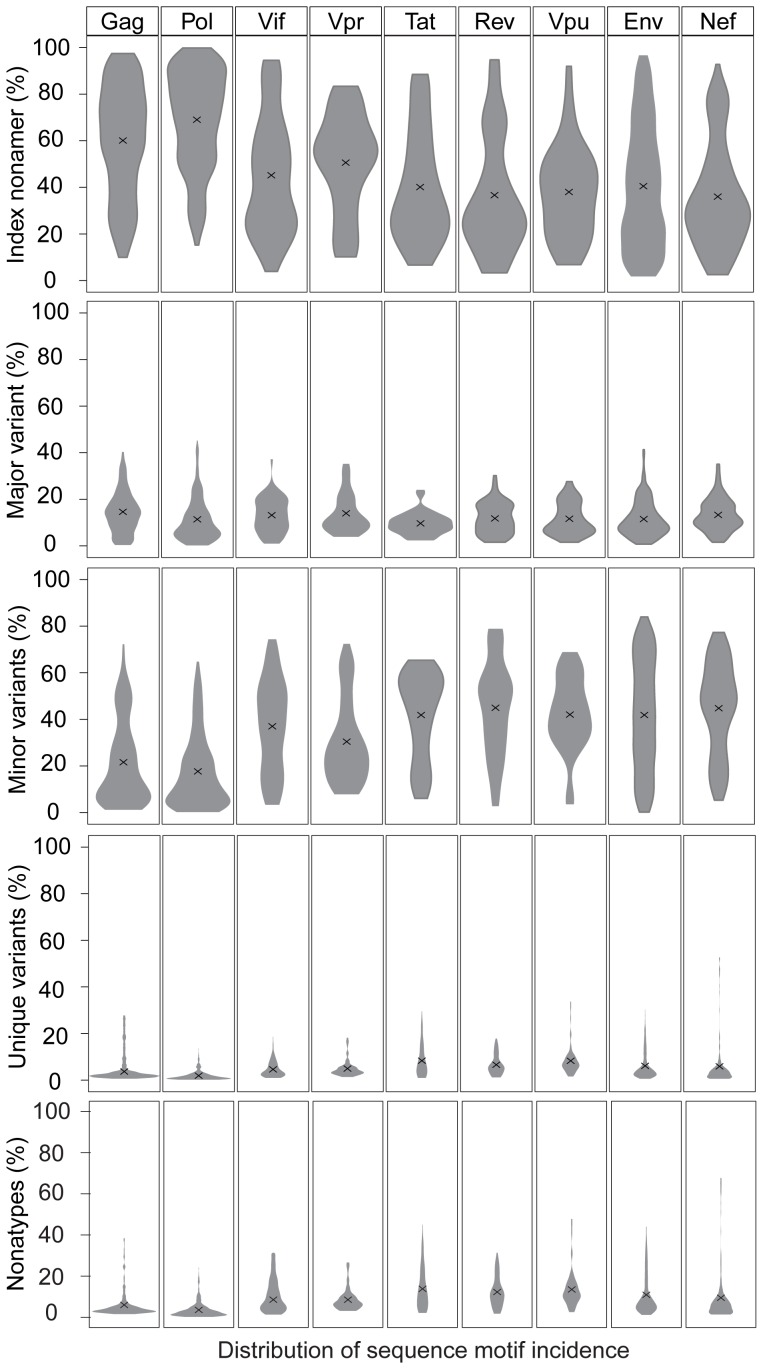
Frequency distribution violin plots of the diversity motifs incidences of HIV-1 clade B proteins. The legend for the violin plot is described in Figure 4.

An additional salient finding was that the dynamics in the changes of the incidence patterns of the variant motifs were repeated for each of the individual proteins, as most evident with the large number of nonamer positions of Gag, Pol, Env and Nef (Figure 5). The only distinct differences between the individual proteins were the fraction of nonamer positions with low incidences of variants (Gag and Pol), compared to those with high incidences of variants (Env and Nef) (Figure 6). Thus, despite the differences in the structures and functions of the proteins, the motifs and incidences of the variants of the index sequences were basically the same for each protein, with similar dynamics in the patterns of change with increased mutation.

### Correspondence of the Index Sequences to those of HXB2 and C1P Proteins

**Figure 7:**
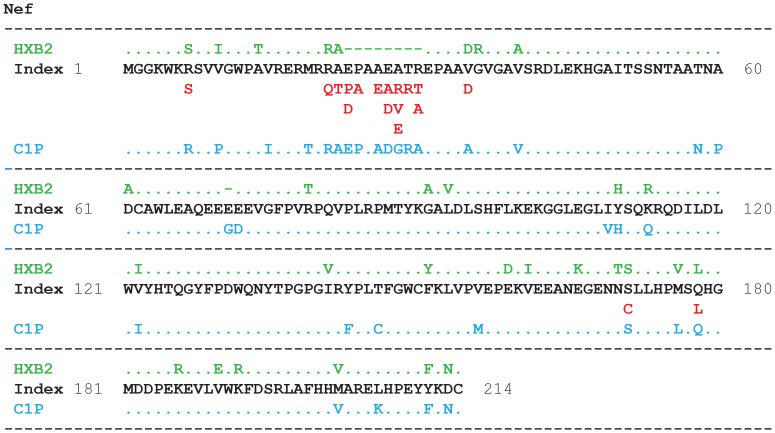
Comparison of HIV-1 clade B Nef concatenated index sequence with the HXB2 and C1P sequences. The numbers before and after the concatenated index sequence represent amino acid positions of the comparison; the comparison is shown in blocks of 60 amino acids. Identity of the index amino acids with those of HXB2 (green) and/or C1P (blue) is represented by “**.**”; those that differ are shown by the respective amino acid. Amino acid mutations of the aligned viruses that did not follow the concatenation of the index are shown in red. The corresponding amino acids of HXB2 and C1P sequences are also shown at these positions, without representing identical residues by “**.**”. The green dashes represent amino acid deletions in HXB2.

The correspondence of HIV-1 clade B index nonamer sequences herein defined, concatenated as full-length proteins, was compared to Nef (Figure 7) and the other proteins (Figure S3) of the individual HXB2 and C1P viruses. There was a high single amino acid identity (>82%) between the historical index and the individual sequences of these viruses (Table S4A), with differences that included a small fraction of indels (insertions/deletions), besides mutations (Figure 7). In contrast, the nonamer sequence identity was much less than that of single amino acids, with only 61% of the HXB2 and 49% of the C1P nonamers identical to the index sequences (Table S4B). As expected, the match between the index and the individual HXB2 and C1P sequences occurred mainly at the highly conserved nonamer positions, and almost none at the highly diverse positions (Table S3), highlighting the consistency of sequence changes in the viral population. Based on this, approximately half of the overlapping nonamer sequences of a given HIV-1 strain can be expected to differ from the historical index sequences of this study.

### Index Switching

**Figure 8:**
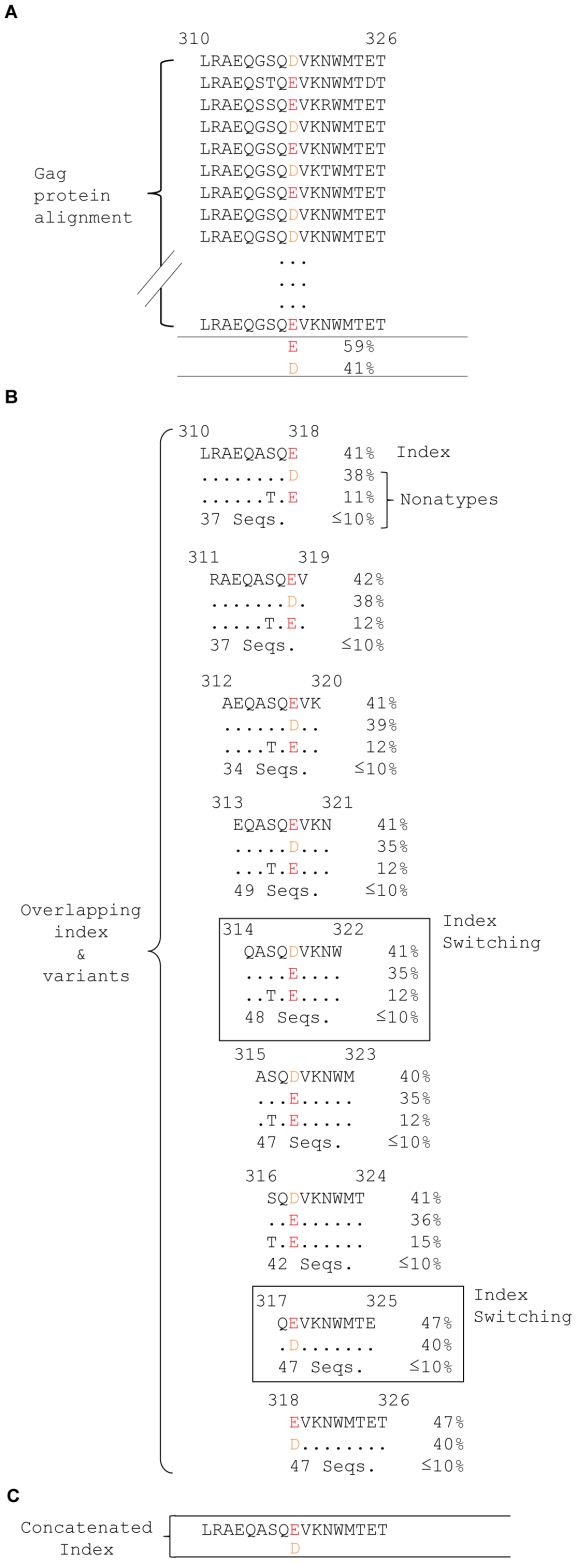
Index switching. **A.** Gag protein alignment region of amino acid (aa) positions 310 to 326 is shown for the first and last five sequences of the aligned viruses. The aa position 318 (coloured) is involved in index switching and includes two aa, the prevalent E observed in 59% of the sequences, with its variant D in 41%. **B.** The nine aligned overlapping nonamer positions (310–318, 311–319, *etc*.) represent the sliding windows of the alignment region in A. Each nonamer position is shown with the index sequence, two of the variant nonatype sequences and the total number of remaining variant nonatypes of incidence equal or below 10%. The first nonamer position, 310 to 318, is shown with the index sequence (41%) containing aa E at the 9^th^ position. The major variant (38%) contains the variable aa D at the corresponding position. A minor variant (11%) has a mutation at aa position 7 relative to the index. A total of 37 nonatypes had individual incidences less or equal to 10%. The dominance of aa E in the index is maintained for the next few nonamer positions. At position 314 to 322, index switching is observed, where the sequence with aa D is now the index (41%) and the one with E is the major variant (35%). This relationship continued till position 316–324, with reversal of sequence ranks to the original state at position 317–325, where the sequence with aa E is the index. At an index switching position, the index is alternative to that expected, relative to the preceding position. **C.** Concatenated index, formed by linking the overlapping index sequences of the nine nonamer positions. At position 318 (coloured), the aa D, which did not follow the concatenation of the index is shown below the sequence.

Index switching, another relevant finding, results from similar incidences of the index sequences and major variants at some mixed-variable and several highly diverse nonamer positions where the incidences of the major variants (average of 8%) were almost indistinguishable from the index sequences (average 12%). Consequently, there were a significant number of nonamer positions in the proteome where fitness increase of one or more amino acid mutations were sufficient to change the incidence rank of a given variant nonatype as the index, alternative to the expected index sequence based on the preceding position (Figure 8). Index-switching positions were readily revealed by amino acid mutations that did not follow the consecutive concatenation of the index sequences, as shown by the red labeled residues in Figures 7 and S3. Index switching may also occur with the other variant motifs and likely represents a subset of a larger phenomenon, motif switching that may be common in a quasispecies population, where the members of the motifs dynamically swap ranking as mutation accumulates. This is likely particularly so at the hyper-variable nonamer positions where the difference in the incidences of the different nonatypes is almost negligible.

## Discussion

This study has elucidated the HIV-1 variant nonamer sequence structure and incidence with increased mutation of the clade B virus proteome. The virus samples, collected over at least 26 years (1983 to 2008, based on records with available isolation date), likely included many differences in mode of HIV transmission, stage of infection, treatment, co-infection, *etc*. The results thus provide a compendium of the possible spectrum of sequence variants of HIV-1 under many different conditions and a model of the collective evolution of multiple HIV-1 populations. It was apparent that virtually all nonamer positions were capable of generating multiple variants of index sequences for the cooperative fitness of protein structure; only two Pol positions were completely conserved. The remarkable plasticity of the virus was demonstrated both by incidences of the three variant motifs at the different nonamer positions, and by the presence of numerous variant nonatypes (distinct sequences), especially at the highly diverse positions. Such regions of high diversity were present in each protein, Gag and Pol included, but most commonly in Rev, Vpu, Env, and Nef, where 23 to 27% of the aligned nonamer positions contained 80% or more of sequences that were variants of the index sequence. The incidence of nonatype sequences at the highly diverse positions was remarkable; for example, about 70% in Nef. Importantly, the reliability of this collective evolutionary diversity was supported by the consistency with the individual patterns of mutational change of HXB2 and C1P viruses.

Despite the many differences in structure and function of the HIV-1 proteins, the change of incidence of the major, minor and unique variants with increased sequence change was essentially the same for each protein, differing only in the relative fractions of conserved and diverse sequences. An interpretation of these data is that the three variant motifs represent inherent patterns in the organization of a vast number of variable protein sequences that facilitate HIV-1 fitness-selection. By definition, as the fraction of total variants of the nonamer positions increased, the minor variants became the predominant form, particularly as they replaced both the index and corresponding major variant at positions with more than 50% total variants. The large fractions of nonatypes at highly diverse positions is in keeping with the quasispecies model, that progeny viruses contain virtually any sequence change consistent with the cooperative fitness of protein sequences [2]. This pattern of sequence change was hypothesized by Eigen [46] to have resulted from a combination of Darwin’s selective evolution of the dominant replicator and the presence of a “clan” of variant molecules that also have maximum reproductive fitness. The extreme sequence plasticity could provide a spectrum of viral variants that allow the virus population to efficiently exploit changes in selection pressure and facilitate the long-term evolutionary stability and versatility of the virus [46].

The intense repertoire of variants in the mixed variable and highly diverse regions of the virus proteome likely supports a combination of mechanisms for loss of host immune control of the virus. In addition to escape from T cell immunity, the multitude of mutated sequences likely contribute to the general collapse of the early immunity to the virus by altered peptide ligand inhibition of T-cell responses and T-cell exhaustion [47]–[56]. Index switching may be an important mechanism that facilitates this loss of immune control. A small incidence increase of a particular amino acid mutation in the mixed-variable and the highly diverse positions can result in a variant nonatype replacing the prevalent, index sequence of a quasispecies population.

Several methodologies for vaccine development that were designed to overcome HIV-1 sequence diversity have been reported, the majority as strategies based upon the use of centralized sequences to limit the differences between viral strains and the vaccine [15]. The design of these vaccines has generally been based on phylogenetic relationship or the consensus, most common sequence, of the viral population [57]–[61]. However, a limitation of these approaches as a vaccine strategy is that, as shown herein, the centralized or consensus sequence is not an indicator of conservation; the average incidence of the prevalent, index sequence was about 50%, and at the highly diverse positions was as low as 2%. A recent modification of the centralized approach was to prepare sets of ‘mosaic’ proteins, assembled from fragments of natural sequences that are compressed into a small number of native-like proteins [62]–[64]. However, the full-length mosaic vaccines do not overcome the low incidence (average 12%) of the index sequences at the highly diverse positions and have the possibility of unnatural nonamer sequences as epitopes at the junctions of the mosaic fragment. A reported alternative was the use of concatenated mosaic sequences selected from the conserved regions of Gag, Pol and Env [64]; however these also contained highly diverse sequences, including a sequence of Pol that matched a minor variant of incidence 3% in our dataset. Further, these mosaic constructs are also subject to unnatural junctional epitopes.

Given that all nonamer positions are capable of generating variants, barring the two completely conserved Pol positions, our rationale for a new vaccine strategy is one based on the selection of peptide sequences conserved in over 90% of the recorded virus population as a means for establishing selective T-cell based immunity. The goal is the selective development of immune responses that are restricted to highly conserved sequences, with a greatly reduced incidence of mutations that could function as altered peptide ligands. These conserved T-cell epitopes would not be subject to competition in TCR activation by a large or even greater fraction of variant sequences that could function in promiscuous HLA binding but act as antagonists to the TCR receptor. The importance of T-cell responses in the control of HIV-1 infection has been revealed by the findings of the role of T-cells in the reduced viremia of elite controllers [20]–[22]. In addition, rhesus macaque animal model experiments [23]–[27] suggested that CD8+ T cells, even against only a few epitopes, have the potential of limiting virus replication following virus infection and result in long-term immune control under conditions that limit HIV-1 immune escape. The immunity provided by peptide-based vaccines composed solely of the limited set of highly conserved index sequences, many of which are immunogenic as observed by matches to reported T-cell epitopes (data not shown), may be considered a possible strategy for the development of an HIV-1 vaccine.

## Supporting Information

Figure S1 Sample alignments showing the anchor regions of relatively conserved amino acids (boxed in red) that facilitated reliable alignment. The numbers represent the amino acid positions of the protein alignment.
(TIF)

Figure S2 The effect of mutation on overlapping nonamer sequences. **A.** The numbers represent amino acids of overlapping nonamer sequences. **B.** A change in one amino acid, for example from C to P at position 9, will affect nine nonamers spanning 17 amino acids (boxed in red).
(TIF)

Figure S3 Comparison of concatenated index sequence of each HIV-1 clade B protein with the corresponding proteins of HXB2 (green) and C1P (blue) sequences. The numbers before and after the concatenated index sequence represent amino acid positions of the comparison; the comparison is shown in blocks of 60 amino acids. Identical amino acids between the comparisons are represented by “.”; those that differ are shown by the respective amino acids. Amino acid mutations of the aligned viruses that did not follow the concatenation of the index are shown in red. The corresponding amino acids of HXB2 and C1P sequences are also shown at these positions, but without representing identical amino acids by “.”. The green and blue dashes represent amino acid deletions in HXB2 and C1P, respectively.
(PDF)

Table S1 HIV-1 clade B sequences analysed.
(DOC)

Table S2 Accession numbers of reference HIV-1 clade B, consensus HXB2 and C1P protein sequences.
(DOC)

Table S3 Diversity of HIV-1 clade B proteome.
(PDF)

Table S4 Correspondence of concatenated index with HXB2 and C1P sequences. **A.** Amino acid (aa) level comparisons. **B.** Nonamer level comparisons.
(DOC)

## References

[1] Batschelet E, Domingo E, Weissmann C (1976) The proportion of revertant and mutant phage in a growing population, as a function of mutation and growth rate. Gene 1: 27–32. 1052321 10.1016/0378-1119(76)90004-4

[2] Eigen M (1993) Viral quasispecies. Sci Am 269: 42–49. 8337597 10.1038/scientificamerican0793-42

[3] Domingo E, Menendez-Arias L, Holland JJ (1997) RNA virus fitness. Rev Med Virol 7: 87–96. 10398474 10.1002/(sici)1099-1654(199707)7:2<87::aid-rmv188>3.0.co;2-0

[4] Biebricher CK, Eigen M (2006) What is a quasispecies? Curr Top Microbiol Immunol 299: 1–31. 16568894 10.1007/3-540-26397-7_1

[5] Lauring AS, Andino R (2010) Quasispecies theory and the behavior of RNA viruses. PLoS Pathog 6: e1001005. 20661479 10.1371/journal.ppat.1001005 PMC2908548

[6] Wain-Hobson S (1993) The fastest genome evolution ever described: HIV variation in situ. Curr Opin Genet Dev 3: 878–883. 7509668 10.1016/0959-437x(93)90008-d

[7] Holland J, Spindler K, Horodyski F, Grabau E, Nichol S, et al (1982) Rapid evolution of RNA genomes. Science 215: 1577–1585. 7041255 10.1126/science.7041255

[8] Jetzt AE, Yu H, Klarmann GJ, Ron Y, Preston BD, et al (2000) High rate of recombination throughout the human immunodeficiency virus type 1 genome. J Virol 74: 1234–1240. 10627533 10.1128/jvi.74.3.1234-1240.2000 PMC111457

[9] McMichael AJ, Borrow P, Tomaras GD, Goonetilleke N, Haynes BF (2010) The immune response during acute HIV-1 infection: clues for vaccine development. Nat Rev Immunol 10: 11–23. 20010788 10.1038/nri2674 PMC3119211

[10] Boutwell CL, Rolland MM, Herbeck JT, Mullins JI, Allen TM (2010) Viral evolution and escape during acute HIV-1 infection. J Infect Dis 202 Suppl 2 S309–314. 20846038 10.1086/655653 PMC2945609

[11] Troyer RM, McNevin J, Liu Y, Zhang SC, Krizan RW, et al (2009) Variable fitness impact of HIV-1 escape mutations to cytotoxic T lymphocyte (CTL) response. PLoS Pathog 5: e1000365. 19343217 10.1371/journal.ppat.1000365 PMC2659432

[12] Abram ME, Ferris AL, Shao W, Alvord WG, Hughes SH (2010) Nature, position, and frequency of mutations made in a single cycle of HIV-1 replication. J Virol 84: 9864–9878. 20660205 10.1128/JVI.00915-10 PMC2937799

[13] Liu Y, McNevin JP, Holte S, McElrath MJ, Mullins JI (2011) Dynamics of viral evolution and CTL responses in HIV-1 infection. PLoS One 6: e15639. 21283794 10.1371/journal.pone.0015639 PMC3024315

[14] Barouch DH, Korber B (2010) HIV-1 vaccine development after STEP. Annu Rev Med 61: 153–167. 20059334 10.1146/annurev.med.042508.093728 PMC2819364

[15] McBurney SP, Ross TM (2008) Viral sequence diversity: challenges for AIDS vaccine designs. Expert Rev Vaccines 7: 1405–1417. 18980542 10.1586/14760584.7.9.1405 PMC2702717

[16] Walker BD, Burton DR (2008) Toward an AIDS vaccine. Science 320: 760–764. 18467582 10.1126/science.1152622

[17] Taylor BS, Sobieszczyk ME, McCutchan FE, Hammer SM (2008) The challenge of HIV-1 subtype diversity. N Engl J Med 358: 1590–1602. 18403767 10.1056/NEJMra0706737 PMC2614444

[18] Rammensee HG (1995) Chemistry of peptides associated with MHC class I and class II molecules. Curr Opin Immunol 7: 85–96. 7772286 10.1016/0952-7915(95)80033-6

[19] Wucherpfennig KW, Call MJ, Deng L, Mariuzza R (2009) Structural alterations in peptide-MHC recognition by self-reactive T cell receptors. Current opinion in immunology 21: 590–595. 19699075 10.1016/j.coi.2009.07.008 PMC2787854

[20] Blankson JN (2010) Control of HIV-1 replication in elite suppressors. Discovery medicine 9: 261–266. 20350494

[21] Ndhlovu ZM, Proudfoot J, Cesa K, Alvino DM, McMullen A, et al (2012) Elite controllers with low to absent effector CD8+ T cell responses maintain highly functional, broadly directed central memory responses. Journal of virology 86: 6959–6969. 22514340 10.1128/JVI.00531-12 PMC3393560

[22] Autran B, Descours B, Avettand-Fenoel V, Rouzioux C (2011) Elite controllers as a model of functional cure. Current opinion in HIV and AIDS 6: 181–187. 21460722 10.1097/COH.0b013e328345a328

[23] Mudd PA, Martins MA, Ericsen AJ, Tully DC, Power KA, et al (2012) Vaccine-induced CD8+ T cells control AIDS virus replication. Nature 491: 129–133. 23023123 10.1038/nature11443 PMC3883109

[24] Hansen SG, Ford JC, Lewis MS, Ventura AB, Hughes CM, et al (2011) Profound early control of highly pathogenic SIV by an effector memory T-cell vaccine. Nature 473: 523–527. 21562493 10.1038/nature10003 PMC3102768

[25] Hansen SG, Vieville C, Whizin N, Coyne-Johnson L, Siess DC, et al (2009) Effector memory T cell responses are associated with protection of rhesus monkeys from mucosal simian immunodeficiency virus challenge. Nature medicine 15: 293–299. 10.1038/nm.1935 PMC2720091 19219024

[26] Kawada M, Tsukamoto T, Yamamoto H, Iwamoto N, Kurihara K, et al (2008) Gag-specific cytotoxic T-lymphocyte-based control of primary simian immunodeficiency virus replication in a vaccine trial. Journal of virology 82: 10199–10206. 18667518 10.1128/JVI.01103-08 PMC2566295

[27] Tsukamoto T, Takeda A, Yamamoto T, Yamamoto H, Kawada M, et al (2009) Impact of cytotoxic-T-lymphocyte memory induction without virus-specific CD4+ T-Cell help on control of a simian immunodeficiency virus challenge in rhesus macaques. Journal of virology 83: 9339–9346. 19587045 10.1128/JVI.01120-09 PMC2738219

[28] Wheeler DL, Barrett T, Benson DA, Bryant SH, Canese K, et al (2005) Database resources of the National Center for Biotechnology Information. Nucleic Acids Res 33: D39–45. 15608222 10.1093/nar/gki062 PMC540016

[29] Altschul SF, Gish W, Miller W, Myers EW, Lipman DJ (1990) Basic local alignment search tool. J Mol Biol 215: 403–410. 2231712 10.1016/S0022-2836(05)80360-2

[30] Koo QY, Khan AM, Jung KO, Ramdas S, Miotto O, et al (2009) Conservation and variability of West Nile virus proteins. PLoS One 4: e5352. 19401763 10.1371/journal.pone.0005352 PMC2670515

[31] Khan AM, Miotto O, Nascimento EJ, Srinivasan KN, Heiny AT, et al (2008) Conservation and variability of dengue virus proteins: implications for vaccine design. PLoS Negl Trop Dis 2: e272. 18698358 10.1371/journal.pntd.0000272 PMC2491585

[32] Heiny AT, Miotto O, Srinivasan KN, Khan AM, Zhang GL, et al (2007) Evolutionarily conserved protein sequences of influenza a viruses, avian and human, as vaccine targets. PLoS One 2: e1190. 18030326 10.1371/journal.pone.0001190 PMC2065905

[33] Leitner T, Korber B, Daniels M, Calef C, Foley B (2005) HIV-1 Subtype and Circulating Recombinant Form (CRF) Reference Sequences, 2005. The Human Retroviruses and AIDS 2005 Compendium. Los Alamos: Los Alamos National Laboratory.

[34] Yokoyama S, Gojobori T (1987) Molecular evolution and phylogeny of the human AIDS viruses LAV, HTLV-III, and ARV. Journal of molecular evolution 24: 330–336. 3110425 10.1007/BF02134131

[35] Pei J, Kim BH, Grishin NV (2008) PROMALS3D: a tool for multiple protein sequence and structure alignments. Nucleic Acids Res 36: 2295–2300. 18287115 10.1093/nar/gkn072 PMC2367709

[36] Thompson JD, Higgins DG, Gibson TJ (1994) CLUSTAL W: improving the sensitivity of progressive multiple sequence alignment through sequence weighting, position-specific gap penalties and weight matrix choice. Nucleic Acids Res 22: 4673–4680. 7984417 10.1093/nar/22.22.4673 PMC308517

[37] Thompson JD, Thierry JC, Poch O (2003) RASCAL: rapid scanning and correction of multiple sequence alignments. Bioinformatics 19: 1155–1161. 12801878 10.1093/bioinformatics/btg133

[38] Ratner L, Haseltine W, Patarca R, Livak KJ, Starcich B, et al (1985) Complete nucleotide sequence of the AIDS virus, HTLV-III. Nature 313: 277–284. 2578615 10.1038/313277a0

[39] Sahu GK, Sarria JC, Cloyd MW (2010) Recovery of replication-competent residual HIV-1 from plasma of a patient receiving prolonged, suppressive highly active antiretroviral therapy. J Virol 84: 8348–8352. 20519388 10.1128/JVI.00362-10 PMC2916539

[40] Khan AM, Heiny AT, Lee KX, Srinivasan KN, Tan TW, et al (2006) Large-scale analysis of antigenic diversity of T-cell epitopes in dengue virus. BMC Bioinformatics 7 Suppl 5 S4. 10.1186/1471-2105-7-S5-S4 PMC1764481 17254309

[41] Khan AM, Miotto O, Heiny AT, Salmon J, Srinivasan KN, et al (2006) A systematic bioinformatics approach for selection of epitope-based vaccine targets. Cell Immunol 244: 141–147. 17434154 10.1016/j.cellimm.2007.02.005 PMC2041846

[42] Brusic V, Zeleznikow J (1999) Computational binding assays of antigenic peptides. Letters in Peptide Science 6: 313–324.

[43] Shannon CE (1948) A mathematical theory of communication. Bell System Technical Journal 27: 379–423 and 623–656.

[44] Miotto O, Heiny A, Tan TW, August JT, Brusic V (2008) Identification of human-to-human transmissibility factors in PB2 proteins of influenza A by large-scale mutual information analysis. BMC Bioinformatics 9 Suppl 1 S18. 10.1186/1471-2105-9-S1-S18 PMC2259419 18315849

[45] Korber BT, Foley BT, Kuiken CL, Pillai SK, Sodroski JG (1998) Numbering Positions in HIV Relative to HXB2CG. Human Retroviruses and AIDS.

[46] Eigen M, Schuster P (1977) The hypercycle. A principle of natural self-organization. Part A: Emergence of the hypercycle. Die Naturwissenschaften 64: 541–565. 593400 10.1007/BF00450633

[47] Klenerman P, Zinkernagel RM (1998) Original antigenic sin impairs cytotoxic T lymphocyte responses to viruses bearing variant epitopes. Nature 394: 482–485. 9697771 10.1038/28860

[48] Purbhoo MA, Sewell AK, Klenerman P, Goulder PJ, Hilyard KL, et al (1998) Copresentation of natural HIV-1 agonist and antagonist ligands fails to induce the T cell receptor signaling cascade. Proc Natl Acad Sci U S A 95: 4527–4532. 9539771 10.1073/pnas.95.8.4527 PMC22523

[49] Reid SW, McAdam S, Smith KJ, Klenerman P, O'Callaghan CA, et al (1996) Antagonist HIV-1 Gag peptides induce structural changes in HLA B8. J Exp Med 184: 2279–2286. 8976183 10.1084/jem.184.6.2279 PMC2196387

[50] Klenerman P, Meier UC, Phillips RE, McMichael AJ (1995) The effects of natural altered peptide ligands on the whole blood cytotoxic T lymphocyte response to human immunodeficiency virus. European journal of immunology 25: 1927–1931. 7542596 10.1002/eji.1830250720

[51] Sewell AK, Harcourt GC, Goulder PJ, Price DA, Phillips RE (1997) Antagonism of cytotoxic T lymphocyte-mediated lysis by natural HIV-1 altered peptide ligands requires simultaneous presentation of agonist and antagonist peptides. Eur J Immunol 27: 2323–2329. 9341776 10.1002/eji.1830270929

[52] Kirksey TJ, Pogue-Caley RR, Frelinger JA, Collins EJ (1999) The structural basis for the increased immunogenicity of two HIV-reverse transcriptase peptide variant/class I major histocompatibility complexes. J Biol Chem 274: 37259–37264. 10601290 10.1074/jbc.274.52.37259

[53] Moschella F, Ombra MN, Del Pozzo G, Guardiola J (2003) Administration of different antigenic forms of altered peptide ligands derived from HIV-1 RTase influences their effects on T helper cell activation. Hum Immunol 64: 1–8. 12507809 10.1016/s0198-8859(02)00783-8

[54] Streeck H, Brumme ZL, Anastario M, Cohen KW, Jolin JS, et al (2008) Antigen load and viral sequence diversification determine the functional profile of HIV-1-specific CD8+ T cells. PLoS Med 5: e100. 18462013 10.1371/journal.pmed.0050100 PMC2365971

[55] Sauce D, Larsen M, Fastenackels S, Pauchard M, Ait-Mohand H, et al (2011) HIV disease progression despite suppression of viral replication is associated with exhaustion of lymphopoiesis. Blood 117: 5142–5151. 21436070 10.1182/blood-2011-01-331306 PMC3109539

[56] Bouhdoud L, Villain P, Merzouki A, Arella M, Couture C (2000) T-cell receptor-mediated anergy of a human immunodeficiency virus (HIV) gp120-specific CD4(+) cytotoxic T-cell clone, induced by a natural HIV type 1 variant peptide. J Virol 74: 2121–2130. 10666241 10.1128/jvi.74.5.2121-2130.2000 PMC111692

[57] Almeida RR, Rosa DS, Ribeiro SP, Santana VC, Kallas EG, et al (2012) Broad and cross-clade CD4+ T-cell responses elicited by a DNA vaccine encoding highly conserved and promiscuous HIV-1 M-group consensus peptides. PLoS One 7: e45267. 23028895 10.1371/journal.pone.0045267 PMC3445454

[58] Kothe DL, Li Y, Decker JM, Bibollet-Ruche F, Zammit KP, et al (2006) Ancestral and consensus envelope immunogens for HIV-1 subtype C. Virology. 352: 438–449. 10.1016/j.virol.2006.05.011 16780913

[59] Nickle DC, Rolland M, Jensen MA, Pond SL, Deng W, et al (2007) Coping with viral diversity in HIV vaccine design. PLoS Comput Biol 3: e75. 17465674 10.1371/journal.pcbi.0030075 PMC1857809

[60] Thomson SA, Jaramillo AB, Shoobridge M, Dunstan KJ, Everett B, et al (2005) Development of a synthetic consensus sequence scrambled antigen HIV-1 vaccine designed for global use. Vaccine 23: 4647–4657. 15964105 10.1016/j.vaccine.2005.04.045

[61] Malm M, Rollman E, Ustav M, Hinkula J, Krohn K, et al (2005) Cross-clade protection induced by human immunodeficiency virus-1 DNA immunogens expressing consensus sequences of multiple genes and epitopes from subtypes A, B, C, and FGH. Viral immunology 18: 678–688. 16359234 10.1089/vim.2005.18.678

[62] Fischer W, Perkins S, Theiler J, Bhattacharya T, Yusim K, et al (2007) Polyvalent vaccines for optimal coverage of potential T-cell epitopes in global HIV-1 variants. Nat Med 13: 100–106. 17187074 10.1038/nm1461

[63] Barouch DH, O'Brien KL, Simmons NL, King SL, Abbink P, et al (2010) Mosaic HIV-1 vaccines expand the breadth and depth of cellular immune responses in rhesus monkeys. Nat Med 16: 319–323. 20173752 10.1038/nm.2089 PMC2834868

[64] Stephenson KE, SanMiguel A, Simmons NL, Smith K, Lewis MG, et al (2012) Full-length HIV-1 immunogens induce greater magnitude and comparable breadth of T lymphocyte responses to conserved HIV-1 regions compared with conserved-region-only HIV-1 immunogens in rhesus monkeys. Journal of virology 86: 11434–11440. 22896617 10.1128/JVI.01779-12 PMC3486282

