# Supplementary figures and images for: Dissecting the Dynamics of HIV-1 Protein Sequence Diversity
Source: PLoS One. 2013 Apr 4;8(4):e59994. doi: 10.1371/journal.pone.0059994 (PMC3617185; doi:10.1371/journal.pone.0059994)

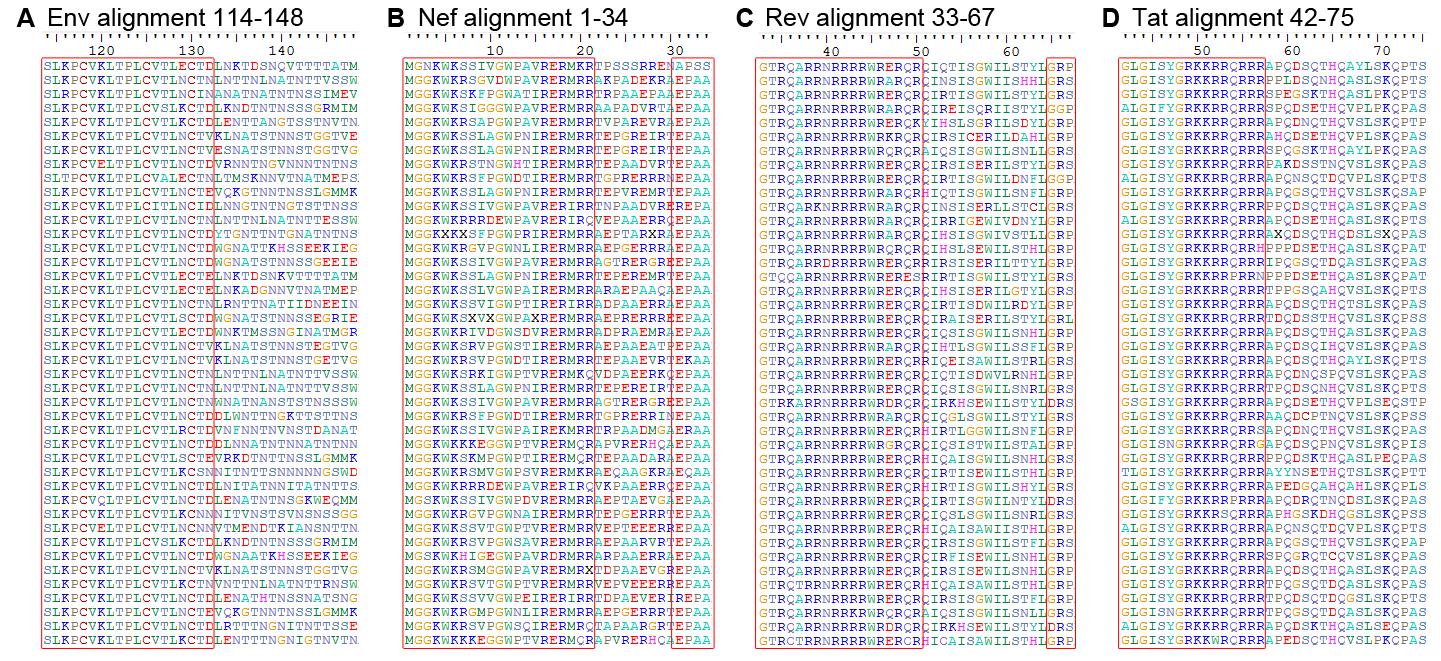

Supplement: Figure S1 — Sample alignments showing the anchor regions of relatively conserved amino acids (boxed in red) that facilitated reliable alignment. The numbers represent the amino acid positions of the protein alignment. (TIF) [file pone.0059994.s001.tif]

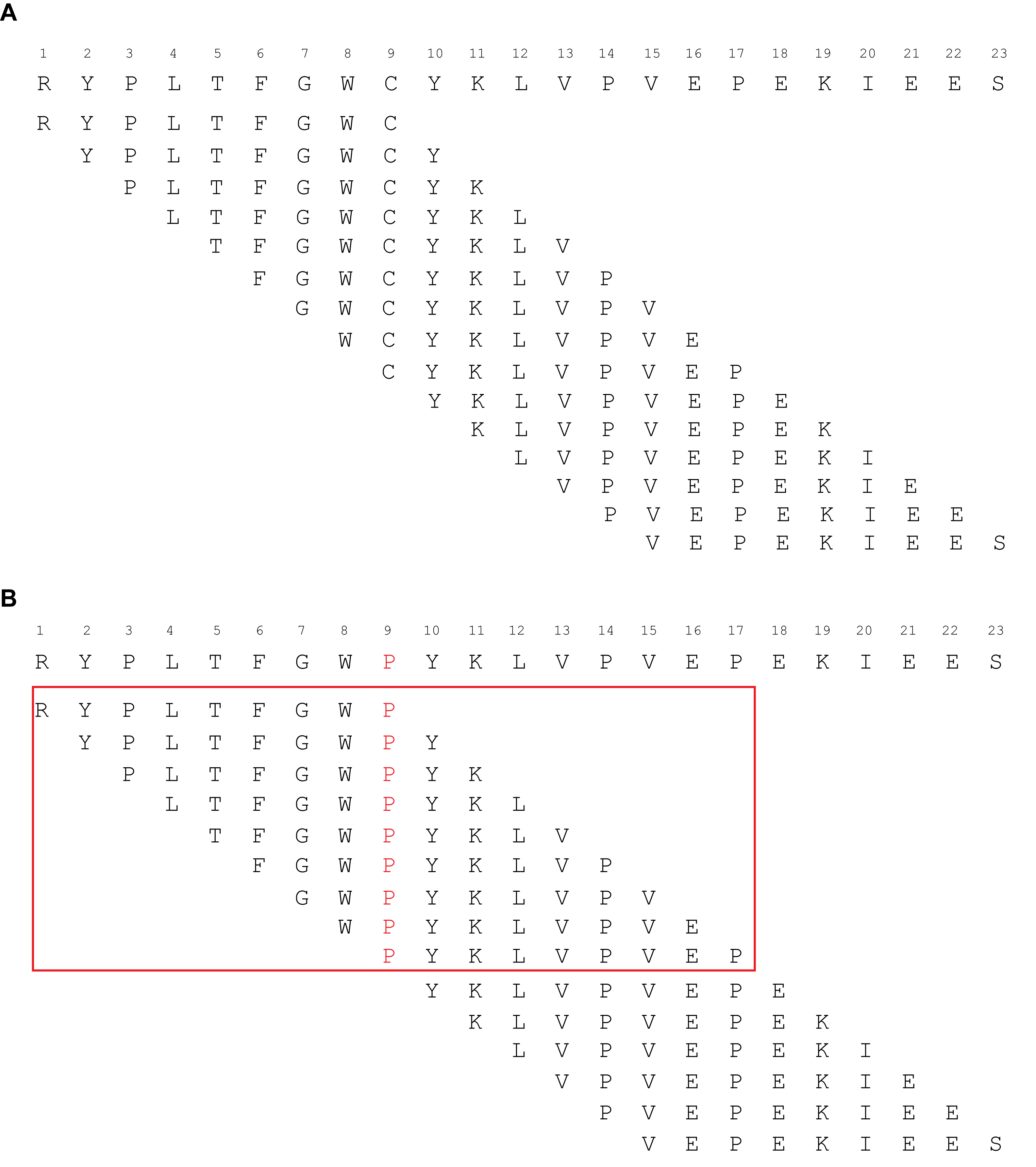

Supplement: Figure S2 — The effect of mutation on overlapping nonamer sequences. A. The numbers represent amino acids of overlapping nonamer sequences. B. A change in one amino acid, for example from C to P at position 9, will affect nine nonamers spanning 17 amino acids (boxed in red). (TIF) [file pone.0059994.s002.tif]
