# Supplementary material for: Dissecting the Dynamics of HIV-1 Protein Sequence Diversity
Source: PLoS One. 2013 Apr 4;8(4):e59994. doi: 10.1371/journal.pone.0059994 (PMC3617185; doi:10.1371/journal.pone.0059994)
Supplement: Figure S3 — Comparison of concatenated index sequence of each HIV-1 clade B protein with the corresponding proteins of HXB2 (green) and C1P (blue) sequences. The numbers before and after the concatenated index sequence represent amino acid positions of the comparison; the comparison is shown in blocks of 60 amino acids. Identical amino acids between the comparisons are represented by “.”; those that differ are shown by the respective amino acids. Amino acid mutations of the aligned viruses that did not follow the concatenation of the index are shown in red. The corresponding amino acids of HXB2 and C1P sequences are also shown at these positions, but without representing identical amino acids by “.”. The green and blue dashes represent amino acid deletions in HXB2 and C1P, respectively. (PDF) [file pone.0059994.s003.pdf]

Gag

```
---------------------------------------------------------------------------
HXB2       .............................K..............................
Index 1    MGARASVLSGGELDRWEKIRLRPGGKKKYRLKHIVWASRELERFAVNPGLLETSEGCRQI   60
                                    K
C1P        ..............K..R...........R..............................
---------------------------------------------------------------------------
HXB2       ...............R..............R.EI.......D................AA
Index 61   LGQLQPSLQTGSEELKSLYNTVATLYCVHQKIEVKDTKEALEKIEEEQNKSKKKAQQAAA   120
                          R              R D        D                QQ
C1P        ...............R.....I........R.D........E................AA
---------------------------------------------------------------------------
HXB2       ---DTGHSNQ---V.........I....................................
Index 121  AAAGTGNSSQIAQISQNYPIVQNLQGQMVHQAISPRTLNAWVKVVEEKAFSPEVIPMFSA   180
            QETAARNAKAS V
              D
C1P        .A-DTGTGNNS-KV...F..........................................
---------------------------------------------------------------------------
HXB2       ........................................V...................
Index 181  LSEGATPQDLNTMLNTVGGHQAAMQMLKETINEEAAEWDRLHPVHAGPIAPGQMREPRGS   240
C1P        ............................................................
---------------------------------------------------------------------------
HXB2       ............................................................
Index 241  DIAGTTSTLQEQIGWMTNNPPIPVGEIYKRWIILGLNKIVRMYSPTSILDIRQGPKEPFR   300
C1P        ..............................................G..........S..
---------------------------------------------------------------------------
HXB2       .................E..........................................
Index 301  DYVDRFYKTLRAEQASQEVKNWMTETLLVQNANPDCKTILKALGPAATLEEMMTACQGVG  360
                            D
C1P        .................E..........................................
---------------------------------------------------------------------------
HXB2       ................T.-S.TI........................T.R..........
Index 361  GPGHKARVLAEAMSQVTNTAAATMMQRGNFRNQRKIVKCFNCGKEGHIARNCRAPRKKGC   420
                           S  S TI                          K
C1P        ..............H.T.-PVNI.....S.....RM.............K..........
---------------------------------------------------------------------------
HXB2       ...........................Y.............PTA-----.......S.V.
Index 421  WKCGKEGHQMKDCTERQANFLGKIWPSHKGRPGNFLQSRPEPTAPEPTAPPEESFRFGEE   480
                                                    QSR   P
C1P        .............N...V...................N...PSA-----..A..LG....
---------------------------------------------------------------------------
HXB2       .TT.PQ....I.......T.............
Index 481  TTTPSQKQEPKDKELYPLASLRSLFGNDPSSQ   512
            AA  S    I
C1P        ATT.PQ...TV.......T.......S.....
---------------------------------------------------------------------------
```

Pol

```
---------------------------------------------------------------------------
HXB2       .L..D...L...............................P.............N...V.
Index 1    FFRENLAFPQGKAREFSSEQTRANSPTRRELQVWGRDNNSLSEAGADRQGTVSFSFPQIT   60
C1P        .......................I...S...R...GG...P....DS.......DL....
---------------------------------------------------------------------------
HXB2       ................................S.........................L.
Index 61   LWQRPLVTIKIGGQLKEALLDTGADDTVLEEMNLPGRWKPKMIGGIGGFIKVRQYDQIPI   120
C1P        ...............I..............DID........I........R.....HV..
---------------------------------------------------------------------------
HXB2       ............................................................
Index 121  EICGHKAIGTVLVGPTPVNIIGRNLLTQIGCTLNFPISPIETVPVKLKPGMDGPKVKQWP   180
C1P        ....Q...T...I............M............................R.....
---------------------------------------------------------------------------
HXB2       ............................................................
Index 181  LTEEKIKALVEICTEMEKEGKISKIGPENPYNTPVFAIKKKDSTKWRKLVDFRELNKRTQ  240
C1P        ...............L.....................V...........T..........
---------------------------------------------------------------------------
```

```
--------------------------------------------------------------------------------
HXB2           ....................................E.......................
Index 241  DFWEVQLGIPHPAGLKKKKSVTVLDVGDAYFSVPLDKDFRKYTAFTIPSINNETPGIRYQ  300
C1P            ............S...............................................
--------------------------------------------------------------------------------
HXB2           ............................................................
Index 301  YNVLPQGWKGSPAIFQSSMTKILEPFRKQNPDIVIYQYMDDLYVGSDLEIGQHRTKIEEL 360
C1P            ............................................................
--------------------------------------------------------------------------------
HXB2           .......L....................................................
Index 361  RQHLLRWGFTTPDKKHQKEPPFLWMGYELHPDKWTVQPIVLPEKDSWTVNDIQKLVGKLN 420
C1P            ....K...Y.........................................L.........
--------------------------------------------------------------------------------
HXB2           ......P....R...............I................................
Index 421  WASQIYPGIKVKQLCKLLRGTKALTEVVPLTEEAELELAENREILKEPVHGVYYDPSKDL 480
                     A                    I
C1P            ......P....................V...A..........................E.
--------------------------------------------------------------------------------
HXB2           ........Q.........................................T.........
Index 481  IAEIQKQGQGQWTYQIYQEPFKNLKTGKYARMRGAHTNDVKQLTEAVQKIATESIVIWGK 540
                       Y
C1P            ........L.........................................S........R
--------------------------------------------------------------------------------
HXB2           ....K.......................................................
Index 541  TPKFKLPIQKETWETWWTEYWQATWIPEWEFVNTPPLVKLWYQLEKEPIVGAETFYVDGA  600
                   R
C1P            ....R.............D..............................A..........
--------------------------------------------------------------------------------
HXB2           ..............N.......T.....................................
Index 601  ANRETKLGKAGYVTDRGRQKVVSLTDTTNQKTELQAIYLALQDSGLEVNIVTDSQYALGI  660
C1P            ........R............I...............H.......S..............
--------------------------------------------------------------------------------
HXB2           ......Q......N..............................................
Index 661  IQAQPDKSESELVSQIIEQLIKKEKVYLAWVPAHKGIGGNEQVDKLVSAGIRKVLFLDGI 720
C1P            .............N....L..N...I..................................
--------------------------------------------------------------------------------
HXB2           ....D.......................................................
Index 721  DKAQEEHEKYHSNWRAMASDFNLPPVVAKEIVASCDKCQLKGEAMHGQVDCSPGIWQLDC 780
C1P            .R...D............N......I.............V....................
--------------------------------------------------------------------------------
HXB2           .........................................................GA.
Index 781  THLEGKVILVAVHVASGYIEAEVIPAETGQETAYFLLKLAGRWPVKTIHTDNGSNFTSTT 840
C1P            ......I............................I...........V.....P..I..A
--------------------------------------------------------------------------------
HXB2           .R..........................................................
Index 841  VKAACWWAGIKQEFGIPYNPQSQGVVESMNKELKKIIGQVRDQAEHLKTAVQMAVFIHNF 900
C1P            ....................................E.......................
--------------------------------------------------------------------------------
HXB2           ..............................................N.............
Index 901  KRKGGIGGYSAGERIVDIIATDIQTKELQKQITKIQNFRVYYRDSRDPLWKGPAKLLWKG 960
C1P            ..................V.............................I...........
--------------------------------------------------------------------------------
HXB2           ...........................................
Index 961  EGAVVIQDNSDIKVVPRRKAKIIRDYGKQMAGDDCVASRQDED 1003
C1P            ...........................................
--------------------------------------------------------------------------------
```

```
Vif
------------------------------------------------------------------------------
HXB2            ..............................V.G..R..........P.............
Index 1         MENRWQVMIVWQVDRMRIRTWKSLVKHHMYISGKAKGWFYRHHYESTHPRISSEVHIPLG   60
                                              V K
C1P             .....................I........I.K.....V......NP...VG.......E
------------------------------------------------------------------------------
HXB2            ..R............................K........E...............D...
Index 61        DAKLVITTYWGLHTGERDWHLGQGVSIEWRKKRYSTQVDPGLADQLIHLYYFDCFSESAI   120
                                               R        D
C1P             .....V....V....................GN.......G...R............T..
------------------------------------------------------------------------------
HXB2            .K.L...I......................A...............T.............
Index 121       RNAILGHIVSPRCEYQAGHNKVGSLQYLALAALITPKKIKPPLPSVRKLTEDRWNKPQKT   160
                       R                      T
C1P             .......R...S..................T......R.R....................
------------------------------------------------------------------------------
HXB2            ............
Index 181       KGHRGSHTMNGH   192
C1P             .D..........
------------------------------------------------------------------------------
Vpr
------------------------------------------------------------------------------
HXB2            ..............H.....................I.......H...............
Index 1         MEQAPEDQGPQREPYNEWTLELLEELKNEAVRHFPRPWLHGLGQYIYETYGDTWAGVEAI   60
                                                            H
C1P             ....................................V.......H.........E....M
------------------------------------------------------------------------------
HXB2            ............QNWV--.T----------------
Index 61        IRILQQLLFIHFRIGCRHSRIGITPQRRARNGASRS   96
                                        R
C1P             L......................IP...T...S...
------------------------------------------------------------------------------

Tat
------------------------------------------------------------------------------
HXB2            .........................................A................H.
Index 1         MEPVDPRLEPWKHPGSQPKTACTNCYCKKCCFHCQVCFITKGLGISYGRKKRRQRRRAPQ   60
C1P             .D.............................L.......Q.................PS.
------------------------------------------------------------------------------
HXB2            N.....A......T............----------------
Index 61        DSQTHQVSLSKQPASQPRGDPTGPKESKKKVERETETDPVHQ   102
                                                      S
C1P             .CK...A......T...G....................P..-
------------------------------------------------------------------------------

Rev
------------------------------------------------------------------------------
HXB2            ............IR......L........N......................HS..ER..
Index 1         MAGRSGDSDEELLKTVRLIKFLYQSNPPPSPEGTRQARRNRRRRWRERQRQIRSISEWIL   60
                                             N                      QT  G
C1P             .........DD.........L........SQ.....................RS..ER..
------------------------------------------------------------------------------
HXB2            GTY...S........................................T........
Index 61        SNYLGRPAEPVPLQLPPLERLTLDCNEDCGTSGTQGVGSPQILVESPTVLESGTKE   116
                 TH   S                                        A
C1P             .TY...P................A.S............N....L...TI....A..
------------------------------------------------------------------------------
```

Vpu

```
--------------------------------------------------------------------------
HXB2          ..PIP.V........I..........I..................LI............-
Index 1       MQSLQILAIVALVVAAIIAIVVWSIVFIEYRKILRQRKIDRLIDRIRERAEDSGNESEGD  60
C1P           ..P.V.YS......V.........................K..............D....
--------------------------------------------------------------------------
HXB2          --.I.................V...
Index 61      QEELSALVEMGVEMGHHAPWDIDDL  85
                 V                 V
C1P           ...L.....R.----.L....I...
--------------------------------------------------------------------------
```

Env

```
--------------------------------------------------------------------------
HXB2          .R.K.K---..................S.T.K............................
Index 1       MKVMETRRNYQHLWRWGTMLLGMLMICKAAENLWVTVYYGVPVWKEATTTLFCASDAKAY  60
C1P           .T.KGI.K.......G.IL...I....S.T.Q.........................R..
--------------------------------------------------------------------------
HXB2          ...V.....................V...........D......................
Index 61      DTEAHNVWATHACVPTDPNPQEVVLENVTENFNMWKNNMVEQMHEDIISLWDQSLKPCVK  120
                 V                     G
C1P           K..V..............S...IP.I.........E.K..D...................
--------------------------------------------------------------------------
HXB2          ......S.K...LK.D.NTNSSSGRMIM--------------.K.E........S.S..G
Index 121     LTPLCVTLNCTDNWNNTGNVSDSSWKGGGKISQTNSSGENRMERGDIKNCSFNITTSIRD  180
                                TSVNTNSSVEATSNT          K E          N
                               NS GGTINS N
                                  TEETL
                                   NVL
                                    D
C1P           ..........I.VNITNTNSTNPTSSWETM------------.K.E..........N...
--------------------------------------------------------------------------
HXB2          .......F.....II..DNDTTS---------.K.TS.......................
Index 181     KVQKEYALFYKLDVVPIDNDNNSSNNTNNTSTYRLINCNTSVITQACPKVSFEPIPIHYC  240
                               GENETNGKYNSTNTN    S
                                 ED AAS   SY
C1P           .I.......NR......NNSNTY---------....S.....L.................
--------------------------------------------------------------------------
HXB2          ...........N.T..........................................V...
Index 241     APAGFAILKCNDKKFNGTGPCTNVSTVQCTHGIRPVVSTQLLLNGSLAEEEVVIRSENFT  300
C1P           ...........ET....S...K..T.......................V..........S
--------------------------------------------------------------------------
HXB2          D..........T...............R.R.......VTI.-K..NM.............
Index 301     NNAKTIIVQLNESVEINCTRPNNNTRKSIHIGPGRAFYATGEIIGDIRQAHCNISRAKWN  360
                                                       D
C1P           D......I...T...............G.............D.............T....
--------------------------------------------------------------------------
HXB2          ...K..AS.......N....I.K..........T.............S.Q......FNST
Index 361     NTLRQIVIKLREQFGNNKTIVFNQSSGGDPEIVMHSFNCGGEFFYCNTTQLFNSTWNSND  420
                 K   T       G                                 P      G
                     K
C1P           D..KH..G..K...RNATK....H.........................R......NVST
--------------------------------------------------------------------------
HXB2          WST.GSNNTEGSDT--...............K..........S.................
Index 421     SRPENNTGGNETTGTEITLPCRIKQIINMWQEVGKAMYAPPIRGQIRCSSNITGLLLTRD  480
                  SENTTGNGEHPP
                   TEVKEGISDS
                     S T Q
C1P           WNISQTEGSYNTEEN-..........V.................I.........IM....
--------------------------------------------------------------------------
```

```
--------------------------------------------------------------------------
HXB2       ...SNNE-----S.I...G............................K......Q.....
Index 481  GGNNNNSNGTQGNETFRPGGGDMRDNWRSELYKYKVVKIEPLGVAPTRAKRRVVQREKRA  540
              QTGTDENNTT I   I                            K      R
              R T    G
                I
C1P        ..KNRTENT---T.T...G..N.M........N..............K......Q.....
--------------------------------------------------------------------------
HXB2       V.-I..L................M....................................
Index 541  VGTIGAMFLGFLGAAGSTMGAASITLTVQARQLLSGIVQQQNNLLRAIEAQQHLLQLTVW  600
           I  L  L                L
C1P        A.-L..L................L....................................
--------------------------------------------------------------------------
HXB2       ........I...............................A.......EQ..NHT.....
Index 601  GIKQLQARVLAVERYLKDQQLLGIWGCSGKLICTTAVPWNASWSNKSLDNIWGNMTWMEW  660
                                                   T       NE  D
                                                           EQ  N
C1P        ................R..................T....A.....N.TT..N.....Q.
--------------------------------------------------------------------------
HXB2       D...N...S..HS.............................N.........L.......
Index 661  EREIDNYTGLIYTLIEESQNQQEKNEQELLELDKWASLWNWFDITNWLWYIKIFIMIVGG  720
C1P        .K......EI..D...Q......................S....................
--------------------------------------------------------------------------
HXB2       .V......A...................H..TP.....................IR..N.
Index 721  LIGLRIVFTVLSIVNRVRQGYSPLSFQTHLPAQRGPDRPEGIEEEGGERDRDRSGPLVDG  780
                                       R  TP
C1P        ......I.A...................H..AR.........................N.
--------------------------------------------------------------------------
HXB2       S..L..D.....................................................
Index 781  FLAIIWVDLRSLCLFSYHRLRDLLLIVTRIVELLGRRGWEALKYWWNLLQYWSQELKNSA  840
C1P        ...L.....................LAA............I...C...............
--------------------------------------------------------------------------
HXB2       .................V...V.G.C...R...R........I..
Index 841  VSLLNATAIAVAEGTDRIIEVVQRAGRAILHIPTRIRQGLERALL  885
                            V   L   C
C1P        ....D...VT..G....IL..LR.IW..F....R...........
--------------------------------------------------------------------------
```

Nef

```
--------------------------------------------------------------------------
HXB2       ......S..I...T......RA--------...DR....A....................
Index 1    MGGKWKRSVVGWPAVRERMRRAEPAAEATREPAAVGVGAVSRDLEKHGAITSSNTAATNA  60
                 S             QTPA EARRT    D
                                 D   DV A
                                      E
C1P        ......R..P....I...T.RAEP.ADGRA....A....V.................N.P
--------------------------------------------------------------------------
HXB2       A.........-.......T...........A.V................H..R.......
Index 61   DCAWLEAQEEEEEVGFPVRPQVPLRPMTYKGALDLSHFLKEKGGLEGLIYSQKRQDILDL  120
C1P        ..........GD....................................VH..Q.......
--------------------------------------------------------------------------
HXB2       .I..................V.........Y.......D.I....K...TS....V.L..
Index 121  WVYHTQGYFPDWQNYTPGPGIRYPLTFGWCFKLVPVEPEKVEEANEGENNSLLHPMSQHG  180
                                                             C      L
C1P        .I....................F..C.........M..............S....L.Q..
--------------------------------------------------------------------------
HXB2       .....R...E.R.........V........F.N.
Index 181  MDDPEKEVLVWKFDSRLAFHHMARELHPEYYKDC  214
C1P        .....................V...K....F.N.
--------------------------------------------------------------------------
```
