# Supplementary material for: Dissecting the Dynamics of HIV-1 Protein Sequence Diversity
Source: PLoS One. 2013 Apr 4;8(4):e59994. doi: 10.1371/journal.pone.0059994 (PMC3617185; doi:10.1371/journal.pone.0059994)
Supplement: Table S1 — HIV-1 clade B sequences analysed. (DOC) [file pone.0059994.s004.doc]

**Table S1 | HIV-1 clade B sequences analysed.**

| **Protein** | **Amino acids ^a^** | **Nonamer positions ^b^** | **Sequences (No.) ^c^** |
| --- | --- | --- | --- |
| Gag | 500 | 492 | 6403 |
| Pol | 1003 | 762 | 30604 |
| Vif | 192 | 184 | 1147 |
| Vpr | 78 | 88 | 1041 |
| Tat | 86 | 93 | 1569 |
| Rev | 116 | 108 | 1533 |
| Vpu | 81 | 65 | 1223 |
| Env | 856 | 876 | 9661 |
| Nef | 206 | 206 | 4871 |
| *Proteome* | *3040* | *2874* | *58052* |

**^a^** Protein size with respect to clade HXB2 reference sequence (see Table S2).

**^b^** Number of nonamer positions analysed, each with more than 100 sequences. Some proteins had more nonamer positions than the expected from the HXB2 protein size because of different lengths of the proteins and alignment gaps. Several of the proteins had fewer nonamer positions than expected from the HXB2 reference sequence. This is particularly true of Pol and is the result of removal of positions with low support (less than 100 sequences).

^c^ Retrieved from NCBI Entrez Protein Database. These included both full-length and a large number of partial sequences, which ranged in size from 17 to 912 amino acids.
