# Supplementary material for: Dissecting the Dynamics of HIV-1 Protein Sequence Diversity
Source: PLoS One. 2013 Apr 4;8(4):e59994. doi: 10.1371/journal.pone.0059994 (PMC3617185; doi:10.1371/journal.pone.0059994)
Supplement: Table S2 — Accession numbers of reference HIV-1 clade B, consensus HXB2 and C1P protein sequences. (DOC) [file pone.0059994.s005.doc]

**Table S2 | Accession numbers of reference HIV-1 clade B, consensus HXB2 and C1P protein sequences.**

| **Protein** | **Reference ^a^** | **HXB2 ^a^** | **C1P ^a^** |
| --- | --- | --- | --- |
| Gag | 29119256 | 120845 | 293573191 |
| Pol | 29119257 | 77416881 | 293573192 |
| Vif | 29119258 | 62291046 | 293573193 |
| Vpr | 29119259 | 62291049 | 293573194 |
| Tat | 29119263 | 6094428 | 293573195 |
| Rev | 29119264 | 132438 | 293573196 |
| Vpu | 29119260 | 139440 | 293573197 |
| Env | 29119261 | 6015102 | 293573198 |
| Nef | 29119262 | 288558856 | 293573199 |

^a^ Accession number (GIs) obtained from Los Alamos HIV sequence database (<http://www.hiv.lanl.gov/content/sequence/HIV/mainpage.html>).
