# Supplementary material for: Dissecting the Dynamics of HIV-1 Protein Sequence Diversity
Source: PLoS One. 2013 Apr 4;8(4):e59994. doi: 10.1371/journal.pone.0059994 (PMC3617185; doi:10.1371/journal.pone.0059994)
Supplement: Table S3 — Diversity of HIV-1 clade B proteome. (PDF) [file pone.0059994.s006.pdf]

Table S3 | Diversity of HIV-1 clade B proteome. ^

Protein	Aligned nonamers: Position[a]	Aligned nonamers: No.[b]	H(x)[c]	Index[d]: Sequence	Index[d]: [%]	Variants[e]: Total [%]	Variants[e]: Major[f] [%]	Variants[e]: Minor[g] [%]	Variants[e]: Unique[h] [%]	Nonatypes[i] [%]	HXB2[j]: Sequence	HXB2[j]: [%]	C1P[k]: Sequence	C1P[k]: [%]
Gag	& 1-9	1156	1.2	MGARASVLS	82	18	10	7	2	3	....	82	....	82
	& 2-10	1160	1.3	GARASVLSG	81	19	9	7	2	3	....	81	....	81
	& 3-11	1164	1.3	ARASVLSGG	81	19	9	8	2	4	....	81	....	81
	& 4-12	1157	2.8	RASVLSGGE	42	58	24	31	3	5	....	42	....	42
	& 5-13	1157	2.8	ASVLSGGEL	42	58	24	31	3	5	....	42	....	42
	& 6-14	1322	2.8	SVLSGGELD	43	57	25	30	3	5	....	43	....	43
	& 7-15	1320	4.1	VLSGGELDR	28	72	15	54	4	7	....	28	........K	15
	& 8-16	1319	3.8	LSGGELDRW	31	69	15	50	3	7	....	31	.......K.	15
	& 9-17	1322	3.6	SGGELDRWE	34	66	15	48	3	6	....	34	......K..	15
	& 10-18	1324	3.9	GGELDRWEK	33	67	13	51	3	7	....	33	.....K..R	2
	& 11-19	1453	3.7	GELDRWEKI	39	61	12	47	3	6	....	39	....K..R.	2
	& 12-20	1452	3.8	ELDRWEKIR	39	61	12	47	3	7	....	39	...K..R..	2
	& 13-21	1471	2.8	LDRWEKIRL	50	50	19	29	3	5	....	50	..K..R...	4
	& 14-22	1477	2.9	DRWEKIRLR	50	50	19	29	3	5	....	50	.K..R....	4
	& 15-23	1485	2.8	RWEKIRLRP	50	50	20	28	3	5	....	50	K..R.....	4
	& 16-24	1551	1.1	WEKIRLRPG	85	15	8	6	1	3	....	85	..R......	8
	& 17-25	1603	1.3	EKIRLRPGG	82	18	8	8	2	3	....	82	.R.......	8
	& 18-26	1616	2.2	KIRLRPGGK	68	32	8	22	2	4	....	68	R........	7
	& 19-27	1630	1.7	IRLRPGGKK	75	25	8	15	2	4	....	75	....	75
	& 20-28	1648	2.7	RLRPGGKKK	60	40	8	30	3	5	....	60	....	60
	& 21-29	1705	2.6	LRPGGKKKY	60	40	8	30	2	4	....	60	....	60
	& 22-30	1723	3.9	RPGGKKKYR	30	70	22	45	4	6	........K	22	....	30
	& 23-31	1746	4.1	PGGKKKYRL	29	71	20	47	4	7	.......K.	20	....	29
	& 24-32	2549	4.1	GGKKKYKLK	27	73	24	46	3	6	....	27	......R..	24
	& 25-33	2602	4.0	GKKKYKLKH	26	74	25	46	3	5	....	26	.....R...	25
	& 26-34	2624	4.6	KKKYKLKHI	21	79	16	60	4	6	....	21	....R....	16
	& 27-35	2670	4.0	KKYKLKHIV	22	78	20	54	3	5	....	22	...R.....	20
	& 28-36	2672	4.0	KYKLKHIVW	23	77	20	54	3	5	....	23	..R......	20
	& 29-37	2717	3.0	YKLKHIVWA	36	64	24	38	2	3	....	36	.R.......	24
	& 30-38	2720	3.0	KLKHIVWAS	36	64	24	38	2	4	....	36	R........	24
	& 31-39	3064	1.8	LKHIVWASR	68	32	19	11	2	3	....	68	....	68
	& 32-40	3146	1.7	KHIVWASRE	68	32	21	9	2	2	....	68	....	68
	& 33-41	3156	1.6	HIVWASREL	68	32	22	9	1	2	....	68	....	68
	& 34-42	3163	1.7	IVWASRELE	68	32	21	9	1	2	....	68	....	68
	# 35-43	3196	0.9	VWASRELER	90	10	2	6	1	2	....	90	....	90
	# 36-44	3269	0.8	WASRELERF	91	9	2	6	<1	2	....	91	....	91
	# 37-45	3316	0.9	ASRELERFA	90	10	2	6	1	2	....	90	....	90
	& 38-46	3311	1.6	SRELERFAV	77	23	10	12	2	3	....	77	....	77
	& 39-47	3325	1.7	RELERFAVN	76	24	10	12	1	3	....	76	....	76
	& 40-48	3506	1.7	ELERFAVNP	77	23	10	12	1	3	....	77	....	77
	& 41-49	3509	1.9	LERFAVNPG	74	26	10	15	1	3	....	74	....	74
	& 42-50	3524	1.8	ERFAVNPGL	74	26	10	15	2	3	....	74	....	74
	& 43-51	3536	1.8	RFAVNPGLL	74	26	10	15	1	3	....	74	....	74
	& 44-52	3641	1.8	FAVNPGLLE	75	25	10	14	1	3	....	75	....	75
	& 45-53	3649	1.9	AVNPGLLET	73	27	10	16	1	2	....	73	....	73
	& 46-54	3650	2.7	VNPGLLETS	60	40	12	27	2	3	....	60	....	60
	& 47-55	3659	2.8	NPGLLETSE	58	42	10	30	2	4	....	58	....	58
	& 48-56	3672	2.7	PGLLETSEG	59	41	10	29	2	3	....	59	....	59
	& 49-57	3677	2.6	GLLETSEGC	60	40	10	28	2	3	....	60	....	60
	& 50-58	3672	3.0	LLETSEGCR	54	46	10	34	2	4	....	54	....	54
	& 51-59	3668	3.2	LETSEGCRQ	52	48	10	36	2	4	....	52	....	52
	& 52-60	3672	3.1	ETSEGCRQI	53	47	10	35	2	4	....	53	....	53
	& 53-61	3666	3.5	TSEGCRQIL	51	49	9	37	3	6	....	51	....	51
	& 54-62	3662	4.7	SEGCRQILG	31	69	11	53	5	8	....	31	....	31
	& 55-63	3664	4.2	EGCRQILGQ	40	60	12	44	4	7	....	40	....	40
	& 56-64	3667	3.4	GCRQILGQL	45	55	19	32	3	5	....	45	....	45
	& 57-65	3660	3.5	CRQILGQLQ	44	56	20	33	3	6	....	44	....	44
	& 58-66	3658	3.7	RQILGQLQP	43	57	19	34	4	6	....	43	....	43
	& 59-67	3648	4.2	QILGQLQPS	30	70	15	51	3	6	....	30	....	30
	& 60-68	3650	4.2	ILGQLQPSL	30	70	15	52	4	7	....	30	....	30
	& 61-69	3640	4.7	LGQLQPSLQ	28	72	13	54	4	8	....	28	....	28
	& 62-70	3648	4.4	GQLQPSLQT	30	70	14	52	3	7	....	30	....	30
	& 63-71	3654	2.9	QLQPSLQTG	51	49	20	27	2	4	....	51	....	51
	& 64-72	3684	3.0	LQPSLQTGS	51	49	20	28	2	4	....	51	....	51
	& 65-73	3687	2.9	QPSLQTGSE	51	49	20	27	2	4	....	51	....	51
	& 66-74	3692	2.8	PSLQTGSEE	53	47	19	26	2	4	....	53	....	53

[&] 67-75	3687	3.0	SLQTGSEEL	54	46	17	26	2	4	....	54	....	54
[&] 68-76	3695	3.1	LQTGSEELK	37	63	34	27	2	4	........R	34	........R	34
[&] 69-77	3699	2.9	QTGSEELKS	39	61	34	24	2	4	.......R.	34	.......R.	34
[&] 70-78	3704	2.3	TGSEELKSL	42	58	40	16	2	3	......R..	40	......R..	40
[&] 71-79	3696	3.3	GSEELRSLY	26	74	21	51	2	4	....	26	....	26
[&] 72-80	3695	3.3	SEELRSLYN	26	74	21	51	2	4	....	26	....	26
[&] 73-81	3709	3.5	EELRSLYNT	25	75	20	53	2	4	....	25	....	25
[&] 74-82	3700	4.1	ELRSLYNTV	20	80	16	61	3	5	....	20	I	5
[+] 75-83	3693	4.2	LRSLYNTVA	20	80	16	61	3	5	....	20	.......I.	4
[+] 76-84	3697	4.5	RSLYNTVAT	17	83	15	65	3	5	....	17	......I..	2
[&] 77-85	3707	3.6	SLYNTVATL	32	68	22	44	2	4	....	32	.....I...	5
[&] 78-86	3696	3.7	LYNTVATLY	32	68	22	44	3	4	....	32	....I....	4
[&] 79-87	3688	3.5	YNTVATLYC	34	66	22	41	3	5	....	34	...I.....	4
[&] 80-88	3696	2.6	NTVATLYCV	57	43	15	27	2	4	....	57	..I......	8
[&] 81-89	3694	2.7	TVATLYCVH	56	44	14	28	2	4	....	56	.I.......	8
[&] 82-90	3653	3.0	VATLYCVHQ	51	49	15	31	2	4	....	51	I........	8
[&] 83-91	3630	3.8	ATLYCVHQK	26	74	24	48	3	5	........R	24	........R	24
[&] 84-92	3623	3.9	TLYCVHQKI	25	75	24	48	3	5	.......R.	24	.......R.	24
[+] 85-93	3615	4.2	LYCVHQRIE	20	80	17	60	3	6	....	20	........D	15
[+] 86-94	3611	4.8	YCVHQKIDV	14	86	14	69	4	7	.....R.EI	10	.....R...	14
[+] 87-95	3598	5.3	CVHQKIDVK	13	87	13	69	5	9	....R.EI.	10	....R....	13
[+] 88-96	3594	5.3	VHQKIDVKD	13	87	13	69	5	9	...R.EI..	10	...R.....	13
[+] 89-97	3592	5.3	HQKIDVKDT	13	87	13	69	5	9	..R.EI...	10	..R......	13
[+] 90-98	3601	5.5	QKIDVKDTK	12	88	11	72	5	10	.R.EI....	10	.R.......	11
[+] 91-99	3606	5.2	KIDVKDTKE	13	87	11	72	4	8	R.EI.....	10	R........	11
[&] 92-100	3625	3.9	IDVKDTKEA	25	75	24	49	3	5	.EI......	13	....	25
[&] 93-101	3602	3.7	EVKDTKEAL	25	75	25	47	2	4	.I.......	13	D........	25
[&] 94-102	3604	3.6	VKDTKEALE	27	73	25	45	2	4	I.......D	8	....	27
[&] 95-103	3600	3.1	KDTKEALEK	35	65	32	31	2	4	.......D.	32	....	35
[&] 96-104	3604	2.7	DTKEALDKI	42	58	34	22	2	4	....	42	......E..	34
[&] 97-105	3598	2.8	TKEALDKIE	42	58	33	23	2	4	....	42	.....E...	33
[&] 98-106	3594	2.8	KEALDKIEE	42	58	33	23	2	4	....	42	....E....	33
[&] 99-107	3585	2.6	EALDKIEEE	43	57	35	20	2	4	....	43	...E.....	35
[&] 100-108	3633	2.6	ALDKIEEEQ	43	57	35	20	2	4	....	43	..E......	35
[&] 101-109	3614	3.1	LDKIEEEQN	37	63	33	27	3	5	....	37	.E.......	33
[&] 102-110	3601	3.3	DKIEEEQNK	36	64	33	28	3	5	....	36	E........	33
[&] 103-111	3589	2.8	KIEEEQNKS	65	35	8	24	3	5	....	65	....	65
[&] 104-112	3590	2.6	IEEEQNKSK	67	33	8	23	3	5	....	67	....	67
[&] 105-113	3565	2.5	EEEQNKSKK	71	29	4	22	3	5	....	71	....	71
[&] 106-114	3616	2.8	EEQNKSKKK	67	33	4	25	4	6	....	67	....	67
[&] 107-115	3552	3.2	EQNKSKKKA	64	36	3	28	4	7	....	64	....	64
[&] 108-116	3546	3.2	QNKSKKKAQ	64	36	4	29	4	7	....	64	....	64
[&] 109-117	3538	3.4	NKSKKKAQQ	61	39	3	31	4	8	....	61	....	61
[&] 110-118	3536	3.5	KSKKKAQQA	60	40	5	31	5	8	....	60	....	60
[&] 111-119	3519	4.0	SKKKAQQAA	50	50	8	37	5	9	....	50	....	50
[&] 112-120	3425	3.9	KKKAQQAAA	51	49	8	36	5	9	....	51	....	51
[&] 113-121	565	4.9	KKAQQAAAA	32	68	10	44	14	24	........-	0	....	32
[&] 114-122	420	5.0	KAQQAAAAA	25	75	14	46	14	25	.......--	0	....	25
[&] 115-123	230	4.8	AQQAQQAAA	25	75	13	44	18	29	....AA---	0	....AA..-	0
[&] 116-124	228	4.8	QQAQQAAAG	23	77	13	47	18	29	...AA---D	0	...AA..-D	0
[&] 117-125	222	4.9	QAQQAAAGT	23	77	12	42	22	33	..AA---D.	0	..AA..-D.	0
[&] 118-126	222	4.9	AQQAAAGTG	23	77	13	44	21	33	.AA---D..	0	.AA..-D..	0
[&] 119-127	214	4.9	QQAAAGTGN	23	77	7	51	18	31	AA---D..H	0	AA..-D..T	0
[&] 120-128	215	5.0	QAAAGTGNS	23	77	8	50	19	32	A---D..H.	0	A..-D..TG	0
[&] 121-129	213	4.8	AAAGTGNSS	24	76	8	49	18	31	---D..H.N	0	..-D..TGN	0
122-130 *	7	2.6	QAGAGNSSQ	14	86	14	0	71	86	--DT.H.N.	0	A-DT.TGNN	0
123-131 *	4	1.6	ETAARSAKI	25	75	25	0	50	75	-DTGH.NQ-	0	-DTGTGNNS	0
[+] 124-132	215	5.3	DTGNNSQAA	17	83	8	48	27	38	...HSN.--	0	...TGNNS-	0
[+] 125-133	216	5.2	TGNNSQAAQ	17	83	8	50	25	36	..HSN.---	0	..TGNNS-K	0
[+] 126-134	211	5.4	GNNSQAAQI	10	90	9	55	26	37	.HSN.---V	0	.TGNNS-KV	0
[+] 127-135	213	5.4	NNSQAAQIS	10	90	8	55	26	38	HSN.---V.	0	TGNNS-KV.	0
[+] 128-136	215	5.5	NSQAAQISQ	11	89	7	54	27	39	SN.---V..	0	GNNS-KV..	0
[+] 129-137	215	5.3	SQAAQISQN	13	87	9	54	24	35	N.---V...	0	NNS-KV...	0
[+] 130-138	217	5.0	QAAQISQNY	13	87	10	58	19	29	.---V....	0	NS-KV...F	0
[+] 131-139	196	4.7	AAQISQNYP	15	85	11	59	15	26	---V.....	0	S-KV...F.	0
[&] 132-140	197	4.0	SQVSQNYPI	26	74	11	54	9	18	--.......	0	-K....F..	0
[&] 133-141	2950	2.3	QVSQNYPIV	69	31	8	21	2	3	-........	0	K....F...	<1
[&] 134-142	2961	1.7	VSQNYPIVQ	77	23	8	13	1	3	....	77	....F....	1
[&] 135-143	3026	1.0	SQNYPIVQN	89	11	3	8	1	2	....	89	...F.....	2
[&] 136-144	3008	2.4	QNYPIVQNL	54	46	19	25	2	3	I	19	..F......	<1
[&] 137-145	3010	2.2	NYPIVQNLQ	57	43	19	22	2	3	.......I.	19	.F.......	1

& 138-146	2951	2.2	YPIVQNLQG	56	44	20	22	2	3	......I..	20	F........	1
& 139-147	3218	2.1	PIVQNLQGQ	58	42	20	21	2	3	.....I...	20	....	58
& 140-148	3198	2.1	IVQNLQGQM	58	42	20	21	2	3	....I....	20	....	58
& 141-149	3190	2.1	VQNLQGQMV	58	42	19	21	2	3	...I.....	19	....	58
& 142-150	3344	2.1	QNLQGQMVH	58	42	19	21	2	3	..I......	19	....	58
& 143-151	3341	2.0	NLQGQMVHQ	59	41	20	19	2	3	.I.......	20	....	59
& 144-152	3328	2.9	LQGQMVHQA	43	57	16	38	2	3	I........	16	....	43
& 145-153	3275	2.3	QGQMVHQAI	56	44	15	28	2	3	....	56	....	56
& 146-154	3227	2.4	GQMVHQAIS	56	44	14	28	2	3	....	56	....	56
& 147-155	3165	2.4	QMVHQAISP	56	44	14	28	2	3	....	56	....	56
& 148-156	3132	2.5	MVHQAISPR	55	45	14	29	2	3	....	55	....	55
& 149-157	3058	2.4	VHQAISPRT	56	44	14	28	2	3	....	56	....	56
& 150-158	2757	2.3	HQAISPRTL	57	43	15	26	1	3	....	57	....	57
& 151-159	2574	2.3	QAISPRTLN	56	44	16	27	1	3	....	56	....	56
& 152-160	2039	2.3	AISPRTLNA	55	45	16	27	2	3	....	55	....	55
& 153-161	2049	1.4	ISPRTLNAW	71	29	23	5	1	2	....	71	....	71
# 154-162	2049	0.5	SPRTLNAWV	95	5	1	3	<1	2	....	95	....	95
# 155-163	2045	0.4	PRTLNAWVK	97	3	<1	2	<1	2	....	97	....	97
# 156-164	1976	0.4	RTLNAWVKV	97	3	<1	2	<1	2	....	97	....	97
& 157-165	1976	1.2	TLNAWVKVV	71	29	25	3	1	2	....	71	....	71
& 158-166	2001	1.2	LNAWVKVVE	70	30	27	2	<1	2	....	70	....	70
& 159-167	1985	1.3	NAWVKVVEE	68	32	27	4	1	2	....	68	....	68
& 160-168	1881	1.3	AWVKVVEEK	68	32	28	3	1	2	....	68	....	68
& 161-169	1863	1.4	WVKVVEEKA	67	33	28	4	1	2	....	67	....	67
& 162-170	1854	1.4	VKVVEEKAF	67	33	28	4	1	2	....	67	....	67
& 163-171	1879	1.4	KVVEEKAFS	66	34	29	3	2	3	....	66	....	66
& 164-172	1880	1.4	VVEEKAFSP	66	34	29	3	2	3	....	66	....	66
& 165-173	1880	1.4	VEEKAFSPE	66	34	29	3	2	3	....	66	....	66
# 166-174	1872	0.6	EEKAFSPEV	94	6	<1	3	2	3	....	94	....	94
# 167-175	1863	0.6	EKAFSPEVI	94	6	<1	3	2	3	....	94	....	94
# 168-176	1861	0.6	KAFSPEVIP	95	5	<1	3	2	2	....	95	....	95
# 169-177	1854	0.5	AFSPEVIPM	95	5	<1	2	2	2	....	95	....	95
# 170-178	1854	0.4	FSPEVIPMF	96	4	<1	2	1	2	....	96	....	96
& 171-179	1862	1.3	SPEVIPMFS	76	24	14	8	1	2	....	76	....	76
& 172-180	1877	1.3	PEVIPMFSA	76	24	15	7	2	2	....	76	....	76
& 173-181	1869	1.3	EVIPMFSAL	76	24	15	7	1	2	....	76	....	76
& 174-182	1922	1.5	VIPMFSALS	72	28	15	11	2	2	....	72	....	72
& 175-183	1925	1.6	IPMFSALSE	72	28	15	12	2	3	....	72	....	72
& 176-184	1926	1.5	PMFSALSEG	72	28	15	12	1	2	....	72	....	72
& 177-185	1925	1.5	MFSALSEGA	72	28	15	12	1	2	....	72	....	72
& 178-186	1924	1.5	FSALSEGAT	72	28	15	12	1	2	....	72	....	72
& 179-187	1924	1.5	SALSEGATP	72	28	15	12	1	2	....	72	....	72
# 180-188	1922	0.8	ALSEGATPQ	91	9	3	5	2	2	....	91	....	91
# 181-189	1923	0.8	LSEGATPQD	91	9	3	5	1	2	....	91	....	91
# 182-190	1920	0.8	SEGATPQDL	91	9	3	5	1	2	....	91	....	91
# 183-191	1920	0.6	EGATPQDLN	94	6	1	4	1	2	....	94	....	94
# 184-192	1919	0.8	GATPQDLNT	92	8	<1	5	2	3	....	92	....	92
# 185-193	1920	0.8	ATPQDLNTM	92	8	<1	5	2	3	....	92	....	92
# 186-194	1922	0.8	TPQDLNTML	92	8	<1	5	2	3	....	92	....	92
# 187-195	1920	0.8	PQDLNTMLN	93	8	<1	5	2	3	....	93	....	93
# 188-196	1921	0.9	QDLNTMLNT	92	8	<1	5	2	3	....	92	....	92
# 189-197	1922	0.7	DLNTMLNTV	93	7	<1	4	2	3	....	93	....	93
# 190-198	1921	0.7	LNTMLNTVG	93	7	<1	4	2	3	....	93	....	93
# 191-199	1918	0.7	NTMLNTVGG	93	7	1	5	1	3	....	93	....	93
# 192-200	1922	0.7	TMLNTVGGH	93	7	1	5	1	2	....	93	....	93
# 193-201	1913	0.6	MLNTVGGHQ	94	6	1	4	1	2	....	94	....	94
# 194-202	1894	0.5	LNTVGGHQA	94	6	1	3	1	2	....	94	....	94
# 195-203	1870	0.5	NTVGGHQAA	94	6	1	3	1	2	....	94	....	94
# 196-204	1834	0.5	TVGGHQAAM	94	6	1	3	1	2	....	94	....	94
# 197-205	1824	0.3	VGGHQAAMQ	97	3	<1	1	1	2	....	97	....	97
# 198-206	1807	0.4	GGHQAAMQM	96	4	2	1	1	2	....	96	....	96
# 199-207	1798	0.4	GHQAAMQML	96	4	2	1	<1	1	....	96	....	96
# 200-208	1795	0.4	HQAAMQMLK	96	4	2	1	1	2	....	96	....	96
& 201-209	1787	1.0	QAAMQMLKE	82	18	15	3	1	2	....	82	....	82
& 202-210	1780	1.0	AAMQMLKET	81	19	14	3	1	2	....	81	....	81
& 203-211	1770	1.0	AMQMLKETI	81	19	14	3	1	2	....	81	....	81
& 204-212	1765	1.1	MQMLKETIN	81	19	14	4	1	2	....	81	....	81
& 205-213	1655	1.3	QMLKETINE	78	22	15	6	2	3	....	78	....	78
& 206-214	1654	1.3	MLKETINEE	78	22	15	6	2	3	....	78	....	78
& 207-215	1652	1.2	LKETINEEA	79	21	15	5	2	2	....	79	....	79
& 208-216	1653	1.2	KETINEEAA	79	21	15	5	2	3	....	79	....	79

& 209-217	1644	1.4	ETINEEAAE	77	23	14	7	2	3	....	77	....	77
# 210-218	1639	0.8	TINEEAAEW	92	8	2	5	1	3	....	92	....	92
# 211-219	1637	0.7	INEEAAEWD	93	7	2	4	1	2	....	93	....	93
# 212-220	1638	0.7	NEEAAEWDR	93	7	2	4	2	2	....	93	....	93
& 213-221	1628	1.8	EEAAEWDRL	69	31	17	12	2	3	V	17	....	69
& 214-222	1610	1.6	EAAEWDRLH	71	29	18	9	2	3	.......V.	18	....	71
& 215-223	1611	1.5	AAEWDRLHP	72	28	18	9	2	3	......V..	18	....	72
& 216-224	1608	1.8	AEWDRLHPV	69	31	17	11	2	4	.....V...	17	....	69
& 217-225	1623	2.0	EWDRLHPVH	66	34	17	15	2	4	....V....	17	....	66
& 218-226	1623	1.9	WDRLHPVHA	67	33	17	14	2	4	...V.....	17	....	67
& 219-227	1619	2.0	DRLHPVHAG	67	33	17	14	2	4	..V......	17	....	67
& 220-228	1555	1.9	RLHPVHAGP	68	32	16	13	2	4	.V.......	16	....	68
& 221-229	1547	3.0	LHPVHAGPI	50	50	12	35	3	6	V........	12	....	50
& 222-230	1554	2.5	HPVHAGPIA	58	42	18	22	3	5	....	58	....	58
& 223-231	1552	2.5	PVHAGPIAP	58	42	18	21	3	5	....	58	....	58
& 224-232	1552	2.5	VHAGPIAPG	58	42	18	21	3	5	....	58	....	58
& 225-233	1553	2.3	HAGPIAPGQ	59	41	18	21	2	4	....	59	....	59
& 226-234	1558	2.5	AGPIAPGQM	58	42	17	22	3	4	....	58	....	58
& 227-235	1556	2.5	GPIAPGQMR	58	42	17	22	3	4	....	58	....	58
& 228-236	1555	2.7	PIAPGQMRE	56	44	16	24	3	5	....	56	....	56
& 229-237	1555	2.6	IAPGQMREP	57	43	16	24	3	5	....	57	....	57
& 230-238	1566	1.5	APGQMREPR	78	22	7	13	2	3	....	78	....	78
& 231-239	1566	1.0	PGQMREPRG	86	14	5	7	2	3	....	86	....	86
& 232-240	1541	1.1	GQMREPRGS	85	15	5	8	2	3	....	85	....	85
& 233-241	1541	1.1	QMREPRGSD	85	15	5	8	2	3	....	85	....	85
& 234-242	1529	1.2	MREPRGSDI	85	15	5	8	2	3	....	85	....	85
# 235-243	1532	0.6	REPRGSDIA	93	7	3	3	1	2	....	93	....	93
# 236-244	1531	0.6	EPRGSDIAG	93	7	3	3	<1	2	....	93	....	93
# 237-245	1526	0.5	PRGSDIAGT	95	5	<1	3	1	2	....	95	....	95
# 238-246	1525	0.5	RGSDIAGTT	95	5	<1	3	1	2	....	95	....	95
# 239-247	1517	0.5	GSDIAGTTS	95	5	<1	3	1	2	....	95	....	95
& 240-248	1515	1.0	SDIAGTTST	84	16	9	5	1	2	....	84	....	84
& 241-249	1516	1.0	DIAGTTSTL	84	16	9	5	1	2	....	84	....	84
& 242-250	1517	1.1	IAGTTSTLQ	84	16	9	5	2	3	....	84	....	84
& 243-251	1514	1.1	AGTTSTLQE	83	17	9	6	2	3	....	83	....	83
& 244-252	1511	1.1	GTTSTLQEQ	83	17	9	5	2	3	....	83	....	83
& 245-253	1510	1.3	TTSTLQEQI	82	18	9	7	2	3	....	82	....	82
& 246-254	1510	2.2	TSTLQEQIG	66	34	11	21	2	4	....	66	....	66
& 247-255	1508	2.2	STLQEQIGW	66	34	11	21	2	4	....	66	....	66
& 248-256	1507	2.2	TLQEQIGWM	66	34	10	22	2	4	....	66	....	66
& 249-257	1507	1.8	LQEQIGWMT	71	29	14	13	2	4	....	71	....	71
& 250-258	1494	3.1	QEQIGWMTN	46	54	13	37	3	5	....	46	....	46
& 251-259	1494	3.0	EQIGWMTNN	46	54	13	38	3	5	....	46	....	46
& 252-260	1597	3.0	QIGWMTNNP	48	52	13	37	3	5	....	48	....	48
& 253-261	1598	3.1	IGWMTNNPP	48	52	13	37	3	6	....	48	....	48
& 254-262	1599	3.2	GWMTNNPPI	48	52	13	36	3	6	....	48	....	48
& 255-263	1602	2.5	WMTNNPPIP	53	47	16	28	2	4	....	53	....	53
& 256-264	1603	2.5	MTNNPPIPV	53	47	16	28	2	4	....	53	....	53
& 257-265	1605	2.5	TNNPPIPVG	53	47	16	28	2	4	....	53	....	53
& 258-266	1600	2.8	NNPPIPVGE	51	49	15	32	3	5	....	51	....	51
& 259-267	1607	1.4	NPPIPVGEI	81	19	5	12	2	3	....	81	....	81
& 260-268	1609	1.4	PPIPVGEIY	81	19	5	12	2	3	....	81	....	81
& 261-269	1606	1.4	PIPVGEIYK	81	19	5	12	2	3	....	81	....	81
& 262-270	1606	1.7	IPVGEIYKR	74	26	9	15	2	3	....	74	....	74
& 263-271	1602	1.4	PVGEIYKRW	79	21	10	10	2	3	....	79	....	79
& 264-272	1595	1.3	VGEIYKRWI	79	21	10	10	2	3	....	79	....	79
& 265-273	1480	1.4	GEIYKRWII	79	21	10	9	2	3	....	79	....	79
& 266-274	1478	2.0	EIYKRWIIL	66	34	10	22	2	4	....	66	....	66
& 267-275	1480	1.7	IYKRWIILG	70	30	10	18	2	3	....	70	....	70
& 268-276	1480	1.7	YKRWIILGL	70	30	10	18	1	3	....	70	....	70
& 269-277	1482	1.7	KRWIILGLN	70	30	10	18	2	3	....	70	....	70
& 270-278	1486	1.7	RWIILGLNK	70	30	10	18	2	3	....	70	....	70
& 271-279	1484	1.4	WIILGLNKI	72	28	19	8	1	2	....	72	....	72
& 272-280	1490	1.4	IILGLNKIV	72	28	19	7	1	2	....	72	....	72
& 273-281	1498	1.4	ILGLNKIVR	72	28	19	7	1	2	....	72	....	72
& 274-282	1499	1.3	LGLNKIVRM	73	27	19	7	2	2	....	73	....	73
# 275-283	1495	0.5	GLNKIVRMY	95	5	1	3	<1	2	....	95	....	95
# 276-284	1453	0.5	LNKIVRMYS	95	5	1	3	1	2	....	95	....	95
# 277-285	1458	0.5	NKIVRMYSP	95	5	1	2	1	2	....	95	....	95
& 278-286	1469	1.7	KIVRMYSPT	59	41	30	10	2	3	....	59	....	59
& 279-287	1454	1.8	IVRMYSPTS	57	43	29	11	2	3	....	57	........G	<1

& 280-288	1453	1.8	VRMYSPTSI	57	43	29	12	2	2	....	57	.......G.	<1
& 281-289	1449	1.8	RMYSPTSIL	57	43	29	12	2	3	....	57	......G..	<1
& 282-290	1478	1.9	MYSPTSILD	56	44	30	12	2	3	....	56	.....G...	<1
& 283-291	1481	1.9	YSPTSILDI	56	44	30	12	2	3	....	56	....G....	<1
& 284-292	1479	2.6	SPTSILDIR	38	62	26	33	2	4	....	38	...G.....	<1
& 285-293	1478	2.6	PTSILDIRQ	38	62	27	33	2	3	....	38	..G......	<1
& 286-294	1478	2.5	TSILDIRQG	38	62	27	33	2	3	....	38	.G.......	<1
& 287-295	1481	1.3	SILDIRQGP	72	28	22	4	1	2	....	72	G........	<1
& 288-296	1473	1.2	ILDIRQGPK	74	26	22	3	2	3	....	74	....	74
& 289-297	1468	1.2	LDIRQGPKE	74	26	22	2	2	2	....	74	....	74
& 290-298	1468	1.3	DIRQGPKEP	72	28	22	4	2	3	....	72	........S	1
& 291-299	1467	1.2	IRQGPKEPF	73	27	22	4	1	2	....	73	.......S.	1
& 292-300	1466	1.3	RQGPKEPFR	73	27	22	4	1	2	....	73	......S..	1
# 293-301	1462	0.5	QGPKEPFRD	95	5	2	2	1	2	....	95	.....S...	2
# 294-302	1462	0.5	GPKEPFRDY	95	5	2	2	1	2	....	95	....S....	2
# 295-303	1461	0.5	PKEPFRDYV	95	5	2	2	1	2	....	95	...S.....	2
# 296-304	1434	0.5	KEPFRDYVD	95	5	2	2	1	2	....	95	..S......	2
# 297-305	1433	0.5	EPFRDYVDR	95	5	2	2	1	2	....	95	.S.......	2
# 298-306	1429	0.5	PFRDYVDRF	95	5	2	2	1	2	....	95	S........	2
& 299-307	1428	0.8	FRDYVDRFY	88	12	9	2	1	2	....	88	....	88
& 300-308	1374	0.9	RDYVDRFYK	87	13	9	3	1	2	....	87	....	87
& 301-309	1372	1.1	DYVDRFYKT	85	15	7	6	2	3	....	85	....	85
& 302-310	1372	1.0	YVDRFYKTL	85	15	7	6	2	3	....	85	....	85
& 303-311	1313	1.0	VDRFYKTLR	88	12	5	6	1	2	....	88	....	88
& 304-312	1309	0.9	DRFYKTLRA	88	12	5	6	1	2	....	88	....	88
& 305-313	1303	0.9	RFYKTLRAE	88	12	5	6	1	2	....	88	....	88
& 306-314	1289	1.0	FYKTLRAEQ	88	12	5	6	1	2	....	88	....	88
& 307-315	1283	1.2	YKTLRAEQA	84	16	4	10	2	3	....	84	....	84
& 308-316	1230	1.5	KTLRAEQAS	77	23	11	10	2	3	....	77	....	77
& 309-317	1224	1.4	TLRAEQASQ	78	22	12	9	2	3	....	78	....	78
& 310-318	1205	2.1	LRAEQASQE	41	59	38	18	2	3	....	41	....	41
& 311-319	1204	2.1	RAEQASQEV	42	58	38	18	2	3	....	42	....	42
& 312-320	1203	2.0	AEQASQEVK	41	59	39	18	2	3	....	41	....	41
& 313-321	1119	2.4	EQASQDVKN	41	59	35	22	3	5	.....E...	35	.....E...	35
& 314-322	1118	2.4	QASQDVKNW	41	59	35	22	2	4	....E....	35	....E....	35
& 315-323	1117	2.4	ASQDVKNWM	40	60	35	22	2	4	...E.....	35	...E.....	35
& 316-324	1116	2.2	SQDVKNWMT	41	59	36	21	2	4	..E......	36	..E......	36
& 317-325	1114	2.0	QEVKNWMTE	47	53	40	10	2	4	....	47	....	47
& 318-326	1114	2.0	EVKNWMTET	47	53	40	10	3	4	....	47	....	47
& 319-327	1129	1.0	VKNWMTETL	88	12	4	7	2	3	....	88	....	88
& 320-328	1129	1.1	KNWMTETLL	87	13	4	7	2	3	....	87	....	87
& 321-329	1126	1.2	NWMTETLLV	86	14	4	8	2	4	....	86	....	86
& 322-330	1101	0.9	WMTETLLVQ	88	12	5	5	2	3	....	88	....	88
& 323-331	1101	0.9	MTETLLVQN	88	12	5	5	2	3	....	88	....	88
& 324-332	1101	1.2	TETLLVQNA	82	18	6	10	2	3	....	82	....	82
& 325-333	1101	1.3	ETLLVQNAN	82	18	6	10	2	3	....	82	....	82
& 326-334	1101	0.9	TLLVQNANP	87	13	6	4	2	3	....	87	....	87
& 327-335	1102	0.9	LLVQNANPD	88	12	6	4	2	3	....	88	....	88
& 328-336	1103	0.9	LVQNANPDC	88	12	6	4	2	3	....	88	....	88
& 329-337	1104	1.2	VQNANPDCK	82	18	6	9	2	3	....	82	....	82
& 330-338	1105	1.2	QNANPDCKT	82	18	6	10	2	3	....	82	....	82
& 331-339	1105	1.2	NANPDCKTI	82	18	6	9	2	3	....	82	....	82
& 332-340	1103	1.2	ANPDCKTIL	82	18	6	9	2	3	....	82	....	82
& 333-341	1103	1.0	NPDCKTILK	87	13	6	5	2	3	....	87	....	87
& 334-342	1103	0.9	PDCKTILKA	87	13	6	5	2	3	....	87	....	87
& 335-343	1106	0.9	DCKTILKAL	87	13	6	5	1	3	....	87	....	87
& 336-344	1107	1.0	CKTILKALG	87	13	6	5	2	3	....	87	....	87
& 337-345	1107	1.0	KTILKALGP	87	13	6	6	2	3	....	87	....	87
& 338-346	1109	1.4	TILKALGPA	78	22	14	6	2	4	....	78	....	78
& 339-347	1040	1.2	ILKALGPAA	78	22	15	5	2	3	....	78	....	78
& 340-348	1043	1.4	LKALGPAAT	78	22	14	6	2	4	....	78	....	78
& 341-349	1036	1.4	KALGPAATL	77	23	15	6	2	4	....	77	....	77
& 342-350	1033	1.3	ALGPAATLE	77	23	15	6	2	4	....	77	....	77
& 343-351	1029	1.4	LGPAATLEE	76	24	15	6	3	4	....	76	....	76
& 344-352	943	1.4	GPAATLEEM	76	24	15	6	3	5	....	76	....	76
& 345-353	909	1.5	PAATLEEMM	75	25	15	6	3	5	....	75	....	75
& 346-354	907	1.7	AATLEEMMT	72	28	15	9	3	5	....	72	....	72
& 347-355	881	1.1	ATLEEMMTA	88	12	2	7	2	4	....	88	....	88
& 348-356	765	0.9	TLEEMMTAC	89	11	3	6	2	3	....	89	....	89
# 349-357	722	0.7	LEEMMTACQ	91	9	3	4	2	3	....	91	....	91
# 350-358	706	0.6	EEMMTACQG	93	7	3	2	2	3	....	93	....	93

# 351-359	698	0.6	EMMTACQGV	92	8	3	2	2	3	....	92	....	92
# 352-360	709	0.7	MMTACQGVG	91	9	3	3	2	3	....	91	....	91
# 353-361	808	0.7	MTACQGVGG	92	8	3	3	2	3	....	92	....	92
# 354-362	808	0.6	TACQGVGGP	92	8	4	2	2	2	....	92	....	92
& 355-363	807	1.2	ACQGVGGPG	64	36	33	1	2	3	....	64	....	64
& 356-364	805	1.3	CQGVGGPGH	64	36	33	1	2	3	....	64	....	64
& 357-365	794	1.2	QGVGGPGHK	64	36	33	1	2	3	....	64	....	64
& 358-366	794	1.2	GVGGPGHKA	64	36	32	1	2	3	....	64	....	64
& 359-367	779	1.4	VGGPGHKAR	63	37	30	5	2	3	....	63	....	63
& 360-368	819	1.7	GGPGHKARV	62	38	26	11	<1	2	....	62	....	62
& 361-369	844	1.7	GPGHKARVL	63	37	25	11	<1	2	....	63	....	63
& 362-370	563	1.7	PGHKARVLA	63	37	23	13	2	3	....	63	....	63
& 363-371	549	1.8	GHKARVLAE	62	38	23	13	2	3	....	62	....	62
& 364-372	562	0.9	HKARVLAEA	85	15	8	4	2	3	....	85	....	85
& 365-373	572	0.9	KARVLAEAM	85	15	8	4	2	3	....	85	....	85
& 366-374	584	1.1	ARVLAEAMS	82	18	8	7	3	4	....	82	....	82
& 367-375	586	1.4	RVLAEAMSQ	80	20	8	10	3	4	....	80	........H	1
& 368-376	584	2.4	VLAEAMSQV	57	43	11	28	3	5	....	57	.......H.	<1
& 369-377	584	3.0	LAEAMSQVT	44	56	16	36	3	7	....	44	......H..	<1
& 370-378	574	3.9	AEAMSQVTN	33	67	16	44	7	13	....	33	.....H...	<1
& 371-379	133	2.4	EAMSQVSNT	67	33	3	17	14	20	......T.-	0	....H.T.-	0
& 372-380	130	2.5	AMSQVSNTA	67	33	2	13	18	24	.....T.-S	0	...H.T.-P	0
& 373-381	130	2.5	MSQVSNTAA	67	33	2	12	19	25	....T.-S.	0	..H.T.-PV	0
& 374-382	128	2.8	SQVSNTAAA	63	38	4	10	23	28	...T.-S.T	0	.H.T.-PVN	0
& 375-383	128	3.3	QVSNTAAAT	52	48	9	16	23	30	..T.-S.TI	0	H.T.-PVNI	0
& 376-384	120	2.9	VSNTAAATM	56	44	9	17	18	24	.T.-S.TI.	0	.T.-PVNI.	0
& 377-385	120	2.9	SNTAAATMM	58	43	8	16	19	25	T.-S.TI..	0	T.-PVNI..	0
& 378-386	120	2.9	NTAAATMMQ	58	43	8	16	19	25	.-S.TI...	0	.-PVNI...	0
& 379-387	120	2.9	TAAATMMQR	58	43	8	17	18	24	-S.TI....	0	-PVNI....	0
+ 380-388	506	5.3	SATIMMQRG	16	84	14	55	15	25	....	16	PVN......	<1
& 381-389	562	4.9	ATIMMQRGN	21	79	14	56	10	19	....	21	VN......S	0
& 382-390	573	4.4	TIMMQRGNF	24	76	15	53	8	14	....	24	N......S.	0
& 383-391	673	3.8	IMMQRGNFR	28	72	18	46	8	12	....	28	......S..	0
& 384-392	763	3.9	MMQRGNFRN	28	72	19	47	6	11	....	28	.....S...	0
& 385-393	803	4.3	MQRGNFRNQ	28	72	16	49	7	13	....	28	....S....	0
& 386-394	807	4.5	QRGNFRNQR	26	74	12	53	9	15	....	26	...S.....	0
& 387-395	805	4.8	RGNFRNQRK	26	74	12	52	10	17	....	26	..S.....R	0
+ 388-396	806	5.1	GNFRNQRKI	18	82	14	57	12	19	....	18	.S.....RM	0
+ 389-397	779	5.2	NFRNQRKIV	16	84	13	58	13	20	....	16	S.....RM.	0
+ 390-398	778	5.2	FRNQRKIVK	16	84	13	58	12	19	....	16	.....RM..	0
+ 391-399	777	5.0	RNQRKIVKC	16	84	14	59	11	17	....	16	....RM...	0
+ 392-400	781	4.6	NQRKIVKCF	20	80	17	53	10	15	....	20	...RM....	0
& 393-401	784	4.0	QRKIVKCFN	28	72	19	47	7	12	....	28	..RM.....	0
& 394-402	783	3.8	RKIVKCFNC	28	72	18	49	5	9	....	28	.RM......	0
& 395-403	767	3.3	KIVKCFNCG	31	69	21	43	4	8	....	31	RM.......	0
& 396-404	765	3.2	IVKCFNCGK	28	72	22	45	4	7	....	28	M........	1
& 397-405	766	2.0	VKCFNCGKE	54	46	32	11	3	5	....	54	....	54
& 398-406	794	1.1	KCFNCGKEG	86	14	4	7	3	4	....	86	....	86
& 399-407	792	1.1	CFNCGKEGH	87	13	4	7	3	4	....	87	....	87
& 400-408	927	1.7	FNCGKEGHI	75	25	10	12	3	5	........T	1	....	75
& 401-409	932	1.7	NCGKEGHIA	75	25	10	12	3	5	.......T.	1	....	75
& 402-410	935	2.6	CGKEGHIAR	44	56	31	21	4	6	......T..	1	........K	31
& 403-411	923	2.5	GKEGHIARN	45	55	31	21	3	6	.....T...	1	.......K.	31
& 404-412	935	2.6	KEGHIARNC	45	55	30	21	3	6	....T....	1	......K..	30
& 405-413	942	2.8	EGHIAKNCR	33	67	32	31	4	6	...T.R...	1	....	33
& 406-414	942	2.5	GHIAKNCRA	35	65	34	28	3	5	..T.R....	1	....	35
& 407-415	938	2.5	HIAKNCRAP	36	64	34	28	3	5	.T.R.....	1	....	36
& 408-416	931	2.6	IAKNCRAPR	35	65	34	28	3	5	T.R......	1	....	35
& 409-417	942	2.0	ARNCRAPRK	42	58	38	16	3	4	....	42	.K.......	38
& 410-418	1055	2.7	RNCRAPRKK	36	64	30	31	4	5	....	36	K........	30
& 411-419	1031	1.9	NCRAPRKKG	65	35	13	19	3	4	....	65	....	65
& 412-420	1027	1.8	CRAPRKKGC	65	35	14	18	3	4	....	65	....	65
& 413-421	965	1.6	RAPRKKGCW	68	32	15	14	2	4	....	68	....	68
& 414-422	995	1.2	APRKKGCWK	80	20	13	6	2	3	....	80	....	80
& 415-423	987	1.2	PRKKGCWKC	81	19	12	5	2	3	....	81	....	81
& 416-424	978	1.2	RKKGCWKCG	80	20	12	5	2	3	....	80	....	80
& 417-425	926	1.9	KKGCWKCGK	63	37	14	21	2	3	....	63	....	63
& 418-426	923	1.9	KGCWKCGKE	63	37	14	22	1	3	....	63	....	63
& 419-427	923	1.5	GCWKCGKEG	74	26	14	10	2	3	....	74	....	74
& 420-428	907	1.5	CWKCGKEGH	74	26	14	10	2	3	....	74	....	74
& 421-429	866	1.6	WKCGKEGHQ	74	26	14	11	2	4	....	74	....	74

& 422-430	865	1.6	KCGKEGHQM	73	27	13	12	2	4	....	73	....	73
& 423-431	867	1.4	CGKEGHQMK	76	24	13	9	2	3	....	76	....	76
& 424-432	863	1.9	GKEGHQMKD	63	37	13	22	2	4	....	63	....	63
& 425-433	859	1.9	KEGHQMKDC	63	37	13	22	2	4	....	63	....	63
& 426-434	859	1.7	EGHQMKDCT	72	28	14	13	2	4	....	72	........N	<1
& 427-435	854	1.8	GHQMKDCTE	71	29	14	13	2	4	....	71	.......N.	<1
& 428-436	855	1.8	HQMKDCTER	71	29	13	13	3	5	....	71	......N..	<1
& 429-437	857	1.8	QMKDCTERQ	71	29	13	13	2	4	....	71	.....N...	<1
& 430-438	865	2.4	MKDCTERQA	62	38	10	26	3	5	....	62	....N...V	0
& 431-439	872	2.2	KDCTERQAN	64	36	10	23	3	5	....	64	...N...V.	0
& 432-440	874	2.2	DCTERQANF	64	36	10	24	2	5	....	64	..N...V..	0
& 433-441	871	1.7	CTERQANFL	73	27	14	11	2	4	....	73	.N...V...	0
& 434-442	873	1.7	TERQANFLG	72	28	14	12	2	4	....	72	N...V....	0
& 435-443	877	1.3	ERQANFLGK	77	23	15	6	1	3	....	77	...V.....	15
& 436-444	878	1.4	RQANFLGKI	77	23	14	8	1	3	....	77	..V......	14
& 437-445	879	1.3	QANFLGKIW	78	22	14	7	1	3	....	78	.V.......	14
& 438-446	877	1.3	ANFLGKIWP	78	22	14	6	2	3	....	78	V........	14
# 439-447	875	0.6	NFLGKIWPS	93	7	2	4	1	2	....	93	....	93
& 440-448	885	2.0	FLGKIWPSH	58	42	24	15	3	4	........Y	24	....	58
& 441-449	883	2.1	LGKIWPSHK	57	43	24	16	4	5	.......Y.	24	....	57
& 442-450	882	2.2	GKIWPSHKG	57	43	23	16	4	6	......Y..	23	....	57
& 443-451	882	2.2	KIWPSHKGR	57	43	23	16	3	6	.....Y...	23	....	57
& 444-452	884	2.1	IWPSHKGRP	58	42	24	15	3	5	....Y....	24	....	58
& 445-453	881	2.0	WPSHKGRPG	59	41	24	14	3	5	...Y.....	24	....	59
& 446-454	880	1.9	PSHKGRPGN	60	40	24	14	3	4	..Y......	24	....	60
& 447-455	882	1.9	SHKGRPGNF	60	40	24	13	3	4	.Y.......	24	....	60
& 448-456	882	2.2	HKGRPGNFL	56	44	23	18	3	5	Y........	23	....	56
& 449-457	885	0.9	KGRPGNFLQ	89	11	3	6	2	3	....	89	....	89
& 450-458	887	1.7	GRPGNFLQS	63	37	27	8	2	4	....	63	........N	27
& 451-459	887	1.7	RPGNFLQSR	63	37	26	9	2	4	....	63	.......N.	26
& 452-460	886	1.8	PGNFLQSRP	63	37	25	10	2	4	....	63	......N..	25
& 453-461	883	1.9	GNFLQSRPE	62	38	25	11	2	5	....	62	.....N...	25
& 454-462	876	1.9	NFLQSRPEP	61	39	25	12	2	4	....	61	....N....	25
& 455-463	876	2.4	FLQSRPEPT	54	46	24	18	3	6	....	54	...N....S	1
& 456-464	942	2.5	LQSRPEPTA	54	46	23	20	4	6	....	54	..N....S.	<1
457-465 *	48	2.2	QSRPEQSRP	38	63	31	19	13	17	.....PTA-	0	.N...P.A-	0
458-466 *	46	2.4	SRPEQSRPE	39	61	30	17	13	20	....PTA--	0	N...P.A--	0
459-467 *	47	2.0	RPEPTAPEP	43	57	38	4	15	19	......---	0	....S.---	0
460-468 *	46	2.1	PEQSRPEPT	39	61	39	4	17	22	..PTA----	0	..P.A----	0
461-469 *	46	2.2	EQSRPEPTA	39	61	37	4	20	24	.PTA-----	0	.P.A-----	0
462-470 *	46	2.1	PTAPEPTAP	39	61	39	4	17	22	..------.	0	.S.-----.	0
463-471 *	57	2.2	SRPEPTAPP	46	54	32	7	16	21	TA-----..	0	.A-----..	0
464-472 *	57	2.4	RPEPTAPPE	47	53	23	12	18	23	A------...	0	A-----..A	0
465-473 *	57	1.7	PEPTAPPEE	70	30	9	9	12	18	-----....	0	-----..A.	0
466-474 *	57	1.5	EPTAPPEES	72	28	9	14	5	12	----.....	0	----..A..	0
& 467-475	204	2.4	PPAPPEESF	47	53	25	23	5	9	---......	0	---..A..L	0
& 468-476	204	2.7	PAPPEESFR	45	55	24	25	6	12	--.......	0	--..A..LG	0
& 469-477	204	1.7	APPEESFRF	75	25	7	12	6	9	-.......S	0	-..A..LG.	0
& 470-478	955	2.6	PPEESFRFG	57	43	18	22	3	6	.......S.	<1	..A..LG..	0
& 471-479	950	2.7	PEESFRFGE	56	44	18	22	4	7	......S.V	<1	.A..LG...	0
& 472-480	945	2.9	EESFRFGEE	53	47	18	24	5	8	.....S.V.	<1	A..LG....	0
& 473-481	903	2.9	ESFRFGEET	51	49	19	26	5	8	....S.V..	<1	..LG....A	0
& 474-482	903	3.5	SFRFGEETT	39	61	19	37	6	10	...S.V...	<1	.LG....A.	0
& 475-483	902	3.8	FRFGEETTT	33	67	19	42	7	11	..S.V....	<1	LG....A..	0
& 476-484	899	3.7	RFGEETTTP	33	67	19	41	6	10	.S.V.....	<1	G....A...	0
& 477-485	889	3.9	FGEETTTPS	27	73	19	47	7	11	S.V.....P	<1	....A...P	<1
& 478-486	882	3.7	GEETTTPSQ	30	70	19	45	6	10	.V.....P.	<1	...A...P.	<1
& 479-487	888	3.7	EETTTPSQK	30	70	19	45	6	10	V.....P..	<1	..A...P..	<1
& 480-488	885	3.8	ETTTPSQKQ	30	70	18	46	6	10	.....P...	11	.A...P...	<1
& 481-489	884	3.9	TTTPSQKQE	29	71	18	47	6	10	....P....	11	A...P....	<1
+ 482-490	907	4.5	AAPSSKQEP	18	82	15	59	8	13	TT.PQ....	10	TT.PQ...T	<1
+ 483-491	881	4.7	APSSKQEPK	18	82	15	57	10	15	T.PQ....I	8	T.PQ...TV	0
& 484-492	875	4.4	PSQKQEPID	24	76	18	50	9	14	.P.......	7	.P....TV.	0
& 485-493	865	4.5	SQKQEPIDK	22	78	18	51	9	14	P........	8	P....TV..	0
& 486-494	872	4.4	QKQEPIDKE	26	74	18	49	8	13	....	26	....TV...	<1
& 487-495	863	4.9	KQEPIDKEL	21	79	18	52	10	16	....	21	...TV....	<1
& 488-496	862	4.9	QEPIDKELY	21	79	18	52	10	16	....	21	..TV.....	<1
& 489-497	862	4.8	EPIDKELYP	21	79	18	52	9	15	....	21	.TV......	<1
& 490-498	853	4.8	PIDKELYPL	22	78	17	52	9	15	....	22	TV.......	<1
& 491-499	837	4.5	IDKELYPLA	28	72	16	46	9	15	........T	5	V.......T	<1
& 492-500	855	4.0	DKELYPLAS	34	66	16	43	7	11	.......T.	6	.......T.	6

	[&] 493-501	540	3.6	KELYPLASL	44	56	11	37	8	13	......T..	11	......T..	11
	[&] 494-502	518	4.1	ELYPLASLR	26	74	18	48	8	14	.....T...	7	.....T...	7
	[&] 495-503	520	3.7	LYPLASLRS	32	68	19	42	7	11	....T....	8	....T....	8
	[&] 496-504	526	2.8	YPLASLRSL	44	56	20	30	6	8	...T.....	12	...T.....	12
	[&] 497-505	544	2.8	PLASLRSLF	42	58	19	34	4	7	..T......	12	..T......	12
	[&] 498-506	543	2.8	LASLRSLFG	42	58	20	34	5	7	.T.......	12	.T.......	12
	[&] 499-507	552	3.1	ASLRSLFGN	26	74	18	53	4	6	T........	14	T.......S	<1
	[&] 500-508	556	2.2	SLRSLFGND	40	60	29	28	3	4	....	40	.......S.	18
	[&] 501-509	554	2.1	LRSLFGNDP	44	56	29	24	3	4	....	44	......S..	18
	[&] 502-510	416	2.0	RSLFGNDPS	52	48	28	17	4	5	....	52	.....S...	<1
	[&] 503-511	414	1.2	SLFGNDPSS	80	20	12	5	4	5	....	80	....S....	2
	[&] 504-512	410	1.6	LFGNDPSSQ	73	27	12	11	4	6	....	73	...S.....	2
Pol	[&] 1-9	237	2.3	FFRENLAFP	45	55	34	16	5	9	.L..D...L	<1	....	45
	[&] 2-10	247	2.3	FRENLAFPQ	45	55	33	17	5	9	L..D...L.	<1	....	45
	[&] 3-11	245	2.5	RENLAFPQG	43	57	31	20	6	11	..D...L..	6	....	43
	[&] 4-12	247	3.4	ENLAFPQGK	32	68	17	43	8	13	.D...L...	5	....	32
	[&] 5-13	262	3.2	NLAFPQGKA	32	68	18	43	6	11	D...L....	5	....	32
	[&] 6-14	269	2.6	LAFPQGKAR	48	52	25	22	4	9	...L.....	5	....	48
	[&] 7-15	267	2.7	AFPQGKARE	46	54	25	23	6	10	..L......	5	....	46
	[&] 8-16	265	3.2	FPQGKAREF	41	59	20	32	7	12	.L.......	4	....	41
	[&] 9-17	267	4.0	PQGKAREFS	24	76	16	51	9	17	L........	3	....	24
	[&] 10-18	265	3.8	QGKAREFSS	26	74	18	46	9	17	....	26	....	26
	[&] 11-19	267	3.9	GKAREFSSE	24	76	19	48	9	18	....	24	....	24
	[&] 12-20	471	2.8	KAREFSSEQ	56	44	11	27	6	10	....	56	....	56
	[&] 13-21	468	2.8	AREFSSEQT	59	41	11	24	6	11	....	59	....	59
	[&] 14-22	469	2.7	REFSSEQTR	59	41	11	24	6	11	....	59	....	59
	[&] 15-23	468	2.9	EFSSEQTRA	56	44	11	26	6	11	....	56	....	56
	[&] 16-24	465	3.5	FSSEQTRAN	40	60	17	37	6	13	....	40	I	17
	[&] 17-25	465	3.2	SSEQTRANS	44	56	16	35	5	11	....	44	.......I.	16
	[&] 18-26	471	2.7	SEQTRANSP	55	45	17	22	6	10	....	55	......I..	17
	[&] 19-27	474	2.5	EQTRANSPT	57	43	18	19	6	9	....	57	.....I...	18
	[&] 20-28	474	2.8	QTRANSPTR	52	48	16	25	7	10	....	52	....I...S	1
	[&] 21-29	467	3.4	TRANSPTRR	32	68	21	40	7	11	....	32	...I...S.	1
	[&] 22-30	475	3.0	RANSPTRRE	36	64	25	33	6	10	....	36	..I...S..	1
	[&] 23-31	476	3.2	ANSPTRREL	34	66	24	36	7	11	....	34	.I...S...	2
	[&] 24-32	494	3.2	NSPTRRELQ	34	66	24	35	7	11	....	34	I...S...R	0
	[&] 25-33	434	2.6	SPTRRELQV	45	55	32	17	7	10	....	45	...S...R.	<1
	[&] 26-34	433	2.6	PTRRELQVW	45	55	32	17	7	10	....	45	..S...R..	<1
	[&] 27-35	452	2.5	TRRELQVWG	46	54	31	17	6	9	....	46	.S...R...	<1
	[&] 28-36	448	3.0	RRELQVWGR	33	67	29	32	6	9	....	33	S...R...G	<1
	[&] 29-37	477	2.9	RELQVWGRD	35	65	28	33	4	9	....	35	...R...GG	0
	[&] 30-38	468	2.6	ELQVWGRDN	55	45	18	21	5	10	....	55	..R...GG.	0
	[&] 31-39	468	3.4	LQVWGRDNN	37	63	18	39	6	12	....	37	.R...GG..	0
	[&] 32-40	485	3.8	QVWGRDNNS	28	72	17	49	5	12	....	28	R...GG...	0
	[&] 33-41	484	4.6	VWGRDNNSL	23	77	15	54	9	17	........P	2	...GG...P	0
	[&] 34-42	486	4.5	WGRDNNSLS	23	77	15	54	8	17	.......P.	2	..GG...P.	0
	[&] 35-43	573	4.6	GRDNNSLSE	26	74	13	52	9	17	......P..	2	.GG...P..	0
	[&] 36-44	590	4.9	RDNNSLSEA	24	76	12	55	9	18	.....P...	4	GG...P...	0
	[&] 37-45	618	4.5	DNNSLSEAG	28	72	13	51	8	15	....P....	8	G...P....	<1
	[+] 38-46	622	5.0	NNSLSEAGA	15	85	12	65	9	17	...P.....	7	...P....D	<1
	[+] 39-47	671	5.3	NSLSEAGAD	16	84	9	65	10	19	..P......	7	..P....DS	0
	[+] 40-48	700	5.2	SLSEAGADR	20	80	10	62	9	18	.P.......	10	.P....DS.	0
	[+] 41-49	699	5.1	LSEAGADRQ	20	80	9	62	9	17	P........	9	P....DS..	0
	[&] 42-50	704	4.4	SEAGADRQG	33	67	9	51	6	13	....	33	....DS...	0
	[&] 43-51	730	5.4	EAGADRQGT	24	76	8	59	9	19	....	24	...DS....	0
	[&] 44-52	733	5.7	AGADRQGTV	22	78	7	60	11	21	....	22	..DS.....	0
	[&] 45-53	735	5.5	GADRQGTVS	23	77	7	60	10	20	....	23	.DS......	0
	[+] 46-54	732	5.9	ADRQGTVSF	17	83	7	63	14	24	....	17	DS.......	0
	[+] 47-55	743	5.9	DRQGTVSFS	17	83	7	62	14	24	........N	3	S.......D	0
	[&] 48-56	749	5.4	RQGTVSFSF	26	74	7	55	12	21	.......N.	3	.......DL	0
	[&] 49-57	759	5.2	QGTVSFSFP	27	73	7	56	9	18	......N..	3	......DL.	0
	[&] 50-58	763	4.9	GTVSFSFPQ	29	71	7	55	8	16	.....N...	3	.....DL..	0
	[&] 51-59	842	5.0	TVSFSFPQI	29	71	7	57	7	14	....N...V	<1	....DL...	0
	[&] 52-60	865	3.5	VSFSFPQIT	45	55	8	44	3	7	...N...V.	<1	...DL....	<1
	[&] 53-61	882	2.9	SFSFPQITL	50	50	8	39	2	5	..N...V..	<1	..DL.....	<1
	[&] 54-62	971	2.8	FSFPQITLW	51	49	8	39	2	4	.N...V...	<1	.DL......	<1
	[&] 55-63	976	2.1	SFPQITLWQ	57	43	14	27	1	2	N...V....	<1	DL.......	<1
	[&] 56-64	1078	0.8	FPQITLWQR	84	16	14	<1	<1	1	...V.....	<1	L........	14
	[#] 57-65	7010	0.4	PQITLWQRP	96	4	<1	2	<1	1	..V......	1	....	96
	[&] 58-66	6829	1.5	QITLWQRPL	70	30	21	9	<1	2	.V.......	1	....	70
	[&] 59-67	6894	1.6	ITLWQRPLV	70	30	20	9	<1	2	V........	1	....	70

[&] 60-68	10152	2.3	TLWQRPLVT	62	38	18	19	1	2	....	62	....	62
[&] 61-69	10138	3.0	LWQRPLVTI	53	47	14	31	2	3	....	53	....	53
[&] 62-70	10290	3.4	WQRPLVTIK	49	51	13	36	2	4	....	49	....	49
[&] 63-71	10240	3.9	QRPLVTIKI	42	58	12	44	2	4	....	42	....	42
[&] 64-72	10294	4.2	RPLVTIKIG	41	59	12	45	3	5	....	41	....	41
[&] 65-73	10368	4.2	PLVTIKIGG	40	60	11	46	3	5	....	40	....	40
[&] 66-74	10472	4.4	LVTIKIGGQ	39	61	11	46	3	5	....	39	....	39
[&] 67-75	10642	3.9	VTIKIGGQL	50	50	9	39	3	5	....	50	....	50
[&] 68-76	10554	4.2	TIKIGGQLK	48	52	8	41	3	6	....	48	I	<1
[&] 69-77	10691	3.6	IKIGGQLKE	51	49	9	38	2	4	....	51	.......I.	<1
[&] 70-78	10924	3.0	KIGGQLKEA	59	41	8	31	2	3	....	59	......I..	1
[&] 71-79	11126	2.6	IGGQLKEAL	65	35	10	24	1	3	....	65	.....I...	1
[&] 72-80	11316	2.1	GGQLKEALL	73	27	6	20	1	2	....	73	....I....	2
[&] 73-81	11375	1.8	GQLKEALLD	77	23	6	16	1	2	....	77	...I.....	2
[&] 74-82	11393	1.7	QLKEALLDT	78	22	6	15	<1	2	....	78	..I......	2
[&] 75-83	11460	1.6	LKEALLDTG	79	21	6	14	<1	1	....	79	.I.......	2
[&] 76-84	11671	0.9	KEALLDTGA	89	11	3	8	<1	1	....	89	I........	2
[#] 77-85	11853	0.5	EALLDTGAD	95	5	3	2	<1	1	....	95	....	95
[#] 78-86	12120	0.6	ALLDTGADD	93	7	3	4	<1	1	....	93	....	93
[#] 79-87	12133	0.6	LLDTGADDT	93	7	3	4	<1	1	....	93	....	93
[#] 80-88	12149	0.6	LDTGADDTV	92	8	3	5	<1	1	....	92	....	92
[&] 81-89	12074	0.9	DTGADDTVL	89	11	3	8	<1	1	....	89	....	89
[&] 82-90	12069	1.0	TGADDTVLE	88	12	2	9	<1	1	....	88	....	88
[&] 83-91	11875	1.7	GADDTVLEE	72	28	16	11	<1	1	....	72	........D	16
[&] 84-92	11558	2.2	ADDTVLEEM	65	35	13	22	<1	2	....	65	.......DI	3
[&] 85-93	11135	3.8	DDTVLEEMN	46	54	9	43	2	3	........S	9	......DID	<1
[&] 86-94	11118	3.8	DTVLEEMNL	46	54	9	43	2	3	.......S.	9	.....DID.	<1
[&] 87-95	11125	3.8	TVLEEMNLP	45	55	9	44	2	3	......S..	9	....DID..	<1
[&] 88-96	11126	3.9	VLEEMNLPG	45	55	9	44	2	3	.....S...	9	...DID...	<1
[&] 89-97	10947	4.4	LEEMNLPGR	37	63	9	52	2	4	....S....	7	..DID....	<1
[&] 90-98	11036	4.1	EEMNLPGRW	39	61	9	50	1	3	...S.....	7	.DID.....	<1
[&] 91-99	10981	4.2	EMNLPGRWK	39	61	9	51	2	3	..S......	7	DID......	<1
[&] 92-100	11085	3.6	MNLPGRWKP	44	56	9	46	1	2	.S.......	8	ID.......	2
[&] 93-101	11307	3.1	NLPGRWKPK	49	51	11	39	<1	2	S........	9	D........	9
[&] 94-102	11531	2.2	LPGRWKPKM	62	38	15	22	<1	1	....	62	I	8
[&] 95-103	11547	2.2	PGRWKPKMI	62	38	15	22	<1	1	....	62	.......I.	7
[&] 96-104	11593	2.2	GRWKPKMIG	63	37	16	21	<1	1	....	63	......I..	7
[&] 97-105	11594	2.2	RWKPKMIGG	63	37	15	21	<1	1	....	63	.....I...	7
[&] 98-106	11885	1.5	WKPKMIGGI	79	21	9	12	<1	1	....	79	....I....	9
[&] 99-107	11886	1.5	KPKMIGGIG	79	21	8	12	<1	1	....	79	...I.....	8
[&] 100-108	11997	1.3	PKMIGGIGG	81	19	9	10	<1	1	....	81	..I......	9
[&] 101-109	11925	1.4	KMIGGIGGF	81	19	8	10	<1	1	....	81	.I.......	8
[&] 102-110	11875	1.7	MIGGIGGFI	79	21	6	14	<1	2	....	79	I........	6
[&] 103-111	12013	1.2	IGGIGGFIK	86	14	5	8	<1	1	....	86	........R	<1
[&] 104-112	12062	1.2	GGIGGFIKV	86	14	6	8	<1	1	....	86	.......R.	<1
[&] 105-113	11889	1.5	GIGGFIKVR	79	21	8	13	<1	1	....	79	......R..	<1
[&] 106-114	11851	1.6	IGGFIKVRQ	78	22	8	14	<1	1	....	78	.....R...	<1
[&] 107-115	11844	1.5	GGFIKVRQY	78	22	8	14	<1	1	....	78	....R....	<1
[&] 108-116	11743	1.9	GFIKVRQYD	73	27	7	19	<1	2	....	73	...R.....	<1
[&] 109-117	11653	2.2	FIKVRQYDQ	71	29	6	22	1	2	....	71	..R.....H	0
[&] 110-118	11329	3.0	IKVRQYDQI	56	44	15	28	1	3	....	56	.R.....HV	0
[&] 111-119	10856	4.2	KVRQYDQIP	31	69	18	49	2	4	L	18	R.....HV.	0
[&] 112-120	10676	4.9	VRQYDQIPI	27	73	11	59	2	5	.......L.	11	.....HV..	<1
[&] 113-121	10641	5.1	RQYDQIPIE	26	74	11	60	3	5	......L..	11	....HV...	<1
[&] 114-122	10712	4.8	QYDQIPIEI	29	71	12	56	2	5	.....L...	12	...HV....	<1
[&] 115-123	10705	4.8	YDQIPIEIC	29	71	12	56	3	5	....L....	12	..HV.....	<1
[&] 116-124	10708	4.8	DQIPIEICG	29	71	12	56	3	5	...L.....	12	.HV......	<1
[&] 117-125	10718	4.8	QIPIEICGH	30	70	11	55	3	5	..L......	11	HV......Q	0
[&] 118-126	10732	4.7	IPIEICGHK	30	70	12	55	3	5	.L.......	12	V......Q.	<1
[&] 119-127	10721	5.0	PIEICGHKA	26	74	12	59	3	5	L........	12	......Q..	<1
[&] 120-128	10982	4.0	IEICGHKAI	42	58	13	42	2	4	....	42	.....Q...	1
[&] 121-129	11210	3.3	EICGHKAIG	58	42	7	33	2	4	....	58	....Q...T	0
[&] 122-130	11184	3.3	ICGHKAIGT	59	41	6	33	2	4	....	59	...Q...T.	0
[&] 123-131	11209	3.3	CGHKAIGTV	59	41	7	33	2	4	....	59	..Q...T..	0
[&] 124-132	11254	3.2	GHKAIGTVL	59	41	6	33	2	3	....	59	.Q...T...	0
[&] 125-133	10882	4.0	HKAIGTVLV	44	56	15	39	2	4	....	44	Q...T...I	0
[&] 126-134	10933	3.6	KAIGTVLVG	47	53	16	35	2	4	....	47	...T...I.	<1
[&] 127-135	10962	3.5	AIGTVLVGP	49	51	17	33	2	4	....	49	..T...I..	<1
[&] 128-136	11261	2.7	IGTVLVGPT	55	45	22	22	1	2	....	55	.T...I...	<1
[&] 129-137	11602	1.9	GTVLVGPTP	61	39	28	11	<1	1	....	61	T...I....	<1
[&] 130-138	11549	2.3	TVLVGPTPV	55	45	26	18	<1	1	....	55	...I.....	26

& 131-139	11625	2.1	VLVGPTPVN	55	45	27	17	<1	1	....	55	..I......	27
& 132-140	11581	2.4	LVGPTPVNI	54	46	26	20	<1	1	....	54	.I.......	26
& 133-141	11556	2.4	VGPTPVNII	54	46	25	20	<1	2	....	54	I........	25
& 134-142	12019	1.5	GPTPVNIIG	79	21	8	12	<1	1	....	79	....	79
& 135-143	11997	1.5	PTPVNIIGR	79	21	8	12	<1	1	....	79	....	79
& 136-144	11891	1.6	TPVNIIGRN	77	23	8	14	<1	1	....	77	....	77
& 137-145	11820	1.8	PVNIIGRNL	76	24	8	15	<1	1	....	76	....	76
& 138-146	11653	2.2	VNIIGRNLL	72	28	5	23	<1	2	....	72	........M	5
& 139-147	11745	1.6	NIIGRNLLT	78	22	9	13	<1	1	....	78	.......M.	9
& 140-148	11687	1.7	IIGRNLLTQ	77	23	8	14	<1	1	....	77	......M..	8
& 141-149	11516	2.4	IGRNLLTQI	53	47	25	21	<1	2	....	53	.....M...	5
& 142-150	11559	2.3	GRNLLTQIG	53	47	25	21	<1	1	....	53	....M....	5
& 143-151	11506	2.4	RNLLTQIGC	52	48	25	22	<1	2	....	52	...M.....	5
& 144-152	11615	2.4	NLLTQIGCT	52	48	25	22	<1	2	....	52	..M......	5
& 145-153	11633	2.2	LLTQIGCTL	53	47	26	20	<1	2	....	53	.M.......	5
& 146-154	11728	2.0	LTQIGCTLN	54	46	27	18	<1	1	....	54	M........	5
& 147-155	11594	1.5	TQIGCTLNF	59	41	35	5	<1	1	....	59	....	59
& 148-156	2933	1.5	QIGCTLNFP	62	38	32	5	1	2	....	62	....	62
& 149-157	2700	1.3	IGCTLNFPI	64	36	32	2	1	2	....	64	....	64
# 150-158	2737	0.4	GCTLNFPIS	97	3	<1	2	<1	2	....	97	....	97
# 151-159	2737	0.4	CTLNFPISP	96	4	<1	3	<1	2	....	96	....	96
# 152-160	2748	0.5	TLNFPISPI	96	4	<1	3	<1	2	....	96	....	96
# 153-161	2761	0.8	LNFPISPIE	92	8	2	5	<1	2	....	92	....	92
# 154-162	2787	0.9	NFPISPIET	91	9	2	6	1	2	....	91	....	91
# 155-163	2797	0.9	FPISPIETV	91	9	2	6	<1	2	....	91	....	91
& 156-164	7252	0.9	PISPIETVP	90	10	3	6	<1	1	....	90	....	90
# 157-165	7351	0.9	ISPIETVPV	90	10	3	6	<1	1	....	90	....	90
& 158-166	7220	1.0	SPIETVPVK	89	11	3	7	<1	1	....	89	....	89
& 159-167	7289	0.9	PIETVPVKL	90	10	3	6	<1	1	....	90	....	90
# 160-168	7091	0.9	IETVPVKLK	90	10	4	6	<1	1	....	90	....	90
# 161-169	7153	0.8	ETVPVKLKP	90	10	4	5	<1	1	....	90	....	90
# 162-170	7226	0.5	TVPVKLKPG	95	5	<1	3	<1	1	....	95	....	95
# 163-171	7228	0.5	VPVKLKPGM	95	5	<1	3	<1	1	....	95	....	95
# 164-172	7283	0.5	PVKLKPGMD	96	4	<1	3	<1	1	....	96	....	96
# 165-173	7422	0.5	VKLKPGMDG	96	4	<1	3	<1	1	....	96	....	96
# 166-174	7480	0.4	KLKPGMDGP	96	4	<1	3	<1	1	....	96	....	96
& 167-175	7447	0.9	LKPGMDGPK	83	17	14	2	<1	1	....	83	........R	14
& 168-176	7458	1.0	KPGMDGPKV	82	18	14	3	<1	1	....	82	.......R.	14
& 169-177	7551	1.0	PGMDGPKVK	82	18	14	4	<1	1	....	82	......R..	14
& 170-178	7626	1.0	GMDGPKVKQ	82	18	14	4	<1	1	....	82	.....R...	14
& 171-179	7679	1.0	MDGPKVKQW	82	18	14	4	<1	1	....	82	....R....	14
& 172-180	7732	1.0	DGPKVKQWP	82	18	14	3	<1	1	....	82	...R.....	14
& 173-181	8123	0.9	GPKVKQWPL	82	18	14	3	<1	1	....	82	..R......	14
& 174-182	8301	1.0	PKVKQWPLT	82	18	14	4	<1	1	....	82	.R.......	14
& 175-183	8649	1.1	KVKQWPLTE	81	19	13	5	<1	1	....	81	R........	13
# 176-184	8815	0.5	VKQWPLTEE	95	5	1	4	<1	1	....	95	....	95
# 177-185	8894	0.4	KQWPLTEEK	96	4	<1	3	<1	1	....	96	....	96
# 178-186	8967	0.4	QWPLTEEKI	96	4	<1	3	<1	1	....	96	....	96
# 179-187	9435	0.6	WPLTEEKIK	94	6	1	5	<1	1	....	94	....	94
# 180-188	9651	0.7	PLTEEKIKA	93	7	1	5	<1	1	....	93	....	93
# 181-189	9839	0.7	LTEEKIKAL	93	7	1	5	<1	1	....	93	....	93
& 182-190	9589	2.1	TEEKIKALV	66	34	11	22	<1	2	....	66	....	66
& 183-191	9702	2.2	EEKIKALVE	66	34	9	23	1	2	....	66	....	66
& 184-192	10053	2.2	EKIKALVEI	67	33	9	23	1	2	....	67	....	67
& 185-193	10466	2.1	KIKALVEIC	67	33	10	22	<1	2	....	67	....	67
& 186-194	10377	2.8	IKALVEICT	63	37	8	28	1	3	....	63	....	63
& 187-195	10460	2.9	KALVEICTE	63	37	8	28	2	3	....	63	....	63
& 188-196	10506	3.5	ALVEICTEM	48	52	16	35	2	3	....	48	L	16
& 189-197	10559	3.4	LVEICTEME	48	52	16	35	2	3	....	48	.......L.	16
& 190-198	10473	3.8	VEICTEMEK	48	52	11	38	2	4	....	48	......L..	11
& 191-199	11199	2.9	EICTEMEKE	61	39	11	26	2	3	....	61	.....L...	11
& 192-200	11290	2.8	ICTEMEKEG	62	38	11	26	1	2	....	62	....L....	11
& 193-201	16771	2.7	CTEMEKEGK	64	36	10	25	1	2	....	64	...L.....	10
& 194-202	17052	2.7	TEMEKEGKI	64	36	10	24	1	2	....	64	..L......	10
& 195-203	17544	2.2	EMEKEGKIS	66	34	13	20	<1	2	....	66	.L.......	13
& 196-204	17779	2.4	MEKEGKISK	62	38	13	24	<1	2	....	62	L........	13
& 197-205	19386	1.9	EKEGKISKI	75	25	5	19	<1	1	....	75	....	75
& 198-206	19448	1.9	KEGKISKIG	75	25	5	19	<1	1	....	75	....	75
& 199-207	19755	1.4	EGKISKIGP	80	20	5	14	<1	1	....	80	....	80
& 200-208	19898	1.1	GKISKIGPE	86	14	5	8	<1	1	....	86	....	86
& 201-209	19919	1.1	KISKIGPEN	86	14	5	8	<1	1	....	86	....	86

& 202-210	20000	1.0	ISKIGPENP	86	14	5	8	<1	1	....	86	....	86
& 203-211	19977	1.0	SKIGPENPY	86	14	5	8	<1	1	....	86	....	86
# 204-212	19970	0.7	KIGPENPYN	90	10	6	4	<1	1	....	90	....	90
# 205-213	20230	0.4	IGPENPYNT	96	4	1	3	<1	1	....	96	....	96
# 206-214	20304	0.3	GPENPYNTP	97	3	<1	2	<1	0	....	97	....	97
& 207-215	19953	0.8	PENPYNTPV	84	16	13	2	<1	1	....	84	....	84
& 208-216	19954	0.9	ENPYNTPVF	84	16	13	2	<1	1	....	84	....	84
& 209-217	19951	0.9	NPYNTPVFA	83	17	14	4	<1	1	....	83	V	2
& 210-218	19936	0.9	PYNTPVFAI	82	18	14	4	<1	1	....	82	.......V.	2
& 211-219	19780	1.1	YNTPVFAIK	81	19	13	5	<1	1	....	81	......V..	2
& 212-220	19748	1.2	NTPVFAIKK	80	20	13	7	<1	1	....	80	.....V...	2
& 213-221	19844	1.2	TPVFAIKKK	80	20	13	7	<1	1	....	80	....V....	2
& 214-222	19523	2.0	PVFAIKKKD	65	35	13	21	<1	1	....	65	...V.....	1
& 215-223	19461	2.3	VFAIKKKDS	62	38	13	24	<1	1	....	62	..V......	<1
& 216-224	19508	2.2	FAIKKKDST	68	32	12	19	<1	2	....	68	.V.......	<1
& 217-225	19227	2.6	AIKKKDSTK	66	34	6	27	1	2	....	66	V........	<1
& 218-226	19331	2.5	IKKKDSTKW	67	33	6	26	<1	2	....	67	....	67
& 219-227	19310	2.5	KKKDSTKWR	67	33	6	26	<1	2	....	67	....	67
& 220-228	19454	2.4	KKDSTKWRK	68	32	7	25	<1	2	....	68	....	68
& 221-229	19249	2.6	KDSTKWRKL	66	34	5	28	1	2	....	66	....	66
& 222-230	19151	2.8	DSTKWRKLV	66	34	5	28	1	3	....	66	........T	<1
& 223-231	19368	2.3	STKWRKLVD	70	30	7	22	<1	2	....	70	.......T.	<1
& 224-232	19471	2.1	TKWRKLVDF	73	27	7	19	<1	2	....	73	......T..	<1
& 225-233	19751	1.5	KWRKLVDFR	77	23	12	11	<1	1	....	77	.....T...	<1
& 226-234	20066	0.9	WRKLVDFRE	88	12	4	7	<1	1	....	88	....T....	<1
& 227-235	20053	0.9	RKLVDFREL	88	12	4	7	<1	1	....	88	...T.....	<1
& 228-236	20088	0.9	KLVDFRELN	89	11	4	7	<1	1	....	89	..T......	<1
& 229-237	20043	1.0	LVDFRELNK	88	12	4	7	<1	1	....	88	.T.......	<1
& 230-238	20085	1.2	VDFRELNKR	79	21	14	6	<1	1	....	79	T........	<1
& 231-239	20234	0.9	DFRELNKRT	83	17	14	3	<1	1	....	83	....	83
& 232-240	20313	0.9	FRELNKRTQ	83	17	14	3	<1	1	....	83	....	83
& 233-241	20302	0.9	RELNKRTQD	83	17	14	2	<1	1	....	83	....	83
& 234-242	20274	0.9	ELNKRTQDF	83	17	14	2	<1	1	....	83	....	83
& 235-243	20275	1.0	LNKRTQDFW	82	18	14	3	<1	1	....	82	....	82
& 236-244	20272	1.0	NKRTQDFWE	82	18	14	3	<1	1	....	82	....	82
& 237-245	20112	1.1	KRTQDFWEV	80	20	14	6	<1	1	....	80	....	80
& 238-246	20152	1.1	RTQDFWEVQ	81	19	14	5	<1	1	....	81	....	81
# 239-247	20402	0.5	TQDFWEVQL	95	5	2	3	<1	0	....	95	....	95
# 240-248	20415	0.5	QDFWEVQLG	95	5	2	3	<1	0	....	95	....	95
# 241-249	20408	0.5	DFWEVQLGI	94	6	2	3	<1	0	....	94	....	94
# 242-250	20454	0.4	FWEVQLGIP	95	5	2	2	<1	0	....	95	....	95
# 243-251	20430	0.4	WEVQLGIPH	95	5	2	2	<1	0	....	95	....	95
# 244-252	20484	0.3	EVQLGIPHP	96	4	3	1	<1	0	....	96	....	96
& 245-253	20263	0.8	VQLGIPHPA	88	12	6	6	<1	0	....	88	........S	6
# 246-254	20407	0.7	QLGIPHPAG	91	9	6	3	<1	0	....	91	.......S.	6
& 247-255	20312	0.8	LGIPHPAGL	89	11	6	5	<1	0	....	89	......S..	6
& 248-256	20018	1.2	GIPHPAGLK	84	16	5	10	<1	1	....	84	.....S...	5
& 249-257	19699	1.6	IPHPAGLKK	80	20	5	14	<1	1	....	80	....S....	5
& 250-258	19288	2.2	PHPAGLKKK	71	29	8	20	<1	1	....	71	...S.....	4
& 251-259	19116	2.4	HPAGLKKKK	69	31	8	22	<1	2	....	69	..S......	4
& 252-260	19157	2.4	PAGLKKKKS	69	31	8	23	<1	2	....	69	.S.......	4
& 253-261	19044	2.6	AGLKKKKSV	67	33	8	25	<1	2	....	67	S........	4
& 254-262	19181	2.1	GLKKKKSVT	72	28	9	19	<1	2	....	72	....	72
& 255-263	19058	2.2	LKKKKSVTV	71	29	8	20	<1	2	....	71	....	71
& 256-264	19101	2.2	KKKKSVTVL	71	29	9	18	<1	2	....	71	....	71
& 257-265	19278	1.8	KKKSVTVLD	74	26	10	15	<1	1	....	74	....	74
& 258-266	19507	1.6	KKSVTVLDV	77	23	11	12	<1	1	....	77	....	77
& 259-267	19978	0.9	KSVTVLDVG	90	10	2	8	<1	1	....	90	....	90
# 260-268	20006	0.7	SVTVLDVGD	92	8	2	6	<1	1	....	92	....	92
# 261-269	19996	0.7	VTVLDVGDA	92	8	2	5	<1	1	....	92	....	92
# 262-270	20126	0.6	TVLDVGDAY	94	6	2	4	<1	1	....	94	....	94
# 263-271	20159	0.6	VLDVGDAYF	93	7	2	4	<1	1	....	93	....	93
# 264-272	20336	0.5	LDVGDAYFS	95	5	<1	4	<1	1	....	95	....	95
& 265-273	19978	1.0	DVGDAYFSV	84	16	11	4	<1	1	....	84	....	84
& 266-274	20009	1.0	VGDAYFSVP	84	16	11	4	<1	1	....	84	....	84
& 267-275	20010	0.9	GDAYFSVPL	85	15	11	4	<1	1	....	85	....	85
& 268-276	19885	1.3	DAYFSVPLD	80	20	11	9	<1	1	....	80	....	80
& 269-277	19322	2.4	AYFSVPLDK	53	47	23	23	<1	1	........E	23	....	53
& 270-278	18934	3.4	YFSVPLDKD	35	65	18	46	<1	2	.......E.	17	....	35
& 271-279	18957	3.3	FSVPLDKDF	35	65	18	46	<1	2	......E..	17	....	35
& 272-280	18929	3.3	SVPLDKDFR	35	65	19	46	<1	1	.....E...	17	....	35

& 273-281	18937	3.3	VPLDKDFRK	35	65	19	46	<1	1	....E....	17	....	35
& 274-282	19242	2.8	PLDKDFRKY	37	63	22	40	<1	1	...E.....	22	....	37
& 275-283	19239	2.8	LDKDFRKYT	37	63	22	40	<1	1	..E......	22	....	37
& 276-284	19264	2.8	DKDFRKYTA	37	63	22	40	<1	1	.E.......	22	....	37
& 277-285	19339	2.5	KDFRKYTAF	37	63	28	35	<1	1	E........	28	....	37
& 278-286	19868	1.3	DFRKYTAFT	69	31	25	6	<1	1	....	69	....	69
# 279-287	20343	0.2	FRKYTAFTI	99	1	<1	1	<1	<1	....	99	....	99
# 280-288	20352	0.2	RKYTAFTIP	99	1	<1	1	<1	<1	....	99	....	99
# 281-289	20183	0.2	KYTAFTIPS	98	2	<1	1	<1	<1	....	98	....	98
& 282-290	19550	1.8	YTAFTIPSI	58	42	26	16	<1	1	....	58	....	58
& 283-291	19554	1.8	TAFTIPSIN	58	42	26	16	<1	1	....	58	....	58
& 284-292	19483	1.8	AFTIPSINN	58	42	26	16	<1	1	....	58	....	58
& 285-293	19337	2.0	FTIPSINNE	56	44	25	18	<1	1	....	56	....	56
& 286-294	19221	2.2	TIPSINNET	56	44	24	19	<1	1	....	56	....	56
& 287-295	19225	2.2	IPSINNETP	56	44	24	19	<1	1	....	56	....	56
& 288-296	19192	2.2	PSINNETPG	56	44	25	19	<1	1	....	56	....	56
& 289-297	18793	2.7	SINNETPGI	50	50	22	27	<1	2	....	50	....	50
& 290-298	18848	2.7	INNETPGIR	50	50	22	27	<1	2	....	50	....	50
& 291-299	19417	1.1	NNETPGIRY	85	15	7	8	<1	1	....	85	....	85
& 292-300	19373	1.1	NETPGIRYQ	85	15	7	8	<1	1	....	85	....	85
& 293-301	19423	1.1	ETPGIRYQY	85	15	7	8	<1	1	....	85	....	85
& 294-302	19546	0.9	TPGIRYQYN	87	13	7	6	<1	1	....	87	....	87
& 295-303	19640	0.8	PGIRYQYNV	88	12	7	4	<1	1	....	88	....	88
& 296-304	19630	0.8	GIRYQYNVL	88	12	7	4	<1	1	....	88	....	88
& 297-305	19642	0.8	IRYQYNVLP	88	12	7	4	<1	1	....	88	....	88
# 298-306	20066	0.3	RYQYNVLPQ	96	4	2	1	<1	<1	....	96	....	96
# 299-307	20112	0.3	YQYNVLPQG	97	3	2	1	<1	<1	....	97	....	97
# 300-308	20116	0.3	QYNVLPQGW	97	3	2	1	<1	<1	....	97	....	97
# 301-309	20187	0.3	YNVLPQGWK	97	3	2	1	<1	<1	....	97	....	97
# 302-310	20220	0.3	NVLPQGWKG	97	3	2	<1	<1	<1	....	97	....	97
# 303-311	20275	0.3	VLPQGWKGS	97	3	2	<1	<1	<1	....	97	....	97
# 304-312	20299	0.3	LPQGWKGSP	97	3	2	1	<1	<1	....	97	....	97
# 305-313	20222	0.5	PQGWKGSPA	95	5	2	3	<1	<1	....	95	....	95
# 306-314	20254	0.5	QGWKGSPAI	94	6	2	3	<1	<1	....	94	....	94
# 307-315	20287	0.3	GWKGSPAIF	96	4	2	1	<1	<1	....	96	....	96
# 308-316	20314	0.3	WKGSPAIFQ	96	4	2	1	<1	<1	....	96	....	96
& 309-317	19890	1.6	KGSPAIFQS	72	28	15	13	<1	1	....	72	....	72
& 310-318	19907	1.6	GSPAIFQSS	72	28	15	13	<1	1	....	72	....	72
& 311-319	19912	1.6	SPAIFQSSM	72	28	15	13	<1	1	....	72	....	72
& 312-320	19797	1.8	PAIFQSSMT	70	30	14	15	<1	1	....	70	....	70
& 313-321	19576	2.2	AIFQSSMTK	63	37	14	22	<1	1	....	63	....	63
& 314-322	19607	2.0	IFQSSMTKI	64	36	15	20	<1	1	....	64	....	64
& 315-323	19605	2.0	FQSSMTKIL	65	35	15	20	<1	1	....	65	....	65
& 316-324	19548	2.3	QSSMTKILE	62	38	13	25	<1	1	....	62	....	62
& 317-325	19535	2.3	SSMTKILEP	62	38	13	25	<1	1	....	62	....	62
& 318-326	19881	1.2	SMTKILEPF	83	17	6	11	<1	1	....	83	....	83
& 319-327	19823	1.2	MTKILEPFR	82	18	6	11	<1	1	....	82	....	82
& 320-328	19591	1.9	TKILEPFRK	75	25	6	19	<1	1	....	75	....	75
& 321-329	19485	2.1	KILEPFRKQ	73	27	6	20	<1	1	....	73	....	73
& 322-330	19759	1.8	ILEPFRKQN	79	21	4	16	<1	1	....	79	....	79
& 323-331	19788	1.8	LEPFRKQNP	79	21	4	16	<1	1	....	79	....	79
& 324-332	19401	2.6	EPFRKQNPD	62	38	16	22	<1	2	....	62	....	62
& 325-333	19069	3.0	PFRKQNPDI	58	42	12	29	1	2	....	58	....	58
& 326-334	18845	3.5	FRKQNPDIV	54	46	11	33	1	3	....	54	....	54
& 327-335	18864	3.5	RKQNPDIVI	54	46	11	33	1	3	....	54	....	54
& 328-336	18694	3.7	KQNPDIVIY	51	49	10	37	2	3	....	51	....	51
& 329-337	18873	3.2	QNPDIVIYQ	54	46	12	33	1	2	....	54	....	54
& 330-338	19089	2.7	NPDIVIYQY	58	42	13	28	<1	1	....	58	....	58
& 331-339	18671	3.5	PDIVIYQYM	43	57	15	41	<1	2	....	43	....	43
& 332-340	18658	3.5	DIVIYQYMD	43	57	15	41	<1	2	....	43	....	43
& 333-341	18913	2.6	IVIYQYMDD	54	46	19	27	<1	1	....	54	....	54
& 334-342	19399	1.8	VIYQYMDDL	65	35	21	13	<1	1	....	65	....	65
& 335-343	19681	1.6	IYQYMDDLY	67	33	23	10	<1	1	....	67	....	67
& 336-344	19668	1.6	YQYMDDLYV	66	34	22	11	<1	1	....	66	....	66
& 337-345	19670	1.6	QYMDDLYVG	67	33	23	10	<1	1	....	67	....	67
& 338-346	19627	1.6	YMDDLYVGS	67	33	23	10	<1	1	....	67	....	67
& 339-347	19551	1.7	MDDLYVGSD	67	33	23	10	<1	1	....	67	....	67
& 340-348	20029	0.8	DDLYVGSDL	90	10	4	5	<1	1	....	90	....	90
& 341-349	19982	0.9	DLYVGSDLE	89	11	4	6	<1	1	....	89	....	89
& 342-350	19955	1.0	LYVGSDLEI	88	12	4	7	<1	1	....	88	....	88
& 343-351	19703	1.6	YVGSDLEIG	74	26	13	12	<1	1	....	74	....	74

& 344-352	19640	1.8	VGSDLEIGQ	74	26	13	13	<1	1	....	74	....	74
& 345-353	19719	1.7	GSDLEIGQH	74	26	13	12	<1	1	....	74	....	74
& 346-354	19948	1.3	SDLEIGQHR	79	21	14	7	<1	1	....	79	....	79
& 347-355	19074	2.6	DLEIGQHRT	54	46	17	28	<1	2	....	54	....	54
& 348-356	19045	2.6	LEIGQHRTK	55	45	16	28	<1	2	....	55	....	55
& 349-357	18824	3.0	EIGQHRTKI	50	50	15	33	<1	2	....	50	....	50
& 350-358	18663	3.3	IGQHRTKIE	49	51	14	36	1	2	....	49	....	49
& 351-359	18628	3.4	GQHRTKIEE	48	52	14	37	1	2	....	48	....	48
& 352-360	18840	2.7	QHRTKIEEL	57	43	15	26	<1	2	....	57	....	57
& 353-361	18875	2.5	HRTKIEELR	59	41	16	24	<1	1	....	59	....	59
& 354-362	18466	3.7	RTKIEELRQ	46	54	9	43	1	3	....	46	....	46
& 355-363	18261	4.0	TKIEELRQH	45	55	9	45	2	3	....	45	....	45
& 356-364	18724	2.7	KIEELRQHL	59	41	16	25	<1	2	....	59	....	59
& 357-365	18523	3.3	IEELRQHLL	51	49	14	34	1	2	....	51	....	51
& 358-366	17904	4.2	EELRQHLLR	31	69	20	48	2	3	....	31	........K	20
& 359-367	18035	4.0	ELRQHLLRW	31	69	20	47	1	3	....	31	.......K.	20
& 360-368	18101	3.8	LRQHLLRWG	32	68	21	46	1	2	....	32	......K..	21
& 361-369	17861	4.4	RQHLLRWGF	26	74	17	56	1	3	L	6	.....K...	17
& 362-370	17476	5.3	QHLLRWGFT	20	80	14	64	2	4	.......L.	5	....K...Y	2
& 363-371	17743	4.1	HLLRWGFTT	26	74	23	50	1	2	......L..	5	...K...Y.	3
& 364-372	17912	3.9	LLRWGFTTP	26	74	23	50	1	2	.....L...	5	..K...Y..	4
& 365-373	17637	4.0	LRWGFTTPD	26	74	23	50	1	3	....L....	5	.K...Y...	3
& 366-374	17445	4.3	RWGFTTPDK	25	75	22	51	2	3	...L.....	5	K...Y....	11
& 367-375	17608	2.9	WGFTTPDKK	52	48	18	30	<1	2	..L......	9	...Y.....	18
& 368-376	17151	3.0	GFTTPDKKH	51	49	17	31	<1	2	.L.......	9	..Y......	17
& 369-377	16986	3.0	FTTPDKKHQ	51	49	17	30	1	2	L........	9	.Y.......	17
& 370-378	17045	2.7	TTPDKKHQK	60	40	16	22	1	2	....	60	Y........	16
& 371-379	17352	1.7	TPDKKHQKE	79	21	6	14	<1	1	....	79	....	79
& 372-380	17260	1.7	PDKKHQKEP	79	21	6	15	<1	1	....	79	....	79
& 373-381	16472	1.7	DKKHQKEPP	79	21	6	15	<1	1	....	79	....	79
& 374-382	16289	1.6	KKHQKEPPF	78	22	8	13	<1	1	....	78	....	78
& 375-383	16175	1.1	KHQKEPPFL	86	14	4	9	<1	1	....	86	....	86
& 376-384	15871	1.2	HQKEPPFLW	86	14	4	9	<1	1	....	86	....	86
& 377-385	15330	1.1	QKEPPFLWM	87	13	5	8	<1	1	....	87	....	87
& 378-386	14910	1.1	KEPPFLWMG	87	13	5	8	<1	1	....	87	....	87
& 379-387	14938	0.9	EPPFLWMGY	88	12	5	6	<1	1	....	88	....	88
& 380-388	13335	0.9	PPFLWMGYE	88	12	6	5	<1	1	....	88	....	88
& 381-389	13201	0.8	PFLWMGYEL	89	11	6	5	<1	1	....	89	....	89
& 382-390	12954	0.8	FLWMGYELH	89	11	6	5	<1	1	....	89	....	89
& 383-391	12885	0.8	LWMGYELHP	89	11	6	5	<1	1	....	89	....	89
# 384-392	12536	0.3	WMGYELHPD	97	3	<1	1	<1	1	....	97	....	97
# 385-393	12412	0.4	MGYELHPDK	96	4	<1	3	<1	1	....	96	....	96
# 386-394	12290	0.4	GYELHPDKW	96	4	<1	3	<1	1	....	96	....	96
# 387-395	12105	0.4	YELHPDKWT	96	4	<1	3	<1	1	....	96	....	96
# 388-396	11518	0.4	ELHPDKWTV	96	4	<1	3	<1	1	....	96	....	96
# 389-397	11426	0.5	LHPDKWTVQ	95	5	<1	4	<1	1	....	95	....	95
# 390-398	11226	0.7	HPDKWTVQP	93	7	<1	5	<1	1	....	93	....	93
# 391-399	10726	0.7	PDKWTVQPI	92	8	<1	6	<1	1	....	92	....	92
& 392-400	9830	2.6	DKWTVQPIV	59	41	9	31	<1	2	....	59	....	59
& 393-401	9662	2.6	KWTVQPIVL	59	41	9	31	<1	2	....	59	....	59
& 394-402	9559	2.5	WTVQPIVLP	60	40	9	30	<1	2	....	60	....	60
& 395-403	5063	3.0	TVQPIVLPE	55	45	10	34	2	3	....	55	....	55
& 396-404	4986	3.0	VQPIVLPEK	55	45	10	34	2	3	....	55	....	55
& 397-405	4775	3.3	QPIVLPEKD	54	46	9	35	3	5	....	54	....	54
& 398-406	4501	3.3	PIVLPEKDS	54	46	9	34	3	5	....	54	....	54
& 399-407	4456	3.2	IVLPEKDSW	54	46	9	34	3	5	....	54	....	54
& 400-408	4298	3.2	VLPEKDSWT	54	46	9	33	3	5	....	54	....	54
& 401-409	4253	1.4	LPEKDSWTV	81	19	5	12	1	2	....	81	....	81
& 402-410	4111	1.4	PEKDSWTVN	82	18	5	12	1	2	....	82	....	82
& 403-411	3881	1.4	EKDSWTVND	82	18	5	12	1	2	....	82	....	82
& 404-412	3637	1.0	KDSWTVNDI	87	13	7	5	<1	1	....	87	L	<1
& 405-413	3614	0.9	DSWTVNDIQ	88	12	7	4	<1	1	....	88	.......L.	<1
# 406-414	3601	0.5	SWTVNDIQK	95	5	1	3	<1	1	....	95	......L..	<1
# 407-415	3505	0.2	WTVNDIQKL	98	2	<1	<1	<1	1	....	98	.....L...	<1
# 408-416	3483	0.2	TVNDIQKLV	98	2	<1	<1	<1	1	....	98	....L....	<1
# 409-417	3474	0.2	VNDIQKLVG	98	2	<1	<1	<1	1	....	98	...L.....	<1
# 410-418	3458	0.2	NDIQKLVGK	98	2	<1	<1	<1	1	....	98	..L......	<1
# 411-419	3448	0.2	DIQKLVGKL	98	2	<1	<1	<1	1	....	98	.L.......	<1
# 412-420	3419	0.2	IQKLVGKLN	98	2	<1	<1	<1	1	....	98	L........	<1
# 413-421	3428	0.1	QKLVGKLNW	99	1	<1	<1	<1	1	....	99	....	99
# 414-422	3387	0.2	KLVGKLNWA	99	1	<1	<1	<1	1	....	99	....	99

# 415-423	3389	0.2	LVGKLNWAS	99	1	<1	<1	<1	1	....	99	....	99
# 416-424	3372	0.2	VGKLNWASQ	99	1	<1	<1	<1	1	....	99	....	99
# 417-425	3364	0.1	GKLNWASQI	99	1	<1	<1	<1	1	....	99	....	99
# 418-426	3357	0.1	KLNWASQIY	99	1	<1	<1	<1	1	....	99	....	99
& 419-427	3273	1.4	LNWASQIYP	52	48	42	6	<1	1	....	52	....	52
& 420-428	3264	1.4	NWASQIYPG	52	48	42	6	<1	1	....	52	....	52
& 421-429	3272	1.4	WASQIYPGI	52	48	42	6	<1	1	....	52	....	52
& 422-430	3242	1.6	ASQIYPGIK	50	50	41	8	<1	1	....	50	....	50
& 423-431	3215	1.8	SQIYPGIKV	48	52	41	10	<1	1	....	48	....	48
& 424-432	3075	2.7	QIYAGIKVK	30	70	28	41	<1	2	...P....R	19	...P.....	28
& 425-433	3046	3.0	IYAGIKVKQ	28	72	27	43	1	3	..P....R.	18	..P......	27
& 426-434	3040	3.0	YAGIKVKQL	28	72	27	43	1	3	.P....R..	18	.P.......	27
& 427-435	3012	3.0	AGIKVKQLC	28	72	27	43	1	3	P....R...	18	P........	27
& 428-436	2973	2.0	GIKVKQLCK	55	45	29	14	1	2	....R....	29	....	55
& 429-437	2962	2.2	IKVKQLCKL	55	45	28	15	1	3	...R.....	28	....	55
& 430-438	2972	2.4	KVKQLCKLL	52	48	27	20	1	3	..R......	27	....	52
& 431-439	2959	2.5	VKQLCKLLR	50	50	27	22	1	3	.R.......	27	....	50
& 432-440	2969	2.4	KQLCKLLRG	50	50	27	22	<1	2	R........	27	....	50
& 433-441	2914	2.5	QLCKLLRGT	52	48	25	23	1	3	....	52	....	52
& 434-442	2914	2.2	LCKLLRGTK	54	46	27	19	<1	2	....	54	....	54
& 435-443	2865	2.7	CKLLRGTKA	48	52	25	27	1	2	....	48	....	48
& 436-444	2847	2.7	KLLRGTKAL	47	53	25	27	1	2	....	47	....	47
& 437-445	2889	2.5	LLRGTKALT	51	49	26	22	1	2	....	51	....	51
& 438-446	2869	2.8	LRGTKALTE	46	54	22	32	1	2	....	46	....	46
& 439-447	2843	2.8	RGTKALTEV	48	52	21	30	1	3	....	48	....	48
& 440-448	2762	3.5	GTKALTEVV	26	74	24	48	1	3	I	24	....	26
& 441-449	2734	3.9	TKALTEVVP	25	75	23	51	2	4	.......I.	23	....	25
& 442-450	2807	3.0	KALTEVIPL	35	65	33	31	1	3	....	35	......V..	33
& 443-451	2809	3.1	ALTEVIPLT	34	66	32	32	1	3	....	34	.....V...	32
& 444-452	2542	4.2	LTEVVPLTE	23	77	19	56	2	5	....I....	19	........A	5
& 445-453	2503	4.3	TEVVPLTEE	24	76	18	57	2	5	...I.....	18	.......A.	6
& 446-454	2365	4.3	EVVPLTEEA	23	77	18	57	2	5	..I......	18	......A..	6
& 447-455	2160	4.0	VVPLTEEAE	25	75	19	53	2	5	.I.......	19	.....A...	6
& 448-456	1407	3.7	VPLTEEAEL	30	70	18	49	3	6	I........	18	....A....	8
& 449-457	1083	3.1	PLTEEAELE	45	55	16	36	3	6	....	45	...A.....	12
& 450-458	898	2.4	LTEEAELEL	51	49	17	30	2	4	....	51	..A......	17
& 451-459	669	2.8	TEEAELELA	49	51	18	31	3	7	....	49	.A.......	18
& 452-460	592	2.4	EEAELELAE	52	48	19	27	2	5	....	52	A........	19
# 453-461	431	0.7	EAELELAEN	91	9	6	1	2	3	....	91	....	91
# 454-462	411	0.7	AELELAENR	91	9	6	<1	2	3	....	91	....	91
# 455-463	401	0.6	ELELAENRE	91	9	6	<1	2	3	....	91	....	91
# 456-464	400	0.7	LELAENREI	90	10	6	1	3	4	....	90	....	90
# 457-465	396	0.7	ELAENREIL	90	10	6	1	3	4	....	90	....	90
& 458-466	390	1.1	LAENREILK	84	16	6	7	3	4	....	84	....	84
& 459-467	342	1.4	AENREILKE	81	19	6	10	4	6	....	81	....	81
& 460-468	337	1.2	ENREILKEP	84	16	5	6	5	7	....	84	....	84
& 461-469	331	1.2	NREILKEPV	84	16	5	5	5	8	....	84	....	84
& 462-470	312	1.3	REILKEPVH	84	16	5	6	6	8	....	84	....	84
& 463-471	310	1.3	EILKEPVHG	84	16	5	5	5	8	....	84	....	84
& 464-472	304	1.8	ILKEPVHGV	74	26	9	11	6	10	....	74	....	74
& 465-473	280	1.7	LKEPVHGVY	74	26	10	9	7	10	....	74	....	74
& 466-474	276	1.8	KEPVHGVYY	73	27	10	10	7	10	....	73	....	73
& 467-475	265	1.5	EPVHGVYYD	77	23	11	6	6	9	....	77	....	77
& 468-476	259	1.3	PVHGVYYDP	80	20	12	3	5	7	....	80	....	80
& 469-477	245	1.5	VHGVYYDPS	77	23	11	7	5	8	....	77	....	77
& 470-478	238	1.5	HGVYYDPSK	76	24	11	7	5	8	....	76	....	76
& 471-479	231	1.8	GVYYDPSKD	71	29	8	14	7	10	....	71	........E	6
& 472-480	203	1.8	VYYDPSKDL	71	29	9	13	7	10	....	71	.......E.	5
& 473-481	201	1.8	YYDPSKDLI	71	29	9	15	5	8	....	71	......E..	8
& 474-482	199	1.7	YDPSKDLIA	71	29	9	14	6	8	....	71	.....E...	8
& 475-483	198	1.7	DPSKDLIAE	71	29	9	14	6	8	....	71	....E....	8
& 476-484	195	2.3	PSKDLIAEI	62	38	8	27	4	10	....	62	...E.....	7
& 477-485	192	2.4	SKDLIAEIQ	60	40	8	27	5	10	....	60	..E......	7
& 478-486	189	2.2	KDLIAEIQK	63	37	8	22	6	10	....	63	.E.......	7
& 479-487	188	2.2	DLIAEIQKQ	63	37	9	23	6	11	....	63	E........	7
& 480-488	187	2.0	LIAEIQKQG	68	32	9	17	6	11	....	68	....	68
& 481-489	176	3.5	IAEIQKQGQ	42	58	10	38	10	19	....	42	L	1
& 482-490	168	3.3	AEIQKQGQG	45	55	10	34	11	19	....	45	.......L.	2
483-491 *	66	3.2	EIQKQGYGQ	26	74	23	41	11	23	.....Q...	23	......L..	0
484-492 *	64	3.0	IQKQGYGQW	28	72	23	39	9	20	.....Q...	23	.....L...	0
485-493 *	64	2.8	QKQGYGQWT	28	72	25	39	8	17	....Q....	25	....L....	0

486-494 *	64	2.8	KQGYGQWTY	28	72	25	39	8	17	...Q.....	25	...L.....	0
487-495 *	64	2.8	QGYGQWTYQ	28	72	25	39	8	17	..Q......	25	..L......	0
488-496 *	64	2.8	GYGQWTYQI	28	72	25	39	8	17	.Q.......	25	.L.......	0
489-497 *	64	2.7	YGQWTYQIY	28	72	27	39	6	16	Q........	27	L........	0
490-498 *	64	0.9	GQWTYQIYQ	78	22	19	0	3	5	....	78	....	78
491-499 *	64	0.2	QWTYQIYQE	97	3	2	0	2	3	....	97	....	97
492-500 *	64	0.4	WTYQIYQEP	95	5	2	0	3	5	....	95	....	95
493-501 *	64	0.5	TYQIYQEPF	94	6	2	0	5	6	....	94	....	94
494-502 *	64	0.7	YQIYQEPFK	89	11	6	0	5	6	....	89	....	89
495-503 *	64	0.7	QIYQEPFKN	89	11	6	0	5	6	....	89	....	89
496-504 *	64	0.7	IYQEPFKNL	89	11	5	0	6	8	....	89	....	89
497-505 *	64	0.9	YQEPFKNLK	88	13	5	0	8	9	....	88	....	88
498-506 *	64	0.8	QEPFKNLKT	88	13	5	0	8	9	....	88	....	88
499-507 *	63	0.9	EPFKNLKTG	87	13	5	0	8	10	....	87	....	87
500-508 *	63	0.9	PFKNLKTGK	87	13	5	0	8	10	....	87	....	87
501-509 *	62	0.6	FKNLKTGKY	90	10	5	0	5	6	....	90	....	90
502-510 *	62	0.6	KNLKTGKYA	90	10	5	0	5	6	....	90	....	90
503-511 *	62	0.9	NLKTGKYAR	82	18	13	0	5	6	....	82	....	82
504-512 *	62	2.3	LKTGKYARM	53	47	13	27	6	15	....	53	....	53
505-513 *	62	2.2	KTGKYARMR	55	45	13	27	5	13	....	55	....	55
506-514 *	62	2.6	TGKYARMRG	37	63	19	39	5	15	....	37	....	37
507-515 *	62	3.2	GKYARMRGA	27	73	19	45	8	23	....	27	....	27
508-516 *	68	3.1	KYARMRGAH	34	66	18	41	7	21	....	34	....	34
509-517 *	68	3.2	YARMRGAHT	34	66	18	40	9	22	....	34	....	34
510-518 *	68	3.3	ARMRGAHTN	32	68	18	40	10	24	....	32	....	32
511-519 *	68	3.3	RMRGAHTND	32	68	18	38	12	25	....	32	....	32
512-520 *	68	3.4	MRGAHTNDV	31	69	16	40	13	25	....	31	....	31
513-521 *	68	2.9	RGAHTNDVK	38	62	16	32	13	22	....	38	....	38
514-522 *	68	2.9	GAHTNDVKQ	38	62	16	32	13	22	....	38	....	38
515-523 *	68	2.1	AHTNDVKQL	56	44	18	18	9	15	....	56	....	56
516-524 *	68	1.6	HTNDVKQLT	65	35	18	13	4	10	....	65	....	65
517-525 *	68	1.9	TNDVKQLTE	60	40	18	16	6	13	....	60	....	60
518-526 *	68	2.0	NDVKQLTEA	59	41	18	19	4	13	....	59	....	59
519-527 *	68	1.9	DVKQLTEAV	60	40	18	19	3	12	....	60	....	60
520-528 *	68	1.8	VKQLTEAVQ	62	38	18	16	4	12	....	62	....	62
521-529 *	68	1.3	KQLTEAVQK	78	22	7	9	6	12	....	78	....	78
522-530 *	68	1.3	QLTEAVQKI	79	21	7	9	4	10	....	79	....	79
523-531 *	68	2.0	LTEAVQKIA	60	40	19	16	4	13	........T	19	........S	0
524-532 *	68	2.3	TEAVQKIAT	56	44	19	18	7	18	.......T.	19	.......S.	0
525-533 *	68	2.1	EAVQKIATE	56	44	22	15	7	15	......T..	22	......S..	0
526-534 *	68	2.4	AVQKIATES	46	54	24	24	7	15	.....T...	24	.....S...	0
527-535 *	68	2.4	VQKIATESI	49	51	24	19	9	16	....T....	24	....S....	0
528-536 *	68	2.8	QKIATESIV	34	66	24	34	9	18	...T.....	24	...S.....	0
529-537 *	68	2.7	KIATESIVI	35	65	24	34	7	16	..T......	24	..S......	0
530-538 *	68	2.7	IATESIVIW	35	65	22	35	7	16	.T.......	22	.S.......	0
531-539 *	68	2.7	ATESIVIWG	37	63	22	32	9	16	T........	22	S........	0
532-540 *	68	2.3	TESIVIWGK	56	44	16	19	9	18	....	56	........R	3
533-541 *	68	2.5	ESIVIWGKT	46	54	16	31	7	15	....	46	.......R.	1
534-542 *	67	2.4	SIVIWGKTP	46	54	16	31	6	13	....	46	......R..	1
535-543 *	67	2.0	IVIWGKTPK	55	45	16	24	4	10	....	55	.....R...	1
536-544 *	67	1.7	VIWGKTPKF	60	40	16	21	3	7	....	60	....R....	0
537-545 *	67	2.0	IWGKTPKFK	40	60	36	21	3	9	....	40	...R....R	0
538-546 *	66	2.0	WGKTPKFKL	41	59	36	20	3	9	....	41	..R....R.	0
539-547 *	66	1.9	GKTPKFKLP	41	59	38	20	2	8	....	41	.R....R..	0
540-548 *	66	1.9	KTPKFKLPI	41	59	38	20	2	8	....	41	R....R...	0
541-549 *	66	1.7	TPKFKLPIQ	41	59	38	20	2	6	....	41	....R....	38
542-550 *	66	1.1	PKFRLPIQK	55	45	44	0	2	3	...K.....	44	....	55
543-551 *	61	1.1	KFRLPIQKE	56	44	43	0	2	3	..K......	43	....	56
544-552 *	61	1.3	FRLPIQKET	54	46	41	0	5	7	.K.......	41	....	54
545-553 *	61	1.2	RLPIQKETW	56	44	41	0	3	5	K........	41	....	56
546-554 *	61	0.5	LPIQKETWE	92	8	5	0	3	5	....	92	....	92
547-555 *	60	1.2	PIQKETWET	80	20	8	3	8	12	....	80	....	80
548-556 *	53	0.9	IQKETWETW	87	13	4	0	9	11	....	87	....	87
549-557 *	53	0.9	QKETWETWW	87	13	4	0	9	11	....	87	....	87
550-558 *	53	1.6	KETWETWWT	68	32	19	4	9	13	....	68	....	68
551-559 *	53	2.2	ETWETWWTE	49	51	19	21	11	17	....	49	........D	17
552-560 *	53	2.2	TWETWWTEY	49	51	19	21	11	17	....	49	.......D.	17
553-561 *	51	2.1	WETWWTEYW	49	51	22	20	10	16	....	49	......D..	16
554-562 *	51	2.1	ETWWTEYWQ	49	51	22	20	10	16	....	49	.....D...	16
555-563 *	51	2.1	TWWTEYWQA	49	51	22	22	8	14	....	49	....D....	16
556-564 *	51	1.7	WWTEYWQAT	51	49	24	22	4	8	....	51	...D.....	24

557-565 *	51	1.7	WTEYWQATW	51	49	24	22	4	8	....	51	..D......	24
558-566 *	51	1.7	TEYWQATWI	51	49	24	22	4	8	....	51	.D.......	24
559-567 *	51	1.0	EYWQATWIP	73	27	24	0	4	6	....	73	D........	24
560-568 *	51	0.1	YWQATWIPE	98	2	2	0	0	2	....	98	....	98
561-569 *	51	0.3	WQATWIPEW	96	4	2	0	2	4	....	96	....	96
562-570 *	51	0.1	QATWIPEWE	98	2	2	0	0	2	....	98	....	98
563-571 *	51	0.3	ATWIPEWEF	96	4	2	0	2	4	....	96	....	96
564-572 *	51	0.3	TWIPEWEFV	96	4	2	0	2	4	....	96	....	96
565-573 *	51	0.4	WIPEWEFVN	94	6	2	0	4	6	....	94	....	94
566-574 *	51	0.7	IPEWEFVNT	90	10	2	0	8	10	....	90	....	90
567-575 *	51	0.7	PEWEFVNTP	90	10	2	0	8	10	....	90	....	90
568-576 *	51	0.7	EWEFVNTPP	90	10	2	0	8	10	....	90	....	90
569-577 *	51	0.7	WEFVNTPPL	90	10	2	0	8	10	....	90	....	90
570-578 *	51	0.6	EFVNTPPLV	92	8	2	0	6	8	....	92	....	92
571-579 *	51	0.6	FVNTPPLVK	92	8	2	0	6	8	....	92	....	92
572-580 *	51	0.4	VNTPPLVKL	94	6	2	0	4	6	....	94	....	94
573-581 *	51	0.4	NTPPLVKLW	94	6	2	0	4	6	....	94	....	94
574-582 *	51	0.4	TPPLVKLWY	94	6	2	0	4	6	....	94	....	94
575-583 *	51	0.1	PPLVKLWYQ	98	2	2	0	0	2	....	98	....	98
576-584 *	51	0.1	PLVKLWYQL	98	2	2	0	0	2	....	98	....	98
577-585 *	51	0.1	LVKLWYQLE	98	2	2	0	0	2	....	98	....	98
578-586 *	51	0.3	VKLWYQLEK	96	4	2	0	2	4	....	96	....	96
579-587 *	51	0.9	KLWYQLEKE	78	22	18	0	4	6	....	78	....	78
580-588 *	51	1.0	LWYQLEKEP	78	22	18	0	4	6	....	78	....	78
581-589 *	51	1.0	WYQLEKEPI	78	22	18	0	4	6	....	78	....	78
582-590 *	51	1.9	YQLEKEPIV	55	45	18	22	6	12	....	55	........A	6
583-591 *	51	1.9	QLEKEPIVG	57	43	18	20	6	12	....	57	.......A.	4
584-592 *	51	2.0	LEKEPIVGA	55	45	18	20	8	14	....	55	......A..	4
585-593 *	51	2.0	EKEPIVGAE	55	45	18	20	8	14	....	55	.....A...	4
586-594 *	51	2.0	KEPIVGAET	55	45	18	20	8	14	....	55	....A....	4
587-595 *	51	2.0	EPIVGAETF	53	47	18	24	6	14	....	53	...A.....	4
588-596 *	51	1.9	PIVGAETFY	53	47	20	24	4	12	....	53	..A......	4
589-597 *	51	1.9	IVGAETFYV	53	47	20	24	4	12	....	53	.A.......	4
590-598 *	51	1.9	VGAETFYVD	53	47	20	24	4	12	....	53	A........	4
591-599 *	51	1.0	GAETFYVDG	75	25	20	4	2	6	....	75	....	75
592-600 *	51	1.0	AETFYVDGA	75	25	20	4	2	6	....	75	....	75
593-601 *	51	1.1	ETFYVDGAA	75	25	18	4	4	8	....	75	....	75
594-602 *	51	0.9	TFYVDGAAN	76	24	18	4	2	6	....	76	....	76
595-603 *	51	0.9	FYVDGAANR	76	24	18	4	2	6	....	76	....	76
596-604 *	89	1.0	YVDGAANRE	83	17	10	4	2	6	....	83	....	83
597-605 *	89	1.5	VDGAANRET	72	28	11	15	2	8	....	72	....	72
598-606 *	89	1.9	DGAANRETK	62	38	11	25	2	9	....	62	....	62
599-607 *	89	2.9	GAANRETKL	46	54	10	36	8	18	....	46	....	46
600-608 *	89	2.9	AANRETKLG	46	54	10	36	8	18	....	46	....	46
601-609 *	89	3.1	ANRETKLGK	43	57	10	39	8	19	....	43	........R	3
602-610 *	89	2.7	NRETKLGKA	51	49	10	33	7	17	....	51	.......R.	3
603-611 *	89	2.6	RETKLGKAG	52	48	10	33	6	16	....	52	......R..	3
604-612 *	89	2.6	ETKLGKAGY	52	48	10	33	6	16	....	52	.....R...	3
605-613 *	89	2.8	TKLGKAGYV	48	52	10	35	7	17	....	48	....R....	3
606-614 *	89	2.4	KLGKAGYVT	51	49	19	25	6	13	....	51	...R.....	3
607-615 *	89	2.8	LGKAGYVTD	36	64	19	37	8	16	........N	19	..R......	2
608-616 *	89	2.3	GKAGYVTDR	46	54	19	29	6	11	.......N.	19	.R.......	2
609-617 *	89	2.3	KAGYVTDRG	46	54	19	29	6	11	......N..	19	R........	2
610-618 *	89	2.2	AGYVTDRGR	47	53	19	27	7	11	.....N...	19	....	47
611-619 *	89	2.2	GYVTDRGRQ	47	53	19	27	7	11	....N....	19	....	47
612-620 *	89	2.2	YVTDRGRQK	47	53	19	27	7	11	...N.....	19	....	47
613-621 *	89	2.6	VTDRGRQKV	42	58	19	28	11	16	..N......	19	....	42
614-622 *	88	2.5	TDRGRQKVV	45	55	19	24	11	16	.N.......	19	I	2
615-623 *	88	3.0	DRGRQKVVS	41	59	17	30	13	20	N.......T	2	.......I.	2
616-624 *	87	2.8	RGRQKVVSL	53	47	11	18	17	25	.......T.	2	......I..	1
617-625 *	87	2.7	GRQKVVSLT	56	44	9	17	17	24	......T..	2	.....I...	1
618-626 *	87	3.0	RQKVVSLTD	52	48	9	21	18	26	.....T...	2	....I....	1
619-627 *	87	3.0	QKVVSLTDT	51	49	9	23	17	26	....T....	2	...I.....	2
620-628 *	87	3.0	KVVSLTDTT	51	49	9	23	17	26	...T.....	2	..I......	2
621-629 *	87	3.0	VVSLTDTTN	51	49	9	24	16	25	..T......	2	.I.......	2
622-630 *	87	2.9	VSLTDTTNQ	51	49	9	25	15	24	.T.......	2	I........	2
623-631 *	87	2.8	SLTDTTNQK	52	48	9	23	16	23	T........	2	....	52
624-632 *	87	2.1	LTDTTNQKT	62	38	17	10	10	16	....	62	....	62
625-633 *	87	1.1	TDTTNQKTE	84	16	3	7	6	10	....	84	....	84
626-634 *	87	0.7	DTTNQKTEL	90	10	5	3	2	5	....	90	....	90
627-635 *	87	0.3	TTNQKTELQ	97	3	1	0	2	3	....	97	....	97

628-636 *	87	0.2	TNQKTELQA	98	2	1	0	1	2	....	98	....	98
629-637 *	87	0.2	NQKTELQAI	98	2	1	0	1	2	....	98	....	98
630-638 *	87	1.9	QKTELQAIY	48	52	32	15	5	8	....	48	........H	32
631-639 *	87	1.9	KTELQAIYL	48	52	32	15	5	8	....	48	.......H.	32
632-640 *	87	1.9	TELQAIYLA	49	51	31	15	5	8	....	49	......H..	31
633-641 *	87	2.0	ELQAIYLAL	48	52	31	15	6	9	....	48	.....H...	31
634-642 *	86	2.0	LQAIYLALQ	49	51	30	15	6	9	....	49	....H....	30
635-643 *	86	2.1	QAIYLALQD	49	51	28	17	6	10	....	49	...H.....	28
636-644 *	86	2.0	AIYLALQDS	49	51	29	17	5	9	....	49	..H......	29
637-645 *	85	2.1	IYLALQDSG	47	53	29	18	6	12	....	47	.H.......	29
638-646 *	85	2.7	YLALQDSGL	42	58	22	24	12	18	....	42	H.......S	7
639-647 *	85	1.3	LALQDSGLE	78	22	12	2	8	11	....	78	.......S.	12
640-648 *	85	1.5	ALQDSGLEV	74	26	12	6	8	12	....	74	......S..	12
641-649 *	85	1.5	LQDSGLEVN	74	26	12	7	7	11	....	74	.....S...	12
642-650 *	85	1.4	QDSGLEVNI	75	25	12	7	6	9	....	75	....S....	12
643-651 *	84	1.4	DSGLEVNIV	76	24	11	7	6	10	....	76	...S.....	11
644-652 *	84	1.3	SGLEVNIVT	79	21	10	4	8	11	....	79	..S......	10
645-653 *	84	1.3	GLEVNIVTD	79	21	10	4	8	11	....	79	.S.......	10
646-654 *	84	1.2	LEVNIVTDS	80	20	11	4	6	8	....	80	S........	11
647-655 *	84	0.6	EVNIVTDSQ	92	8	4	0	5	6	....	92	....	92
648-656 *	84	0.5	VNIVTDSQY	93	7	4	0	4	5	....	93	....	93
649-657 *	82	0.4	NIVTDSQYA	95	5	1	0	4	5	....	95	....	95
650-658 *	80	0.3	IVTDSQYAL	96	4	1	0	3	4	....	96	....	96
651-659 *	80	0.2	VTDSQYALG	98	3	1	0	1	3	....	98	....	98
652-660 *	80	0.2	TDSQYALGI	98	3	1	0	1	3	....	98	....	98
653-661 *	80	0.1	DSQYALGII	99	1	1	0	0	1	....	99	....	99
654-662 *	80	0.1	SQYALGIIQ	99	1	1	0	0	1	....	99	....	99
655-663 *	80	0.2	QYALGIIQA	98	3	1	0	1	3	....	98	....	98
656-664 *	80	0.3	YALGIIQAQ	96	4	1	0	3	4	....	96	....	96
657-665 *	79	0.3	ALGIIQAQP	96	4	1	0	3	4	....	96	....	96
658-666 *	79	0.4	LGIIQAQPD	95	5	3	0	3	4	....	95	....	95
659-667 *	39	1.0	GIIQAQPDK	82	18	8	5	5	10	........Q	8	....	82
660-668 *	39	1.0	IIQAQPDKS	82	18	8	5	5	10	.......Q.	8	....	82
661-669 *	39	1.0	IQAQPDKSE	82	18	8	5	5	10	......Q..	8	....	82
662-670 *	39	1.0	QAQPDKSES	82	18	8	5	5	10	.....Q...	8	....	82
663-671 *	39	1.0	AQPDKSESE	82	18	8	5	5	10	....Q....	8	....	82
664-672 *	39	1.7	QPDKSESEL	67	33	13	18	3	13	...Q.....	8	....	67
665-673 *	39	1.6	PDKSESELV	67	33	13	21	0	10	..Q......	10	....	67
666-674 *	39	2.2	DKSESELVS	51	49	15	28	5	15	.Q......N	10	........N	15
667-675 *	39	2.3	KSESELVSQ	49	51	15	28	8	18	Q......N.	10	.......N.	15
668-676 *	39	1.9	SESELVSQI	49	51	28	23	0	10	......N..	28	......N..	28
669-677 *	39	1.9	ESELVSQII	49	51	28	23	0	10	.....N...	28	.....N...	28
670-678 *	39	1.9	SELVSQIIE	49	51	28	23	0	10	....N....	28	....N....	28
671-679 *	39	2.4	ELVSQIIEQ	41	59	28	23	8	21	...N.....	28	...N....L	0
672-680 *	39	2.4	LVSQIIEQL	41	59	28	23	8	21	..N......	28	..N....L.	0
673-681 *	39	1.8	VSQIIEQLI	54	46	28	10	8	13	.N.......	28	.N....L..	0
674-682 *	39	2.3	SQIIEQLIK	33	67	28	31	8	15	N........	28	N....L..N	0
675-683 *	39	1.7	QIIEQLIKK	62	38	21	10	8	13	....	62	....L..N.	0
676-684 *	39	1.8	IIEQLIKKE	59	41	21	10	10	15	....	59	...L..N..	0
677-685 *	39	2.1	IEQLIKKEK	54	46	21	15	10	18	....	54	..L..N...	0
678-686 *	39	2.2	EQLIKKEKV	54	46	18	15	13	21	....	54	.L..N...I	0
679-687 *	39	2.2	QLIKKEKVY	54	46	18	15	13	21	....	54	L..N...I.	0
680-688 *	39	1.8	LIKKEKVYL	64	36	18	5	13	18	....	64	..N...I..	3
681-689 *	39	1.9	IKKEKVYLA	62	38	18	5	15	21	....	62	.N...I...	3
682-690 *	39	1.9	KKEKVYLAW	62	38	18	5	15	21	....	62	N...I....	3
683-691 *	39	1.2	KEKVYLAWV	79	21	8	5	8	13	....	79	...I.....	5
684-692 *	39	1.2	EKVYLAWVP	79	21	8	5	8	13	....	79	..I......	5
685-693 *	39	1.1	KVYLAWVPA	79	21	10	5	5	10	....	79	.I.......	5
686-694 *	39	0.6	VYLAWVPAH	90	10	5	0	5	8	....	90	I........	5
687-695 *	39	0.3	YLAWVPAHK	95	5	3	0	3	5	....	95	....	95
688-696 *	39	0.3	LAWVPAHKG	95	5	3	0	3	5	....	95	....	95
689-697 *	46	0.3	AWVPAHKGI	96	4	2	0	2	4	....	96	....	96
690-698 *	46	0.2	WVPAHKGIG	98	2	2	0	0	2	....	98	....	98
691-699 *	46	0.2	VPAHKGIGG	98	2	2	0	0	2	....	98	....	98
692-700 *	46	0.2	PAHKGIGGN	98	2	2	0	0	2	....	98	....	98
693-701 *	48	0.4	AHKGIGGNE	92	8	4	0	4	6	....	92	....	92
694-702 *	48	0.7	HKGIGGNEQ	88	13	4	0	8	10	....	88	....	88
695-703 *	48	1.0	KGIGGNEQV	83	17	6	4	6	10	....	83	....	83
696-704 *	48	1.0	GIGGNEQVD	83	17	6	4	6	10	....	83	....	83
697-705 *	53	1.2	IGGNEQVDK	81	19	6	6	8	11	....	81	....	81
698-706 *	55	1.1	GGNEQVDKL	82	18	5	5	7	11	....	82	....	82

699-707 *	55	1.1	GNEQVDKLV	82	18	5	5	7	11	....	82	....	82
700-708 *	55	1.1	NEQVDKLVS	82	18	5	5	7	11	....	82	....	82
701-709 *	55	2.8	EQVDKLVSA	44	56	18	18	20	25	....	44	....	44
702-710 *	55	2.6	QVDKLVSAG	45	55	18	18	18	24	....	45	....	45
703-711 *	55	2.5	VDKLVSAGI	45	55	18	25	11	18	....	45	....	45
704-712 *	55	2.2	DKLVSAGIR	47	53	20	27	5	13	....	47	....	47
705-713 *	55	2.4	KLVSAGIRK	42	58	20	33	5	15	....	42	....	42
706-714 *	55	2.4	LVSAGIRKV	42	58	20	33	5	15	....	42	....	42
707-715 *	55	2.4	VSAGIRKVL	42	58	20	33	5	15	....	42	....	42
708-716 *	17	1.9	SAGIRKVLF	47	53	24	18	12	24	....	47	....	47
709-717 *	32	1.9	AGIRKVLFL	47	53	25	25	3	13	....	47	....	47
710-718 *	33	0.0	GIRKVLFLD	100	0	0	0	0	0	....	100	....	100
711-719 *	33	0.2	IRKVLFLDG	97	3	3	0	0	3	....	97	....	97
712-720 *	35	0.4	RKVLFLDGI	91	9	3	0	6	9	....	91	....	91
713-721 *	34	0.8	KVLFLDGID	85	15	3	0	12	15	....	85	....	85
714-722 *	33	0.6	VLFLDGIDK	88	12	3	0	9	12	....	88	........R	0
715-723 *	34	0.6	LFLDGIDKA	91	9	3	0	6	9	....	91	.......R.	0
& 716-724	523	1.0	FLDGIDKAQ	86	14	4	9	2	3	....	86	......R..	1
& 717-725	517	1.8	LDGIDKAQE	70	30	13	16	2	4	........D	13	.....R...	1
& 718-726	516	2.5	DGIDKAQEE	48	52	21	29	2	6	.......D.	12	....R...D	<1
& 719-727	516	2.5	GIDKAQEEH	48	52	21	28	2	5	......D..	12	...R...D.	<1
& 720-728	526	2.6	IDKAQEEHE	49	51	19	30	2	6	.....D...	12	..R...D..	<1
& 721-729	525	2.8	DKAQEEHEK	47	53	18	31	3	7	....D....	12	.R...D...	<1
& 722-730	529	2.5	KAQEEHEKY	49	51	18	31	2	5	...D.....	15	R...D....	<1
& 723-731	532	2.1	AQEEHEKYH	52	48	23	24	1	4	..D......	15	...D.....	23
& 724-732	529	2.8	QEEHEKYHS	38	62	21	40	2	5	.D.......	14	..D......	21
& 725-733	528	2.8	EEHEKYHSN	38	62	21	40	2	5	D........	14	.D.......	21
& 726-734	528	2.1	EHEKYHSNW	54	46	22	22	2	4	....	54	D........	22
& 727-735	535	1.6	HEKYHSNWR	73	27	13	13	2	3	....	73	....	73
& 728-736	533	1.8	EKYHSNWRA	69	31	13	15	2	4	....	69	....	69
& 729-737	535	1.9	KYHSNWRAM	66	34	13	19	2	4	....	66	....	66
& 730-738	532	1.8	YHSNWRAMA	69	31	13	16	3	5	....	69	....	69
& 731-739	522	2.4	HSNWRAMAS	62	38	12	23	3	7	....	62	........N	4
& 732-740	525	2.6	SNWRAMASD	60	40	12	25	4	8	....	60	.......N.	3
& 733-741	532	1.9	NWRAMASDF	76	24	3	18	3	6	....	76	......N..	2
& 734-742	538	2.1	WRAMASDFN	71	29	4	22	3	7	....	71	.....N...	2
& 735-743	538	2.4	RAMASDFNL	67	33	4	25	4	7	....	67	....N....	2
& 736-744	543	2.2	AMASDFNLP	71	29	4	23	3	7	....	71	...N.....	2
& 737-745	543	2.1	MASDFNLPP	71	29	4	22	3	6	....	71	..N......	2
& 738-746	534	2.7	ASDFNLPPV	57	43	14	25	3	8	....	57	.N......I	1
& 739-747	541	2.8	SDFNLPPVV	57	43	13	26	4	8	....	57	N......I.	1
& 740-748	550	2.4	DFNLPPVVA	59	41	15	24	2	5	....	59	......I..	15
& 741-749	550	2.2	FNLPPVVAK	61	39	17	21	2	4	....	61	.....I...	17
& 742-750	548	2.1	NLPPVVAKE	61	39	17	20	2	4	....	61	....I....	17
& 743-751	551	1.7	LPPVVAKEI	65	35	20	14	<1	3	....	65	...I.....	20
& 744-752	548	1.7	PPVVAKEIV	65	35	19	14	<1	3	....	65	..I......	19
& 745-753	548	1.7	PVVAKEIVA	65	35	19	14	<1	3	....	65	.I.......	19
& 746-754	536	2.2	VVAKEIVAS	60	40	18	21	1	4	....	60	I........	18
& 747-755	546	1.5	VAKEIVASC	78	22	5	16	<1	3	....	78	....	78
& 748-756	545	1.4	AKEIVASCD	79	21	6	14	1	3	....	79	....	79
& 749-757	545	1.4	KEIVASCDK	79	21	6	14	1	3	....	79	....	79
& 750-758	545	1.3	EIVASCDKC	80	20	6	13	1	3	....	80	....	80
& 751-759	548	1.3	IVASCDKCQ	80	20	6	13	1	3	....	80	....	80
& 752-760	547	1.8	VASCDKCQL	76	24	4	18	2	5	....	76	V	2
& 753-761	549	1.7	ASCDKCQLK	76	24	4	18	2	5	....	76	.......V.	2
& 754-762	547	1.8	SCDKCQLKG	76	24	4	17	2	5	....	76	......V..	2
& 755-763	553	1.4	CDKCQLKGE	81	19	4	13	2	4	....	81	.....V...	3
& 756-764	553	1.4	DKCQLKGEA	81	19	4	13	2	4	....	81	....V....	3
& 757-765	539	1.9	KCQLKGEAM	70	30	11	16	3	5	....	70	...V.....	2
& 758-766	543	2.0	CQLKGEAMH	69	31	11	17	3	6	....	69	..V......	2
& 759-767	542	2.0	QLKGEAMHG	69	31	11	17	3	6	....	69	.V.......	2
& 760-768	543	1.9	LKGEAMHGQ	69	31	11	17	2	5	....	69	V........	2
& 761-769	544	1.4	KGEAMHGQV	75	25	15	8	2	3	....	75	....	75
& 762-770	545	1.3	GEAMHGQVD	75	25	15	8	1	3	....	75	....	75
& 763-771	545	1.4	EAMHGQVDC	74	26	15	9	2	4	....	74	....	74
& 764-772	544	1.3	AMHGQVDCS	75	25	17	5	3	4	....	75	....	75
& 765-773	543	1.3	MHGQVDCSP	76	24	17	5	3	4	....	76	....	76
# 766-774	559	0.6	HGQVDCSPG	93	7	4	3	1	2	....	93	....	93
# 767-775	558	0.6	GQVDCSPGI	93	7	3	3	1	3	....	93	....	93
# 768-776	559	0.6	QVDCSPGIW	92	8	3	3	1	3	....	92	....	92
# 769-777	557	0.6	VDCSPGIWQ	92	8	3	3	1	3	....	92	....	92

# 770-778	557	0.7	DCSPGIWQL	93	7	2	4	2	3	....	93	....	93
# 771-779	557	0.6	CSPGIWQLD	93	7	2	4	1	3	....	93	....	93
# 772-780	557	0.6	SPGIWQLDC	93	7	2	4	1	3	....	93	....	93
# 773-781	559	0.5	PGIWQLDCT	94	6	2	3	1	2	....	94	....	94
# 774-782	559	0.5	GIWQLDCTH	94	6	2	3	1	2	....	94	....	94
# 775-783	558	0.6	IWQLDCTHL	94	6	1	3	2	3	....	94	....	94
# 776-784	557	0.5	WQLDCTHLE	94	6	3	2	1	2	....	94	....	94
# 777-785	557	0.6	QLDCTHLEG	94	6	3	2	2	3	....	94	....	94
# 778-786	558	0.6	LDCTHLEGK	93	7	3	3	2	3	....	93	....	93
& 779-787	545	1.4	DCTHLEGKV	50	50	45	3	1	2	....	50	I	45
& 780-788	545	1.4	CTHLEGKVI	50	50	45	3	1	3	....	50	.......I.	45
& 781-789	543	1.7	THLEGKVIL	48	52	42	9	2	4	....	48	......I..	42
& 782-790	543	1.7	HLEGKVILV	48	52	42	9	2	4	....	48	.....I...	42
& 783-791	544	1.8	LEGKVILVA	48	52	42	9	2	4	....	48	....I....	42
& 784-792	546	1.7	EGKVILVAV	48	52	41	9	2	3	....	48	...I.....	41
& 785-793	548	1.7	GKVILVAVH	48	52	41	9	2	3	....	48	..I......	41
& 786-794	549	1.7	KVILVAVHV	48	52	41	9	2	3	....	48	.I.......	41
& 787-795	549	1.7	VILVAVHVA	48	52	41	9	2	3	....	48	I........	41
& 788-796	564	0.7	ILVAVHVAS	90	10	7	2	1	2	....	90	....	90
& 789-797	560	0.7	LVAVHVASG	90	10	7	2	2	3	....	90	....	90
# 790-798	560	0.6	VAVHVASGY	92	8	5	1	1	2	....	92	....	92
& 791-799	556	0.9	AVHVASGYI	87	13	5	6	1	3	....	87	....	87
& 792-800	556	0.9	VHVASGYIE	87	13	5	6	1	3	....	87	....	87
& 793-801	557	0.9	HVASGYIEA	88	12	5	6	2	3	....	88	....	88
& 794-802	557	0.9	VASGYIEAE	87	13	5	6	2	3	....	87	....	87
& 795-803	554	0.9	ASGYIEAEV	88	12	5	6	2	3	....	88	....	88
& 796-804	554	0.9	SGYIEAEVI	88	12	5	5	2	3	....	88	....	88
& 797-805	554	0.9	GYIEAEVIP	87	13	5	6	2	3	....	87	....	87
& 798-806	557	1.0	YIEAEVIPA	86	14	4	8	2	4	....	86	....	86
# 799-807	560	0.8	IEAEVIPAE	90	10	2	6	2	4	....	90	....	90
# 800-808	564	0.5	EAEVIPAET	94	6	1	2	2	3	....	94	....	94
# 801-809	564	0.5	AEVIPAETG	94	6	1	3	2	3	....	94	....	94
# 802-810	564	0.5	EVIPAETGQ	94	6	1	2	2	3	....	94	....	94
# 803-811	563	0.5	VIPAETGQE	94	6	<1	2	2	3	....	94	....	94
# 804-812	584	0.7	IPAETGQET	93	7	<1	3	3	4	....	93	....	93
# 805-813	585	0.6	PAETGQETA	93	7	<1	3	3	4	....	93	....	93
# 806-814	584	0.5	AETGQETAY	94	6	<1	3	2	4	....	94	....	94
# 807-815	584	0.5	ETGQETAYF	94	6	2	2	2	3	....	94	....	94
& 808-816	571	1.4	TGQETAYFL	58	42	37	4	2	3	....	58	I	37
& 809-817	571	1.3	GQETAYFLL	58	42	37	3	2	3	....	58	.......I.	37
& 810-818	568	1.4	QETAYFLLK	58	42	37	4	2	3	....	58	......I..	37
& 811-819	567	1.3	ETAYFLLKL	58	42	37	4	1	2	....	58	.....I...	37
& 812-820	568	1.3	TAYFLLKLA	58	42	38	4	<1	2	....	58	....I....	38
& 813-821	569	1.5	AYFLLKLAG	51	49	38	10	<1	2	....	51	...I.....	38
& 814-822	570	1.6	YFLLKLAGR	50	50	38	10	1	2	....	50	..I......	38
& 815-823	571	1.5	FLLKLAGRW	51	49	39	10	<1	2	....	51	.I.......	39
& 816-824	571	1.4	LLKLAGRWP	50	50	40	8	1	2	....	50	I........	40
# 817-825	583	0.6	LKLAGRWPV	91	9	7	2	<1	2	....	91	....	91
& 818-826	575	1.1	KLAGRWPVK	83	17	7	10	1	2	....	83	....	83
& 819-827	572	1.9	LAGRWPVKT	70	30	7	20	2	5	....	70	....	70
& 820-828	569	2.4	AGRWPVKTI	64	36	5	26	4	7	....	64	V	5
& 821-829	568	2.4	GRWPVKTIH	64	36	5	26	4	7	....	64	.......V.	5
& 822-830	566	2.1	RWPVKTIHT	67	33	6	23	4	6	....	67	......V..	5
& 823-831	566	2.1	WPVKTIHTD	67	33	6	23	3	5	....	67	.....V...	5
& 824-832	566	2.1	PVKTIHTDN	67	33	6	23	3	5	....	67	....V....	5
& 825-833	566	2.1	VKTIHTDNG	68	32	6	23	3	5	....	68	...V.....	5
& 826-834	562	3.3	KTIHTDNGS	49	51	7	38	6	9	....	49	..V.....P	<1
& 827-835	568	2.9	TIHTDNGSN	52	48	8	37	3	6	....	52	.V.....P.	<1
& 828-836	572	2.0	IHTDNGSNF	61	39	12	25	2	3	....	61	V.....P..	<1
& 829-837	572	2.0	HTDNGSNFT	64	36	9	26	<1	3	....	64	.....P..I	6
& 830-838	570	2.0	TDNGSNFTS	64	36	9	27	<1	3	........G	<1	....P..I.	6
& 831-839	565	3.5	DNGSNFTST	37	63	16	45	2	6	.......GA	<1	...P..I..	4
& 832-840	560	4.4	NGSNFTSTT	30	70	12	54	4	12	......GA.	<1	..P..I..A	1
& 833-841	558	4.5	GSNFTSTTV	30	70	12	53	6	14	.....GA..	<1	.P..I..A.	1
& 834-842	558	4.5	SNFTSTTVK	30	70	11	53	6	14	....GA..R	<1	P..I..A..	1
& 835-843	564	3.8	NFTSTTVKA	35	65	12	49	4	9	...GA..R.	<1	..I..A...	3
& 836-844	564	3.8	FTSTTVKAA	35	65	12	49	4	9	..GA..R..	<1	.I..A....	3
& 837-845	565	3.8	TSTTVKAAC	35	65	12	49	4	9	.GA..R...	<1	I..A.....	3
& 838-846	571	3.3	STTVKAACW	39	61	14	43	3	7	GA..R....	<1	..A......	9
& 839-847	574	3.3	TTVKAACWW	39	61	14	44	3	7	A..R.....	<1	.A.......	9
& 840-848	580	2.0	TVKAACWWA	63	37	20	14	2	4	..R......	<1	A........	20

& 841-849	587	1.0	VKAACWWAG	87	13	3	7	3	4	.R.......	<1	....	87
& 842-850	588	1.1	KAACWWAGI	84	16	6	8	2	3	R........	<1	....	84
& 843-851	585	1.4	AACWWAGIK	79	21	6	12	3	5	....	79	....	79
& 844-852	585	1.3	ACWWAGIKQ	82	18	6	9	3	4	....	82	....	82
& 845-853	585	1.3	CWWAGIKQE	82	18	6	9	3	5	....	82	....	82
& 846-854	585	1.3	WWAGIKQEF	82	18	6	9	3	5	....	82	....	82
& 847-855	584	1.3	WAGIKQEFG	82	18	6	9	3	5	....	82	....	82
& 848-856	582	1.3	AGIKQEFGI	82	18	6	9	3	5	....	82	....	82
& 849-857	582	1.3	GIKQEFGIP	82	18	6	9	2	4	....	82	....	82
& 850-858	582	1.2	IKQEFGIPY	82	18	6	10	2	3	....	82	....	82
& 851-859	580	0.8	KQEFGIPYN	89	11	4	6	1	3	....	89	....	89
# 852-860	583	0.5	QEFGIPYNP	94	6	4	1	1	2	....	94	....	94
# 853-861	582	0.2	EFGIPYNPQ	98	2	<1	<1	1	2	....	98	....	98
# 854-862	582	0.2	FGIPYNPQS	98	2	<1	<1	1	1	....	98	....	98
# 855-863	582	0.2	GIPYNPQSQ	98	2	<1	0	2	2	....	98	....	98
# 856-864	583	0.2	IPYNPQSQG	98	2	<1	0	1	2	....	98	....	98
# 857-865	584	0.2	PYNPQSQGV	98	2	<1	0	1	1	....	98	....	98
# 858-866	578	0.4	YNPQSQGVV	94	6	4	<1	1	2	....	94	....	94
# 859-867	579	0.5	NPQSQGVVE	93	7	4	1	1	2	....	93	....	93
& 860-868	581	0.8	PQSQGVVES	89	11	4	5	1	2	....	89	....	89
& 861-869	579	1.2	QSQGVVESM	82	18	4	12	2	3	....	82	....	82
& 862-870	579	1.2	SQGVVESMN	82	18	4	12	2	3	....	82	....	82
& 863-871	579	1.5	QGVVESMNK	76	24	6	16	2	3	....	76	....	76
& 864-872	579	1.6	GVVESMNKE	75	25	6	17	2	4	....	75	....	75
& 865-873	580	1.6	VVESMNKEL	75	25	6	17	2	4	....	75	....	75
& 866-874	581	1.6	VESMNKELK	76	24	6	16	2	4	....	76	....	76
& 867-875	587	1.5	ESMNKELKK	78	22	6	13	3	5	....	78	....	78
& 868-876	585	1.5	SMNKELKKI	78	22	6	13	3	4	....	78	....	78
& 869-877	584	1.5	MNKELKKII	78	22	5	14	3	4	....	78	....	78
& 870-878	584	1.5	NKELKKIIG	80	20	5	11	3	5	....	80	........E	3
& 871-879	584	1.5	KELKKIIGQ	80	20	5	11	3	5	....	80	.......E.	3
& 872-880	583	1.4	ELKKIIGQV	81	19	4	11	3	5	....	81	......E..	3
& 873-881	583	1.4	LKKIIGQVR	81	19	4	11	3	5	....	81	.....E...	3
& 874-882	582	1.5	KKIIGQVRD	79	21	4	12	4	6	....	79	....E....	3
& 875-883	582	1.5	KIIGQVRDQ	79	21	4	12	4	6	....	79	...E.....	3
& 876-884	579	1.3	IIGQVRDQA	81	19	4	11	3	4	....	81	..E......	4
& 877-885	578	1.3	IGQVRDQAE	81	19	4	11	3	4	....	81	.E.......	4
& 878-886	578	1.3	GQVRDQAEH	81	19	4	11	3	4	....	81	E........	4
# 879-887	578	0.7	QVRDQAEHL	90	10	5	3	2	3	....	90	....	90
& 880-888	577	0.9	VRDQAEHLK	87	13	5	6	2	3	....	87	....	87
# 881-889	577	0.6	RDQAEHLKT	92	8	4	3	2	3	....	92	....	92
# 882-890	577	0.7	DQAEHLKTA	92	8	4	3	2	3	....	92	....	92
# 883-891	578	0.6	QAEHLKTAV	93	7	3	2	2	3	....	93	....	93
# 884-892	577	0.6	AEHLKTAVQ	93	7	3	2	2	3	....	93	....	93
# 885-893	579	0.6	EHLKTAVQM	93	7	3	2	2	3	....	93	....	93
# 886-894	580	0.6	HLKTAVQMA	93	7	3	2	2	3	....	93	....	93
# 887-895	582	0.5	LKTAVQMAV	94	6	3	<1	2	2	....	94	....	94
# 888-896	582	0.7	KTAVQMAVF	91	9	4	4	2	2	....	91	....	91
# 889-897	582	0.7	TAVQMAVFI	90	10	4	4	2	2	....	90	....	90
& 890-898	585	0.7	AVQMAVFIH	90	10	4	5	2	2	....	90	....	90
# 891-899	566	0.7	VQMAVFIHN	90	10	4	4	1	2	....	90	....	90
# 892-900	565	0.6	QMAVFIHNF	91	9	4	5	<1	2	....	91	....	91
# 893-901	566	0.6	MAVFIHNFK	91	9	4	5	<1	2	....	91	....	91
& 894-902	566	0.7	AVFIHNFKR	90	10	4	5	1	2	....	90	....	90
& 895-903	561	0.9	VFIHNFKRK	88	12	4	8	<1	2	....	88	....	88
& 896-904	561	0.9	FIHNFKRKG	88	12	4	8	1	3	....	88	....	88
# 897-905	562	0.7	IHNFKRKGG	91	9	4	5	<1	2	....	91	....	91
# 898-906	559	0.4	HNFKRKGGI	95	5	2	3	<1	2	....	95	....	95
# 899-907	557	0.4	NFKRKGGIG	96	4	2	2	<1	1	....	96	....	96
& 900-908	557	0.9	FKRKGGIGG	88	12	4	7	<1	3	....	88	....	88
& 901-909	557	0.8	KRKGGIGGY	89	11	4	6	<1	2	....	89	....	89
& 902-910	557	1.2	RKGGIGGYS	83	17	6	10	1	3	....	83	....	83
& 903-911	558	1.1	KGGIGGYSA	84	16	6	10	<1	3	....	84	....	84
& 904-912	562	1.0	GGIGGYSAG	85	15	6	8	1	2	....	85	....	85
& 905-913	560	1.0	GIGGYSAGE	85	15	6	8	1	3	....	85	....	85
& 906-914	560	1.0	IGGYSAGER	85	15	6	8	1	3	....	85	....	85
& 907-915	559	1.1	GGYSAGERI	85	15	4	10	1	3	....	85	....	85
& 908-916	545	2.0	GYSAGERIV	51	49	33	15	1	4	....	51	....	51
& 909-917	542	1.6	YSAGERIVD	55	45	36	8	1	3	....	55	....	55
& 910-918	542	1.9	SAGERIVDI	52	48	32	15	1	3	....	52	....	52
& 911-919	541	1.8	AGERIVDII	54	46	32	13	1	3	....	54	V	<1

& 912-920	540	1.9	GERIVDIIA	54	46	30	15	1	3	....	54	.......V.	<1
& 913-921	537	2.5	ERIVDIIAT	46	54	26	26	2	4	....	46	......V..	<1
& 914-922	539	2.5	RIVDIIATD	46	54	26	26	2	4	....	46	.....V...	<1
& 915-923	535	2.8	IVDIIATDI	44	56	24	28	3	6	....	44	....V....	0
& 916-924	534	2.7	VDIIATDIQ	45	55	24	28	3	6	....	45	...V.....	0
& 917-925	545	1.9	DIIATDIQT	70	30	10	18	3	5	....	70	..V......	0
& 918-926	545	2.3	IIATDIQTK	64	36	10	22	4	7	....	64	.V.......	0
& 919-927	543	2.0	IATDIQTKE	69	31	10	18	4	6	....	69	V........	0
& 920-928	542	2.0	ATDIQTKEL	68	32	10	18	4	6	....	68	....	68
& 921-929	543	1.9	TDIQTKELQ	70	30	10	17	3	5	....	70	....	70
& 922-930	544	1.6	DIQTKELQK	77	23	6	15	3	4	....	77	....	77
& 923-931	544	1.6	IQTKELQKQ	75	25	6	17	2	4	....	75	....	75
& 924-932	549	1.4	QTKELQKQI	80	20	6	12	2	4	....	80	....	80
& 925-933	549	1.9	TKELQKQIT	73	27	5	20	2	5	....	73	....	73
& 926-934	548	2.1	KELQKQITK	71	29	5	22	3	6	....	71	....	71
& 927-935	540	2.1	ELQKQITKI	71	29	6	21	3	6	....	71	....	71
& 928-936	541	2.1	LQKQITKIQ	71	29	6	20	3	7	....	71	....	71
& 929-937	543	2.3	QKQITKIQN	69	31	6	21	4	8	....	69	....	69
& 930-938	544	2.3	KQITKIQNF	69	31	6	21	4	8	....	69	....	69
& 931-939	546	2.2	QITKIQNFR	70	30	6	20	5	8	....	70	....	70
& 932-940	546	2.1	ITKIQNFRV	72	28	6	18	5	8	....	72	....	72
& 933-941	546	2.0	TKIQNFRVY	72	28	6	17	5	8	....	72	....	72
& 934-942	545	1.4	KIQNFRVYY	83	17	2	11	4	6	....	83	....	83
& 935-943	546	1.3	IQNFRVYYR	84	16	3	10	3	5	....	84	....	84
& 936-944	549	0.9	QNFRVYYRD	88	12	3	6	2	3	....	88	....	88
& 937-945	548	1.4	NFRVYYRDS	78	22	7	14	1	3	....	78	....	78
& 938-946	547	1.2	FRVYYRDSR	80	20	9	10	1	2	....	80	....	80
& 939-947	545	1.5	RVYYRDSRD	75	25	9	15	2	3	........N	1	....	75
& 940-948	543	1.4	VYYRDSRDP	76	24	9	13	2	3	.......N.	1	....	76
& 941-949	541	2.2	YYRDSRDPL	62	38	11	25	2	4	......N..	1	I	11
& 942-950	541	2.2	YRDSRDPLW	62	38	11	25	2	4	.....N...	1	.......I.	11
& 943-951	544	1.9	RDSRDPLWK	64	36	11	23	1	3	....N....	1	......I..	11
& 944-952	545	2.0	DSRDPLWKG	64	36	11	23	2	3	...N.....	1	.....I...	11
& 945-953	545	1.9	SRDPLWKGP	65	35	11	23	2	3	..N......	1	....I....	11
& 946-954	546	1.3	RDPLWKGPA	77	23	11	10	1	2	.N.......	1	...I.....	11
& 947-955	544	1.4	DPLWKGPAK	77	23	8	15	1	3	N........	1	..I......	8
& 948-956	543	1.0	PLWKGPAKL	83	17	8	9	<1	2	....	83	.I.......	8
& 949-957	543	1.0	LWKGPAKLL	83	17	8	9	<1	2	....	83	I........	8
# 950-958	542	0.4	WKGPAKLLW	95	5	4	<1	<1	1	....	95	....	95
# 951-959	542	0.4	KGPAKLLWK	95	5	4	<1	<1	1	....	95	....	95
# 952-960	543	0.3	GPAKLLWKG	95	5	4	<1	<1	1	....	95	....	95
# 953-961	541	0.3	PAKLLWKGE	96	4	4	<1	<1	1	....	96	....	96
# 954-962	542	0.3	AKLLWKGEG	96	4	4	<1	<1	1	....	96	....	96
# 955-963	542	0.3	KLLWKGEGA	96	4	4	<1	<1	1	....	96	....	96
# 956-964	541	0.0	LLWKGEGAV	100	0	0	0	0	0	....	100	....	100
# 957-965	544	0.0	LWKGEGAVV	100	0	0	0	0	0	....	100	....	100
# 958-966	539	0.1	WKGEGAVVI	99	1	1	<1	0	<1	....	99	....	99
# 959-967	538	0.2	KGEGAVVIQ	98	2	1	<1	<1	1	....	98	....	98
# 960-968	538	0.3	GEGAVVIQD	96	4	1	2	<1	1	....	96	....	96
# 961-969	532	0.7	EGAVVIQDN	90	10	5	4	<1	2	....	90	....	90
& 962-970	531	0.9	GAVVIQDNS	89	11	4	5	2	3	....	89	....	89
& 963-971	528	1.6	AVVIQDNSD	70	30	19	9	3	5	....	70	....	70
& 964-972	526	1.6	VVIQDNSDI	69	31	19	9	3	5	....	69	....	69
& 965-973	527	1.7	VIQDNSDIK	69	31	19	9	3	5	....	69	....	69
& 966-974	525	1.7	IQDNSDIKV	69	31	19	9	3	5	....	69	....	69
& 967-975	527	1.5	QDNSDIKVV	71	29	19	7	3	5	....	71	....	71
& 968-976	528	1.5	DNSDIKVVP	71	29	19	7	3	4	....	71	....	71
& 969-977	526	1.4	NSDIKVVPR	72	28	20	6	3	4	....	72	....	72
& 970-978	528	1.3	SDIKVVPRR	72	28	20	6	2	2	....	72	....	72
& 971-979	527	0.9	DIKVVPRRK	77	23	21	<1	<1	1	....	77	....	77
& 972-980	526	0.9	IKVVPRRKA	75	25	24	<1	<1	1	....	75	....	75
& 973-981	523	0.9	KVVPRRKAK	75	25	24	<1	<1	1	....	75	....	75
& 974-982	522	0.9	VVPRRKAKI	75	25	24	<1	<1	1	....	75	....	75
& 975-983	522	1.0	VPRRKAKII	74	26	23	2	<1	2	....	74	....	74
& 976-984	519	1.1	PRRKAKIIR	73	27	22	4	<1	2	....	73	....	73
& 977-985	518	1.3	RRKAKIIRD	72	28	22	5	1	3	....	72	....	72
& 978-986	518	1.2	RKAKIIRDY	72	28	22	5	<1	2	....	72	....	72
& 979-987	518	1.2	KAKIIRDYG	72	28	22	5	<1	2	....	72	....	72
& 980-988	518	1.3	AKIIRDYGK	71	29	22	4	2	3	....	71	....	71
# 981-989	516	0.5	KIIRDYGKQ	94	6	2	3	<1	2	....	94	....	94
# 982-990	515	0.6	IIRDYGKQM	93	7	2	4	<1	2	....	93	....	93

	# 983-991	516	0.6	IRDYGKQMA	93	7	2	4	<1	2	....	93	....	93
	# 984-992	514	0.4	RDYGKQMAG	95	5	2	2	<1	2	....	95	....	95
	# 985-993	511	0.7	DYGKQMAGD	91	9	3	4	2	3	....	91	....	91
	# 986-994	506	0.7	YGKQMAGDD	90	10	4	4	2	3	....	90	....	90
	& 987-995	507	0.8	GKQMAGDDC	90	10	3	5	2	4	....	90	....	90
	& 988-996	505	0.9	KQMAGDDCV	90	10	3	5	3	5	....	90	....	90
	# 989-997	505	0.8	QMAGDDCVA	90	10	3	5	2	4	....	90	....	90
	& 990-998	500	1.3	MAGDDCVAS	80	20	10	8	2	5	....	80	....	80
	& 991-999	498	1.6	AGDDCVASR	75	25	10	12	3	5	....	75	....	75
	& 992-1000	493	1.6	GDDCVASRQ	75	25	10	12	3	5	....	75	....	75
	& 993-1001	478	1.8	DDCVASRQD	71	29	10	15	4	6	....	71	....	71
	& 994-1002	480	1.6	DCVASRQDE	72	28	11	13	3	5	....	72	....	72
	& 995-1003	468	1.7	CVASRQDED	72	28	12	13	4	6	....	72	....	72
Vif	# 1-9	1140	0.6	MENRWQVMI	94	6	2	4	1	2	....	94	....	94
	# 2-10	1140	0.6	ENRWQVMIV	94	6	2	4	1	2	....	94	....	94
	# 3-11	1141	0.6	NRWQVMIVW	94	6	2	4	<1	2	....	94	....	94
	# 4-12	1141	0.6	RWQVMIVWQ	94	6	2	4	<1	2	....	94	....	94
	# 5-13	1141	0.6	WQVMIVWQV	94	6	2	4	<1	2	....	94	....	94
	# 6-14	1141	0.7	QVMIVWQVD	93	7	2	5	<1	2	....	93	....	93
	# 7-15	1141	0.7	VMIVWQVDR	93	7	2	5	1	2	....	93	....	93
	# 8-16	1142	0.6	MIVWQVDRM	94	6	1	4	1	3	....	94	....	94
	# 9-17	1143	0.6	IVWQVDRMR	94	6	1	4	1	3	....	94	....	94
	# 10-18	1143	0.5	VWQVDRMRI	95	5	<1	3	1	3	....	95	....	95
	& 11-19	1139	1.6	WQVDRMRIR	73	27	14	11	2	4	....	73	....	73
	& 12-20	1136	2.0	QVDRMRIRT	65	35	13	19	3	4	....	65	....	65
	& 13-21	1136	2.0	VDRMRIRTW	66	34	13	19	3	4	....	66	....	66
	& 14-22	1137	2.9	DRMRIRTWK	49	51	15	33	3	5	....	49	I	<1
	& 15-23	1137	2.8	RMRIRTWKS	49	51	15	33	3	5	....	49	.......I.	<1
	& 16-24	1135	2.8	MRIRTWKSL	50	50	15	33	3	5	....	50	......I..	<1
	& 17-25	1135	2.8	RIRTWKSLV	50	50	15	32	3	4	....	50	.....I...	<1
	& 18-26	1135	2.8	IRTWKSLVK	50	50	16	32	3	5	....	50	....I....	<1
	& 19-27	1135	3.0	RTWKSLVKH	48	52	15	34	3	5	....	48	...I.....	<1
	& 20-28	1138	2.2	TWKSLVKHH	60	40	16	22	2	4	....	60	..I......	<1
	& 21-29	1137	2.4	WKSLVKHHM	57	43	15	26	2	4	....	57	.I.......	<1
	& 22-30	1134	2.9	KSLVKHHMY	54	46	14	29	3	5	....	54	I........	<1
	& 23-31	1130	3.5	SLVKHHMYI	39	61	20	38	3	7	V	20	....	39
	& 24-32	1130	3.5	LVKHHMYIS	40	60	20	37	3	7	.......V.	20	....	40
	+ 25-33	1131	4.7	VKHHMYISG	17	83	15	63	5	11	......V..	5	........K	15
	+ 26-34	1131	4.9	KHHMYISGK	17	83	14	63	6	12	.....V...	5	.......K.	14
	+ 27-35	1129	5.1	HHMYISGKA	16	84	13	64	7	14	....V....	4	......K..	13
	+ 28-36	1129	5.6	HMYVSKKAK	11	89	11	67	10	18	.....G..R	1	...I.....	11
	+ 29-37	1128	6.5	MYVSKKAKG	8	92	7	69	16	26	....G..R.	1	..I......	7
	+ 30-38	1130	6.1	YISKKAKGW	11	89	9	67	13	22	.V.G..R..	1	....	11
	+ 31-39	1119	7.2	ISGKAKGWF	4	96	4	74	19	31	V....R...	1	..K.....V	4
	+ 32-40	1126	6.4	SKKAKGWFY	8	92	6	73	13	22	.G..R....	2	.......V.	6
	+ 33-41	1125	6.8	KKAKGWFYR	7	93	6	73	14	25	G..R.....	2	......V..	4
	+ 34-42	1126	5.9	KAKGWFYRH	16	84	8	66	10	18	..R......	2	.....V...	8
	+ 35-43	1126	5.8	AKGWFYRHH	17	83	8	66	9	17	.R.......	2	....V....	8
	+ 36-44	1128	5.7	KGWFYRHHY	17	83	8	67	8	16	R........	3	...V.....	8
	& 37-45	1128	5.1	GWFYRHHYE	21	79	12	61	7	13	....	21	..V......	12
	& 38-46	1127	4.2	WFYRHHYES	31	69	18	47	4	9	....	31	.V......N	3
	& 39-47	1123	5.3	FYRHHYEST	22	78	13	56	8	16	........P	5	V......NP	1
	& 40-48	1133	4.2	YRHHYESTH	35	65	12	47	6	10	.......P.	8	......NP.	1
	& 41-49	1133	4.2	RHHYESTHP	35	65	12	46	6	10	......P..	8	.....NP..	1
	& 42-50	1133	4.2	HHYESTHPR	28	72	17	50	5	10	.....P...	7	....NP...	2
	& 43-51	1133	4.6	HYESTHPRI	26	74	14	53	6	12	....P....	7	...NP...V	<1
	& 44-52	1132	4.7	YESTHPRIS	26	74	14	53	7	13	...P.....	7	..NP...VG	<1
	& 45-53	1132	4.6	ESTHPRISS	26	74	14	53	6	12	..P......	7	.NP...VG.	<1
	& 46-54	1132	4.5	STHPRISSE	26	74	14	55	6	11	.P.......	6	NP...VG..	<1
	& 47-55	1135	4.2	THPRISSEV	28	72	14	54	4	9	P........	7	P...VG...	<1
	& 48-56	1138	3.0	HPRISSEVH	38	62	19	40	3	5	....	38	...VG....	<1
	& 49-57	1139	2.3	PRISSEVHI	57	43	22	19	2	5	....	57	..VG.....	<1
	& 50-58	1139	2.3	RISSEVHIP	57	43	22	18	3	5	....	57	.VG......	<1
	& 51-59	1142	1.7	ISSEVHIPL	78	22	7	13	2	4	....	78	VG.......	<1
	& 52-60	1141	1.4	SSEVHIPLG	83	17	4	11	2	4	....	83	G.......E	<1
	& 53-61	1136	2.2	SEVHIPLGD	64	36	18	14	3	5	....	64	.......E.	2
	& 54-62	1132	2.6	EVHIPLGDA	60	40	17	19	4	8	....	60	......E..	2
	& 55-63	1131	3.9	VHIPLGDAK	35	65	21	37	7	12	........R	21	.....E...	2
	& 56-64	1131	4.1	HIPLGDAKL	34	66	19	40	8	12	.......R.	19	....E....	2
	& 57-65	1124	4.1	IPLGDAKLV	34	66	19	40	8	13	......R..	19	...E.....	2
	& 58-66	1120	4.8	PLGDAKLVI	26	74	12	52	9	15	.....R...	12	..E.....V	<1

& 59-67	1119	5.6	LGDAKLVIT	21	79	11	57	11	19	....R....	11	.E.....V.	0
& 60-68	1119	5.5	GDAKLVITT	21	79	11	58	10	18	...R.....	11	E.....V..	0
& 61-69	1120	5.3	DAKLVITTY	23	77	11	57	8	16	..R......	11	.....V...	4
& 62-70	1125	4.6	AKLVITTYW	26	74	14	55	5	11	.R.......	14	....V....	5
& 63-71	1129	4.2	KLVITTYWG	28	72	16	53	4	8	R........	16	...V....V	0
& 64-72	1132	2.9	LVITTYWGL	50	50	17	31	2	4	....	50	..V....V.	0
& 65-73	1130	3.0	VITTYWGLH	48	52	18	31	3	5	....	48	.V....V..	0
& 66-74	1135	2.9	ITTYWGLHT	49	51	18	30	3	5	....	49	V....V...	0
& 67-75	1137	2.1	TTYWGLHTG	67	33	9	22	2	4	....	67	....V....	0
& 68-76	1138	0.9	TYWGLHTGE	89	11	4	6	1	3	....	89	...V.....	0
& 69-77	1138	1.2	YWGLHTGER	85	15	4	10	1	3	....	85	..V......	0
& 70-78	1137	2.1	WGLHTGERD	63	37	20	16	1	4	....	63	.V.......	0
& 71-79	1137	2.1	GLHTGERDW	63	37	20	16	1	4	....	63	V........	0
& 72-80	1136	2.1	LHTGERDWH	62	38	20	16	2	4	....	62	....	62
& 73-81	1135	2.2	HTGERDWHL	62	38	19	16	2	4	....	62	....	62
& 74-82	1139	1.8	TGERDWHLG	65	35	21	12	2	3	....	65	....	65
& 75-83	1140	1.8	GERDWHLGQ	66	34	21	11	2	4	....	66	....	66
& 76-84	1140	1.8	ERDWHLGQG	66	34	21	11	2	4	....	66	....	66
& 77-85	1139	2.0	RDWHLGQGV	65	35	20	13	2	4	....	65	....	65
& 78-86	1139	1.8	DWHLGQGVS	67	33	21	10	2	4	....	67	....	67
& 79-87	1140	1.1	WHLGQGVSI	87	13	3	9	<1	3	....	87	....	87
& 80-88	1140	1.1	HLGQGVSIE	87	13	3	10	<1	3	....	87	....	87
& 81-89	1141	1.1	LGQGVSIEW	87	13	3	9	<1	3	....	87	....	87
& 82-90	1142	1.2	GQGVSIEWR	85	15	3	11	1	3	....	85	....	85
& 83-91	1138	2.0	QGVSIEWRK	75	25	3	19	2	5	....	75	....	75
& 84-92	1133	3.5	GVSIEWRKK	33	67	27	37	4	7	....	33	........G	5
& 85-93	1131	4.3	VSIEWRKKR	28	72	22	43	7	12	....	28	.......GN	0
& 86-94	1132	4.0	SIEWRKKRY	29	71	23	42	6	11	....	29	......GN.	0
& 87-95	1129	4.9	IEWRKKRYS	22	78	19	51	8	15	....	22	.....GN..	0
& 88-96	1130	4.8	EWRKKRYST	22	78	20	51	7	14	....	22	....GN...	0
& 89-97	1130	4.8	WRKKRYSTQ	22	78	20	51	7	14	....	22	...GN....	0
+ 90-98	1129	5.0	RKKRYSTQV	20	80	19	53	8	15	....	20	..GN.....	0
+ 91-99	1128	5.1	KKRYSTQVD	19	81	19	53	9	16	....	19	.GN......	0
& 92-100	1130	4.7	RRYSTQVDP	21	79	21	52	6	12	K........	21	GN.......	0
+ 93-101	1130	4.6	RYSTQVDPG	18	82	18	58	6	12	........E	1	N........	0
& 94-102	1129	4.1	YSTQVDPDL	20	80	19	56	4	9	.......E.	1	.......G.	19
+ 95-103	1129	4.2	STQVDPDLA	20	80	19	57	5	9	......E..	1	......G..	19
& 96-104	1133	3.2	TQVDPDLAD	35	65	22	39	3	6	.....E...	2	.....G...	22
& 97-105	1133	3.8	QVDPDLADQ	23	77	21	53	4	7	....E....	2	....G...R	1
& 98-106	1133	3.8	VDPDLADQL	23	77	21	53	3	7	...E.....	2	...G...R.	1
& 99-107	1134	3.4	DPDLADQLI	25	75	23	49	3	6	..E......	2	..G...R..	2
& 100-108	1134	3.1	PDLADQLIH	29	71	24	45	3	5	.E.......	2	.G...R...	2
& 101-109	1132	3.9	DLADQLIHL	26	74	17	54	4	7	E........	1	G...R....	2
& 102-110	1136	3.3	LADQLIHLY	47	53	10	40	3	6	....	47	...R.....	2
& 103-111	1142	3.1	ADQLIHLYY	48	52	11	39	2	5	....	48	..R......	2
& 104-112	1142	3.1	DQLIHLYYF	48	52	11	38	2	5	....	48	.R.......	3
& 105-113	1141	3.2	QLIHLYYFD	47	53	12	39	3	5	....	47	R........	2
& 106-114	1141	2.6	LIHLYYFDC	50	50	23	25	2	4	....	50	....	50
& 107-115	1141	2.6	IHLYYFDCF	50	50	23	25	2	4	....	50	....	50
& 108-116	1141	2.9	HLYYFDCFS	49	51	22	26	3	5	....	49	....	49
& 109-117	1139	3.4	LYYFDCFSE	46	54	12	39	4	7	........D	3	....	46
& 110-118	1140	2.8	YYFDCFSES	52	48	12	34	3	5	.......D.	4	........T	2
& 111-119	1140	2.1	YFDCFSESA	64	36	17	18	2	3	......D..	17	.......T.	2
& 112-120	1141	2.0	FDCFSESAI	64	36	17	18	2	3	.....D...	17	......T..	2
& 113-121	1141	2.1	DCFSESAIR	63	37	17	18	2	4	....D....	17	.....T...	2
& 114-122	1139	3.4	CFSESAIRN	44	56	11	42	3	6	...D....K	3	....T....	2
& 115-123	1138	3.7	FSESAIRNA	40	60	10	46	3	7	..D....K.	2	...T.....	2
& 116-124	1137	4.1	SESAIRNAI	36	64	8	52	4	8	.D....K.L	1	..T......	2
& 117-125	1135	4.0	ESAIRNAIL	37	63	10	48	4	8	D....K.L.	1	.T.......	2
& 118-126	1135	3.5	SAIRNAILG	40	60	12	44	4	7	....K.L..	3	T........	2
& 119-127	1135	4.7	AIRNAILGH	25	75	13	56	6	11	...K.L...	2	....	25
+ 120-128	1133	5.7	IRNAILGHI	15	85	8	67	9	16	..K.L....	2	........R	8
+ 121-129	1131	5.7	RNAILGHIV	14	86	8	68	9	16	.K.L.....	2	.......R.	8
+ 122-130	1130	6.4	NAILGHIVS	10	90	7	72	12	21	K.L......	1	......R..	7
+ 123-131	1133	5.5	AILGHIVSP	15	85	11	66	8	15	.L.......	2	.....R...	11
+ 124-132	1129	6.1	ILGHRVSPR	9	91	8	74	9	18	L...I....	1	........S	6
+ 125-133	1132	5.8	LGHRVSPRC	11	89	9	71	8	16	...I.....	9	.......S.	6
+ 126-134	1133	5.9	GHRVSPRCE	11	89	9	70	9	18	..I......	9	......S..	7
+ 127-135	1133	6.0	HRVSPRCEY	11	89	9	70	10	19	.I.......	9	.....S...	7
+ 128-136	1134	5.4	RVSPRCEYQ	15	85	14	62	9	17	I........	14	....S....	7
& 129-137	1136	4.4	VSPRCEYQA	32	68	17	44	7	13	....	32	...S.....	17

	[&] 130-138	1136	4.3	SPRCEYQAG	32	68	18	43	7	13	....	32	..S......	18
	[&] 131-139	1137	3.7	PRCEYQAGH	37	63	21	38	4	9	....	37	.S.......	21
	[&] 132-140	1135	3.9	RCEYQAGHN	36	64	19	41	5	9	....	36	S........	19
	[&] 133-141	1134	3.0	CEYQAGHNK	59	41	7	31	4	7	....	59	....	59
	[&] 134-142	1132	3.0	EYQAGHNKV	59	41	7	31	4	8	....	59	....	59
	[&] 135-143	1134	2.3	YQAGHNKVG	70	30	4	23	3	6	....	70	....	70
	[&] 136-144	1133	2.2	QAGHNKVGS	73	27	3	21	3	6	....	73	....	73
	[&] 137-145	1134	1.8	AGHNKVGSL	77	23	4	17	2	4	....	77	....	77
	[&] 138-146	1134	1.5	GHNKVGSLQ	81	19	4	14	2	4	....	81	....	81
	[&] 139-147	1133	1.6	HNKVGSLQY	80	20	4	14	2	4	....	80	....	80
	[&] 140-148	1132	1.5	NKVGSLQYL	81	19	4	14	2	4	....	81	....	81
	[&] 141-149	1134	1.1	KVGSLQYLA	86	14	4	9	<1	3	....	86	....	86
	[#] 142-150	1137	0.7	VGSLQYLAL	93	7	2	4	1	2	....	93	....	93
	[&] 143-151	1135	2.2	GSLQYLALA	45	55	36	17	1	3	....	45	........T	36
	[&] 144-152	1134	2.4	SLQYLALAA	41	59	37	20	2	3	....	41	.......T.	37
	[&] 145-153	1135	2.3	LQYLALAAL	42	58	37	19	2	4	....	42	......T..	37
	[&] 146-154	1130	3.5	QYLALTALI	32	68	25	41	3	6	.....A...	25	....	32
	[&] 147-155	1129	4.4	YLALTALIT	25	75	20	49	6	10	....A....	20	....	25
	[&] 148-156	1129	4.5	LALTALITP	25	75	19	49	7	12	...A.....	19	....	25
	[&] 149-157	1128	4.8	ALTALITPK	24	76	18	50	8	14	..A......	18	....	24
	[+] 150-158	1125	5.5	LTALITPKK	20	80	15	55	10	18	.A.......	15	........R	4
	[+] 151-159	1121	6.4	TALITPKKI	14	86	11	60	15	25	A........	11	.......R.	2
	[&] 152-160	1124	5.5	ALITPKKIK	26	74	5	57	11	20	....	26	......R.R	<1
	[&] 153-161	1124	5.4	LITPKKIKP	28	72	6	56	11	19	....	28	.....R.R.	<1
	[&] 154-162	1124	5.4	ITPKKIKPP	28	72	6	56	11	19	....	28	....R.R..	<1
	[&] 155-163	1127	4.4	TPKKIKPPL	39	61	7	46	8	13	....	39	...R.R...	<1
	[&] 156-164	1128	3.6	PKKIKPPLP	47	53	8	40	5	9	....	47	..R.R....	<1
	[&] 157-165	1129	3.5	KKIKPPLPS	48	52	8	40	4	8	....	48	.R.R.....	<1
	[&] 158-166	1130	3.2	KIKPPLPSV	50	50	8	38	4	7	....	50	R.R......	<1
	[&] 159-167	1132	4.4	IKPPLPSVR	21	79	14	60	5	9	........T	14	.R.......	<1
	[&] 160-168	1136	3.1	KPPLPSVRK	25	75	22	50	3	5	.......T.	22	R........	1
	[&] 161-169	1136	2.8	PPLPSVRKL	26	74	23	48	3	4	......T..	23	....	26
	[&] 162-170	1137	2.9	PLPSVRKLT	26	74	23	48	3	5	.....T...	23	....	26
	[&] 163-171	1137	2.8	LPSVRKLTE	26	74	23	48	3	5	....T....	23	....	26
	[&] 164-172	1136	2.8	PSVRKLTED	26	74	23	48	3	5	...T.....	23	....	26
	[&] 165-173	1136	2.9	SVRKLTEDR	26	74	23	49	3	5	..T......	23	....	26
	[&] 166-174	1135	2.8	VRKLTEDRW	26	74	23	49	2	4	.T.......	23	....	26
	[&] 167-175	1135	2.6	RKLTEDRWN	26	74	25	47	2	4	T........	23	....	26
	[&] 168-176	1135	0.8	KLTEDRWNK	90	10	6	3	1	2	....	90	....	90
	[&] 169-177	1135	0.9	LTEDRWNKP	88	12	6	5	<1	2	....	88	....	88
	[&] 170-178	1129	1.3	TEDRWNKPQ	81	19	6	11	1	3	....	81	....	81
	[&] 171-179	1127	2.0	EDRWNKPQK	69	31	10	18	2	4	....	69	....	69
	[&] 172-180	1126	2.2	DRWNKPQKT	66	34	10	21	3	5	....	66	....	66
	[&] 173-181	1126	2.5	RWNKPQKTK	63	37	10	24	3	6	....	63	....	63
	[&] 174-182	1126	2.7	WNKPQKTKG	60	40	9	28	3	6	....	60	........D	4
	[&] 175-183	1124	2.9	NKPQKTKGH	58	42	9	29	4	8	....	58	.......D.	4
	[&] 176-184	1124	3.2	KPQKTKGHR	54	46	9	33	4	8	....	54	......D..	3
	[&] 177-185	1123	3.4	PQKTKGHRG	50	50	7	38	5	8	....	50	.....D...	3
	[&] 178-186	1116	3.6	QKTKGHRGS	48	52	7	39	6	10	....	48	....D....	3
	[&] 179-187	1112	3.5	KTKGHRGSH	50	50	7	38	5	9	....	50	...D.....	4
	[&] 180-188	1113	3.1	TKGHRGSHT	56	44	8	32	4	8	....	56	..D......	5
	[&] 181-189	1114	3.3	KGHRGSHTM	56	44	8	32	5	10	....	56	.D.......	5
	[&] 182-190	1111	3.6	GHRGSHTMN	52	48	6	35	7	11	....	52	D........	4
	[&] 183-191	1111	3.4	HRGSHTMNG	56	44	7	32	6	11	....	56	....	56
	[&] 184-192	1109	3.3	RGSHTMNGH	56	44	7	31	6	10	....	56	....	56
Vpr	[&] 1-9	994	1.4	MEQAPEDQG	82	18	5	11	2	4	....	82	....	82
	[&] 2-10	992	1.4	EQAPEDQGP	81	19	5	11	3	5	....	81	....	81
	[&] 3-11	987	1.7	QAPEDQGPQ	79	21	5	13	3	5	....	79	....	79
	[&] 4-12	993	1.4	APEDQGPQR	83	17	4	10	3	5	....	83	....	83
	[&] 5-13	991	1.4	PEDQGPQRE	83	17	4	11	3	5	....	83	....	83
	[&] 6-14	986	1.3	EDQGPQREP	83	17	4	10	3	4	....	83	....	83
	[&] 7-15	984	2.0	DQGPQREPY	74	26	6	16	4	6	........H	6	....	74
	[&] 8-16	980	2.2	QGPQREPYN	72	28	6	18	4	7	.......H.	6	....	72
	[&] 9-17	970	2.6	GPQREPYNE	67	33	6	21	6	9	......H..	6	....	67
	[&] 10-18	971	2.6	PQREPYNEW	67	33	6	22	6	9	.....H...	6	....	67
	[&] 11-19	968	3.0	QREPYNEWT	59	41	8	27	6	10	....H....	6	....	59
	[&] 12-20	967	2.9	REPYNEWTL	60	40	8	27	6	9	...H.....	7	....	60
	[&] 13-21	960	2.9	EPYNEWTLE	59	41	8	28	5	9	..H......	7	....	59
	[&] 14-22	958	2.9	PYNEWTLEL	60	40	8	28	5	9	.H.......	7	....	60
	[&] 15-23	961	2.9	YNEWTLELL	60	40	8	28	5	9	H........	7	....	60
	[&] 16-24	973	2.1	NEWTLELLE	72	28	8	16	4	8	....	72	....	72

[&] 17-25	982	1.9	EWTLELLEE	75	25	8	14	3	6	....	75	....	75
[&] 18-26	1001	1.4	WTLELLEEL	81	19	9	8	2	4	....	81	....	81
[&] 19-27	1002	1.3	TLELLEELK	81	19	9	8	2	4	....	81	....	81
[&] 20-28	989	2.5	LELLEELKN	52	48	23	21	3	5	....	52	....	52
[&] 21-29	990	2.5	ELLEELKNE	52	48	23	21	3	6	....	52	....	52
[&] 22-30	995	2.5	LLEELKNEA	53	47	23	21	3	6	....	53	....	53
[&] 23-31	999	2.5	LEELKNEAV	53	47	22	21	3	6	....	53	....	53
[&] 24-32	991	2.8	EELKNEAVR	50	50	21	25	4	7	....	50	....	50
[&] 25-33	992	2.8	ELKNEAVRH	51	49	21	25	4	7	....	51	....	51
[&] 26-34	995	2.6	LKNEAVRHF	52	48	21	24	3	6	....	52	....	52
[&] 27-35	995	2.6	KNEAVRHFP	52	48	21	24	3	6	....	52	....	52
[&] 28-36	990	2.8	NEAVRHFPR	51	49	20	26	4	7	....	51	....	51
[&] 29-37	960	3.6	EAVRHFPRP	28	72	19	47	5	8	I	19	V	11
[&] 30-38	962	3.5	AVRHFPRPW	28	72	19	48	4	7	.......I.	19	.......V.	12
[&] 31-39	962	3.5	VRHFPRPWL	28	72	19	48	4	7	......I..	19	......V..	12
[&] 32-40	948	3.7	RHFPRPWLH	26	74	19	51	4	8	.....I...	19	.....V...	12
[&] 33-41	940	4.5	HFPRPWLHG	20	80	14	60	5	11	....I....	14	....V....	10
[&] 34-42	940	4.5	FPRPWLHGL	20	80	14	60	5	11	...I.....	14	...V.....	10
[&] 35-43	941	4.5	PRPWLHGLG	20	80	14	60	5	11	..I......	14	..V......	10
[+] 36-44	938	4.6	RPWLHGLGQ	20	80	14	60	6	12	.I.......	14	.V.......	10
[+] 37-45	915	5.2	PWLHGLGQY	14	86	11	66	8	15	I.......H	11	V.......H	7
[&] 38-46	954	3.0	WLHGLGQHI	35	65	26	36	3	6	....	35	....	35
[&] 39-47	953	3.0	LHGLGQHIY	35	65	26	36	3	6	....	35	....	35
[&] 40-48	945	3.8	HGLGQHIYE	33	67	16	46	5	10	....	33	....	33
[&] 41-49	961	3.7	GLGQHIYET	35	65	17	43	6	10	....	35	....	35
[&] 42-50	974	2.6	LGQHIYETY	43	57	35	19	3	7	....	43	....	43
[&] 43-51	974	2.7	GQHIYETYG	43	57	34	19	3	7	....	43	....	43
[&] 44-52	971	2.7	QHIYETYGD	43	57	34	20	3	7	....	43	....	43
[&] 45-53	975	2.6	HIYETYGDT	43	57	35	19	3	6	....	43	....	43
[&] 46-54	1009	1.6	IYETYGDTW	79	21	4	14	2	5	....	79	....	79
[&] 47-55	982	2.5	YETYGDTWA	63	37	10	23	4	7	....	63	........E	5
[&] 48-56	983	2.6	ETYGDTWAG	63	37	10	24	4	7	....	63	.......E.	5
[&] 49-57	994	1.9	TYGDTWAGV	67	33	10	20	3	4	....	67	......E..	10
[&] 50-58	992	2.0	YGDTWAGVE	66	34	13	18	2	5	....	66	.....E...	13
[&] 51-59	987	2.1	GDTWAGVEA	66	34	13	19	3	6	....	66	....E....	13
[&] 52-60	969	2.6	DTWAGVEAI	55	45	12	30	3	6	....	55	...E....M	<1
[&] 53-61	965	3.1	TWAGVEAII	51	49	12	33	4	8	....	51	..E....ML	<1
[&] 54-62	963	3.3	WAGVEAIIR	50	50	11	33	5	10	....	50	.E....ML.	<1
[&] 55-63	929	4.2	AGVEAIIRI	39	61	9	43	9	15	....	39	E....ML..	<1
[&] 56-64	950	3.2	GVEAIIRIL	55	45	7	33	5	10	....	55	....ML...	<1
[&] 57-65	948	3.3	VEAIIRILQ	55	45	6	32	6	10	....	55	...ML....	<1
[&] 58-66	948	3.3	EAIIRILQQ	55	45	6	33	6	11	....	55	..ML.....	<1
[&] 59-67	946	3.0	AIIRILQQL	58	42	6	30	5	10	....	58	.ML......	<1
[&] 60-68	948	3.1	IIRILQQLL	58	42	6	31	5	9	....	58	ML.......	<1
[&] 61-69	957	2.6	IRILQQLLF	63	37	8	25	3	7	....	63	L........	<1
[&] 62-70	955	2.8	RILQQLLFI	59	41	8	30	4	7	....	59	....	59
[&] 63-71	956	2.6	ILQQLLFIH	60	40	8	29	3	6	....	60	....	60
[&] 64-72	995	1.6	LQQLLFIHF	76	24	11	12	2	3	....	76	....	76
[&] 65-73	995	1.6	QQLLFIHFR	75	25	11	13	2	3	........Q	1	....	75
[&] 66-74	995	1.7	QLLFIHFRI	74	26	11	14	2	4	.......QN	1	....	74
[&] 67-75	997	1.8	LLFIHFRIG	73	27	11	15	1	3	......QNW	1	....	73
[&] 68-76	1004	1.6	LFIHFRIGC	75	25	11	13	1	3	.....QNWV	1	....	75
[&] 69-77	984	2.6	FIHFRIGCR	51	49	20	26	2	4	....QNWV-	0	....	51
[&] 70-78	989	2.5	IHFRIGCRH	52	48	20	26	2	4	...QNWV--	0	....	52
[&] 71-79	1001	1.8	HFRIGCRHS	56	44	29	12	2	3	..QNWV--.	0	....	56
[&] 72-80	1001	1.9	FRIGCRHSR	56	44	29	12	2	3	.QNWV--.T	0	....	56
[&] 73-81	1002	1.7	RIGCRHSRI	57	43	31	10	2	3	QNWV--.T-	0	....	57
[&] 74-82	1002	1.8	IGCRHSRIG	56	44	31	10	2	3	NWV--.T--	0	....	56
[&] 75-83	999	1.7	GCRHSRIGI	57	43	31	9	3	4	WV--.T---	0	....	57
[&] 76-84	943	3.5	CRHSRIGIT	30	70	17	49	4	7	V--.T----	0	I	17
[+] 77-85	924	5.2	RHSRIGITP	12	88	8	73	7	13	--.T-----	0	.......I.	5
[+] 78-86	889	5.0	HSRIGITPQ	14	86	12	66	9	14	-.T------	0	......I..	6
[+] 79-87	886	5.3	SRIGITPQR	13	87	10	65	11	17	.T-------	0	.....I...	6
[+] 80-88	883	5.4	RIGITPQRR	13	87	11	65	11	19	T--------	0	....I....	5
[+] 81-89	872	6.0	IGITPQRRA	10	90	9	64	16	25	---------	0	...I....T	1
[+] 82-90	875	6.0	GITPQRRAR	10	90	9	64	16	25	---------	0	..I....T.	1
[+] 83-91	873	6.2	ITPQRRARN	10	90	9	62	18	27	---------	0	.I....T..	1
[+] 84-92	875	6.1	TPQRRARNG	10	90	9	63	17	26	---------	0	I....T...	1
[+] 85-93	905	5.4	RQRRARNGA	16	84	15	54	15	22	---------	0	P...T...S	<1
[&] 86-94	922	4.4	QRRARNGAS	41	59	8	39	12	18	---------	0	...T...S.	2
[&] 87-95	970	3.8	RRARNGASR	48	52	10	34	8	14	---------	0	..T...S..	2

	& 88-96	980	3.7	RARNGASRS	48	52	10	35	7	12	---------	0	.T...S...	2
Tat	& 1-9	1257	2.3	MEPVDPRLE	65	35	9	25	1	4	....	65	.D.......	6
	& 2-10	1260	2.3	EPVDPRLEP	65	35	9	25	1	4	....	65	D........	6
	& 3-11	1261	1.9	PVDPRLEPW	71	29	10	18	1	3	....	71	....	71
	& 4-12	1259	2.3	VDPRLEPWK	67	33	9	22	2	4	....	67	....	67
	& 5-13	1258	2.2	DPRLEPWKH	68	32	9	21	2	4	....	68	....	68
	& 6-14	1258	2.2	PRLEPWKHP	68	32	9	21	2	4	....	68	....	68
	& 7-15	1258	2.0	RLEPWKHPG	70	30	9	19	2	4	....	70	....	70
	& 8-16	1264	1.1	LEPWKHPGS	86	14	3	10	<1	2	....	86	....	86
	& 9-17	1264	1.2	EPWKHPGSQ	85	15	2	11	1	3	....	85	....	85
	& 10-18	1264	1.0	PWKHPGSQP	88	12	3	8	<1	2	....	88	....	88
	& 11-19	1260	2.2	WKHPGSQPK	58	42	23	16	2	4	....	58	....	58
	& 12-20	1261	2.3	KHPGSQPKT	58	42	24	16	2	4	....	58	....	58
	& 13-21	1262	2.4	HPGSQPKTA	56	44	22	20	2	5	....	56	....	56
	& 14-22	1264	2.5	PGSQPKTAC	56	44	22	20	2	5	....	56	....	56
	& 15-23	1263	3.3	GSQPKTACT	43	57	15	38	4	7	....	43	....	43
	& 16-24	1258	4.9	SQPKTACTN	24	76	9	61	6	12	....	24	....	24
	& 17-25	1258	4.8	QPKTACTNC	24	76	9	61	6	12	....	24	....	24
	& 18-26	1257	4.9	PKTACTNCY	23	77	9	61	7	12	....	23	....	23
	& 19-27	1257	4.8	KTACTNCYC	23	77	9	61	7	12	....	23	....	23
	& 20-28	1260	3.9	TACTNCYCK	31	69	15	50	4	8	....	31	....	31
	& 21-29	1251	4.9	ACTNCYCKK	24	76	11	59	6	12	....	24	....	24
	& 22-30	1254	4.3	CTNCYCKKC	29	71	12	54	5	9	....	29	....	29
	& 23-31	1254	4.3	TNCYCKKCC	29	71	12	55	4	9	....	29	....	29
	& 24-32	1252	4.5	NCYCKKCCF	30	70	10	55	5	11	....	30	L	8
	& 25-33	1257	2.9	CYCKKCCFH	55	45	11	31	2	6	....	55	.......L.	11
	& 26-34	1255	2.9	YCKKCCFHC	55	45	11	31	3	6	....	55	......L..	11
	& 27-35	1255	2.9	CKKCCFHCQ	55	45	11	31	3	6	....	55	.....L...	11
	& 28-36	1247	3.3	KKCCFHCQV	52	48	11	32	5	9	....	52	....L....	11
	& 29-37	1246	3.3	KCCFHCQVC	52	48	11	32	5	9	....	52	...L.....	11
	& 30-38	1256	2.2	CCFHCQVCF	66	34	16	15	4	6	....	66	..L......	16
	& 31-39	1251	3.8	CFHCQVCFI	32	68	24	38	6	9	....	32	.L.......	3
	& 32-40	1249	5.0	FHCQVCFIT	21	79	12	59	8	13	....	21	L.......Q	<1
	& 33-41	1250	4.3	HCQVCFITK	25	75	12	56	6	10	....	25	.......Q.	2
	& 34-42	1250	4.8	CQVCFITKG	22	78	10	60	7	11	........A	3	......Q..	2
	& 35-43	1250	4.8	QVCFITKGL	22	78	10	60	7	11	.......A.	3	.....Q...	2
	& 36-44	1252	4.7	VCFITKGLG	23	77	10	61	6	11	......A..	3	....Q....	2
	& 37-45	1220	4.2	CFITKGLGI	26	74	11	59	4	7	.....A...	3	...Q.....	2
	& 38-46	1218	4.5	FITKGLGIS	26	74	11	58	5	9	....A....	3	..Q......	<1
	& 39-47	1218	4.6	ITKGLGISY	25	75	11	59	5	10	...A.....	3	.Q.......	<1
	& 40-48	1226	3.1	TKGLGISYG	47	53	13	37	3	6	..A......	6	Q........	1
	& 41-49	1230	1.7	KGLGISYGR	73	27	14	11	2	4	.A.......	14	....	73
	& 42-50	1248	1.7	GLGISYGRK	72	28	14	13	1	3	A........	14	....	72
	& 43-51	1252	1.0	LGISYGRKK	87	13	3	8	2	3	....	87	....	87
	& 44-52	1251	1.2	GISYGRKKR	85	15	3	11	2	3	....	85	....	85
	& 45-53	1249	1.4	ISYGRKKRR	83	17	2	13	2	3	....	83	....	83
	& 46-54	1245	1.5	SYGRKKRRQ	81	19	2	14	3	4	....	81	....	81
	& 47-55	1246	1.2	YGRKKRRQR	86	14	2	9	2	4	....	86	....	86
	& 48-56	1247	1.0	GRKKRRQRR	89	11	3	6	2	4	....	89	....	89
	& 49-57	1245	1.5	RKKRRQRRR	82	18	3	12	3	4	....	82	....	82
	& 50-58	1239	3.0	KKRRQRRRA	47	53	16	33	4	7	....	47	........P	16
	& 51-59	1230	3.8	KRRQRRRAP	36	64	15	44	5	9	........H	5	.......PS	<1
	& 52-60	1243	4.6	RRQRRRAPQ	30	70	13	49	9	14	.......H.	5	......PS.	<1
	& 53-61	1238	5.4	RQRRRAPQD	25	75	9	54	11	18	......H.N	1	.....PS..	<1
	& 54-62	1237	6.0	QRRRAPQDS	20	80	7	58	14	22	.....H.N.	1	....PS..C	0
	+ 55-63	1236	6.6	RRRAPQDSQ	14	86	5	66	16	25	....H.N..	<1	...PS..CK	0
	+ 56-64	1229	7.2	RRAPQDSQT	10	90	4	63	22	33	...H.N...	<1	..PS..CK.	0
	+ 57-65	1226	7.4	RAPQDSQTH	10	90	4	61	25	37	..H.N....	<1	.PS..CK..	0
	+ 58-66	1225	7.4	APQDSQTHQ	10	90	4	61	24	35	.H.N.....	<1	PS..CK...	0
	+ 59-67	1227	7.3	PQDSQTHQV	8	92	7	61	23	35	H.N.....A	<1	S..CK...A	0
	+ 60-68	1227	7.4	QDSQTHQVS	8	92	7	61	24	36	.N.....A.	1	..CK...A.	0
	+ 61-69	1224	7.1	DSQTHQVSL	9	91	7	62	21	32	N.....A..	1	.CK...A..	0
	+ 62-70	1227	6.7	SQTHQVSLS	13	87	8	61	18	29	.....A...	8	CK...A...	0
	+ 63-71	1224	6.4	QTHQVSLSK	16	84	8	59	16	26	....A....	8	K...A....	1
	& 64-72	1016	5.5	THQVSLSKQ	20	80	13	54	13	20	...A.....	13	...A.....	13
	& 65-73	601	4.9	HQVSLSKQP	26	74	15	46	12	20	..A......	15	..A......	15
	+ 66-74	600	5.4	QVSLSKQPA	14	86	12	58	16	24	.A......T	5	.A......T	5
	+ 67-75	601	5.7	VSLSKQPAS	14	86	10	59	17	27	A......T.	5	A......T.	5
	& 68-76	599	5.1	SLSKQPASQ	23	77	16	45	16	23	......T..	16	......T..	16
	+ 69-77	593	5.5	LSKQPASQP	20	80	11	51	18	27	.....T...	11	.....T...	11
	+ 70-78	594	5.7	SKQPASQPR	17	83	10	57	16	26	....T....	10	....T...G	<1

	& 71-79	598	5.2	KQPASQPRG	24	76	14	47	15	24	...T.....	14	...T...G.	1
	& 72-80	604	5.2	QPASQPRGD	25	75	14	46	15	24	..T......	14	..T...G..	1
	+ 73-81	861	5.7	PASQPRGDP	19	81	13	55	13	22	.T.......	13	.T...G...	4
	+ 74-82	861	5.8	ASQPRGDPT	18	82	12	55	14	24	T........	12	T...G....	4
	& 75-83	860	4.8	SQPRGDPTG	35	65	6	48	11	18	....	35	...G.....	5
	& 76-84	859	4.7	QPRGDPTGP	37	63	6	45	11	18	....	37	..G......	5
	& 77-85	860	5.1	PRGDPTGPK	31	69	5	51	13	20	....	31	.G.......	5
	& 78-86	861	4.1	RGDPTGPKE	45	55	6	39	10	15	....	45	G........	6
	& 79-87	779	4.0	GDPTGPKES	42	58	7	42	8	13	........-	0	....	42
	& 80-88	779	4.4	DPTGPKESK	39	61	7	45	9	15	.......--	0	....	39
	& 81-89	779	4.4	PTGPKESKK	37	63	7	49	7	13	......---	0	....	37
	& 82-90	782	4.3	TGPKESKKK	35	65	7	52	6	12	.....----	0	....	35
	& 83-91	782	4.1	GPKESKKKV	38	62	7	50	5	11	....-----	0	....	38
	& 84-92	783	4.1	PKESKKKVE	37	63	7	50	5	10	...------	0	....	37
	& 85-93	785	4.8	KESKKKVER	28	72	6	59	7	14	..-------	0	....	28
	& 86-94	784	4.5	ESKKKVERE	35	65	8	50	7	14	.--------	0	....	35
	& 87-95	786	4.5	SKKKVERET	35	65	8	50	8	15	---------	0	....	35
	& 88-96	791	4.1	KKKVERETE	47	53	6	39	8	15	---------	0	....	47
	& 89-97	786	4.2	KKVERETET	46	54	6	39	9	17	---------	0	....	46
	& 90-98	785	4.5	KVERETETD	43	57	7	40	10	19	---------	0	....	43
	& 91-99	784	4.7	VERETETDP	41	59	6	40	13	22	---------	0	....	41
	+ 92-100	765	6.6	ERETETDPV	16	84	7	55	22	35	---------	0	....	16
	+ 93-101	758	7.5	RETETDPVH	6	94	4	60	30	45	---------	0	....	6
	94-102 *	66	5.3	ETETDSVHQ	6	94	6	39	48	67	---------	0	.....P..-	0
Rev	& 1-9	612	1.8	MAGRSGDSD	76	24	4	17	3	6	....	76	....	76
	& 2-10	603	2.2	AGRSGDSDE	70	30	4	23	3	7	....	70	........D	4
	& 3-11	602	3.3	GRSGDSDEE	48	52	18	30	4	10	....	48	.......DD	<1
	& 4-12	605	3.5	RSGDSDEEL	46	54	18	31	5	11	....	46	......DD.	<1
	& 5-13	605	3.8	SGDSDEELL	40	60	17	35	7	13	I	6	.....DD..	<1
	& 6-14	627	4.6	GDSDEELLK	31	69	14	43	12	19	.......IR	3	....DD...	<1
	& 7-15	624	5.1	DSDEELLKT	26	74	10	50	14	23	......IR.	2	...DD....	<1
	& 8-16	625	5.2	SDEELLKTV	23	77	10	54	13	23	.....IR..	2	..DD.....	<1
	& 9-17	629	5.0	DEELLKTVR	24	76	11	53	11	20	....IR...	2	.DD......	<1
	& 10-18	629	5.4	EELLKTVRL	23	77	10	51	16	25	...IR....	2	DD.......	<1
	& 11-19	636	5.2	ELLKTVRLI	25	75	10	50	14	23	..IR.....	2	D........	10
	& 12-20	636	4.6	LLKTVRLIK	36	64	9	43	12	20	.IR......	2	....	36
	+ 13-21	635	5.9	LKTVRLIKF	19	81	8	56	17	28	IR......L	1	L	8
	& 14-22	635	5.8	KTVRLIKFL	20	80	8	55	16	27	R......L.	3	.......L.	8
	& 15-23	633	5.1	TVRLIKFLY	21	79	13	54	12	20	......L..	13	......L..	13
	& 16-24	639	4.5	VRLIKFLYQ	28	72	15	49	8	15	.....L...	15	.....L...	15
	& 17-25	631	4.3	RLIKFLYQS	31	69	16	45	8	15	....L....	16	....L....	16
	& 18-26	616	4.2	LIKFLYQSN	32	68	15	46	7	13	...L.....	15	...L.....	15
	& 19-27	617	3.3	IKFLYQSNP	44	56	17	35	4	9	..L......	17	..L......	17
	& 20-28	615	4.1	KFLYQSNPP	36	64	14	43	7	13	.L.......	14	.L.......	14
	& 21-29	616	3.9	FLYQSNPPP	39	61	14	42	6	12	L........	14	L........	14
	& 22-30	616	3.3	LYQSNPPPS	41	59	20	34	5	10	........N	20	....	41
	& 23-31	612	4.2	YQSNPPPNP	22	78	19	52	7	13	....	22	.......SQ	5
	& 24-32	614	4.5	QSNPPPNPE	22	78	17	51	10	16	....	22	......SQ.	5
	& 25-33	615	4.5	SNPPPNPEG	22	78	17	51	10	16	....	22	.....SQ..	5
	+ 26-34	736	4.8	NPPPNPEGT	19	81	15	58	8	15	....	19	....SQ...	4
	+ 27-35	1384	4.2	PPPNPEGTR	20	80	18	58	5	9	....	20	...SQ....	14
	+ 28-36	1384	4.3	PPNPEGTRQ	20	80	18	58	4	9	....	20	..SQ.....	14
	& 29-37	1388	3.8	PSPEGTRQA	24	76	20	53	4	7	.N.......	20	..Q......	14
	& 30-38	1389	3.8	SPEGTRQAR	24	76	20	53	4	7	N........	20	.Q.......	14
	& 31-39	1391	2.9	PEGTRQARR	49	51	14	35	2	5	....	49	Q........	14
	& 32-40	1396	1.3	EGTRQARRN	83	17	5	11	2	3	....	83	....	83
	# 33-41	1396	0.7	GTRQARRNR	93	7	2	4	1	2	....	93	....	93
	# 34-42	1396	0.7	TRQARRNRR	92	8	2	5	1	2	....	92	....	92
	# 35-43	1396	0.8	RQARRNRRR	91	9	2	6	1	2	....	91	....	91
	# 36-44	1396	0.7	QARRNRRRR	92	8	2	5	1	2	....	92	....	92
	# 37-45	1395	0.6	ARRNRRRRW	93	7	2	4	1	2	....	93	....	93
	# 38-46	1396	0.5	RRNRRRRWR	95	5	1	3	1	2	....	95	....	95
	& 39-47	1396	1.8	RNRRRRWRE	70	30	11	18	1	3	....	70	....	70
	& 40-48	1395	1.8	NRRRRWRER	68	32	13	18	1	3	....	68	....	68
	& 41-49	1395	1.9	RRRRWRERQ	68	32	13	18	2	3	....	68	....	68
	& 42-50	1393	2.1	RRRWRERQR	66	34	12	20	2	4	....	66	....	66
	& 43-51	1394	3.0	RRWRERQRQ	53	47	11	33	3	6	....	53	....	53
	& 44-52	1395	3.0	RWRERQRQI	52	48	11	34	4	7	....	52	....	52
	& 45-53	1394	4.7	WRERQRQIR	30	70	9	56	5	10	........H	6	....	30
	+ 46-54	1391	6.2	RERQRQIRS	12	88	8	70	9	18	.......H.	4	....	12
	+ 47-55	1383	6.7	ERQRQIRSI	11	89	6	71	12	22	......H..	4	....	11

	[+] 48-56	1382	6.2	RQRQIRSIS	13	87	7	70	11	18	.....H...	6	....	13
	[+] 49-57	1382	7.1	QRQIRSISE	6	94	4	76	14	25	....H....	2	....	6
	[+] 50-58	1381	7.3	RQIRSISGW	4	96	3	78	15	27	...H...ER	2	.......ER	3
	[+] 51-59	1382	7.4	QIRSISGWI	3	97	3	79	14	27	..H...ER.	2	......ER.	3
	[+] 52-60	1381	7.3	IRTISGWIL	4	96	4	78	14	26	.HS..ER..	2	..S..ER..	4
	[+] 53-61	1374	7.7	QSISGWILS	3	97	3	76	18	31	H...ER..G	1	R...ER...	2
	[+] 54-62	1372	7.5	SISGWILSN	5	95	4	75	16	29	...ER..GT	<1	...ER...T	4
	[+] 55-63	1372	7.2	ISGWILSTY	6	94	4	76	14	26	..ER..G..	1	..ER.....	3
	[+] 56-64	1379	6.8	SGWILSTYL	8	92	4	76	12	22	.ER..G...	1	.ER......	4
	[+] 57-65	1380	6.8	GWILSTYLG	8	92	4	77	11	22	ER..G....	1	ER.......	4
	[+] 58-66	1382	6.1	WILSTHLGR	10	90	9	74	8	17	R..G.Y...	1	R....Y...	6
	[+] 59-67	1382	6.5	ILSTYLGRP	7	93	7	76	9	19	..G.....S	1	....	7
	[+] 60-68	1379	7.0	LSTYLGRSA	6	94	5	77	12	23	.G.......	1	.......P.	4
	[+] 61-69	1379	6.9	STYLGRSAE	6	94	4	78	12	23	G........	1	......P..	4
	[+] 62-70	1386	6.3	TYLGRSAEP	8	92	6	77	9	17	....	8	.....P...	5
	[+] 63-71	1386	5.5	YLGRPAEPV	11	89	10	73	6	13	....S....	9	....	11
	[&] 64-72	1389	4.2	LGRPAEPVP	26	74	18	51	5	9	...S.....	18	....	26
	[&] 65-73	1388	4.4	GRPAEPVPL	25	75	18	53	4	10	..S......	18	....	25
	[&] 66-74	1384	4.7	RPAEPVPLQ	23	77	19	52	6	12	.S.......	19	....	23
	[&] 67-75	1385	4.5	PAEPVPLQL	23	77	19	52	5	11	S........	19	....	23
	[&] 68-76	1385	3.7	AEPVPLQLP	43	57	16	37	4	8	....	43	....	43
	[&] 69-77	1387	2.5	EPVPLQLPP	68	32	4	25	3	6	....	68	....	68
	[&] 70-78	1385	2.6	PVPLQLPPL	65	35	5	26	3	6	....	65	....	65
	[&] 71-79	1385	2.7	VPLQLPPLE	64	36	5	27	3	6	....	64	....	64
	[&] 72-80	1386	2.5	PLQLPPLER	66	34	5	26	3	5	....	66	....	66
	[&] 73-81	1387	2.3	LQLPPLERL	68	32	5	25	2	4	....	68	....	68
	[&] 74-82	1387	2.2	QLPPLERLT	69	31	6	23	2	4	....	69	....	69
	[&] 75-83	1392	1.6	LPPLERLTL	76	24	7	15	1	3	....	76	....	76
	[&] 76-84	1388	2.0	PPLERLTLD	72	28	7	19	2	3	....	72	........A	<1
	[&] 77-85	1388	2.4	PLERLTLDC	67	33	7	24	2	5	....	67	.......A.	<1
	[&] 78-86	1386	3.4	LERLTLDCN	34	66	30	33	3	6	....	34	......A.S	<1
	[&] 79-87	1386	3.5	ERLTLDCNE	35	65	28	33	3	6	....	35	.....A.S.	<1
	[&] 80-88	1386	3.8	RLTLDCNED	34	66	26	36	4	8	....	34	....A.S..	<1
	[&] 81-89	1384	4.0	LTLDCNEDC	32	68	25	39	5	9	....	32	...A.S...	<1
	[&] 82-90	1384	4.1	TLDCNEDCG	32	68	24	40	5	9	....	32	..A.S....	<1
	[&] 83-91	1382	4.5	LDCNEDCGT	30	70	20	45	6	11	....	30	.A.S.....	<1
	[&] 84-92	1382	4.5	DCNEDCGTS	30	70	18	45	7	12	....	30	A.S......	<1
	[&] 85-93	1379	4.4	CNEDCGTSG	32	68	19	44	6	11	....	32	.S.......	19
	[&] 86-94	1379	4.1	NEDCGTSGT	32	68	21	41	6	10	....	32	S........	21
	[&] 87-95	1378	3.1	EDCGTSGTQ	59	41	8	29	4	8	....	59	....	59
	[&] 88-96	1382	2.8	DCGTSGTQG	63	37	8	25	4	8	....	63	....	63
	[&] 89-97	1380	2.5	CGTSGTQGV	68	32	9	19	4	7	....	68	....	68
	[&] 90-98	1381	2.3	GTSGTQGVG	72	28	9	17	3	6	....	72	....	72
	[&] 91-99	1380	3.6	TSGTQGVGS	45	55	13	38	4	8	....	45	........N	13
	[&] 92-100	1499	3.8	SGTQGVGSP	42	58	17	36	5	10	....	42	.......N.	17
	[&] 93-101	1492	3.9	GTQGVGSPQ	41	59	17	37	5	10	....	41	......N..	17
	[&] 94-102	1494	4.6	TQGVGSPQI	30	70	11	54	5	11	....	30	.....N...	11
	[&] 95-103	1494	4.9	QGVGSPQIL	26	74	11	56	6	12	....	26	....N....	10
	[&] 96-104	1496	5.2	GVGSPQILV	24	76	10	60	6	13	....	24	...N....L	<1
	[&] 97-105	1497	5.0	VGSPQILVE	25	75	10	60	6	12	....	25	..N....L.	<1
	[&] 98-106	1498	5.2	GSPQILVES	24	76	10	59	6	13	....	24	.N....L..	<1
	[&] 99-107	1497	5.3	SPQILVESP	25	75	10	57	7	14	....	25	N....L...	<1
	[&] 100-108	1492	5.0	PQILVESPT	21	79	17	55	7	12	....	21	....L....	2
	[+] 101-109	1492	5.1	QILVESPTV	19	81	14	61	6	12	....	19	...L....I	<1
	[+] 102-110	1498	5.0	ILVESPTVL	19	81	14	61	6	11	....	19	..L....I.	<1
	[&] 103-111	1499	4.8	LVESPAVLE	23	77	19	52	6	11	.....T...	19	.L...TI..	<1
	[+] 104-112	1496	5.2	VESPAVLES	20	80	18	56	7	13	....T....	18	L...TI...	<1
	[&] 105-113	1494	4.8	ESPAVLESG	22	78	20	52	6	11	...T.....	20	...TI....	4
	[&] 106-114	1493	5.2	SPAVLESGT	20	80	17	56	6	13	..T......	17	..TI....A	<1
	[&] 107-115	1492	5.2	PAVLESGTK	20	80	17	56	7	13	.T.......	17	.TI....A.	<1
	[&] 108-116	1494	5.2	AVLESGTKE	20	80	18	54	7	13	T........	18	TI....A..	<1
Vpu	[+] 1-9	1037	7.8	MQSLQILAI	7	93	3	59	31	45	..PIP.V..	<1	..P.V.YS.	0
	[+] 2-10	1037	8.0	QSLQILAIV	7	93	3	57	34	48	.PIP.V...	<1	.P.V.YS..	0
	[+] 3-11	1049	7.6	SLQILAIVA	8	92	3	63	27	41	PIP.V....	<1	P.V.YS...	0
	[+] 4-12	1064	6.9	LQILAIVAL	11	89	5	64	20	31	IP.V.....	<1	.V.YS....	0
	[+] 5-13	1068	6.8	QILAIVALV	13	87	5	62	20	31	P.V......	<1	V.YS.....	0
	[&] 6-14	1096	5.3	ILAIVALVV	30	70	8	49	12	20	.V.......	8	.YS......	<1
	[&] 7-15	1093	5.7	LAIVALVVA	25	75	7	58	11	20	V........	6	YS......V	<1
	[+] 8-16	1118	5.6	AIVALVVAA	19	81	10	60	10	18	I	7	S......V.	2
	[&] 9-17	1123	5.2	IVALVVAAI	22	78	12	58	8	16	.......I.	8	......V..	12
	[+] 10-18	1123	5.5	VALVVAAII	17	83	12	61	10	18	......I..	6	.....V...	12

	[+] 11-19	1125	5.2	ALVVAAIIA	18	82	13	61	8	15	.....I...	6	....V....	13
	[+] 12-20	1124	5.1	LVVAAIIAI	19	81	13	61	7	14	....I....	6	...V.....	13
	[+] 13-21	1120	5.4	VVAAIIAIV	16	84	12	64	8	16	...I.....	6	..V......	12
	[+] 14-22	1122	5.2	VAAIIAIVV	17	83	12	63	7	14	..I......	6	.V.......	12
	[+] 15-23	1125	4.9	AAIIAIVVW	19	81	13	62	6	12	.I.......	6	V........	13
	[+] 16-24	1131	4.9	AIIAIVVWS	18	82	15	61	6	12	I........	6	....	18
	[&] 17-25	1146	3.3	IIAIVVWSI	40	60	20	37	3	7	....	40	....	40
	[&] 18-26	1144	3.3	IAIVVWSIV	40	60	20	36	3	7	....	40	....	40
	[&] 19-27	1140	4.1	AIVVWSIVF	24	76	21	50	5	9	I	3	....	24
	[&] 20-28	1141	4.2	IVVWSIVFI	24	76	21	49	6	10	.......I.	3	....	24
	[&] 21-29	1142	4.2	VVWSIVFIE	24	76	21	49	6	10	......I..	3	....	24
	[&] 22-30	1147	3.7	VWSIVFIEY	28	72	23	44	5	8	.....I...	3	....	28
	[&] 23-31	1146	4.0	WSIVFIEYR	27	73	22	45	5	9	....I....	4	....	27
	[&] 24-32	1142	4.3	SIVFIEYRK	25	75	21	48	6	11	...I.....	3	....	25
	[&] 25-33	1141	3.7	IVFIEYRKI	33	67	28	33	6	10	..I......	5	....	33
	[&] 26-34	1141	4.0	VFIEYRKIL	30	70	28	35	7	11	.I.......	5	....	30
	[&] 27-35	1140	4.3	FIEYRKILR	29	71	24	38	9	13	I........	5	....	29
	[&] 28-36	1148	2.8	IEYRKILRQ	63	37	6	26	5	8	....	63	....	63
	[&] 29-37	1145	2.9	EYRKILRQR	61	39	6	27	6	9	....	61	....	61
	[&] 30-38	1143	3.3	YRKILRQRK	56	44	5	32	7	11	....	56	....	56
	[&] 31-39	1144	3.4	RKILRQRKI	55	45	5	33	7	10	....	55	....	55
	[&] 32-40	1140	3.2	KILRQRKID	56	44	6	32	6	10	....	56	....	56
	[&] 33-41	1144	3.2	ILRQRKIDR	57	43	6	31	6	10	....	57	........K	3
	[&] 34-42	1142	3.3	LRQRKIDRL	55	45	6	33	6	10	....	55	.......K.	2
	[&] 35-43	1142	3.3	RQRKIDRLI	53	47	6	36	5	9	....	53	......K..	2
	[&] 36-44	1140	3.9	QRKIDRLID	45	55	8	38	8	12	....	45	.....K...	2
	[&] 37-45	1141	3.9	RKIDRLIDR	45	55	8	38	9	13	....	45	....K....	2
	[&] 38-46	1145	3.7	KIDRLIDRI	47	53	8	37	8	12	L	4	...K.....	2
	[&] 39-47	1140	4.6	IDRLIDRIR	39	61	5	46	9	15	LI	4	..K......	1
	[&] 40-48	1139	4.9	DRLIDRIRE	36	64	5	48	11	17	......LI.	4	.K.......	1
	[&] 41-49	1140	4.7	RLIDRIRER	37	63	6	47	10	16	.....LI..	4	K........	1
	[&] 42-50	1140	4.7	LIDRIRERA	37	63	5	48	9	15	....LI...	4	....	37
	[&] 43-51	1143	4.3	IDRIRERAE	40	60	7	46	8	13	...LI....	4	....	40
	[&] 44-52	1147	4.0	DRIRERAED	43	57	8	44	6	10	..LI.....	4	....	43
	[&] 45-53	1151	3.1	RIRERAEDS	49	51	10	37	4	6	.LI......	5	....	49
	[&] 46-54	1153	2.9	IRERAEDSG	51	49	10	36	3	6	LI.......	4	....	51
	[&] 47-55	1152	2.7	RERAEDSGN	51	49	15	31	3	5	I........	15	....	51
	[&] 48-56	1158	1.1	ERAEDSGNE	86	14	5	8	2	3	....	86	........D	1
	[#] 49-57	1161	0.7	RAEDSGNES	92	8	1	5	1	2	....	92	.......D.	1
	[&] 50-58	1159	1.5	AEDSGNESE	72	28	20	6	2	3	....	72	......D..	<1
	[&] 51-59	1158	1.3	EDSGNESEG	73	27	21	3	2	3	....	73	.....D...	<1
	[&] 52-60	1132	1.4	DSGNESEGD	72	28	21	5	2	3	........-	0	....D....	<1
	[&] 53-61	1130	2.6	SGNESEGDQ	52	48	18	27	3	5	.......--	0	...D.....	<1
	[&] 54-62	1131	2.7	GNESEGDQE	51	49	17	28	4	6	......---	0	..D......	<1
	[&] 55-63	1122	2.9	NESEGDQEE	49	51	17	30	5	7	.....---.	0	.D.......	<1
	[&] 56-64	1169	3.0	ESEGDQEEL	48	52	17	30	5	8	....---.I	0	D........	<1
	[&] 57-65	1149	3.5	SEGDQEELS	42	58	17	35	6	9	...---.I.	0	....	42
	[&] 58-66	1141	4.2	EGDQEELSA	37	63	15	40	8	13	..---.I..	0	....	37
	[&] 59-67	1146	3.9	GDQEELSAL	50	50	7	36	7	13	.---.I...	0	....	50
	[&] 60-68	1115	4.2	DQEELSALV	45	55	7	39	10	16	---.I....	0	....	45
	[&] 61-69	1107	4.7	QEELSALVE	41	59	6	43	11	17	--.I.....	0	....	41
	[&] 62-70	1107	4.9	EELSALVEM	35	65	9	46	11	17	-.I......	0	........R	9
	[&] 63-71	1129	4.8	ELSALVEMG	35	65	9	46	10	17	.I.......	1	.......R.	9
	64-72 *	63	3.8	VSALVEMGV	24	76	13	40	24	37	I........	11	L.....R.-	0
	65-73 *	63	3.4	SALVEMGVE	37	63	13	25	25	35	....	37	.....R.--	0
	66-74 *	63	3.3	ALVEMGVEM	38	62	13	25	24	33	....	38	....R.---	0
	67-75 *	63	3.3	LVEMGVEMG	38	62	13	29	21	32	....	38	...R.----	0
	68-76 *	63	3.4	VEMGVEMGH	37	63	13	29	22	33	....	37	..R.----.	0
	69-77 *	63	3.3	EMGVEMGHH	37	63	13	29	22	32	....	37	.R.----.L	0
	70-78 *	63	3.3	MGVEMGHHA	37	63	13	29	22	32	....	37	R.----.L.	0
	71-79 *	63	2.5	GVEMGHHAP	41	59	33	8	17	22	....	41	.----.L..	0
	72-80 *	64	2.4	VEMGHHAPW	42	58	34	8	16	20	....	42	----.L...	0
	73-81 *	68	2.1	EMGHHAPWD	57	43	21	10	12	18	....	57	---.L....	0
	74-82 *	68	2.7	MGHHAPWDI	31	69	28	28	13	19	V	28	--.L.....	0
	75-83 *	70	3.1	GHHAPWDID	29	71	23	33	16	24	.......V.	23	-.L......	0
	[+] 76-84	1163	6.0	HHAPWDVDD	11	89	8	67	15	22	....	11	.L....I..	5
	[+] 77-85	1169	6.1	HAPWDVDDL	11	89	7	69	14	21	....	11	L....I...	5
Env	[&] 1-9	246	4.2	MKVMETRRN	41	59	6	35	19	28	.R.K.K---	0	.T.KGI.K.	0
	[&] 2-10	243	4.3	KVMETRRNY	41	59	6	33	21	30	R.K.K---.	0	T.KGI.K..	0
	[&] 3-11	253	4.3	VMETRRNYQ	40	60	6	32	22	31	.K.K---..	0	.KGI.K...	5
	[&] 4-12	255	4.4	METRRNYQH	41	59	5	28	25	33	K.K---...	0	KGI.K....	4

[&] 5-13	243	4.2	ETRRNYQHL	43	57	6	30	21	29	.K---....	0	GI.K.....	3
[&] 6-14	241	3.9	TRRNYQHLW	46	54	6	29	18	26	K---.....	0	I.K......	4
[&] 7-15	239	3.8	RRNYQHLWR	49	51	5	28	18	26	---......	0	.K.......	3
[&] 8-16	237	3.5	RNYQHLWRW	52	48	5	26	16	24	--.......	0	K.......G	3
[&] 9-17	241	3.3	NYQHLWRWG	55	45	5	27	14	21	-........	0	.......G.	2
[&] 10-18	258	3.5	YQHLWRWGT	53	47	5	28	15	22	....	53	......G.I	0
[&] 11-19	258	3.3	QHLWRWGTM	55	45	5	27	14	21	....	55	.....G.IL	0
[&] 12-20	258	3.5	HLWRWGTML	53	47	5	29	13	22	....	53	....G.IL.	0
[&] 13-21	266	3.5	LWRWGTMLL	51	49	5	33	11	20	....	51	...G.IL..	0
[&] 14-22	267	3.3	WRWGTMLLG	52	48	6	31	10	18	....	52	..G.IL...	<1
[&] 15-23	269	3.8	RWGTMLLGM	42	58	9	36	12	20	....	42	.G.IL...I	<1
[&] 16-24	270	3.6	WGTMLLGML	41	59	10	40	9	16	....	41	G.IL...I.	<1
[&] 17-25	275	3.7	GTMLLGMLM	40	60	9	42	9	17	....	40	.IL...I..	<1
[&] 18-26	274	3.8	TMLLGMLMI	41	59	8	41	11	19	....	41	IL...I...	<1
[&] 19-27	285	3.4	MLLGMLMIC	47	53	11	31	11	17	....	47	L...I....	2
[&] 20-28	285	3.6	LLGMLMICK	39	61	12	38	12	18	........S	11	...I....S	12
[&] 21-29	285	3.7	LGMLMICKA	40	60	10	38	12	19	.......S.	10	..I....S.	10
[&] 22-30	284	4.4	GMLMICKAA	35	65	7	42	16	25	......S.T	2	.I....S.T	7
[&] 23-31	283	4.8	MLMICKAAE	28	72	7	48	17	28	.....S.T.	2	I....S.T.	6
[&] 24-32	279	5.0	LMICKAAEN	23	77	9	49	19	29	....S.T.K	9	....S.T.Q	<1
[&] 25-33	278	5.0	MICKAAENL	24	76	8	49	19	30	...S.T.K.	8	...S.T.Q.	<1
[&] 26-34	278	4.9	ICKAAENLW	24	76	9	49	18	28	..S.T.K..	9	..S.T.Q..	<1
[&] 27-35	279	4.8	CKAAENLWV	24	76	9	52	15	26	.S.T.K...	9	.S.T.Q...	<1
[&] 28-36	279	4.8	KAAENLWVT	24	76	9	54	13	25	S.T.K....	9	S.T.Q....	<1
[&] 29-37	279	4.4	AAENLWVTV	27	73	10	52	11	20	.T.K.....	10	.T.Q.....	1
[&] 30-38	279	4.4	AENLWVTVY	27	73	10	53	10	20	T.K......	10	T.Q......	1
[&] 31-39	278	3.5	ENLWVTVYY	31	69	17	43	9	13	.K.......	17	.Q.......	4
[&] 32-40	278	2.6	NLWVTVYYG	45	55	26	23	6	9	K........	26	Q........	6
[&] 33-41	282	1.1	LWVTVYYGV	84	16	8	3	4	6	....	84	....	84
[#] 34-42	284	0.5	WVTVYYGVP	94	6	1	3	2	4	....	94	....	94
[#] 35-43	284	0.5	VTVYYGVPV	95	5	<1	2	2	4	....	95	....	95
[#] 36-44	284	0.4	TVYYGVPVW	96	4	<1	<1	3	4	....	96	....	96
[#] 37-45	284	0.5	VYYGVPVWK	94	6	2	1	3	4	....	94	....	94
[&] 38-46	284	0.8	YYGVPVWKE	88	12	7	3	3	4	....	88	....	88
[&] 39-47	284	0.9	YGVPVWKEA	87	13	7	4	3	5	....	87	....	87
[&] 40-48	284	1.0	GVPVWKEAT	86	14	6	5	4	6	....	86	....	86
[&] 41-49	286	1.0	VPVWKEATT	86	14	6	5	4	6	....	86	....	86
[&] 42-50	286	1.0	PVWKEATTT	86	14	6	4	4	6	....	86	....	86
[&] 43-51	286	1.1	VWKEATTTL	86	14	6	4	5	6	....	86	....	86
[&] 44-52	286	1.1	WKEATTTLF	85	15	6	5	5	7	....	85	....	85
[&] 45-53	285	1.1	KEATTTLFC	85	15	6	5	4	6	....	85	....	85
[&] 46-54	241	1.0	EATTTLFCA	85	15	7	5	3	5	....	85	....	85
[#] 47-55	241	0.6	ATTTLFCAS	92	8	2	2	3	5	....	92	....	92
[&] 48-56	241	0.9	TTTLFCASD	87	13	6	4	3	5	....	87	....	87
[#] 49-57	242	0.6	TTLFCASDA	91	9	6	<1	2	3	....	91	....	91
[#] 50-58	242	0.7	TLFCASDAK	90	10	6	2	2	4	....	90	........R	<1
[&] 51-59	242	1.2	LFCASDAKA	80	20	8	10	2	5	....	80	.......R.	<1
[&] 52-60	241	1.6	FCASDAKAY	75	25	8	13	4	7	....	75	......R..	<1
[&] 53-61	238	2.9	CASDAKAYD	45	55	21	29	6	12	....	45	.....R..K	0
[&] 54-62	238	3.7	ASDAKAYDT	41	59	14	37	8	18	....	41	....R..K.	0
[&] 55-63	237	3.7	SDAKAYDTE	41	59	14	37	8	18	....	41	...R..K..	0
[&] 56-64	237	4.4	DAKAYDTEA	22	78	19	48	12	22	V	19	..R..K..V	0
[&] 57-65	235	4.4	AKAYDTEAH	22	78	18	47	13	23	.......V.	18	.R..K..V.	0
[&] 58-66	235	4.5	KAYDTEAHN	22	78	18	46	14	24	......V..	18	R..K..V..	0
[&] 59-67	234	4.4	AYDTEAHNV	21	79	20	46	13	24	.....V...	20	..K..V...	2
[&] 60-68	233	4.0	YDTEVHNVW	22	78	21	46	11	20	....	22	.K.......	2
[&] 61-69	232	3.9	DTEVHNVWA	23	77	22	47	9	18	....	23	K........	2
[&] 62-70	234	2.7	TEVHNVWAT	41	59	28	24	6	10	....	41	....	41
[&] 63-71	235	1.8	EVHNVWATH	57	43	30	7	6	7	....	57	....	57
[&] 64-72	235	1.8	VHNVWATHA	57	43	29	7	6	7	....	57	....	57
[&] 65-73	235	0.8	HNVWATHAC	89	11	6	0	6	6	....	89	....	89
[#] 66-74	272	0.7	NVWATHACV	91	9	5	0	4	4	....	91	....	91
[#] 67-75	272	0.6	VWATHACVP	92	8	5	0	3	4	....	92	....	92
[#] 68-76	287	0.4	WATHACVPT	96	4	1	0	3	3	....	96	....	96
[&] 69-77	606	0.8	ATHACVPTD	86	14	12	1	1	2	....	86	....	86
[&] 70-78	607	0.8	THACVPTDP	85	15	12	2	1	2	....	85	....	85
[&] 71-79	607	2.1	HACVPTDPN	51	49	25	23	2	3	....	51	........S	25
[&] 72-80	607	2.1	ACVPTDPNP	51	49	25	22	2	3	....	51	.......S.	25
[&] 73-81	606	2.4	CVPTDPNPQ	48	52	24	26	2	4	....	48	......S..	24
[&] 74-82	606	2.4	VPTDPNPQE	48	52	25	26	2	4	....	48	.....S...	25
[&] 75-83	604	2.8	PTDPNPQEV	41	59	24	32	3	6	....	41	....S...I	<1

[&] 76-84	601	3.4	TDPNPQEVV	39	61	17	39	6	10	....	39	...S...IP	<1
[&] 77-85	601	3.7	DPNPQEVVL	32	68	17	45	6	10	....	32	..S...IP.	<1
[&] 78-86	600	4.0	PNPQEVVLE	21	79	17	53	9	13	V	1	.S...IP.I	0
[&] 79-87	600	4.0	NPQEVVLEN	21	79	17	53	9	13	.......V.	1	S...IP.I.	0
[&] 80-88	600	3.5	PQEVVLENV	39	61	11	43	8	11	......V..	1	...IP.I..	0
[&] 81-89	624	3.6	QEVVLENVT	37	63	10	46	7	11	.....V...	1	..IP.I...	0
[&] 82-90	625	3.5	EVVLENVTE	37	63	12	43	7	11	....V....	1	.IP.I....	0
[&] 83-91	625	4.1	VVLENVTEN	27	73	12	52	9	14	...V.....	1	IP.I.....	0
[&] 84-92	626	3.9	VLENVTENF	32	68	12	48	7	12	..V......	1	P.I......	0
[&] 85-93	634	3.8	LENVTENFN	25	75	17	52	6	10	.V.......	2	.I.......	0
[&] 86-94	634	3.6	GNVTENFNM	25	75	25	44	6	10	V........	2	I........	0
[&] 87-95	635	2.5	NVTENFNMW	56	44	15	24	5	7	....	56	....	56
[&] 88-96	622	2.9	VTENFNMWK	54	46	9	33	5	8	....	54	........E	1
[&] 89-97	622	3.0	TENFNMWKN	53	47	9	32	6	9	....	53	.......E.	1
[&] 90-98	623	3.5	ENFNMWKNN	46	54	9	38	8	12	........D	5	......E.K	0
[&] 91-99	699	3.7	NFNMWKNNM	41	59	8	43	8	11	.......D.	5	.....E.K.	0
[&] 92-100	695	3.1	FNMWKNNMV	44	56	13	37	6	8	......D..	13	....E.K..	0
[&] 93-101	695	3.3	NMWKNNMVE	43	57	10	41	6	9	.....D...	10	...E.K..D	0
[&] 94-102	695	2.9	MWKNNMVEQ	53	47	10	33	4	8	....D....	10	..E.K..D.	0
[&] 95-103	735	2.7	WKNNMVEQM	52	48	19	25	4	7	...D.....	19	.E.K..D..	0
[&] 96-104	769	3.0	KNNMVEQMH	45	55	18	32	4	8	..D......	18	E.K..D...	0
[&] 97-105	771	2.4	NNMVEQMHE	55	45	19	21	4	7	.D.......	19	.K..D....	0
[&] 98-106	770	2.4	NMVEQMHED	55	45	20	21	4	7	D........	20	K..D.....	0
[&] 99-107	768	1.7	MVEQMHEDI	76	24	10	11	3	5	....	76	..D......	2
[&] 100-108	768	1.7	VEQMHEDII	76	24	10	11	3	5	....	76	.D.......	2
[&] 101-109	767	1.8	EQMHEDIIS	74	26	10	12	3	5	....	74	D........	2
[&] 102-110	758	1.4	QMHEDIISL	78	22	11	8	3	4	....	78	....	78
[&] 103-111	757	1.4	MHEDIISLW	78	22	11	8	3	4	....	78	....	78
[&] 104-112	754	1.5	HEDIISLWD	76	24	12	10	2	4	....	76	....	76
[&] 105-113	770	1.5	EDIISLWDQ	75	25	13	10	2	4	....	75	....	75
[&] 106-114	770	1.4	DIISLWDQS	77	23	13	8	2	3	....	77	....	77
[&] 107-115	768	1.3	IISLWDQSL	77	23	13	8	2	3	....	77	....	77
[&] 108-116	786	1.3	ISLWDQSLK	78	22	13	6	2	3	....	78	....	78
[&] 109-117	795	1.6	SLWDQSLKP	71	29	13	14	2	4	....	71	....	71
[&] 110-118	877	1.4	LWDQSLKPC	76	24	11	11	2	3	....	76	....	76
[&] 111-119	893	1.4	WDQSLKPCV	76	24	11	11	2	3	....	76	....	76
[&] 112-120	892	1.7	DQSLKPCVK	71	29	11	16	2	3	....	71	....	71
[&] 113-121	1029	1.5	QSLKPCVKL	74	26	11	13	2	3	....	74	....	74
[&] 114-122	1032	1.0	SLKPCVKLT	86	14	6	7	1	2	....	86	....	86
[&] 115-123	1034	1.0	LKPCVKLTP	86	14	6	7	1	2	....	86	....	86
[&] 116-124	1066	1.1	KPCVKLTPL	85	15	6	8	2	3	....	85	....	85
[&] 117-125	1517	0.8	PCVKLTPLC	89	11	4	6	1	2	....	89	....	89
[&] 118-126	1568	0.8	CVKLTPLCV	89	11	5	5	1	2	....	89	....	89
[&] 119-127	1594	1.1	VKLTPLCVT	86	14	5	7	1	2	........S	1	....	86
[&] 120-128	2665	1.0	KLTPLCVTL	88	12	4	7	1	2	.......S.	1	....	88
[&] 121-129	2670	1.7	LTPLCVTLN	76	24	5	17	1	2	......S.K	<1	....	76
[&] 122-130	3112	1.9	TPLCVTLNC	74	26	5	20	1	2	.....S.K.	1	....	74
[&] 123-131	3326	2.6	PLCVTLNCT	66	34	4	28	2	4	....S.K..	1	I	2
[&] 124-132	3368	4.1	LCVTLNCTD	44	56	13	40	3	6	...S.K...	1	.......I.	1
[+] 125-133	3673	6.2	CVTLNCTDN	12	88	10	72	6	11	..S.K...L	1	......I.V	<1
[+] 126-134	3675	7.4	VTLNCTDNW	10	90	3	79	8	15	.S.K...LK	1	.....I.VN	<1
[+] 127-135	3677	7.8	TLNCTDNWN	9	91	3	78	9	19	S.K...LK.	1	....I.VNI	0
[+] 128-136	3719	8.3	LNCTDNWNN	6	94	2	81	11	22	.K...LK.D	1	...I.VNIT	0
[+] 129-137	3725	8.3	NCTDNWNNT	6	94	2	79	12	24	K...LK.D.	<1	..I.VNITN	0
[+] 130-138	3912	8.6	CTDNWNNTG	6	94	2	79	13	25	...LK.D.N	1	.I.VNITNT	0
[+] 131-139	3917	8.7	TDNWNNTGN	5	95	2	79	14	27	..LK.D.NT	1	I.VNITNT.	0
[+] 132-140	3911	8.9	DNWNNTGNV	5	95	2	78	15	28	.LK.D.NTN	<1	.VNITNT.S	0
[+] 133-141	3863	8.8	NWNNTGNVS	5	95	2	77	16	29	LK.D.NTN.	<1	VNITNT.ST	0
[+] 134-142	3838	9.0	WNNTGNVSD	5	95	2	77	16	30	K.D.NTN.S	<1	NITNT.STN	0
[+] 135-143	3807	9.0	NNTGNVSDS	5	95	2	76	17	31	.D.NTN.S.	1	ITNT.STNP	0
[+] 136-144	3747	9.0	NTGNVSDSS	5	95	2	76	18	32	D.NTN.S.G	1	TNT.STNPT	0
[+] 137-145	3701	9.1	TGNVSDSSW	5	95	2	75	18	32	.NTN.S.GR	1	NT.STNPTS	0
[+] 138-146	3473	8.9	GNVSDSSWK	4	96	1	76	19	34	NTN.S.GRM	1	T.STNPTSS	0
[+] 139-147	3090	9.2	TSVNSNSSG	2	98	1	78	19	35	.NSS.GRMI	1	N.T.PT..W	0
[+] 140-148	2699	9.0	SVNSNSSGG	2	98	1	77	20	36	NSS.GRMIM	1	.T.PT..WE	0
[+] 141-149	2334	8.8	NSTGGTVGG	2	98	1	76	21	37	S.S.RMIM-	0	TNPTSSWET	0
[+] 142-150	2015	8.7	SSGEEIEGK	2	98	1	75	22	38	...RM.M--	0	NPTSSW.TM	0
[+] 143-151	1735	8.6	SGEEIEGKI	2	98	2	73	23	39	..RM.M---	0	PTSSW.TM-	0
[+] 144-152	1566	8.5	TNVTNATSS	2	98	2	73	22	38	GRMIM----	0	.SSWETM--	0
[+] 145-153	1322	8.3	EEIEGKINQ	2	98	2	73	23	39	RM.M-----	0	SSW.TM---	0
[+] 146-154	1122	8.1	DLLSTNSTT	2	98	2	73	22	39	MIM------	0	SWETM----	0

[+] 147-155	936	7.8	LLSTNSTTN	3	97	2	72	23	38	IM-------	0	WETM-----	0
[+] 148-156	811	7.5	LSTNSTTNS	3	97	3	73	21	37	M--------	0	ETM------	0
[+] 149-157	657	7.2	STNSTTNSS	5	95	4	69	23	37	---------	0	TM-------	0
[+] 150-158	500	6.7	TNSTTNSSG	6	94	5	65	24	38	---------	0	M--------	0
[+] 151-159	463	6.4	NSTTNSSGE	7	93	5	67	22	35	---------	0	---------	0
[+] 152-160	405	6.2	STTNSSGEN	8	92	6	65	21	34	---------	0	---------	0
[+] 153-161	317	5.7	TTNSSGENR	10	90	7	62	21	32	---------	0	---------	0
[+] 154-162	269	5.3	TNSSGENRM	12	88	9	58	21	31	---------	0	---------	0
[+] 155-163	196	4.3	NSSGENRME	17	83	13	54	16	24	--------.	0	--------.	0
[+] 156-164	166	3.8	SSGENRMER	19	81	15	52	14	20	-------.K	0	-------.K	0
[+] 157-165	161	3.5	SGENRMERG	20	80	16	52	12	18	------.K.	0	------.K.	0
[+] 158-166	161	3.5	GENRMERGD	20	80	16	53	11	17	-----.K.E	0	-----.K.E	0
[+] 159-167	161	3.5	ENRMERGDI	20	80	16	53	11	17	----.K.E.	0	----.K.E.	0
[+] 160-168	161	3.5	NRMERGDIK	20	80	16	53	11	17	---.K.E..	0	---.K.E..	0
[+] 161-169	161	3.5	RMERGDIKN	20	80	16	55	9	16	--.K.E...	0	--.K.E...	0
[&] 162-170	160	3.3	MERGDIKNC	20	80	16	58	6	13	-.K.E....	0	-.K.E....	0
[&] 163-171	1262	3.1	EKGEIKNCS	42	58	20	37	2	4	....	42	....	42
[&] 164-172	1530	3.1	KGEIKNCSF	41	59	20	37	2	4	....	41	....	41
[&] 165-173	2641	2.9	GEIKNCSFN	51	49	19	28	2	4	....	51	....	51
[&] 166-174	3721	4.2	EIKNCSFNI	31	69	13	54	2	5	....	31	....	31
[&] 167-175	3944	4.3	IKNCSFNIT	28	72	17	52	3	6	........S	2	....	28
[&] 168-176	3939	3.6	KNCSFNITT	47	53	12	38	2	5	.......S.	2	....	47
[&] 169-177	3938	5.0	NCSFNITTS	23	77	15	59	3	7	......S..	1	........N	15
[+] 170-178	3959	5.8	CSFNITTSI	17	83	10	69	4	9	.....S...	1	.......N.	10
[+] 171-179	3945	6.3	SFNITTSIR	14	86	8	72	5	11	....S....	1	......N..	8
[+] 172-180	3943	6.7	FNITTSIRD	13	87	8	74	6	12	...S....G	1	.....N...	8
[+] 173-181	3942	6.8	NITTSIRDK	13	87	7	73	6	13	..S....G.	1	....N....	7
[+] 174-182	3933	7.3	ITTSIRDKV	10	90	7	75	8	16	.S....G..	1	...N....I	<1
[+] 175-183	3925	7.1	TTNIRDKVQ	12	88	8	72	8	16	S.S..G...	1	.......I.	<1
[+] 176-184	3905	7.1	TNIRDKVQK	12	88	8	72	9	17	.S..G....	1	......I..	<1
[+] 177-185	3906	7.4	NIRDKVQKE	12	88	7	72	9	18	S..G.....	1	.....I...	<1
[+] 178-186	3904	6.8	IRDKVQKEY	20	80	4	68	8	15	..G......	1	....I....	<1
[&] 179-187	3907	6.2	RDKVQKEYA	23	77	5	65	7	13	.G.......	1	...I.....	<1
[+] 180-188	3922	6.4	DKVQKEYAL	20	80	5	69	6	12	G.......F	1	..I......	<1
[&] 181-189	3924	6.2	KVQKEYALF	21	79	5	68	6	12	.......F.	3	.I.......	<1
[&] 182-190	3926	6.1	VQKEYALFY	23	77	5	66	6	11	......F..	3	I.......N	<1
[+] 183-191	3935	6.0	QKEYALFYK	20	80	6	69	5	10	.....F...	2	.......NR	<1
[&] 184-192	3942	5.9	KEYALFYKL	22	78	7	66	5	10	....F....	3	......NR.	<1
[&] 185-193	3958	5.4	EYALFYKLD	27	73	7	62	4	8	...F.....	3	.....NR..	<1
[&] 186-194	3956	5.3	YALFYKLDV	25	75	8	63	4	8	..F.....I	1	....NR...	<1
[&] 187-195	3954	5.4	ALFYKLDVV	22	78	8	67	4	8	.F.....II	1	...NR....	<1
[+] 188-196	3936	5.9	LFYKLDVVP	18	82	7	69	5	10	F.....II.	1	..NR.....	<1
[+] 189-197	3935	5.8	FYKLDVVPI	18	82	7	70	5	9	.....II..	1	.NR......	<1
[+] 190-198	3934	6.5	YKLDVVPID	12	88	7	75	6	12	....II...	1	NR......N	0
[+] 191-199	3933	7.2	KLDVVPIDN	9	91	6	78	7	15	...II....	1	R......N.	<1
[+] 192-200	3901	7.4	LDVVPIDND	9	91	7	76	8	17	..II.....	1	......N.S	0
[+] 193-201	3901	7.8	DVVPIDNDN	8	92	5	77	10	19	.II.....T	1	.....N.S.	0
[+] 194-202	3659	8.2	VVPIGENEN	5	95	5	78	12	23	II..DNDTT	1	....NNSNT	0
[+] 195-203	3600	8.0	VPIDNDNTS	6	94	5	76	13	24	I.....T..	1	...N.S..Y	0
[+] 196-204	2463	8.1	PIGENENNS	7	93	4	74	15	28	..DNDTTS-	0	..NNSNTY-	0
[+] 197-205	1806	8.1	IDNEDNAGN	5	95	3	74	18	32	...DTTS--	0	.N.SNTY--	0
[+] 198-206	1412	7.8	DNEDNAGNN	6	94	2	73	18	32	..DTTS---	0	N.SNTY---	0
[+] 199-207	1138	7.3	NEDNAGNNT	8	92	3	72	18	31	.DTTS----	0	.SNTY----	0
[+] 200-208	953	7.2	EDNAGNNTN	8	92	3	70	18	32	DTTS-----	0	SNTY-----	0
[+] 201-209	716	6.7	DNAGNNTNN	14	86	5	60	22	34	TTS------	0	NTY------	0
[&] 202-210	490	6.0	NAGNNTNNT	21	79	5	52	22	35	TS-------	0	TY-------	0
[&] 203-211	369	5.3	AGNNTNNTS	29	71	4	47	20	32	S--------	0	Y--------	0
[+] 204-212	192	5.7	AKNNNTNTT	7	93	6	62	25	41	---------	0	---------	0
[+] 205-213	168	5.6	SYNSSYSNY	7	93	6	61	26	43	--------.	0	--------.	0
[+] 206-214	168	5.6	YNSSYSNYR	7	93	6	60	27	44	-------.K	0	-------..	0
[+] 207-215	168	5.6	NSSYSNYRL	8	92	7	61	25	43	------.K.	0	------...	0
[+] 208-216	168	5.6	SSYSNYRLI	8	92	7	58	27	44	-----.K.T	0	-----....	0
[+] 209-217	168	5.6	SYSNYRLIN	8	92	7	58	28	44	----.K.TS	0	----....S	0
[+] 210-218	168	5.5	YSNYRLINC	8	92	7	55	30	43	---.K.TS.	0	---....S.	0
[+] 211-219	167	5.3	SNYRLINCN	8	92	8	55	29	40	--.K.TS..	0	--....S..	0
[+] 212-220	167	4.9	NYRLISCNT	16	84	8	53	23	34	-.K.T....	0	-........	0
[&] 213-221	3760	3.5	YRLISCNTS	41	59	24	31	3	5	.K.T.....	2	....	41
[&] 214-222	3894	4.3	RLISCNTSV	36	64	19	41	4	7	K.T......	<1	....	36
[&] 215-223	3880	4.2	LISCNTSVI	34	66	22	41	4	6	.T.......	2	L	2
[&] 216-224	3880	4.1	ISCNTSVIT	34	66	22	40	3	6	T........	2	.......L.	2
[&] 217-225	3881	3.7	SCNTSVITQ	39	61	22	35	3	5	....	39	......L..	3

[&] 218-226	3882	2.8	CNTSVITQA	65	35	4	28	3	4	....	65	.....L...	3
[&] 219-227	3897	2.8	NTSVITQAC	66	34	3	29	2	4	....	66	....L....	3
[&] 220-228	3898	2.7	TSVITQACP	66	34	4	28	2	4	....	66	...L.....	3
[&] 221-229	3897	2.3	SVITQACPK	71	29	3	24	2	3	....	71	..L......	3
[&] 222-230	3883	3.1	VITQACPKV	54	46	14	29	2	4	....	54	.L.......	2
[&] 223-231	3843	2.9	ITQACPKVS	52	48	17	29	2	4	....	52	L........	2
[&] 224-232	3775	2.4	TQACPKVSF	58	42	18	22	2	3	....	58	....	58
[&] 225-233	3719	2.6	QACPKVSFE	56	44	16	26	2	4	....	56	....	56
[&] 226-234	3721	2.6	ACPKVSFEP	56	44	16	26	2	4	....	56	....	56
[&] 227-235	3721	2.6	CPKVSFEPI	56	44	17	25	2	4	....	56	....	56
[&] 228-236	3721	2.6	PKVSFEPIP	56	44	17	25	2	4	....	56	....	56
[&] 229-237	3720	2.6	KVSFEPIPI	55	45	17	25	3	4	....	55	....	55
[&] 230-238	3756	2.7	VSFEPIPIH	55	45	17	26	3	4	....	55	....	55
[&] 231-239	3757	2.2	SFEPIPIHY	67	33	11	20	2	4	....	67	....	67
[&] 232-240	3757	1.6	FEPIPIHYC	78	22	7	12	2	3	....	78	....	78
[&] 233-241	3759	2.2	EPIPIHYCA	65	35	13	20	2	3	....	65	....	65
[&] 234-242	3757	1.7	PIPIHYCAP	71	29	15	12	1	2	....	71	....	71
[&] 235-243	3756	1.7	IPIHYCAPA	71	29	15	12	1	3	....	71	....	71
[&] 236-244	3759	1.7	PIHYCAPAG	71	29	15	12	1	2	....	71	....	71
[&] 237-245	3770	1.8	IHYCAPAGF	71	29	13	14	1	2	....	71	....	71
[&] 238-246	3736	1.8	HYCAPAGFA	71	29	13	14	1	2	....	71	....	71
[&] 239-247	3735	2.0	YCAPAGFAI	71	29	10	18	2	3	....	71	....	71
[&] 240-248	3736	1.9	CAPAGFAIL	72	28	10	16	2	3	....	72	....	72
[&] 241-249	3789	2.1	APAGFAILK	69	31	9	20	2	3	....	69	....	69
[&] 242-250	3793	1.6	PAGFAILKC	78	22	6	14	2	3	....	78	....	78
[&] 243-251	3820	2.6	AGFAILKCN	59	41	13	26	2	3	....	59	....	59
[&] 244-252	3835	3.6	GFAILKCND	39	61	18	40	3	5	........N	18	........E	2
[&] 245-253	3836	4.3	FAILKCNDK	28	72	17	51	3	6	.......N.	17	.......ET	<1
[&] 246-254	3839	5.0	AILKCNDKK	22	78	15	59	4	8	......N.T	15	......ET.	<1
[&] 247-255	3849	5.0	ILKCNDKKF	22	78	15	59	4	8	.....N.T.	15	.....ET..	<1
[&] 248-256	3847	5.4	LKCNDKKFN	21	79	13	61	5	9	....N.T..	13	....ET...	<1
[&] 249-257	3851	5.2	KCNDKKFNG	21	79	14	60	5	8	...N.T...	14	...ET....	<1
[+] 250-258	3854	5.8	CNDKKFNGT	17	83	9	68	6	10	..N.T....	8	..ET....S	0
[+] 251-259	3859	5.8	NDKKFNGTG	17	83	9	69	5	10	.N.T.....	8	.ET....S.	0
[&] 252-260	3871	5.7	DKKFNGTGP	24	76	10	61	5	10	N.T......	10	ET....S..	0
[&] 253-261	3878	5.3	KKFNGTGPC	26	74	11	58	4	8	.T.......	11	T....S...	<1
[+] 254-262	3886	5.8	KFNGTGPCT	18	82	11	66	5	10	T........	10	....S...K	2
[&] 255-263	3923	5.0	FNGTGPCTN	28	72	13	55	4	8	....	28	...S...K.	2
[&] 256-264	3936	4.9	NGTGPCTNV	28	72	13	55	4	8	....	28	..S...K..	2
[&] 257-265	3689	4.3	GTGPCTNVS	32	68	16	49	3	6	....	32	.S...K..T	0
[&] 258-266	3667	4.3	TGPCTNVST	33	67	15	49	3	7	....	33	S...K..T.	0
[&] 259-267	3667	3.3	GPCTNVSTV	48	52	20	29	3	5	....	48	...K..T..	0
[&] 260-268	3684	3.3	PCTNVSTVQ	48	52	20	29	3	5	....	48	..K..T...	0
[&] 261-269	3685	2.7	CTNVSTVQC	52	48	24	22	2	4	....	52	.K..T....	0
[&] 262-270	3685	2.7	TNVSTVQCT	52	48	24	22	2	4	....	52	K..T.....	0
[&] 263-271	3685	1.1	NVSTVQCTH	88	12	1	9	1	3	....	88	..T......	0
[#] 264-272	3674	0.8	VSTVQCTHG	92	8	1	6	1	2	....	92	.T.......	<1
[#] 265-273	3673	0.8	STVQCTHGI	92	8	1	5	1	2	....	92	T........	<1
[&] 266-274	3656	1.5	TVQCTHGIR	73	27	20	6	1	2	....	73	....	73
[&] 267-275	3608	1.4	VQCTHGIRP	73	27	21	5	1	2	....	73	....	73
[&] 268-276	3607	1.4	QCTHGIRPV	72	28	21	6	1	2	....	72	....	72
[&] 269-277	3607	1.5	CTHGIRPVV	72	28	20	7	<1	2	....	72	....	72
[&] 270-278	3608	1.5	THGIRPVVS	72	28	20	7	<1	2	....	72	....	72
[&] 271-279	3612	1.5	HGIRPVVST	72	28	20	7	<1	2	....	72	....	72
[&] 272-280	3620	1.5	GIRPVVSTQ	72	28	20	7	<1	2	....	72	....	72
[&] 273-281	3636	1.5	IRPVVSTQL	72	28	20	7	<1	2	....	72	....	72
[&] 274-282	3638	1.4	RPVVSTQLL	72	28	21	7	<1	2	....	72	....	72
[#] 275-283	3641	0.7	PVVSTQLLL	93	7	2	5	<1	1	....	93	....	93
[#] 276-284	3701	0.7	VVSTQLLLN	92	8	2	5	<1	1	....	92	....	92
[#] 277-285	3833	0.6	VSTQLLLNG	94	6	2	4	<1	1	....	94	....	94
[#] 278-286	3841	0.6	STQLLLNGS	95	5	<1	4	<1	2	....	95	....	95
[#] 279-287	3844	0.6	TQLLLNGSL	95	5	<1	4	<1	2	....	95	....	95
[#] 280-288	3857	0.8	QLLLNGSLA	91	9	3	5	<1	2	....	91	....	91
[#] 281-289	3882	0.9	LLLNGSLAE	90	10	4	6	<1	2	....	90	V	<1
[&] 282-290	4040	2.0	LLNGSLAEE	64	36	19	16	1	2	....	64	.......V.	<1
[&] 283-291	4237	2.8	LNGSLAEEE	54	46	17	27	2	3	....	54	......V..	<1
[&] 284-292	4297	3.6	NGSLAEEEV	41	59	13	44	2	4	....	41	.....V...	<1
[&] 285-293	4493	4.3	GSLAEEEVV	37	63	8	52	3	5	....	37	....V....	0
[&] 286-294	4483	4.2	SLAEEEVVI	39	61	9	48	3	6	....	39	...V.....	0
[&] 287-295	4582	4.1	LAEEEVVIR	39	61	10	48	3	5	....	39	..V......	0
[&] 288-296	4623	4.2	AEEEVVIRS	39	61	10	48	3	5	....	39	.V.......	0

& 289-297	5130	5.4	EEEVVIRSE	23	77	8	65	4	7	V	1	V........	0
& 290-298	5265	5.3	EEVVIRSEN	24	76	8	64	4	7	.......V.	1	....	24
& 291-299	5389	5.2	EVVIRSENF	26	74	8	61	4	7	......V..	1	....	26
& 292-300	5526	5.4	VVIRSENFT	24	76	7	65	4	7	.....V...	1	........S	3
+ 293-301	5685	5.6	VIRSENFTN	19	81	9	68	4	7	....V...D	1	.......SD	1
& 294-302	5692	5.0	IRSENFTNN	23	77	10	63	4	6	...V...D.	1	......SD.	2
& 295-303	5694	5.5	RSENFTNNA	21	79	8	67	4	7	..V...D..	1	.....SD..	2
+ 296-304	5731	5.5	SENFTNNAK	20	80	8	68	4	7	.V...D...	1	....SD...	2
+ 297-305	5732	6.2	ENFTNNAKT	15	85	7	72	6	10	V...D....	1	...SD....	2
+ 298-306	5936	5.4	NFTNNAKTI	18	82	14	64	4	7	...D.....	14	..SD.....	6
+ 299-307	6068	5.4	FTNNAKTII	18	82	13	65	4	8	..D......	13	.SD......	6
& 300-308	6084	4.8	TNNAKTIIV	25	75	17	55	4	6	.D.......	17	SD......I	<1
& 301-309	6096	4.4	NNAKTIIVQ	25	75	23	49	3	5	D........	23	D......I.	<1
& 302-310	6150	3.6	NAKTIIVQL	49	51	6	43	2	4	....	49	......I..	1
& 303-311	6146	4.5	AKTIIVQLN	30	70	17	50	3	5	....	30	.....I...	1
& 304-312	6146	4.9	KTIIVQLNE	25	75	17	55	3	6	........T	2	....I...T	0
+ 305-313	6135	5.7	TIIVQLNES	14	86	10	72	4	7	.......T.	2	...I...T.	0
& 306-314	6149	5.1	IIVQLNESV	21	79	10	66	3	6	......T..	2	..I...T..	0
+ 307-315	6140	6.4	IVQLNESVE	9	91	8	78	5	9	.....T...	1	.I...T...	0
+ 308-316	6139	6.4	VQLNESVEI	10	90	8	77	4	9	....T....	1	I...T....	0
+ 309-317	6141	6.5	QLNESVEIN	10	90	9	76	5	10	...T.....	1	...T.....	1
+ 310-318	6145	6.3	LNESVEINC	12	88	9	74	5	10	..T......	1	..T......	1
+ 311-319	6136	6.6	NESVEINCT	11	89	9	74	6	11	.T.......	1	.T.......	1
+ 312-320	6143	6.2	ESVEINCTR	15	85	9	71	5	10	T........	1	T........	1
& 313-321	6156	5.4	SVEINCTRP	22	78	9	65	4	7	....	22	....	22
& 314-322	6151	5.0	VEINCTRPN	31	69	9	56	4	7	....	31	....	31
& 315-323	6156	5.0	EINCTRPNN	32	68	9	54	4	7	....	32	....	32
& 316-324	6167	3.4	INCTRPNNN	56	44	9	33	3	5	....	56	....	56
& 317-325	6166	3.5	NCTRPNNNT	56	44	8	33	3	5	....	56	....	56
& 318-326	6219	3.4	CTRPNNNTR	55	45	8	34	3	5	....	55	....	55
& 319-327	6209	4.1	TRPNNNTRK	46	54	7	43	3	6	....	46	....	46
& 320-328	6210	4.4	RPNNNTRKS	36	64	16	45	3	6	........R	2	........G	16
& 321-329	6210	4.5	PNNNTRKSI	35	65	16	45	4	7	.......R.	1	.......G.	16
+ 322-330	6195	5.8	NNNTRKSIH	17	83	13	64	5	10	......R.R	<1	......G..	13
+ 323-331	6199	6.0	NNTRKSIHI	16	84	13	65	6	10	.....R.R.	<1	.....G...	13
+ 324-332	6310	6.1	NTRKSIHIG	15	85	13	67	6	10	....R.R..	<1	....G....	13
+ 325-333	6312	6.1	TRKSIHIGP	14	86	12	67	6	10	...R.R...	<1	...G.....	12
+ 326-334	6245	6.1	RKSIHIGPG	15	85	13	68	5	9	..R.R....	<1	..G......	13
+ 327-335	6221	6.4	KSIHIGPGR	12	88	12	71	5	9	.R.R.....	<1	.G.......	12
+ 328-336	6212	6.1	SIHIGPGRA	14	86	9	72	5	9	R.R......	<1	G........	9
& 329-337	6210	5.9	IHIGPGRAF	25	75	6	65	5	9	.R.......	<1	....	25
& 330-338	6209	6.2	HIGPGRAFY	22	78	5	68	5	10	R.......V	<1	....	22
& 331-339	6158	5.7	IGPGRAFYA	23	77	16	57	4	9	.......VT	<1	....	23
& 332-340	6152	5.3	GPGRAFYAT	25	75	18	54	4	7	......VTI	<1	....	25
& 333-341	5529	5.3	PGRAFYATG	27	73	17	51	4	8	.....VTI.	<1	....	27
+ 334-342	5455	6.5	GRAFYATGE	12	88	11	71	6	12	....VTI.-	0	........D	11
+ 335-343	5510	6.7	RAFYATGEI	11	89	11	72	7	13	...VTI.-K	0	.......D.	11
+ 336-344	5493	6.5	AFYATGDII	14	86	11	68	7	13	..VTI.-K.	0	....	14
+ 337-345	5497	6.3	FYATGDIIG	14	86	11	69	6	11	.VTI.-K..	0	....	14
+ 338-346	5492	6.1	YATGDIIGD	13	87	10	72	5	10	VTI.-K..N	0	....	13
+ 339-347	5495	5.7	ATGDIIGDI	17	83	10	68	5	9	TI.-K..NM	0	....	17
& 340-348	5548	5.0	TGDIIGDIR	24	76	19	54	4	7	I.-K..NM.	0	....	24
+ 341-349	5555	5.6	GDIIGDIRQ	16	84	14	66	4	8	.-K..NM..	0	....	16
+ 342-350	6153	5.4	DIIGDIRQA	16	84	16	65	3	7	-K..NM...	0	....	16
& 343-351	6213	3.8	IIGDIRQAH	45	55	10	42	3	5	K..NM....	<1	....	45
& 344-352	6244	3.5	IGDIRQAHC	47	53	11	40	2	5	..NM.....	<1	....	47
& 345-353	6140	3.4	GDIRQAHCN	51	49	10	37	2	5	.NM......	<1	....	51
& 346-354	6152	4.3	DIRQAHCNI	28	72	22	46	3	6	NM.......	<1	....	28
& 347-355	6105	4.0	IRQAHCNIS	33	67	26	38	3	6	M........	<1	....	33
& 348-356	6088	5.3	RQAHCNISR	20	80	15	60	4	8	....	20	........T	<1
+ 349-357	6086	6.7	QAHCNISRA	11	89	7	76	6	11	....	11	.......T.	<1
+ 350-358	6078	7.3	AHCNISRAK	13	87	3	76	8	14	....	13	......T..	<1
+ 351-359	6070	7.3	HCNISRAKW	13	87	3	76	8	14	....	13	.....T...	<1
+ 352-360	6061	7.4	CNISRAKWN	13	87	3	75	9	15	....	13	....T....	<1
+ 353-361	6054	8.1	NISRAKWNN	9	91	2	77	11	20	....	9	...T....D	0
+ 354-362	6019	8.0	ISRAKWNNT	10	90	3	77	11	19	....	10	..T....D.	0
+ 355-363	6018	7.8	SRAKWNNTL	10	90	4	76	10	17	....	10	.T....D..	0
+ 356-364	6009	8.4	RAKWNNTLR	5	95	3	79	13	22	........K	3	T....D..K	0
+ 357-365	6019	8.0	AKWNNTLRQ	7	93	5	76	12	20	.......K.	5	....D..KH	<1
+ 358-366	6008	7.5	KWNNTLKQI	10	90	7	73	10	17	....	10	...D...H.	<1
+ 359-367	5990	7.2	WNNTLKQIV	11	89	6	75	8	14	........A	3	..D...H..	<1

[+] 360-368	5962	8.4	NNTLRQIVI	4	96	4	79	13	22	....K..AS	<1	.D..KH..G	0
[+] 361-369	5961	8.3	NTLRQIVIK	4	96	4	81	11	19	...K..AS.	<1	D..KH..G.	0
[+] 362-370	5951	7.6	TLKQIVTKL	7	93	4	81	8	15	.....AS..	<1	...H..G..	0
[+] 363-371	5941	8.0	LKQIVTKLR	6	94	4	80	10	18	....AS...	<1	..H..G..K	0
[+] 364-372	5844	8.3	KQIVTKLRE	6	94	4	79	11	20	...AS....	<1	.H..G..K.	0
[+] 365-373	5852	7.6	QIVTKLREQ	7	93	5	79	9	16	..AS.....	2	H..G..K..	0
[+] 366-374	5855	7.3	IVTKLREQF	7	93	6	79	8	14	.AS......	2	..G..K...	<1
[+] 367-375	792	6.2	VKKLREQFG	14	86	7	61	18	28	AS.......	3	.G..K...R	0
[+] 368-376	790	6.6	KKLREQFGN	10	90	5	66	19	29	S........	3	G..K...R.	0
[&] 369-377	791	5.6	KLREQFGNN	23	77	8	53	16	24	....	23	..K...R.A	4
[&] 370-378	791	5.5	LREQFGNNK	23	77	9	54	14	23	....	23	.K...R.AT	4
[&] 371-379	791	5.6	REQFGNNKT	23	77	9	53	16	25	....	23	K...R.ATK	0
[&] 372-380	789	5.3	EQFGNNKTI	29	71	9	47	15	23	....	29	...R.ATK.	<1
[+] 373-381	788	5.8	QFGNNKTIV	19	81	6	58	17	26	I	6	..R.ATK..	<1
[+] 374-382	784	5.8	FGNNKTIVF	20	80	6	57	17	25	.......I.	6	.R.ATK...	<1
[+] 375-383	784	6.2	GNNKTIVFN	13	87	5	63	18	27	......I.K	3	R.ATK....	0
[+] 376-384	5065	7.2	GNKTIVFNQ	10	90	6	76	9	15	N....I.K.	1	NATK....H	0
[+] 377-385	5257	6.0	NKTIVFNQS	19	81	14	59	7	12	....I.K..	1	ATK....H.	0
[+] 378-386	5180	6.0	KTIVFNQSS	19	81	14	60	6	11	...I.K...	1	TK....H..	<1
[&] 379-387	4961	5.5	TIVFNQSSG	21	79	15	59	5	9	..I.K....	1	K....H...	<1
[&] 380-388	4929	5.4	IVFNQSSGG	22	78	15	58	5	8	.I.K.....	1	....H....	6
[&] 381-389	4909	5.4	VFNQSSGGD	22	78	15	58	5	8	I.K......	1	...H.....	6
[&] 382-390	4900	4.6	FNQSSGGDP	33	67	9	55	3	6	.K.......	5	..H......	9
[&] 383-391	4752	4.6	NQSSGGDPE	32	68	9	56	3	6	K........	5	.H.......	9
[&] 384-392	4583	4.0	QSSGGDPEI	36	64	10	51	3	5	....	36	H........	10
[&] 385-393	4538	3.1	SSGGDPEIV	56	44	10	31	3	4	....	56	....	56
[&] 386-394	4471	3.4	SGGDPEIVM	47	53	13	37	3	5	........T	13	....	47
[&] 387-395	4454	3.3	GGDPEIVMH	50	50	13	35	3	5	.......T.	13	....	50
[&] 388-396	4441	3.8	GDPEIVMHS	42	58	12	42	4	6	......T..	12	....	42
[&] 389-397	4395	3.8	DPEIVMHSF	42	58	12	42	4	6	.....T...	12	....	42
[&] 390-398	4331	3.9	PEIVMHSFN	42	58	12	42	4	6	....T....	12	....	42
[&] 391-399	4327	3.3	EIVMHSFNC	53	47	13	31	3	5	...T.....	13	....	53
[&] 392-400	4279	3.7	IVMHSFNCG	47	53	12	38	3	6	..T......	12	....	47
[&] 393-401	4277	3.6	VMHSFNCGG	48	52	12	37	3	5	.T.......	12	....	48
[&] 394-402	4290	3.1	MHSFNCGGE	51	49	14	32	2	4	T........	14	....	51
[&] 395-403	4290	2.3	HSFNCGGEF	66	34	10	22	2	3	....	66	....	66
[&] 396-404	4287	2.2	SFNCGGEFF	68	32	10	21	2	3	....	68	....	68
[&] 397-405	3968	1.7	FNCGGEFFY	78	22	7	13	1	3	....	78	....	78
[&] 398-406	3960	1.6	NCGGEFFYC	79	21	7	13	1	3	....	79	....	79
[&] 399-407	3902	1.9	CGGEFFYCN	74	26	7	18	2	3	....	74	....	74
[&] 400-408	3902	2.7	GGEFFYCNT	47	53	26	24	2	3	........S	26	....	47
[&] 401-409	3901	2.9	GEFFYCNTT	47	53	20	31	2	4	.......S.	20	....	47
[&] 402-410	3898	4.6	EFFYCNTTQ	21	79	12	64	3	6	......S..	10	........R	2
[&] 403-411	3898	4.6	FFYCNTTQL	21	79	12	64	3	6	.....S...	10	.......R.	2
[&] 404-412	3898	4.6	FYCNTTQLF	21	79	12	64	3	6	....S....	9	......R..	2
[&] 405-413	3897	4.8	YCNTTQLFN	20	80	12	64	4	7	...S.....	9	.....R...	2
[+] 406-414	3726	5.0	CNTTQLFNS	19	81	12	65	4	7	..S......	10	....R....	2
[+] 407-415	3737	5.4	NTTQLFNST	16	84	11	68	5	9	.S.......	9	...R.....	2
[+] 408-416	3732	5.1	TTQLFNSTW	18	82	11	67	4	8	S........	11	..R......	2
[+] 409-417	3727	6.3	TQLFNSTWN	14	86	8	72	6	11	........F	<1	.R.......	1
[+] 410-418	3725	7.5	PLFNSTWGS	7	93	4	81	8	16	Q......FN	<1	R......NV	0
[+] 411-419	3701	7.1	LFNSTWGSN	7	93	7	80	7	14	......FNS	<1	......NVS	<1
[+] 412-420	3635	7.9	FNSTWGSND	7	93	2	81	9	19	.....FNST	<1	.....NVST	<1
[+] 413-421	3633	8.3	NSTWGSNDS	5	95	2	82	11	22	....FNSTW	<1	....NVSTW	<1
[+] 414-422	3632	8.4	STWGSNDSR	4	96	2	81	13	24	...FNSTWS	<1	...NVSTWN	<1
[+] 415-423	3630	8.4	TWGSNDSRP	4	96	2	79	14	26	..FNSTWST	<1	..NVSTWNI	0
[+] 416-424	3628	8.7	WGSNDSRPE	4	96	2	79	15	27	.FNSTWST.	<1	.NVSTWNIS	0
[+] 417-425	3613	8.5	GSNDSRPEN	4	96	2	78	16	28	FNSTWST.G	<1	NVSTWNISQ	0
[+] 418-426	3610	8.7	SNDSRPENN	5	95	2	78	15	28	NSTWST.GS	<1	VSTWNISQT	0
[+] 419-427	3595	8.6	NDSRPENNT	6	94	2	78	15	27	STWST.GSN	<1	STWNISQTE	0
[+] 420-428	3567	8.6	DSRPENNTG	6	94	2	77	15	28	TWST.GSNN	<1	TWNISQTE.	0
[+] 421-429	3346	8.4	SRPENNTGG	6	94	2	76	16	28	WST.GSNNT	<1	WNISQTE.S	0
[+] 422-430	3055	8.3	RPENNTGGN	8	92	2	74	15	27	ST.GSNNTE	<1	NISQTE.SY	0
[+] 423-431	2411	7.7	PENNTGGNE	13	87	2	70	14	26	T.GSNNTEG	1	ISQTE.SYN	0
[+] 424-432	1945	7.3	ENNTGGNET	15	85	3	68	14	25	.GSNNTEGS	1	SQTE.SYN.	0
[+] 425-433	1232	7.5	SENTTGNGT	5	95	3	76	16	29	GS.N.EGSD	1	QTEGSY.TE	0
[+] 426-434	825	7.1	TEVKNNTEG	5	95	3	75	17	31	SNNTEGSDT	2	..GSY...E	0
[+] 427-435	515	6.4	EVKNNTEGT	7	93	5	71	17	31	NNTEGSDT-	0	.GSY...EN	0
[+] 428-436	322	5.9	STENITHPE	8	92	7	66	19	33	N..GSDT--	0	GSY.TEEN-	0
[+] 429-437	319	5.8	TENITHPEI	8	92	8	66	17	31	..GSDT--.	0	SY.TEEN-.	0
[+] 430-438	319	5.9	TGQSDSPIT	8	92	7	68	17	32	E.SDT--..	0	YNTEEN-..	0

+ 431-439	319	5.6	NITHPEITL	11	89	8	65	16	30	GSDT--...	0	.TEEN-...	0
+ 432-440	319	5.7	ITHPEITLP	11	89	8	66	16	30	SDT--....	0	TEEN-....	0
+ 433-441	319	5.6	THPEITLPC	11	89	8	67	15	28	DT--.....	0	EEN-.....	0
+ 434-442	319	5.5	HPEITLPCR	11	89	8	68	13	27	T--......	0	EN-......	0
+ 435-443	319	5.3	PEITLPCRI	12	88	8	71	8	21	--.......	0	N-.......	0
+ 436-444	319	4.6	EITLPCRIK	16	84	12	66	6	15	-........	0	-........	0
& 437-445	3542	3.3	ITLPCRIKQ	48	52	9	41	2	4	....	48	....	48
& 438-446	3608	3.6	TLPCRIKQI	45	55	9	44	3	5	....	45	....	45
& 439-447	3607	3.5	LPCRIKQII	44	56	12	41	2	4	....	44	V	12
& 440-448	3608	3.7	PCRIKQIIN	42	58	12	43	2	4	....	42	.......V.	12
& 441-449	3606	3.7	CRIKQIINM	40	60	12	45	3	4	....	40	......V..	10
& 442-450	3603	3.7	RIKQIINMW	40	60	12	45	3	5	....	40	.....V...	10
& 443-451	3603	3.2	IKQIINMWQ	52	48	11	35	2	4	....	52	....V....	11
& 444-452	3603	3.9	KQIINMWQE	44	56	11	42	3	5	........K	3	...V.....	11
& 445-453	3604	3.6	QIINMWQEV	46	54	12	40	2	4	.......K.	2	..V......	12
& 446-454	3603	3.5	IINMWQEVG	47	53	12	39	2	4	......K..	2	.V.......	12
& 447-455	3430	3.3	INMWQEVGK	45	55	15	37	2	4	.....K...	3	V........	15
& 448-456	3355	2.7	NMWQEVGKA	61	39	8	28	2	3	....K....	3	....	61
& 449-457	3307	2.8	MWQEVGKAM	60	40	8	30	2	4	...K.....	3	....	60
& 450-458	3307	2.0	WQEVGKAMY	73	27	6	19	2	3	..K......	6	....	73
& 451-459	3310	2.1	QEVGKAMYA	72	28	6	20	2	3	.K.......	6	....	72
& 452-460	3310	2.1	EVGKAMYAP	72	28	6	19	2	3	K........	6	....	72
& 453-461	3311	1.0	VGKAMYAPP	89	11	2	9	<1	2	....	89	....	89
& 454-462	3309	1.2	GKAMYAPPI	86	14	2	11	1	2	....	86	....	86
& 455-463	3308	3.1	KAMYAPPIR	46	54	14	38	3	4	........S	14	....	46
& 456-464	3309	2.9	AMYAPPIRG	48	52	14	36	2	4	.......S.	14	....	48
& 457-465	3305	4.5	MYAPPIRGQ	34	66	9	54	3	6	......S..	8	I	2
& 458-466	3306	4.5	YAPPIRGQI	35	65	8	54	3	6	.....S...	7	.......I.	1
& 459-467	3306	5.4	APPIRGQIR	30	70	5	61	4	9	....S....	5	......I..	1
& 460-468	3306	5.3	PPIRGQIRC	31	69	5	60	4	8	...S.....	5	.....I...	1
& 461-469	3306	5.8	PIRGQIRCS	28	72	5	62	5	11	..S......	4	....I....	1
& 462-470	3304	5.9	IRGQIRCSS	27	73	5	63	5	11	.S.......	4	...I.....	<1
& 463-471	3304	6.0	RGQIRCSSN	27	73	5	62	6	12	S........	3	..I......	<1
& 464-472	3305	4.9	GQIRCSSNI	38	62	4	54	4	8	....	38	.I.......	2
& 465-473	3305	4.9	QIRCSSNIT	39	61	4	53	4	8	....	39	I........	2
& 466-474	3306	3.6	IRCSSNITG	51	49	9	38	3	5	....	51	....	51
& 467-475	3285	3.7	RCSSNITGL	50	50	9	38	3	5	....	50	I	<1
& 468-476	3282	2.7	CSSNITGLL	62	38	8	28	2	4	....	62	.......IM	0
& 469-477	3279	2.9	SSNITGLLL	60	40	8	29	2	4	....	60	......IM.	0
& 470-478	3278	2.5	SNITGLLLT	62	38	10	27	2	3	....	62	.....IM..	0
& 471-479	3277	2.5	NITGLLLTR	62	38	9	27	2	3	....	62	....IM...	0
& 472-480	3278	2.1	ITGLLLTRD	67	33	10	22	1	3	....	67	...IM....	0
& 473-481	3277	2.1	TGLLLTRDG	67	33	9	22	2	3	....	67	..IM.....	0
& 474-482	3276	2.1	GLLLTRDGG	67	33	9	22	2	3	....	67	.IM......	0
& 475-483	3262	4.3	LLLTRDGGN	36	64	7	54	3	6	....	36	IM......K	0
+ 476-484	3216	6.0	LLTRDGGNN	16	84	7	72	5	10	........S	7	M......K.	0
+ 477-485	3063	7.2	LTRDGGNNN	5	95	4	84	7	14	.......S.	3	......K.R	<1
+ 478-486	3008	8.0	TRDGGNNNN	2	98	2	84	11	21	......S..	1	.....K.RT	0
+ 479-487	2898	8.3	RDGGNQNGS	3	97	2	81	15	26	.....S.NE	<1	....KNRTE	0
+ 480-488	2219	8.2	DGGNQNGSN	3	97	2	77	17	30	....S.NE-	0	...KNRTE.	0
+ 481-489	1400	7.8	GGNNTTTNG	4	96	2	74	19	33	...SNNE--	0	..K.R.E.T	0
+ 482-490	971	7.4	GNNTTTNGT	5	95	3	74	18	32	..SNNE---	0	.K.R.E.T-	0
+ 483-491	448	6.4	NNTTTNGTQ	12	88	3	61	24	36	.SNNE----	0	K.R.E.T--	0
+ 484-492	184	5.7	RNISDGTNG	7	93	7	58	28	43	S.NE-----	0	NRTENT---	0
+ 485-493	123	4.8	NISDGTNGN	11	89	10	59	20	35	.NE-----S	0	RTENT---T	0
+ 486-494	121	4.7	ISDGTNGNE	11	89	10	60	19	34	NE-----S.	0	TENT---T.	0
+ 487-495	121	4.7	SDGTNGNET	11	89	10	60	19	34	E-----S.I	0	ENT---T..	0
+ 488-496	120	4.6	NENGTTETF	11	89	11	63	16	31	-----S.I.	0	.T---....	0
+ 489-497	119	4.5	ENGTTETFR	11	89	11	64	14	29	----S.I..	0	T---.....	0
+ 490-498	119	4.4	TNGNETFRP	12	88	11	66	12	27	---S.I...	0	---T.....	0
+ 491-499	104	4.3	GTTETFRPG	13	88	13	63	12	28	--S.I....	0	--.......	0
492-500 *	62	4.0	GNETFRPIG	16	84	11	63	10	31	-S.I...G.	0	-T.....G.	0
& 493-501	1627	3.1	TEIFRPGGG	34	66	20	43	3	4	S........	2	..T......	14
& 494-502	1587	2.9	EIFRPGGGD	34	66	29	33	3	5	....	34	.T......N	5
& 495-503	1064	2.9	TFRPGGGDM	32	68	31	33	4	6	I........	31	.......N.	4
& 496-504	1014	1.8	FRPGGGDMR	75	25	7	14	4	6	....	75	......N.M	0
& 497-505	964	2.0	RPGGGDMRD	72	28	7	17	4	6	....	72	.....N.M.	0
& 498-506	955	2.0	PGGGDMRDN	72	28	8	16	4	6	....	72	....N.M..	0
& 499-507	899	2.6	GGGDMRDNW	57	43	16	22	5	7	....	57	...N.M...	0
& 500-508	879	2.2	GGDMRDNWR	62	38	18	17	4	6	....	62	..N.M....	<1
& 501-509	789	2.3	GDMRDNWRS	58	42	20	18	5	7	....	58	.N.M.....	<1

[&] 502-510	549	2.0	DMRDNWRSE	71	29	8	15	5	8	....	71	N.M......	<1
[&] 503-511	535	1.5	MRDNWRSEL	79	21	10	6	6	7	....	79	.M.......	<1
[&] 504-512	528	1.3	RDNWRSELY	80	20	11	4	5	6	....	80	M........	<1
[#] 505-513	527	0.8	DNWRSELYK	91	9	2	3	4	6	....	91	........N	0
[#] 506-514	529	0.6	NWRSELYKY	94	6	1	1	4	5	....	94	.......N.	0
[#] 507-515	533	0.6	WRSELYKYK	93	7	1	2	4	5	....	93	......N..	0
[#] 508-516	537	0.6	RSELYKYKV	93	7	1	2	3	4	....	93	.....N...	0
[#] 509-517	541	0.7	SELYKYKVV	92	8	2	3	3	4	....	92	....N....	0
[&] 510-518	542	1.7	ELYKYKVVK	70	30	15	11	4	5	....	70	...N.....	0
[&] 511-519	542	1.8	LYKYKVVKI	69	31	15	12	4	6	....	69	..N......	0
[&] 512-520	541	1.8	YKYKVVKIE	69	31	15	13	4	6	....	69	.N.......	0
[&] 513-521	541	1.8	KYKVVKIEP	69	31	15	13	4	6	....	69	N........	0
[&] 514-522	540	2.5	YKVVKIEPL	52	48	16	27	4	6	....	52	....	52
[&] 515-523	540	2.5	KVVKIEPLG	52	48	17	27	4	6	....	52	....	52
[&] 516-524	543	3.3	VVKIEPLGV	39	61	13	44	4	8	....	39	....	39
[&] 517-525	544	3.4	VKIEPLGVA	39	61	13	44	4	9	....	39	....	39
[&] 518-526	544	3.3	KIEPLGVAP	39	61	13	44	4	8	....	39	....	39
[&] 519-527	546	2.7	IEPLGVAPT	42	58	19	34	4	6	....	42	....	42
[&] 520-528	547	3.5	EPLGVAPTR	22	78	20	53	5	8	........K	20	........K	20
[&] 521-529	548	3.4	PLGVAPTRA	22	78	20	53	5	8	.......K.	20	.......K.	20
[&] 522-530	546	3.6	LGVAPTRAK	22	78	19	53	5	9	......K..	19	......K..	19
[&] 523-531	548	2.9	GVAPTRAKR	33	67	22	40	5	8	.....K...	22	.....K...	22
[&] 524-532	549	3.0	VAPTRAKRR	33	67	22	40	5	8	....K....	22	....K....	22
[&] 525-533	548	1.9	APTKAKRRV	47	53	41	8	3	6	....	47	....	47
[&] 526-534	551	1.9	PTKAKRRVV	47	53	41	8	3	6	....	47	....	47
[&] 527-535	549	2.3	TRAKRRVVQ	40	60	40	15	5	7	.K.......	40	.K.......	40
[&] 528-536	557	2.3	KAKRRVVQR	40	60	40	16	4	7	....	40	....	40
[&] 529-537	557	1.3	AKRRVVQRE	82	18	7	7	4	6	....	82	....	82
[&] 530-538	557	1.5	KRRVVQREK	78	22	7	11	4	6	....	78	....	78
[&] 531-539	559	1.5	RRVVQREKR	80	20	7	10	3	6	....	80	....	80
[&] 532-540	560	1.7	RVVQREKRA	77	23	7	13	4	7	....	77	....	77
[&] 533-541	548	2.6	VVQREKRAV	54	46	16	26	5	8	....	54	........A	16
[&] 534-542	395	2.4	VQREKRAVG	63	37	9	21	7	10	....	63	.......A.	<1
[&] 535-543	152	3.2	RREKRAIGT	24	76	23	41	11	17	Q.....V.-	0	Q.....A.-	0
[&] 536-544	152	3.4	REKRAIGTI	24	76	23	39	13	18	.....V.-.	0	.....A.-L	0
[&] 537-545	153	3.4	EKRAIGTIG	24	76	23	39	14	19	....V.-..	0	....A.-L.	0
[&] 538-546	134	3.3	KRAIGTIGA	28	72	26	31	15	20	...V.-...	0	...A.-L..	0
[&] 539-547	133	3.3	RAIGTIGAM	28	72	26	30	16	22	..V.-...L	0	..A.-L..L	0
[+] 540-548	293	4.2	AVGTIGAMF	18	82	13	61	8	16	...-...L.	0	.A.-L..L.	0
[+] 541-549	305	4.2	VGTIGAMFL	18	82	13	62	7	15	..-...L..	0	A.-L..L..	0
[&] 542-550	307	3.6	GTIGAMFLG	31	69	24	39	7	14	.-...L...	0	.-L..L...	0
[&] 543-551	382	3.7	TLGAMFLGF	29	71	25	39	7	13	-I..L....	0	-...L....	0
[+] 544-552	1275	3.7	LGAMFLGFL	18	82	18	61	3	6	I..L.....	18	...L.....	11
[&] 545-553	1263	2.5	GAMFLGFLG	35	65	30	33	2	4	..L......	30	..L......	30
[&] 546-554	1253	2.8	AMFLGFLGA	30	70	30	38	2	4	.L.......	30	.L.......	30
[&] 547-555	1257	2.7	LFLGFLGAA	30	70	30	38	2	4	....	30	....	30
[&] 548-556	1315	1.2	FLGFLGAAG	84	16	6	9	1	3	....	84	....	84
[&] 549-557	1321	0.9	LGFLGAAGS	87	13	7	6	<1	2	....	87	....	87
[&] 550-558	1323	0.8	GFLGAAGST	89	11	6	4	<1	2	....	89	....	89
[&] 551-559	1310	0.8	FLGAAGSTM	89	11	6	4	<1	2	....	89	....	89
[&] 552-560	1310	0.7	LGAAGSTMG	90	10	6	3	<1	2	....	90	....	90
[&] 553-561	1305	0.7	GAAGSTMGA	90	10	6	3	1	2	....	90	....	90
[&] 554-562	1307	0.8	AAGSTMGAA	89	11	6	3	1	2	....	89	....	89
[#] 555-563	1307	0.6	AGSTMGAAS	92	8	3	4	1	2	....	92	....	92
[&] 556-564	1260	2.6	GSTMGAASI	24	76	24	50	2	3	........M	23	L	24
[&] 557-565	1274	3.1	STMGAASLT	24	76	23	51	2	4	.......M.	19	....	24
[&] 558-566	1300	3.2	TMGAASLTL	24	76	23	51	2	5	......M..	18	....	24
[&] 559-567	1399	3.1	MGAASLTLT	24	76	23	51	2	4	.....M...	19	....	24
[&] 560-568	1427	3.2	GAASLTLTV	24	76	22	52	2	4	....M....	19	....	24
[&] 561-569	1462	3.3	AASLTLTVQ	24	76	22	52	2	4	...M.....	19	....	24
[&] 562-570	1459	3.3	ASLTLTVQA	24	76	22	52	3	5	..M......	19	....	24
[&] 563-571	1463	3.1	SLTLTVQAR	24	76	22	52	2	4	.M.......	20	....	24
[+] 564-572	1456	3.8	LTLTVQARQ	18	82	16	63	2	5	M........	8	....	18
[&] 565-573	1502	2.2	TLTVQARQL	55	45	24	19	2	3	....	55	....	55
[&] 566-574	1509	1.8	LTVQARQLL	62	38	24	11	2	3	....	62	....	62
[&] 567-575	1515	1.8	TVQARQLLS	62	38	24	12	2	3	....	62	....	62
[&] 568-576	1478	2.1	VQARQLLSG	59	41	23	16	2	4	....	59	....	59
[&] 569-577	1513	1.9	QARQLLSGI	63	37	23	12	2	3	....	63	....	63
[&] 570-578	1464	2.3	ARQLLSGIV	57	43	23	18	2	4	....	57	....	57
[&] 571-579	1501	2.2	RQLLSGIVQ	59	41	22	16	2	4	....	59	....	59
[&] 572-580	1541	2.3	QLLSGIVQQ	59	41	21	18	2	4	....	59	....	59

& 573-581	1614	1.4	LLSGIVQQQ	82	18	5	11	2	3	....	82	....	82
& 574-582	1619	2.2	LSGIVQQQN	69	31	8	21	2	4	....	69	....	69
& 575-583	1613	2.4	SGIVQQQNN	63	37	8	26	3	5	....	63	....	63
& 576-584	1629	2.4	GIVQQQNNL	63	37	9	26	2	4	....	63	....	63
& 577-585	1634	2.3	IVQQQNNLL	65	35	9	24	2	4	....	65	....	65
& 578-586	1629	2.9	VQQQNNLLR	58	42	7	33	2	5	....	58	....	58
& 579-587	1665	2.5	QQQNNLLRA	62	38	7	29	2	4	....	62	....	62
& 580-588	1669	2.5	QQNNLLRAI	62	38	7	29	2	4	....	62	....	62
& 581-589	1690	2.6	QNNLLRAIE	61	39	7	30	2	4	....	61	....	61
& 582-590	1832	2.7	NNLLRAIEA	58	42	11	29	2	4	....	58	....	58
& 583-591	1890	1.8	NLLRAIEAQ	74	26	8	17	<1	3	....	74	....	74
& 584-592	1941	1.5	LLRAIEAQQ	79	21	9	12	<1	2	....	79	....	79
& 585-593	1944	1.5	LRAIEAQQH	79	21	8	12	1	3	....	79	....	79
& 586-594	1944	2.2	RAIEAQQHL	57	43	24	18	1	3	....	57	....	57
& 587-595	1930	1.6	AIEAQQHLL	65	35	25	8	1	2	....	65	....	65
& 588-596	1849	1.9	IEAQQHLLQ	64	36	23	12	1	3	....	64	....	64
& 589-597	1844	1.9	EAQQHLLQL	64	36	23	12	1	3	....	64	....	64
& 590-598	2008	1.8	AQQHLLQLT	64	36	24	10	1	3	....	64	....	64
& 591-599	1962	1.8	QQHLLQLTV	64	36	24	11	1	3	....	64	....	64
& 592-600	1966	1.7	QHLLQLTVW	65	35	24	10	1	2	....	65	....	65
& 593-601	1967	1.6	HLLQLTVWG	66	34	24	8	1	2	....	66	....	66
& 594-602	1976	1.4	LLQLTVWGI	67	33	26	6	<1	2	....	67	....	67
# 595-603	2014	0.6	LQLTVWGIK	93	7	2	4	<1	2	....	93	....	93
# 596-604	2011	0.6	QLTVWGIKQ	93	7	2	4	<1	1	....	93	....	93
# 597-605	2039	0.3	LTVWGIKQL	97	3	1	1	<1	1	....	97	....	97
# 598-606	2038	0.4	TVWGIKQLQ	96	4	1	2	<1	1	....	96	....	96
# 599-607	2038	0.4	VWGIKQLQA	96	4	1	2	<1	1	....	96	....	96
# 600-608	2042	0.4	WGIKQLQAR	96	4	1	3	<1	1	....	96	....	96
& 601-609	2012	0.9	GIKQLQARV	88	12	6	6	<1	1	I	6	....	88
& 602-610	2007	0.9	IKQLQARVL	88	12	6	5	<1	1	.......I.	6	....	88
& 603-611	1994	0.8	KQLQARVLA	89	11	6	4	<1	1	......I..	6	....	89
& 604-612	1985	1.4	QLQARVLAV	76	24	9	14	<1	2	.....I...	6	....	76
& 605-613	1981	1.5	LQARVLAVE	76	24	9	14	<1	2	....I....	6	....	76
& 606-614	1966	1.7	QARVLAVER	74	26	9	16	1	2	...I.....	6	....	74
& 607-615	1964	1.7	ARVLAVERY	73	27	9	16	1	3	..I......	6	....	73
& 608-616	1964	1.8	RVLAVERYL	73	27	6	20	1	3	.I.......	6	....	73
& 609-617	1898	3.2	VLAVERYLK	42	58	20	36	2	5	I........	3	........R	20
& 610-618	1920	2.8	LAVERYLKD	47	53	20	30	2	4	....	47	.......R.	20
& 611-619	1919	2.8	AVERYLKDQ	47	53	20	31	2	4	....	47	......R..	20
& 612-620	1917	3.0	VERYLKDQQ	45	55	20	32	2	4	....	45	.....R...	20
& 613-621	1918	2.6	ERYLKDQQL	49	51	24	25	2	4	....	49	....R....	24
& 614-622	1914	2.8	RYLKDQQLL	47	53	23	28	2	4	....	47	...R.....	23
& 615-623	1919	2.6	YLKDQQLLG	49	51	24	25	2	3	....	49	..R......	24
& 616-624	1918	2.8	LKDQQLLGI	46	54	25	27	2	4	....	46	.R.......	25
& 617-625	1918	2.7	KDQQLLGIW	49	51	25	24	2	4	....	49	R........	25
& 618-626	1983	1.2	DQQLLGIWG	84	16	4	11	<1	2	....	84	....	84
& 619-627	1984	1.2	QQLLGIWGC	84	16	4	11	<1	2	....	84	....	84
& 620-628	1985	1.4	QLLGIWGCS	81	19	4	14	<1	2	....	81	....	81
& 621-629	1987	1.3	LLGIWGCSG	82	18	4	13	<1	2	....	82	....	82
& 622-630	1970	1.4	LGIWGCSGK	80	20	5	14	<1	2	....	80	....	80
& 623-631	1967	1.4	GIWGCSGKL	82	18	4	13	1	3	....	82	....	82
& 624-632	1962	1.4	IWGCSGKLI	81	19	4	13	1	3	....	81	....	81
& 625-633	1969	1.0	WGCSGKLIC	86	14	4	9	<1	2	....	86	....	86
& 626-634	1966	1.4	GCSGKLICT	81	19	4	14	1	2	....	81	....	81
& 627-635	1963	1.4	CSGKLICTT	81	19	4	14	1	2	....	81	....	81
& 628-636	1903	2.8	SGKLICTTA	49	51	19	30	2	4	....	49	........T	19
& 629-637	1902	2.8	GKLICTTAV	49	51	19	30	2	4	....	49	.......T.	19
& 630-638	1901	2.8	KLICTTAVP	49	51	19	30	2	4	....	49	......T..	19
& 631-639	1924	2.6	LICTTAVPW	51	49	19	28	2	4	....	51	.....T...	19
& 632-640	1930	2.3	ICTTAVPWN	54	46	20	26	1	2	....	54	....T....	20
& 633-641	1858	4.2	CTTAVPWNA	21	79	20	56	3	5	....	21	...T.....	7
& 634-642	1853	4.4	TTAVPWNAS	21	79	20	56	3	7	....	21	..T......	7
& 635-643	1857	4.1	TAVPWNASW	23	77	21	53	3	5	....	23	.T.......	7
& 636-644	1857	4.1	AVPWNASWS	23	77	21	53	3	5	....	23	T........	7
& 637-645	1873	2.8	VPWNTSWSN	33	67	33	31	3	4	....A....	33	....A....	33
& 638-646	1850	3.3	PWNASWSNK	29	71	29	38	3	5	....	29	....	29
& 639-647	1825	3.9	WNTSWSNKS	25	75	24	48	4	6	..A......	24	..A.....N	<1
+ 640-648	1790	5.3	NASWSNKSL	18	82	17	57	7	13	....	18	.......N.	<1
+ 641-649	1692	6.8	ASWSNKSLD	12	88	8	67	14	23	........E	2	......N.T	<1
+ 642-650	1702	7.3	SWSNKSLDN	4	96	4	77	14	25	.......EQ	2	.....N.TT	0
+ 643-651	1702	7.2	WSNKSLDNI	4	96	4	78	13	24	......EQ.	2	....N.TT.	0

+ 644-652	1700	7.2	SNKSLDNIW	4	96	4	78	13	24	.....EQ..	2	...N.TT..	0
+ 645-653	1643	8.0	NKSLDNIWG	4	96	3	71	22	36	....EQ..N	1	..N.TT..N	0
+ 646-654	1662	8.0	KSLDNIWGN	4	96	3	71	23	36	...EQ..NH	<1	.N.TT..N.	0
+ 647-655	1674	7.9	SLDNIWGNM	4	96	3	74	20	33	..EQ..NHT	<1	N.TT..N..	0
+ 648-656	1692	7.6	LNEIWDNMT	4	96	4	77	15	27	.EQ..NHT.	<1	.TT..N...	0
+ 649-657	1715	6.8	EEIWNNMTW	6	94	5	81	8	17	.Q...HT..	<1	TT.......	0
+ 650-658	1790	5.7	QIWDNMTWM	10	90	9	76	5	11	...NHT...	<1	T..N.....	2
+ 651-659	1828	4.2	IWDNMTWME	19	81	14	63	4	7	..NHT....	<1	..N.....Q	11
+ 652-660	1828	4.2	WDNMTWMEW	19	81	14	63	4	7	.NHT.....	<1	.N.....Q.	11
+ 653-661	1828	4.5	DNMTWMEWE	18	82	12	66	4	8	NHT.....D	<1	N.....Q..	11
& 654-662	1887	3.4	NMTWMEWER	34	66	17	46	3	5	HT.....D.	<1	.....Q..K	17
& 655-663	1895	3.3	MTWMEWERE	34	66	18	45	3	5	T.....D..	<1	....Q..K.	18
& 656-664	1900	3.3	TWMEWEREI	34	66	19	45	2	5	.....D...	3	...Q..K..	19
& 657-665	1876	4.4	WMEWEREID	24	76	16	56	4	8	....D...N	2	..Q..K...	16
& 658-666	1863	4.5	MEWEREIDN	23	77	15	56	5	9	...D...N.	2	.Q..K....	15
& 659-667	1873	4.1	EWEREIDNY	24	76	16	56	4	7	..D...N..	2	Q..K.....	16
& 660-668	1893	3.3	WEREIDNYT	39	61	21	37	3	6	.D...N...	4	..K......	21
+ 661-669	1782	5.5	EREIDNYTG	18	82	9	66	7	13	D...N...S	1	.K......E	2
+ 662-670	1751	6.5	REIDNYTGL	15	85	7	66	12	21	...N...S.	1	K......EI	<1
& 663-671	1782	5.9	EIDNYTGLI	22	78	7	62	9	16	..N...S..	1	......EI.	<1
& 664-672	1783	5.9	IDNYTGLIY	22	78	7	62	8	16	.N...S..H	<1	.....EI..	<1
+ 665-673	1724	7.0	DNYTGLIYT	10	90	6	69	14	25	N...S..HS	<1	....EI..D	0
+ 666-674	1741	6.2	NYTGLIYTL	13	87	9	68	10	18	...S..HS.	<1	...EI..D.	0
+ 667-675	1724	6.8	YTGLIYTLI	7	93	6	76	12	20	..S..HS..	<1	..EI..D..	0
+ 668-676	1718	6.8	TGLIYTLIE	7	93	5	75	13	22	.S..HS...	<1	.EI..D...	0
+ 669-677	1681	7.6	GLIYTLIEE	5	95	5	70	20	31	S..HS....	<1	EI..D...Q	0
+ 670-678	1742	6.6	LIYTLIEES	11	89	5	72	12	20	..HS.....	<1	I..D...Q.	0
+ 671-679	1776	5.4	IYTLIEESQ	19	81	8	67	6	12	.HS......	<1	..D...Q..	0
+ 672-680	1749	6.0	YTLIEESQN	16	84	6	68	10	17	HS.......	<1	.D...Q...	0
+ 673-681	1741	6.0	TLIEESQNQ	16	84	7	67	10	17	S........	7	D...Q....	0
& 674-682	1814	4.5	LIEESQNQQ	29	71	12	54	5	9	....	29	...Q.....	7
& 675-683	1810	4.6	IEESQNQQE	28	72	12	55	5	10	....	28	..Q......	7
& 676-684	1819	4.2	EESQNQQEK	38	62	12	43	6	11	....	38	.Q.......	11
& 677-685	1831	4.0	ESQNQQEKN	40	60	12	44	4	9	....	40	Q........	11
& 678-686	1887	2.4	SQNQQEKNE	67	33	6	24	3	5	....	67	....	67
& 679-687	1863	3.0	QNQQEKNEQ	59	41	8	29	4	6	....	59	....	59
& 680-688	1859	3.6	NQQEKNEQE	49	51	11	36	5	8	....	49	....	49
& 681-689	1895	2.8	QQEKNEQEL	54	46	14	29	3	4	....	54	....	54
& 682-690	1902	2.7	QEKNEQELL	55	45	14	29	2	4	....	55	....	55
& 683-691	1874	3.7	EKNEQELLE	41	59	11	43	4	7	....	41	....	41
& 684-692	1873	3.6	KNEQELLEL	42	58	12	42	4	7	....	42	....	42
& 685-693	1889	3.4	NEQELLELD	42	58	12	43	3	5	....	42	....	42
& 686-694	1847	4.0	EQELLELDK	39	61	11	45	4	8	....	39	....	39
& 687-695	1846	4.0	QELLELDKW	39	61	11	45	4	8	....	39	....	39
& 688-696	1872	3.7	ELLELDKWA	49	51	11	36	4	8	....	49	....	49
& 689-697	1808	3.8	LLELDKWAS	40	60	14	42	4	8	....	40	....	40
& 690-698	1803	3.8	LELDKWASL	40	60	14	42	4	8	....	40	....	40
& 691-699	1801	3.8	ELDKWASLW	40	60	14	41	4	8	....	40	....	40
& 692-700	1796	3.6	LDKWASLWN	46	54	13	36	4	8	....	46	........S	8
& 693-701	1797	3.5	DKWASLWNW	47	53	13	36	4	7	....	47	.......S.	8
& 694-702	1768	3.4	KWASLWNWF	47	53	13	36	4	7	....	47	......S..	8
& 695-703	1632	3.8	WASLWNWFD	40	60	12	44	4	8	........N	6	.....S...	7
& 696-704	1627	3.9	ASLWNWFDI	40	60	12	44	5	8	.......N.	6	....S....	7
& 697-705	1615	4.1	SLWNWFDIT	29	71	12	56	3	7	......N..	5	...S.....	6
& 698-706	1637	4.6	LWNWFDITN	24	76	11	61	4	8	.....N...	5	..S......	5
& 699-707	1612	4.6	WNWFDITNW	24	76	11	61	4	8	....N....	5	.S.......	5
& 700-708	1608	4.6	NWFDITNWL	24	76	11	61	4	8	...N.....	5	S........	5
& 701-709	1627	3.9	WFDITNWLW	29	71	16	52	3	5	..N......	5	....	29
& 702-710	1539	3.9	FDITNWLWY	30	70	14	53	3	5	.N.......	6	....	30
& 703-711	1497	3.8	DITNWLWYI	31	69	14	52	3	5	N........	6	....	31
& 704-712	1289	3.0	ITNWLWYIK	42	58	15	41	2	4	....	42	....	42
& 705-713	1168	3.3	TNWLWYIKI	37	63	16	44	2	5	L	1	....	37
& 706-714	1129	2.5	NWLWYIKIF	54	46	18	26	2	4	.......L.	1	....	54
& 707-715	1141	1.0	WLWYIKIFI	85	15	7	6	1	2	......L..	2	....	85
& 708-716	1125	1.5	LWYIKIFIM	76	24	8	14	2	3	.....L...	2	....	76
& 709-717	1111	1.8	WYIKIFIMI	73	27	7	18	2	3	....L....	2	....	73
& 710-718	1078	1.8	YIKIFIMIV	72	28	8	19	1	3	...L.....	2	....	72
& 711-719	1053	2.1	IKIFIMIVG	68	32	7	24	1	3	..L......	2	....	68
& 712-720	1033	2.1	KIFIMIVGG	68	32	6	25	2	3	.L.......	2	....	68
& 713-721	1020	1.8	IFIMIVGGL	73	27	7	19	1	3	L........	5	....	73
& 714-722	961	2.3	FIMIVGGLI	60	40	19	20	2	4	V	19	....	60

& 715-723	955	2.4	IMIVGGLIG	59	41	19	20	2	5	.......V.	19	....	59
& 716-724	941	2.5	MIVGGLIGL	59	41	18	20	3	6	......V..	18	....	59
& 717-725	925	2.2	IVGGLIGLR	61	39	20	16	3	5	.....V...	20	....	61
& 718-726	931	1.9	VGGLIGLRI	63	37	22	13	2	4	....V....	22	....	63
& 719-727	893	2.5	GGLIGLRIV	50	50	20	28	3	5	...V.....	20	I	13
& 720-728	897	2.2	GLIGLRIVF	53	47	23	21	3	5	..V......	23	.......I.	12
& 721-729	852	3.5	LIGLRIVFT	25	75	23	48	4	7	.V......A	9	......I.A	9
& 722-730	822	3.6	IGLRIVFTV	24	76	22	48	5	7	V......A.	9	.....I.A.	9
& 723-731	813	3.3	GLRIVFTVL	30	70	28	37	5	8	......A..	28	....I.A..	9
& 724-732	806	3.3	LRIVFTVLS	30	70	29	36	5	8	.....A...	29	...I.A...	9
& 725-733	782	3.7	RIVFTVLSI	29	71	24	41	5	10	....A....	24	..I.A....	8
& 726-734	776	4.1	IVFTVLSIV	27	73	20	48	6	10	...A.....	20	.I.A.....	8
& 727-735	768	4.3	VFTVLSIVN	26	74	19	49	6	12	..A......	19	I.A......	8
& 728-736	781	3.8	FTVLSIVNR	28	72	26	40	5	10	.A.......	26	.A.......	26
& 729-737	743	3.8	TVLSIVNRV	29	71	25	41	5	10	A........	25	A........	25
& 730-738	734	2.6	VLSIVNRVR	62	38	6	29	3	6	....	62	....	62
& 731-739	730	3.0	LSIVNRVRQ	52	48	9	37	3	6	....	52	....	52
& 732-740	728	2.4	SIVNRVRQG	60	40	10	28	2	5	....	60	....	60
& 733-741	727	2.4	IVNRVRQGY	60	40	10	28	2	5	....	60	....	60
& 734-742	750	1.7	VNRVRQGYS	73	27	10	16	2	4	....	73	....	73
& 735-743	754	1.5	NRVRQGYSP	76	24	10	12	1	3	....	76	....	76
& 736-744	750	1.4	RVRQGYSPL	78	22	10	11	1	3	....	78	....	78
& 737-745	753	1.4	VRQGYSPLS	77	23	10	12	1	3	....	77	....	77
& 738-746	753	1.7	RQGYSPLSF	70	30	10	18	1	3	....	70	....	70
& 739-747	752	1.7	QGYSPLSFQ	70	30	10	18	1	3	....	70	....	70
& 740-748	748	1.4	GYSPLSFQT	78	22	10	11	1	2	....	78	....	78
& 741-749	729	2.8	YSPLSFQTH	34	66	33	31	2	4	....	34	....	34
& 742-750	710	3.9	SPLSFQTHL	25	75	23	47	5	9	....	25	....	25
& 743-751	706	4.0	PLSFQTHLP	24	76	23	47	6	10	....	24	....	24
+ 744-752	686	5.2	LSFQTHLPA	11	89	10	70	9	16	........T	9	....	11
+ 745-753	670	6.0	SFQTRLPAQ	6	94	5	78	10	21	....H..TP	4	....H...R	3
+ 746-754	666	5.9	FQTRLPTPR	7	93	6	76	11	21	...H.....	4	...H..AR.	3
+ 747-755	650	5.7	QTRLPTPRG	7	93	7	75	11	20	..H......	5	..H..AR..	3
+ 748-756	679	5.8	TRLPTPRGP	7	93	7	75	11	20	.H.......	5	.H..AR...	4
+ 749-757	676	5.9	RLPAQRGPD	7	93	7	75	11	21	H..TP....	5	H...R....	4
+ 750-758	705	4.8	LPTPRGPDR	15	85	11	66	7	14	....	15	..AR.....	8
& 751-759	719	4.0	PAPRGPDRP	20	80	17	59	4	9	.T.......	15	..R......	8
+ 752-760	692	4.5	APRGPDRPE	19	81	14	62	5	11	T........	11	.R.......	5
& 753-761	704	3.3	PRGPDRPEG	37	63	23	37	3	7	....	37	R........	5
& 754-762	707	2.4	RGPDRPEGI	59	41	15	23	3	6	....	59	....	59
& 755-763	702	2.8	GPDRPEGIE	54	46	12	31	3	6	....	54	....	54
& 756-764	708	3.0	PDRPEGIEE	50	50	12	35	3	6	....	50	....	50
& 757-765	706	3.1	DRPEGIEEE	49	51	10	39	2	6	....	49	....	49
& 758-766	694	3.1	RPEGIEEEG	49	51	9	40	2	6	....	49	....	49
& 759-767	697	3.1	PEGIEEEGG	48	52	9	40	2	6	....	48	....	48
& 760-768	689	3.6	EGIEEEGGE	42	58	8	47	3	8	....	42	....	42
& 761-769	715	3.7	GIEEEGGER	39	61	9	48	4	8	....	39	....	39
& 762-770	715	3.8	IEEEGGERD	39	61	9	48	5	9	....	39	....	39
& 763-771	724	3.6	EEEGGERDR	43	57	8	45	4	9	....	43	....	43
& 764-772	723	3.7	EEGGERDRD	45	55	6	45	4	9	....	45	....	45
& 765-773	731	4.2	EGGERDRDR	34	66	14	48	5	11	....	34	....	34
& 766-774	742	3.9	GGERDRDRS	38	62	15	43	4	10	....	38	....	38
+ 767-775	736	5.5	GERDRDRSG	16	84	12	64	8	18	I	2	....	16
+ 768-776	718	6.4	ERDRDRSGP	7	93	6	74	13	25	.......IR	3	....	7
+ 769-777	717	6.2	RDRDRSGPL	8	92	6	74	12	23	......IR.	5	....	8
+ 770-778	716	6.2	DRDRSGPLV	9	91	6	74	10	22	.....IR..	5	....	9
+ 771-779	714	6.7	RDRSGPLVD	6	94	4	76	14	28	....IR..N	<1	........N	4
+ 772-780	714	6.6	DRSGPLVDG	7	93	4	76	13	27	...IR..N.	1	.......N.	4
+ 773-781	716	6.6	RSGPLVDGF	7	93	5	74	14	27	..IR..N.S	<1	......N..	3
+ 774-782	716	6.1	SGPLVDGFL	9	91	7	73	11	22	.IR..N.S.	<1	.....N...	5
+ 775-783	708	6.3	GPLVDGFLA	8	92	6	74	12	24	IR..N.S..	<1	....N....	5
+ 776-784	711	5.9	PLVDGFLAI	8	92	7	76	9	19	R..N.S..L	<1	...N....L	3
+ 777-785	725	5.4	LVDGFLAII	18	82	8	67	7	16	..N.S..L.	<1	..N....L.	3
+ 778-786	726	5.2	VDGFLAIIW	18	82	8	68	7	15	.N.S..L..	<1	.N....L..	8
+ 779-787	724	5.6	DGFLAIIWV	17	83	7	69	7	17	N.S..L..D	<1	N....L...	5
& 780-788	736	4.6	GFLAIIWVD	24	76	9	62	4	10	.S..L..D.	<1	....L....	9
& 781-789	735	4.5	FLAIIWVDL	24	76	10	62	4	10	S..L..D..	<1	...L.....	10
& 782-790	736	4.2	LAIIWVDLR	26	74	13	57	4	9	..L..D...	11	..L......	13
& 783-791	727	4.4	AIIWVDLRS	25	75	11	60	5	10	.L..D....	11	.L.......	11
& 784-792	736	3.8	IIWVDLRSL	26	74	20	51	3	7	L..D.....	14	L........	20
& 785-793	728	3.7	IWVDLRSLC	32	68	17	48	3	7	..D......	17	....	32

& 786-794	729	2.9	WVDLRSLCL	37	63	23	38	2	5	.D.......	23	....	37
& 787-795	730	3.0	VDLRSLCLF	36	64	22	40	2	5	D........	22	....	36
& 788-796	727	2.4	DLRSLCLFS	56	44	18	24	2	5	....	56	....	56
& 789-797	725	2.4	LRSLCLFSY	56	44	18	24	2	5	....	56	....	56
& 790-798	720	2.6	RSLCLFSYH	55	45	18	25	2	6	....	55	....	55
& 791-799	716	2.8	SLCLFSYHR	54	46	17	25	4	7	....	54	....	54
& 792-800	723	2.5	LCLFSYHRL	56	44	17	24	3	6	....	56	....	56
& 793-801	718	2.7	CLFSYHRLR	54	46	17	27	3	7	....	54	....	54
& 794-802	729	2.0	LFSYHRLRD	71	29	8	18	2	5	....	71	....	71
& 795-803	733	2.1	FSYHRLRDL	70	30	8	20	2	5	....	70	....	70
& 796-804	733	2.3	SYHRLRDLL	68	32	8	22	2	5	....	68	....	68
& 797-805	745	1.6	YHRLRDLLL	79	21	2	17	2	4	....	79	....	79
& 798-806	743	1.8	HRLRDLLLI	77	23	3	18	2	5	....	77	L	<1
& 799-807	732	2.2	RLRDLLLIV	70	30	8	20	2	6	....	70	.......LA	0
& 800-808	720	2.6	LRDLLLIVT	53	48	21	23	3	7	....	53	......LAA	0
& 801-809	719	2.6	RDLLLIVTR	53	47	21	23	3	7	....	53	.....LAA.	0
& 802-810	713	3.1	DLLLIVTRI	47	53	19	31	3	8	....	47	....LAA..	0
& 803-811	707	3.3	LLLIVTRIV	43	57	18	35	4	8	....	43	...LAA...	0
& 804-812	700	3.5	LLIVTRIVE	42	58	16	38	4	9	....	42	..LAA....	0
& 805-813	693	3.8	LIVTRIVEL	39	61	16	41	5	10	....	39	.LAA.....	0
& 806-814	696	3.7	IVTRIVELL	40	60	16	39	5	10	....	40	LAA......	0
& 807-815	701	3.7	VTRIVELLG	39	61	17	40	4	9	....	39	AA.......	6
& 808-816	704	3.4	TRIVELLGR	41	59	24	30	5	9	....	41	A........	24
& 809-817	720	2.6	RIVELLGRR	65	35	5	26	4	8	....	65	....	65
& 810-818	712	2.6	IVELLGRRG	64	36	5	27	4	8	....	64	....	64
& 811-819	723	2.1	VELLGRRGW	71	29	5	21	3	6	....	71	....	71
& 812-820	720	2.2	ELLGRRGWE	68	32	6	23	3	5	....	68	....	68
& 813-821	896	3.6	LLGRRGWEA	33	67	20	44	3	7	....	33	I	20
& 814-822	913	3.2	LGRRGWEAL	36	64	24	38	2	5	....	36	.......I.	24
& 815-823	910	3.2	GRRGWEALK	36	64	24	38	3	6	....	36	......I..	24
& 816-824	910	3.1	RRGWEALKY	36	64	24	38	3	6	....	36	.....I...	24
& 817-825	910	3.7	RGWEALKYW	27	73	22	49	2	6	....	27	....I...C	2
& 818-826	910	3.8	GWEALKYWW	26	74	23	49	2	6	....	26	...I...C.	2
& 819-827	911	4.1	WEALKYWWN	25	75	20	53	2	7	....	25	..I...C..	2
& 820-828	911	4.2	EALKYWWNL	25	75	19	53	3	7	....	25	.I...C...	2
& 821-829	911	4.0	ALKYWWNLL	26	74	20	51	3	7	....	26	I...C....	2
& 822-830	920	2.4	LKYWWNLLQ	62	38	7	30	2	4	....	62	...C.....	7
& 823-831	919	2.5	KYWWNLLQY	61	39	7	30	2	4	....	61	..C......	7
& 824-832	924	2.3	YWWNLLQYW	63	37	7	29	1	3	....	63	.C.......	7
& 825-833	907	3.1	WWNLLQYWS	50	50	7	40	2	5	....	50	C........	5
& 826-834	915	2.4	WNLLQYWSQ	63	37	8	27	2	5	....	63	....	63
& 827-835	918	2.2	NLLQYWSQE	65	35	8	25	2	4	....	65	....	65
& 828-836	916	1.9	LLQYWSQEL	70	30	8	20	2	3	....	70	....	70
& 829-837	904	2.1	LQYWSQELK	66	34	8	24	2	4	....	66	....	66
& 830-838	896	2.9	QYWSQELKN	52	48	8	37	3	5	....	52	....	52
& 831-839	860	2.6	YWSQELKNS	56	44	9	33	2	4	....	56	....	56
& 832-840	858	2.6	WSQELKNSA	55	45	9	34	2	4	....	55	....	55
& 833-841	838	3.2	SQELKNSAV	45	55	11	42	2	6	....	45	....	45
& 834-842	837	2.6	QELKNSAVS	54	46	14	30	2	5	....	54	....	54
& 835-843	841	2.6	ELKNSAVSL	55	45	15	29	2	4	....	55	....	55
& 836-844	817	3.5	LKNSAVSLL	39	61	15	44	3	6	....	39	....	39
& 837-845	817	3.6	KNSAVSLLN	39	61	14	43	4	8	....	39	........D	1
& 838-846	813	4.0	NSAVSLLNA	34	66	12	51	3	9	....	34	.......D.	<1
& 839-847	805	3.9	SAVSLLNAT	38	62	11	47	4	10	....	38	......D..	<1
& 840-848	808	3.9	AVSLLNATA	38	62	11	47	4	10	....	38	.....D...	<1
& 841-849	804	3.9	VSLLNATAI	38	62	11	46	4	10	....	38	....D...V	0
& 842-850	825	3.6	SLLNATAIA	47	53	8	41	4	10	....	47	...D...VT	0
& 843-851	833	3.5	LLNATAIAV	49	51	8	40	3	8	....	49	..D...VT.	0
& 844-852	830	3.7	LNATAIAVA	47	53	8	42	4	9	....	47	.D...VT..	0
& 845-853	847	2.9	NATAIAVAE	56	44	12	29	3	7	....	56	D...VT..G	0
& 846-854	855	2.8	ATAIAVAEG	56	44	13	29	3	6	....	56	...VT..G.	0
& 847-855	866	2.0	TAIAVAEGT	63	37	21	14	3	5	....	63	..VT..G..	0
& 848-856	886	1.1	AIAVAEGTD	85	15	4	9	2	3	....	85	.VT..G...	0
& 849-857	885	1.1	IAVAEGTDR	84	16	4	10	2	3	....	84	VT..G....	0
& 850-858	849	2.2	AVAEGTDRI	45	55	38	15	2	5	V	38	T..G.....	0
& 851-859	850	2.1	VAEGTDRII	45	55	39	13	2	4	.......V.	39	..G.....L	0
& 852-860	845	2.2	AEGTDRIIE	43	57	40	15	3	5	......V..	40	.G.....L.	0
& 853-861	810	3.4	EGTDRIIEV	28	72	24	44	3	7	.....V...	24	G.....L..	0
+ 854-862	789	4.9	GTDRVIEVV	15	85	12	68	5	12	....	15	....IL..L	<1
+ 855-863	787	5.0	TDRVIEVVQ	14	86	12	67	6	13	....	14	...IL..LR	0
+ 856-864	781	5.3	DRVIEVVQR	13	87	12	67	7	15	........G	1	..IL..LR.	0

	[+] 857-865	766	6.5	RIIEVLQRA	7	93	6	75	13	24	.V...V.G.	<1	..L...R.I	0
	[+] 858-866	763	7.3	IIEVLQRAG	4	96	3	74	18	34	V...V.G.C	<1	.L...R.IW	0
	[+] 859-867	496	6.7	IEVLQRAGR	7	93	5	67	21	36	...V.G.C.	1	L...R.IW.	0
	[+] 860-868	494	6.6	EVLQRAGRA	7	93	5	67	20	35	..V.G.C..	1	...R.IW..	0
	[+] 861-869	491	6.7	VLQRAGRAI	7	93	5	67	20	37	.V.G.C...	1	..R.IW..F	0
	[+] 862-870	506	6.4	LQRAGRAIL	8	92	5	70	17	32	V.G.C...R	1	.R.IW..F.	0
	[+] 863-871	514	6.0	QRAGRAILH	8	92	8	70	14	26	.G.C...R.	1	R.IW..F..	0
	[+] 864-872	512	6.0	RAGRAILHI	9	91	8	70	14	26	G.C...R..	1	.IW..F...	0
	[+] 865-873	517	5.8	AGRAILHIP	9	91	8	72	11	23	.C...R...	1	IW..F....	0
	[+] 866-874	526	5.8	CRAILHIPT	8	92	6	76	10	21	....R...R	5	W..F....R	0
	[&] 867-875	529	4.3	RAILHIPTR	26	74	13	57	5	11	...R...R.	6	..F....R.	2
	[&] 868-876	529	4.3	AILHIPTRI	25	75	13	57	5	12	..R...R..	6	.F....R..	2
	[&] 869-877	534	4.2	ILHIPTRIR	26	74	13	56	5	11	.R...R...	6	F....R...	1
	[&] 870-878	536	3.6	LHIPTRIRQ	28	72	19	50	3	7	R...R....	6	....R....	19
	[&] 871-879	544	2.5	HIPTRIRQG	36	64	35	28	1	4	...R.....	35	...R.....	35
	[&] 872-880	552	2.5	IPTRIRQGL	39	61	30	30	2	4	..R......	30	..R......	30
	[&] 873-881	556	2.5	PTRIRQGLE	40	60	29	29	2	4	.R.......	29	.R.......	29
	[&] 874-882	554	2.5	TRIRQGLER	39	61	30	29	2	4	R........	30	R........	30
	[&] 875-883	551	1.9	RIRQGLERA	68	32	12	17	3	5	I	2	....	68
	[&] 876-884	551	2.0	IRQGLERAL	68	32	12	17	3	5	.......I.	2	....	68
	[&] 877-885	547	2.2	RQGLERALL	65	35	11	21	3	6	......I..	2	....	65
Nef	[+] 1-9	3651	6.0	MGGKWKRSV	11	89	11	72	5	11	......S..	11	....	11
	[+] 2-10	3605	7.1	GGKWKSSVV	8	92	6	76	10	18	I	3	.....R..P	6
	[+] 3-11	3590	7.1	GKWKSSVVG	8	92	6	75	11	19	.......I.	3	....R..P.	6
	[+] 4-12	3603	7.1	KWKSSVVGW	9	91	6	75	10	18	......I..	3	...R..P..	6
	[+] 5-13	3592	7.5	WKSSVVGWP	9	91	6	72	13	22	.....I...	3	..R..P...	<1
	[+] 6-14	3572	8.1	KSSVVGWPA	8	92	4	70	17	27	....I...T	1	.R..P....	<1
	[+] 7-15	3555	8.5	SSVVGWPAV	6	94	2	72	19	30	...I...T.	1	R..P....I	<1
	[+] 8-16	3589	8.0	SVVGWPAVR	8	92	3	74	15	25	..I...T..	1	..P....I.	<1
	[+] 9-17	4047	8.3	VVGWPAVRE	6	94	3	76	15	25	.I...T...	1	.P....I..	<1
	[+] 10-18	4236	7.3	VGWPAVRER	9	91	4	77	10	18	I...T....	1	P....I...	1
	[+] 11-19	4443	6.0	GWPAVRERM	19	81	7	68	6	11	...T.....	7	....I...T	0
	[+] 12-20	4521	6.5	WPAVRERMR	12	88	7	74	7	13	..T......	7	...I...T.	0
	[+] 13-21	4495	6.9	PAVRERMRR	11	89	6	74	8	15	.T.......	6	..I...T..	0
	[+] 14-22	4500	6.5	AVRERMRRA	11	89	9	72	8	13	T........	9	.I...T...	0
	[&] 15-23	1845	5.7	VRERMRRAE	21	79	8	62	9	15	........-	0	I...T....	0
	[&] 16-24	1860	5.3	RERMRRAEP	29	71	12	50	8	15	.......--	0	...T.....	<1
	[+] 17-25	1613	6.4	ERMRRAEPA	18	82	8	60	14	22	......---	0	..T......	<1
	[+] 18-26	1283	7.0	RMRRAEPAA	12	88	9	59	20	31	.....----	0	.T.......	0
	[+] 19-27	575	7.6	MRRAEPAAE	3	97	3	57	38	51	....-----	0	T.......D	0
	[+] 20-28	418	7.6	RRAPAAEAA	3	97	3	48	46	61	...------	0	...EP.ADG	<1
	[+] 21-29	323	7.3	QTEPAAERT	4	96	2	42	52	66	RA-------	0	RA....DGR	<1
	[+] 22-30	243	7.1	TDPAADVRR	3	97	2	42	53	68	A--------	0	AE....G.A	0
	[+] 23-31	244	7.1	EPAAEERRE	2	98	2	43	52	67	--------.	0	....DG.A.	<1
	[+] 24-32	244	7.0	PAEEERREP	2	98	2	48	48	64	-------..	0	..ADG.A..	<1
	[+] 25-33	245	7.0	AAEERREPA	2	98	2	47	48	64	------...	0	..DG.A...	<1
	[+] 26-34	247	6.8	EEERREPAA	5	95	4	47	45	60	-----....	0	ADG.A....	<1
	[+] 27-35	250	6.9	EERREPAAV	6	94	4	44	47	62	----....D	0	DG.A....A	0
	[+] 28-36	252	6.7	ERREPAAVG	6	94	4	50	40	56	---....DR	0	G.A....A.	0
	[+] 29-37	256	5.9	RTEPAADGV	10	90	6	54	30	42	--.....R.	0	.A....A..	3
	[+] 30-38	257	5.0	AEPAADGVG	13	87	10	59	18	28	-.....R..	0	.....A...	3
	[&] 31-39	4585	3.3	EPAADGVGA	40	60	27	30	3	6	.....R...	<1	....A....	4
	[&] 32-40	4576	3.7	PAADGVGAV	26	74	20	51	3	5	....R...A	<1	...A.....	3
	[&] 33-41	4582	3.4	AADGVGAVS	26	74	22	50	2	3	...R...A.	<1	..A......	4
	[&] 34-42	4578	3.6	ADGVGAVSR	26	74	21	51	2	4	..R...A..	<1	.A.......	4
	[&] 35-43	4570	3.6	DGVGAVSRD	26	74	21	51	2	4	.R...A...	<1	A........	4
	[&] 36-44	4628	2.0	GVGAVSRDL	54	46	31	14	1	2	R...A....	<1	....	54
	[&] 37-45	4604	2.6	VGAVSRDLE	48	52	28	23	1	3	...A.....	28	....	48
	[&] 38-46	4555	4.0	GAVSRDLEK	33	67	18	47	2	4	..A......	18	....	33
	[&] 39-47	4510	4.4	AVSRDLEKH	31	69	14	51	3	6	.A.......	14	....	31
	[&] 40-48	4508	4.1	VSRDLEKHG	32	68	15	50	3	5	A........	15	....	32
	[&] 41-49	4555	3.3	SRDLEKHGA	48	52	16	34	2	4	....	48	....	48
	[&] 42-50	4539	4.0	RDLEKHGAI	42	58	11	44	3	6	....	42	....	42
	[&] 43-51	4553	3.8	DLEKHGAIT	43	57	11	43	3	5	....	43	....	43
	[&] 44-52	4495	4.4	LEKHGAITS	40	60	11	46	3	6	....	40	....	40
	[&] 45-53	4485	4.7	EKHGAITSS	39	61	10	47	4	8	....	39	....	39
	[&] 46-54	4499	4.1	KHGAITSSN	42	58	12	43	3	6	....	42	....	42
	[&] 47-55	4466	3.4	HGAITSSNT	53	47	8	37	2	4	....	53	....	53
	[&] 48-56	4265	3.4	GAITSSNTA	51	49	8	38	2	4	....	51	....	51
	[&] 49-57	4189	4.5	AITSSNTAA	40	60	7	49	4	7	....	40	....	40
	[&] 50-58	4170	5.3	ITSSNTAAT	28	72	11	56	5	9	....	28	........N	11

[&] 51-59	4185	4.6	TSSNTAATN	30	70	14	52	3	7	....	30	.......N.	14
[&] 52-60	4177	4.9	SSNTAATNA	29	71	13	54	5	8	....	29	......N.P	1
[&] 53-61	4182	5.2	SNTAATNAD	26	74	9	60	5	8	........A	4	.....N.P.	2
[&] 54-62	4212	5.1	NTAATNADC	27	73	9	59	5	9	.......A.	4	....N.P..	2
[&] 55-63	4212	5.1	TAATNADCA	27	73	9	59	5	9	......A..	4	...N.P...	2
[&] 56-64	4214	5.2	AATNADCAW	27	73	9	59	5	9	.....A...	4	..N.P....	2
[&] 57-65	4277	4.8	ATNADCAWL	28	72	11	57	4	8	....A....	4	.N.P.....	1
[&] 58-66	4519	4.0	TNADCAWLE	30	70	17	50	3	6	...A.....	6	N.P......	2
[&] 59-67	4574	3.2	NADCAWLEA	49	51	23	25	3	5	..A......	23	.P.......	4
[&] 60-68	4561	3.2	ADCAWLEAQ	48	52	24	26	2	5	.A.......	24	P........	4
[&] 61-69	4558	3.1	DCAWLEAQE	49	51	23	25	2	4	A........	23	....	49
[&] 62-70	4527	3.0	CAWLEAQEE	57	43	15	25	2	4	....	57	....	57
[&] 63-71	1150	4.6	AWLEAQEEE	24	76	13	58	4	10	........-	0	........G	6
[&] 64-72	1145	5.0	WLEAQEEEE	23	77	7	64	6	12	.......-.	0	.......GD	2
[&] 65-73	1144	5.1	LEAQEEEEE	24	76	7	62	7	14	......-..	0	......GD.	2
[&] 66-74	1154	4.7	EAQEEEEEV	28	72	8	58	6	12	.....-...	0	.....GD..	2
[&] 67-75	1159	4.3	AQEEEEEVG	34	66	8	53	6	10	....-....	0	....GD...	2
[&] 68-76	1159	4.3	QEEEEEVGF	34	66	8	52	6	10	...-.....	0	...GD....	2
[&] 69-77	1160	4.2	EEEEEVGFP	34	66	8	53	5	10	..-......	0	..GD.....	2
[&] 70-78	1165	3.6	EEEEVGFPV	43	57	9	43	4	8	.-.......	0	.GD......	2
[&] 71-79	1153	3.9	EEEVGFPVR	39	61	10	47	5	8	-.......T	0	GD.......	3
[&] 72-80	4547	1.9	EEVGFPVRP	71	29	12	17	1	2	.......T.	4	D........	1
[&] 73-81	4572	1.6	EVGFPVRPQ	74	26	12	13	<1	2	......T..	4	....	74
[&] 74-82	4582	1.2	VGFPVRPQV	80	20	12	8	<1	2	.....T...	4	....	80
[&] 75-83	4581	1.2	GFPVRPQVP	80	20	12	7	<1	1	....T....	4	....	80
[&] 76-84	4562	1.3	FPVRPQVPL	78	22	12	9	<1	1	...T.....	4	....	78
[&] 77-85	4513	1.4	PVRPQVPLR	78	22	12	9	<1	2	..T......	4	....	78
[&] 78-86	4511	1.4	VRPQVPLRP	78	22	12	9	<1	2	.T.......	4	....	78
[&] 79-87	4510	1.3	RPQVPLRPM	79	21	12	9	<1	2	T........	4	....	79
[#] 80-88	4570	0.7	PQVPLRPMT	93	7	1	5	<1	1	....	93	....	93
[&] 81-89	4530	1.4	QVPLRPMTY	80	20	9	11	<1	2	....	80	....	80
[&] 82-90	4513	1.6	VPLRPMTYK	78	22	9	12	1	2	....	78	....	78
[&] 83-91	4467	2.6	PLRPMTYKG	42	58	35	21	1	3	........A	35	....	42
[&] 84-92	4472	2.7	LRPMTYKGA	42	58	34	22	1	3	.......A.	34	....	42
[&] 85-93	4419	3.8	RPMTYKGAL	30	70	22	46	2	4	......A.V	22	....	30
[&] 86-94	4422	3.8	PMTYKGALD	30	70	22	46	2	5	.....A.V.	22	....	30
[&] 87-95	4405	4.1	MTYKGALDL	30	70	19	48	3	5	....A.V..	19	....	30
[&] 88-96	4401	4.2	TYKGALDLS	30	70	19	48	3	6	...A.V...	19	....	30
[&] 89-97	4405	4.1	YKGALDLSH	30	70	19	48	3	6	..A.V....	19	....	30
[&] 90-98	4429	3.8	KGALDLSHF	34	66	20	43	2	5	.A.V.....	20	....	34
[&] 91-99	4412	3.7	GALDLSHFL	34	66	20	44	2	4	A.V......	20	....	34
[&] 92-100	4420	3.3	ALDLSHFLK	36	64	27	34	2	4	.V.......	27	....	36
[&] 93-101	4414	3.5	LDLSHFLKE	36	64	27	35	2	4	V........	27	....	36
[&] 94-102	4488	2.3	DLSHFLKEK	67	33	9	23	1	3	....	67	....	67
[&] 95-103	4491	2.3	LSHFLKEKG	68	32	9	22	1	2	....	68	....	68
[&] 96-104	4519	2.0	SHFLKEKGG	72	28	9	18	<1	2	....	72	....	72
[&] 97-105	4521	1.9	HFLKEKGGL	72	28	9	17	<1	2	....	72	....	72
[&] 98-106	4511	2.0	FLKEKGGLE	69	31	9	21	<1	2	....	69	....	69
[&] 99-107	4511	2.0	LKEKGGLEG	70	30	9	20	<1	2	....	70	....	70
[&] 100-108	4506	2.2	KEKGGLEGL	68	32	9	22	1	2	....	68	....	68
[&] 101-109	4482	2.6	EKGGLEGLI	51	49	25	23	1	3	....	51	V	25
[&] 102-110	4449	3.8	KGGLEGLIY	31	69	17	51	2	4	........H	17	.......VH	7
[&] 103-111	4474	3.3	GGLEGLIYS	32	68	18	48	2	3	.......H.	18	......VH.	8
[&] 104-112	4470	3.8	GLEGLIYSQ	28	72	17	53	2	4	......H..	17	.....VH..	8
[+] 105-113	4403	4.9	LEGLIYSQK	18	82	11	68	3	6	.....H..R	6	....VH..Q	<1
[+] 106-114	4404	4.9	EGLIYSQKR	17	83	11	68	3	6	....H..R.	6	...VH..Q.	<1
[+] 107-115	4408	4.8	GLIYSQKRQ	19	81	11	66	3	6	...H..R..	6	..VH..Q..	<1
[+] 108-116	4384	5.3	LIYSQKRQD	17	83	9	70	4	7	..H..R...	5	.VH..Q...	<1
[+] 109-117	4391	5.1	IYSQKRQDI	18	82	10	69	3	6	.H..R....	5	VH..Q....	<1
[&] 110-118	4431	4.2	YSQKRQDIL	27	73	13	58	2	4	H..R.....	8	H..Q.....	2
[&] 111-119	4470	2.9	SQKRQDILD	48	52	17	34	2	3	..R......	17	..Q......	8
[&] 112-120	4470	2.9	QKRQDILDL	48	52	17	34	2	3	.R.......	17	.Q.......	8
[&] 113-121	4470	2.5	KRQDILDLW	48	52	17	34	1	2	R........	17	Q........	9
[&] 114-122	4456	2.2	RQDILDLWV	55	45	18	26	1	2	I	18	I	18
[&] 115-123	4456	2.4	QDILDLWVY	53	47	17	28	1	2	.......I.	17	.......I.	17
[&] 116-124	4450	2.7	DILDLWVYH	45	55	17	37	1	2	......I..	17	......I..	17
[&] 117-125	4476	2.0	ILDLWVYHT	58	42	20	21	<1	2	.....I...	20	.....I...	20
[&] 118-126	4477	2.0	LDLWVYHTQ	58	42	20	21	<1	2	....I....	20	....I....	20
[&] 119-127	4477	2.0	DLWVYHTQG	58	42	20	21	<1	2	...I.....	20	...I.....	20
[&] 120-128	4458	2.6	LWVYHTQGY	46	54	18	35	1	2	..I......	18	..I......	18
[&] 121-129	4449	2.6	WVYHTQGYF	46	54	18	35	1	2	.I.......	18	.I.......	18

& 122-130	4452	2.6	VYHTQGYFP	46	54	18	35	1	2	I........	18	I........	18
& 123-131	4540	1.8	YHTQGYFPD	65	35	14	20	<1	2	....	65	....	65
& 124-132	4552	1.7	HTQGYFPDW	66	34	14	18	<1	1	....	66	....	66
& 125-133	4552	1.5	TQGYFPDWQ	75	25	13	11	<1	1	....	75	....	75
& 126-134	4550	1.8	QGYFPDWQN	73	27	12	14	<1	2	....	73	....	73
& 127-135	4551	1.7	GYFPDWQNY	74	26	12	14	<1	2	....	74	....	74
& 128-136	4551	1.7	YFPDWQNYT	74	26	12	14	<1	2	....	74	....	74
& 129-137	4557	1.4	FPDWQNYTP	82	18	5	13	<1	2	....	82	....	82
& 130-138	4557	1.4	PDWQNYTPG	82	18	5	13	<1	2	....	82	....	82
& 131-139	4555	1.4	DWQNYTPGP	82	18	5	12	<1	2	....	82	....	82
& 132-140	4544	1.4	WQNYTPGPG	82	18	5	13	<1	2	....	82	....	82
& 133-141	4435	3.0	QNYTPGPGI	33	67	30	36	1	3	V	17	....	33
& 134-142	4451	2.6	NYTPGPGIR	38	62	31	30	1	2	.......V.	19	....	38
& 135-143	4427	3.0	YTPGPGIRY	37	63	17	44	1	3	......V..	17	........F	1
& 136-144	4427	3.0	TPGPGIRYP	37	63	18	44	1	3	.....V...	18	.......F.	1
& 137-145	4418	3.0	PGPGIRYPL	37	63	17	44	1	3	....V....	17	......F..	1
& 138-146	4423	3.0	GPGIRYPLT	36	64	18	45	1	2	...V.....	17	.....F..C	<1
& 139-147	4419	3.3	PGIRYPLTF	34	66	18	46	1	3	..V......	16	....F..C.	<1
& 140-148	4419	3.3	GIRYPLTFG	34	66	18	46	2	3	.V.......	16	...F..C..	<1
& 141-149	4416	3.3	IRYPLTFGW	34	66	18	46	1	3	V........	16	..F..C...	<1
& 142-150	4522	2.0	RYPLTFGWC	62	38	23	15	<1	2	....	62	.F..C....	<1
& 143-151	4520	2.2	YPLTFGWCF	58	42	22	19	<1	2	........Y	4	F..C.....	<1
& 144-152	4549	1.5	PLTFGWCFK	79	21	6	14	<1	2	.......Y.	4	..C......	6
& 145-153	4549	1.5	LTFGWCFKL	79	21	6	14	<1	2	......Y..	4	.C.......	6
& 146-154	4556	1.5	TFGWCFKLV	79	21	6	14	<1	2	.....Y...	4	C........	6
& 147-155	4623	1.2	FGWCFKLVP	85	15	4	10	<1	1	....Y....	4	....	85
& 148-156	4606	1.5	GWCFKLVPV	78	22	8	13	<1	2	...Y.....	5	........M	1
& 149-157	4567	2.5	WCFKLVPVE	52	48	24	22	1	2	..Y......	3	.......M.	<1
& 150-158	4532	3.0	CFKLVPVEP	47	53	23	28	2	3	.Y.......	3	......M..	<1
& 151-159	4494	4.0	FKLVPVEPE	38	62	12	47	3	5	Y.......D	1	.....M...	<1
& 152-160	4455	4.9	KLVPVEPEK	26	74	9	61	4	8	.......D.	7	....M....	<1
+ 153-161	4334	5.4	LVPVEPEKV	18	82	10	68	4	8	......D.I	1	...M.....	<1
+ 154-162	4329	5.4	VPVEPEKVE	18	82	9	68	5	9	.....D.I.	1	..M......	<1
+ 155-163	4266	5.8	PVEPEKVEE	17	83	10	67	6	11	....D.I..	1	.M.......	<1
+ 156-164	4249	6.0	VEPEKVEEA	16	84	9	67	8	13	...D.I...	<1	M........	<1
+ 157-165	4232	6.4	EPEKVEEAN	12	88	7	73	8	13	..D.I....	<1	....	12
+ 158-166	4207	6.5	PEKVEEANE	12	88	10	70	8	14	.D.I....K	<1	....	12
+ 159-167	4210	6.2	EKVEEANEG	14	86	10	69	8	13	D.I....K.	<1	....	14
& 160-168	4229	5.5	KVEEANEGE	24	76	7	63	6	10	.I....K..	1	....	24
& 161-169	4228	4.9	VEEANEGEN	30	70	10	56	5	8	I....K...	3	....	30
& 162-170	4276	4.7	EEANEGENN	33	67	11	51	5	9	....K...T	1	....	33
& 163-171	4242	5.8	EANEGENNS	20	80	9	64	7	12	...K...T.	1	....	20
& 164-172	4297	5.2	ANEGENNSL	27	73	14	53	6	10	..K...T..	1	....	27
& 165-173	4331	4.9	NEGENNSLL	27	73	15	53	5	9	.K...T...	1	....	27
& 166-174	4366	4.4	EGENNSLLH	30	70	22	44	4	7	K...T....	1	....	30
& 167-175	4483	3.3	GENNSLLHP	36	64	34	27	3	5	...T.....	2	....	36
& 168-176	4438	4.3	ENNSLLHPM	26	74	24	47	3	6	..T.....V	1	L	1
& 169-177	4428	5.1	NNSLLHPMS	24	76	16	56	4	8	.T.....V.	1	.......L.	1
+ 170-178	4427	5.4	NCLLHPMSQ	16	84	15	65	4	8	TS....V.L	1	.S....L..	<1
+ 171-179	4440	5.0	SLLHPMSLH	17	83	17	63	3	6	.....V...	2	.....L.Q.	<1
& 172-180	4500	3.7	LLHPMSQHG	28	72	25	46	1	3	....V.L..	3	....L....	1
& 173-181	4468	4.3	LHPMSQHGM	23	77	23	53	2	5	...V.L...	3	...L.....	1
+ 174-182	4450	5.0	HPMSQHGMD	20	80	12	65	3	6	..V.L....	2	..L......	<1
+ 175-183	4454	5.0	PMSQHGMDD	20	80	12	65	3	6	.V.L.....	2	.L.......	1
+ 176-184	4413	5.5	MSQHGMDDP	19	81	9	68	4	8	V.L......	2	L........	<1
& 177-185	4490	4.5	SQHGMDDPE	23	77	14	59	3	6	.L.......	14	....	23
& 178-186	4456	4.9	QHGMDDPEK	21	79	9	66	4	7	L.......R	8	....	21
& 179-187	4485	4.3	HGMDDPEKE	28	72	17	51	3	6	.......R.	17	....	28
& 180-188	4486	4.3	GMDDPEKEV	28	72	17	51	3	6	......R..	17	....	28
& 181-189	4484	4.2	MDDPEKEVL	28	72	17	51	3	6	.....R...	17	....	28
+ 182-190	4467	5.5	DDPEKEVLV	14	86	8	74	5	9	....R...E	5	....	14
+ 183-191	4484	4.8	DPEKEVLVW	19	81	12	65	4	7	...R...E.	6	....	19
+ 184-192	4457	5.3	PEKEVLVWK	14	86	10	71	5	9	..R...E.R	5	....	14
+ 185-193	4502	4.9	EKEVLVWKF	15	85	10	71	3	6	.R...E.R.	5	....	15
+ 186-194	4504	4.8	KEVLVWKFD	15	85	10	71	3	5	R...E.R..	5	....	15
& 187-195	4563	3.6	EVLVWKFDS	26	74	20	52	3	4	...E.R...	12	....	26
+ 188-196	4480	4.8	VLVWKFDSR	17	83	15	64	4	7	..E.R....	11	....	17
+ 189-197	4479	4.7	LVWKFDSRL	17	83	15	64	3	6	.E.R.....	11	....	17
+ 190-198	4476	4.8	VWKFDSRLA	17	83	15	64	4	7	E.R......	11	....	17
& 191-199	4470	3.7	WKFDSRLAF	39	61	22	36	3	5	.R.......	22	....	39
& 192-200	4439	4.3	KFDSRLAFH	35	65	20	41	4	7	R........	20	....	35

&	193-201	4466	3.5	FDSRLAFHH	55	45	5	37	3	6	....	55	....	55
&	194-202	4389	4.7	DSRLAFHHM	33	67	17	44	5	9	V	17	V	17
&	195-203	4389	4.7	SRLAFHHMA	33	67	17	44	5	9	.......V.	17	.......V.	17
&	196-204	4379	4.9	RLAFHHMAR	32	68	17	46	5	9	......V..	17	......V..	17
&	197-205	4427	4.2	LAFHHMARE	34	66	20	42	4	7	.....V...	20	.....V...	20
+	198-206	4381	5.4	AFHHMAREL	19	81	12	63	6	10	....V....	12	....V...K	4
+	199-207	4372	5.5	FHHMARELH	19	81	12	63	6	10	...V.....	12	...V...K.	4
&	200-208	4397	5.1	HHMARELHP	20	80	13	62	4	8	..V......	12	..V...K..	5
&	201-209	4416	4.4	HMARELHPE	26	74	15	56	4	6	.V.......	15	.V...K...	6
&	202-210	4405	4.9	MARELHPEY	24	76	14	58	5	8	V........	14	V...K....	5
&	203-211	4477	3.8	ARELHPEYY	40	60	14	42	3	6	........F	5	...K....F	5
&	204-212	4462	3.9	RELHPEYYK	39	61	14	43	4	6	.......F.	5	..K....F.	5
&	205-213	4422	4.6	ELHPEYYKD	23	77	18	55	4	7	......F.N	3	.K....F.N	3
&	206-214	3814	4.2	LHPEYYKDC	25	75	15	56	3	5	.....F.N.	3	K....F.N.	3

^All percentages are shown to the nearest whole number. [a] Amino acid number at the start and end of the nonamer position in the protein alignment. The symbols #, & and + denote the highly conserved, mixed-variable and highly diverse nonamer positions, respectively (see Figure 3 for definitions). Positions with less than 100 sequences are denoted with *. [b] Total number of protein sequences analysed at the respective nonamer position; the differences between nonamer positions was due to the inclusion of both partial and full-length sequences in the alignments. [c] Shannon nonamer entropy (see Figure 2 for details). [d] The index nonamer is the most prevalent sequence at the given aligned nonamer position. [e] Variants differ by one or more amino acids from the index sequence. [f] The major variant is the most common variant sequence at the position. [g] Minor variants are multiple different sequences, each occurring more than once and with an incidence less than or occasionally equal to the major. [h] Unique variants are those that occur only once in the alignment. [i] Nonatypes are the distinct sequences among the variants. [j] HXB2 nonamer sequence and its incidence at the given nonamer position; amino acids identical to the index are denoted as ".". [k] C1P nonamer sequence and its incidence at the given nonamer position; amino acids identical to the index are denoted as ".". HXB2 and C1P nonamers not found at the aligned nonamer position are indicated with 0% incidence.
