# Supplementary material for: Dissecting the Dynamics of HIV-1 Protein Sequence Diversity
Source: PLoS One. 2013 Apr 4;8(4):e59994. doi: 10.1371/journal.pone.0059994 (PMC3617185; doi:10.1371/journal.pone.0059994)
Supplement: Table S4 — Correspondence of concatenated index with HXB2 and C1P sequences. A. Amino acid (aa) level comparisons. B. Nonamer level comparisons. (DOC) [file pone.0059994.s007.doc]

**Table S4 | Correspondence of concatenated index with HXB2 and C1P sequences ^.** **A**. Amino acid (aa) level comparisons. **B**. Nonamer level comparisons.

**A**

| **Protein** | **# HXB2 & Index aa identical** | **# C1P & Index aa Identical** | **# HXB2 & C1P aa Identical** | **# HXB2, C1P & Index aa Identical** |
| --- | --- | --- | --- | --- |
|  | **[%]** | | | |
| Gag | 91 | 88 | 91 | 87 |
| Pol | 98 | 94 | 72 | 92 |
| Vif | 93 | 89 | 72 | 85 |
| Vpr | 73 | 91 | 75 | 70 |
| Tat | 80 | 89 | 86 | 77 |
| Rev | 89 | 84 | 71 | 73 |
| Vpu | 84 | 86 | 87 | 81 |
| Env | 82 | 79 | 80 | 74 |
| Nef | 82 | 85 | 81 | 77 |
| *Proteome* | *88* | *87* | *85* | *82* |

**B**

| **Protein** | **# HXB2 & Index Nonamers identical** | **# C1P & Index Nonamers Identical** | **# HXB2 & C1P Nonamers Identical** | **# HXB2, C1P & Index Nonamers Identical** |
| --- | --- | --- | --- | --- |
|  | **[%]** | | | |
| Gag | 77 | 62 | 58 | 55 |
| Pol | 85 | 60 | 56 | 56 |
| Vif | 58 | 34 | 25 | 25 |
| Vpr | 52 | 58 | 35 | 35 |
| Tat | 48 | 48 | 37 | 30 |
| Rev | 56 | 36 | 31 | 24 |
| Vpu | 28 | 43 | 11 | 11 |
| Env | 47 | 37 | 31 | 27 |
| Nef | 17 | 42 | 21 | 15 |
| *Proteome* | *61* | *49* | *41* | *38* |

**^** All comparisons in these analyses contained more than 100 sequences. # denotes “number of”.
